# Hybridization in birds-of-paradise: Widespread ancestral gene flow despite strong sexual selection in a lek-mating system

**DOI:** 10.1016/j.isci.2024.110300

**Published:** 2024-06-19

**Authors:** Mozes P.K. Blom, Valentina Peona, Stefan Prost, Les Christidis, Brett W. Benz, Knud A. Jønsson, Alexander Suh, Martin Irestedt

**Affiliations:** 1 Department for Evolutionary Diversity Dynamics, Museum für Naturkunde, Leibniz Institute for Evolution and Biodiversity Research, 10115 Berlin, Germany; 2 Department of Organismal Biology – Systematic Biology, Evolutionary Biology Centre, Science for Life Laboratory, Uppsala University, 752 36 Uppsala, Sweden; 3 Ecology and Genetics Research Unit, University of Oulu, 90014 Oulu, Finland; 4 Faculty of Science and Engineering, Southern Cross University, Coffs Harbour, NSW 2450, Australia; 5 Department of Ecology and Evolutionary Biology and Museum of Zoology, University of Michigan, Ann Arbor, MI 48108, USA; 6 Natural History Museum of Denmark, University of Copenhagen, 1350 Copenhagen, Denmark; 7 Department of Bioinformatics and Genetics, Swedish Museum of Natural History, 114 18 Stockholm, Sweden

**Keywords:** Ornithology, Evolutionary biology, Evolutionary processes, Phylogenetics, Genomic analysis

## Abstract

Sexual selection can directly contribute to reproductive isolation and is an important mechanism that can lead to speciation. Lek-mating is one of the most extreme forms of sexual selection, but surprisingly does not seem to preclude occasional hybridization in nature. However, hybridization among lekking species may still be trivial if selection against offspring with intermediate phenotypes prohibits introgression. Here we investigate this further by sequencing the genomes of nearly all bird-of-paradise (Paradisaeidae) species and 10 museum specimens of putative hybrid origin. We find that intergeneric hybridization indeed still takes place despite extreme differentiation in form, plumage, and behavior. In parallel, the genomes of contemporary species contain widespread signatures of past introgression, demonstrating that hybridization has repeatedly resulted in shared genetic variation despite strong sexual isolation. Our study raises important questions about extrinsic factors that modulate hybridization probability and the evolutionary consequences of introgressive hybridization between lekking species.

## Highlights

• Whole-genome resequencing of nearly all bird-of-paradise species
• 10 museum specimens are intergeneric F1 hybrids
• Ancestral hybridization has led to introgression between many species
• Hybridization and introgression a recurring theme despite strong sexual selection

## Graphical abstract

**Figure:**
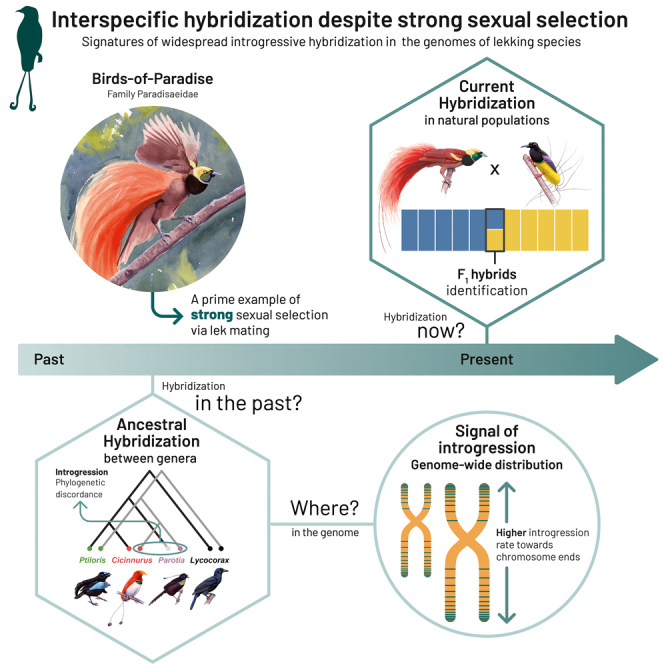

## Introduction

The prevalence of interspecific hybridization and the evolutionary consequences of introgression have been investigated in many organismal groups, ranging from plants^1^ to mammals.^2^ Interspecific hybridization among species can occasionally reassemble existing genetic variation and lead to adaptive change,^3^ but comparative studies across various hybridizing taxa indicate that there is predominantly a negative correlation between the probability of hybridization and the genetic divergence between lineages.^4^ During the initial stages of divergence, the probability of hybridization between sympatric lineages is often high and many incipient species are, as a consequence, likely ephemeral in nature.^5^ Lineages that do persist gradually accumulate traits and loci that reduce gene flow over time, and this process can eventually result in complete reproductive isolation. However, across hybridizing species pairs, the slope of this trajectory can vary and there are several factors that determine the correlation strength between genetic divergence and reproductive isolation.^4^ Sexual selection, for example, can expedite the build-up of reproductive isolation if sexual traits differentiate rapidly during the early stages of species formation.^6^ Similarly, during the latter stages, reinforcement following secondary contact can accelerate reproductive isolation between divergent lineages. The notion that sexual selection can reduce the probability of hybridization is widely supported, and sexual selection has long been considered as a catalyst for speciation across plants and animals.^7^^,^^8^ Therefore, interspecific hybridization and introgression between distinct species with strong sexual isolating mechanisms is thought to be rare.

Lek-mating is one of the most extreme forms of sexual selection, but surprisingly does not seem to preclude occasional interspecific hybridization. In species with lek-mating systems, individuals of the same sex (frequently males) aggregate and engage in competitive displays. In many lekking clades, this has led to a phenotypic radiation in some of the most extravagant ornamental and behavioral traits, and can often markedly differ between closely related species.^9^ These extreme phenotypes tend to be maladaptive, usually serving as an indicator of the physical condition of the lekking sex and are primarily the outcome of strong directional selection driven by female preference.^10^ Consequently, interspecific hybridization and introgression should be selected against since hybrids with intermediate phenotypes do not match the preference for the traits under selection in either parental lineage. However, contrary to expectation, both direct field observations and the description of museum specimens with mosaic phenotypes suggest that hybridization does occasionally take place in the wild. For example, hybridization between lekking capercaillie and black grouse has been recorded on several occasions, and preliminary analyses suggest that backcrossing may also have taken place.^11^ Similarly, research on manakins, another well-studied avian group with lek mating and spectacular male ornamentation, reported that interspecific hybridization may even have led to hybrid speciation.^12^ Based on several avian groups with lek-mating systems, Mayr (1963) posited that hybridization in birds may actually be more common in lekking than non-lekking species due to a lack of pair-bonding prior and post copulation^7^ (though see study by Pierotti and Annett^13^ for opposing view), but this hypothesis remains largely untested. It also remains unclear if interspecific hybridization can lead to introgression among lekking species. Hybrids can be (more) sterile^14^ or strong selection against hybrids may rapidly lead to the purging of introgressed variants over time.^15^

To investigate this further, we focused on an avian radiation that is often regarded as the hallmark example for sexual selection: the birds-of-paradise (Paradisaeidae). Within approximately 10–15 million years, birds-of-paradise radiated from a crow-like ancestor into an extraordinary assemblage of 45 species.^16^^,^^17^ They almost exclusively inhabit the tropical island of New Guinea, but a few species occur on surrounding islands and in the rainforests of northeastern Australia. While one group remained largely “crow-like” (monomorphic and monogamous genera; *Lycocorax*, *Manucodia*, and *Phonygammus* group), an unparalleled diversity in plumage ornamentations and mating rituals can be observed among males within the more species-rich sister group (“core birds-of-paradise”).^9^ Notwithstanding strong female preference for species-specific traits, a few dozen hybrid combinations have been described based on study skins in museum collections; including many instances where the putative parental species are from different genera.^18^ We predict that these study skins are indeed true hybrids and, based on the frequency of hybrids observed in collections, hypothesize that ancestral hybridization may even have led to shared genetic variation between contemporary species. Alternatively, hybrid study skins may have been misdiagnosed or hybridization may take place occasionally but does not lead to effective introgression between lekking species.

To test this hypothesis, we generated whole-genome resequencing data for 40 out of 45 described bird-of-paradise species (1–3 individuals per species) and for 10 historical specimens of putative hybrid origin (Table S1). The five species not included in the present study, either belonged to the “crow-like” outgroup (2 species) or represent recent splits following a taxonomic revision of closely related taxa.^19^ When contemporary fresh tissue samples were not available (50% of the dataset), we took advantage of avian study skins since natural history collections harbor a rich repository of bird-of-paradise specimens. Using a combination of population genetic and phylogenomic approaches, we first investigated whether intergeneric hybridization between radically different forms indeed still takes place in the wild, and then asked whether the genomes of contemporary species carry evidence of past hybridization. We find clear indications that hybridization has been a recurring process throughout the bird-of-paradise radiation, identify multiple instances of introgression and uncover a positive relationship between introgression and the expected recombination rate at a genome-wide scale. At the same time, the Z chromosome has been largely shielded from introgression, inviting further investigation into the role of sex chromosomes in facilitating adaptation or speciation.

## Results and discussion

### Contemporary hybridization between genera

**Figure 1:**
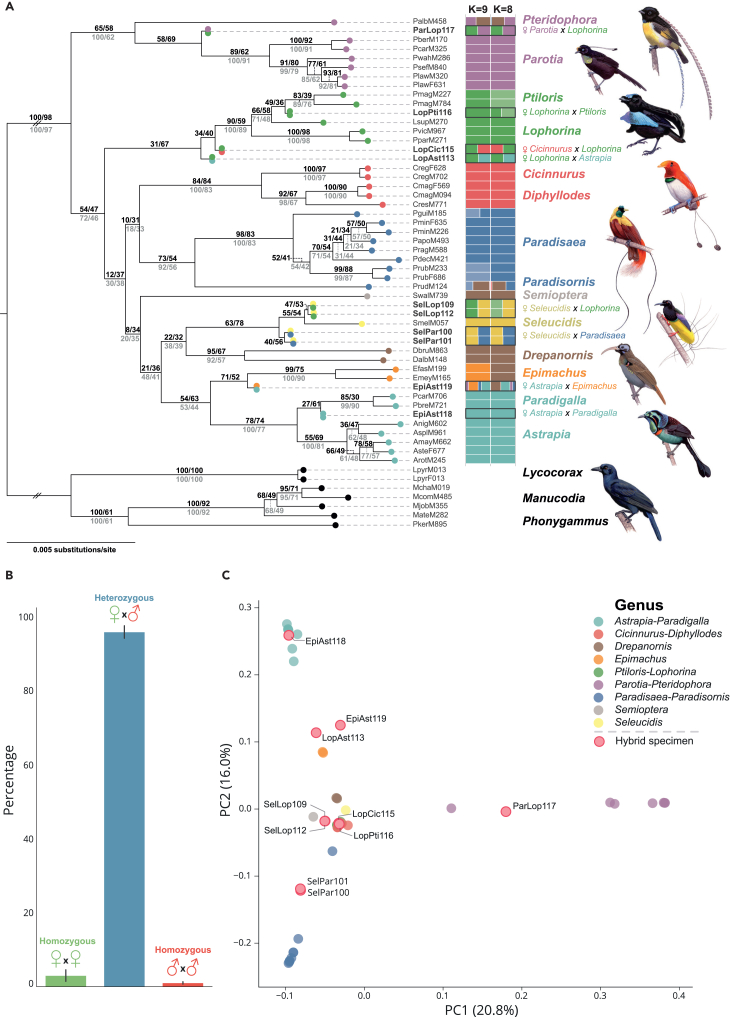
Phylogenomic history, relatedness, and genomic composition of all bird-of-paradise species and 10 recent hybrids (A) Maximum likelihood species tree based on concatenation of 40 kb autosomal genomic windows sampled at 100 kb intervals. Each bipartition is annotated with window and site concordance factors, where the black values above each bipartition correspond to the tree with the 10 recent hybrids included and the gray values below correspond to the same analysis with the 10 recent hybrids excluded. Each individual is highlighted with a color scheme that matches NGSadmix clustering (K = 9) or the major clade that it belongs to. The 10 recent hybrids have been further highlighted with double orbs and a black outline surrounding the horizontal bins. Sex of parent was identified for each hybrid based on mitogenome clustering (Figure S1). (B) The relative frequency distribution of genotypes at ancestrally informative marker sites (AIMs) across all hybrid combinations (mean, ± 1 SD). At AIMs, recent hybrids are almost always heterozygous and carry alleles from each of the parental genera, suggesting that they are all F1 hybrids. (C) PCA based on genotype likelihoods with the placement of the 10 recent hybrids annotated relative to their parental genera. See also Figures S1–S19.

To determine whether intergeneric hybridization takes place between extant lekking bird-of-paradise species, we first assessed the phylogenetic placement and genomic composition of the 10 museum specimens that are putatively of hybrid origin. We sampled autosomal windows across the genome, each 40 kilobases (kb) in length and spaced at least 100 kb apart, and used the resulting window alignments to infer a concatenated maximum-likelihood (IQtree2^20^) and summary-coalescent (ASTRAL-III^21^) species tree. None of the 10 intergeneric hybrids are placed inside a clade that corresponds to a parental genus originally recognized based on morphological characteristics (Figure 1A; Figures S2, S3, S10, and S11). By contrast, in a phylogenetic tree based on complete mitochondrial genomes, the same individuals cluster closely with a specific species that belongs to one of the predicted parental genera (Figure S1). Mitonuclear discordance is consistent with a recent hybridization scenario and reveals the identity of the maternal species for each hybrid combination (Table S2).

Hybrid individuals are intermediately placed between the presumed parental genera in a principal component analysis (PCA, Figure 1B; Figures S19–S24). Admixture analyses^22^ (Figures S25 and S26) support this finding with a roughly equal contribution of each parental cluster at a K-value that recovers most genera (K = 8; Figure 1A). The inferred contribution of parental clusters matches the hybrid combinations as predicted based on morphological characteristics, except for individual *EpiAst118*. Originally suspected to be a hybrid combination between the genera *Epimachus* and *Astrapia*, genetic analyses suggest that this specimen is the outcome of hybridization between *Paradigalla* and *Astrapia* (Figure 1). With parental combinations identified, we then genotyped hybrids at sites throughout the genome with fixed allelic differences between parental genera (ancestry informative marker [AIM] positions^23^^,^^24^). Each hybrid is almost exclusively heterozygous at these specific loci (mean 95.9%; Figure 1B; Figures S27–S35), indicating that each hybrid is an intergeneric F1 cross. These results demonstrate that, notwithstanding strong sexual isolation via female preference, contemporary hybridization still takes place in nature between extant members of genera that widely vary in plumage, courtship display behavior, and lek-type (Tables S1 and S2). Moreover, consistent support for a hybrid origin of all 10 specimens invites further investigation into the genomic composition of other intergeneric hybrid combinations listed in historical collections.^18^

### Phylogenetic discordance

Phylogenomic analyses and population clustering approaches consistently recover monophyletic support for all species belonging to the same genus, but the diversification history between genera is less clear. To investigate phylogenetic relationships and support between all currently described species, we repeated all phylogenetic analyses excluding the 10 F1 hybrids. Irrespective of inference method or data attributes (i.e., 10–40 kb window size or 3,549 ultra-conserved elements [UCEs]^25^), species tree estimation based on autosomal loci largely recovers the same topology; except for the placement of the *Cicinnurus/Diphyllodes* lineage (Figures S2–S17). However, repeating the same phylogenetic analyses with Z-linked loci consistently places *Cicinnurus/Diphyllodes* as sister to *Paradisaea/Paradisornis* and further differs from the autosomal species trees with respect to the placement of *Ptiloris/Lophorina* and *Semioptera* (Figures S2–S17).

Species trees based on distinct phylogenetic methods and chromosome types differ at the intergeneric level, but this uncertainty is not reflected in low bootstrap (consistently 100; Figures S2–S9) or posterior support (consistently 1; Figures S10–S13). However, site (sCF) and window (wCF) concordance factors, which measure the proportion of informative alignment sites or the window trees that support any given bipartition,^26^ tend to be low for the majority of bipartitions deeper in the phylogeny (Figure 1). As expected, but in contrast to bootstrap values, sCF and wCF scale positively with branch lengths (Figure S18). None of the bipartitions that specify relationships between the major clades (which roughly equates to genera) have sCF values over 60 and are frequently below 50, suggesting that most informative sites in an alignment are incongruent for an inferred bipartition of the species tree (Figure 1; Figures S2–S17). The observed topological incongruence between phylogenies (autosomes, Z sex chromosome, and mitogenomes), short internal branches, and the corresponding low CF support is consistent with a Paradisaeidae history that includes periods of rapid diversification and/or interspecific gene flow.

### Genus-wide signatures of ancestral hybridization

**Figure 2:**
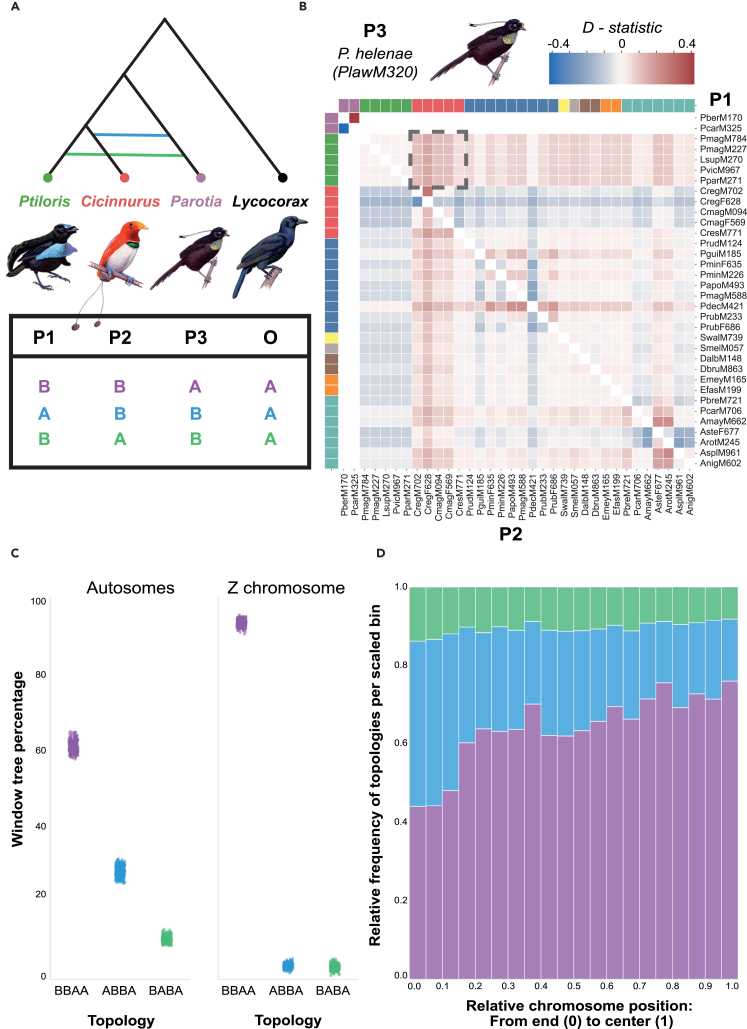
Evidence for hybridization based on four-taxon comparisons (A) Example of a hybridization scenario to be tested between *Parotia* and *Cicinnurus/Diphyllodes* (ABBA) or *Ptiloris/Lophorina* (BABA). (B) Heatmap with Patterson’s *D* statistic between *Parotia helenae* (P3) and all other pairwise combinations of core birds-of-paradise (P1 and P2), in accordance with the species tree (Figure 1A). A dark outline highlights the combinations as presented in (A), but there is consistent support for an excess of allele sharing between *Parotia helenae* and *Cicinnurus/Diphyllodes* relative to all other core birds-of-paradise. The color labels on the side of the matrix match the color scheme used for genera/major clades in (Figure 1A). Patterson’s *D* statistic heatmaps for other *Parotia* as P3 and other putative cases of introgression can be found in supplemental information. (C) Percentage of 10 kb window trees (sampled at 100 kb distance) supporting each of the three possible topologies for the hybridization scenario outlined in (A). Frequency of ABBA, BABA, or BBAA topologies were calculated for each Cartesian combination of individuals belonging to *Ptiloris/Lophorina*, *Cicinnurus/Diphyllodes*, *Parotia*, and two *Lycocorax* individuals (outgroup). In line with the *D-statistic* patterns (B), there is an excess of autosomal window trees supporting a hybridization scenario between *Parotia* and *Cicinnurus/Diphyllodes* (ABBA) in comparison to *Parotia* and *Ptiloris/Lophorina* (BABA). This imbalance in window tree frequencies is not present on the Z chromosome. (D) Summed across the 10 macrochromosomes for all Cartesian combinations, the imbalance between ABBA and BABA topologies is amplified toward the telomeres, suggesting that introgression is unequally distributed across chromosomes. See also Figures S49–S107.

Using Patterson’s *D*-statistic as originally proposed,^27^ by comparing the frequencies of ABBA-BABA site patterns (Dsuite^28^), consistent support for ancestral hybridization was observed between several genera (Figure 2; Figures S36–S48). For example, irrespective of the triad tested, allele sharing between all *Parotia* and almost all *Cicinnurus/Diphyllodes* species (*Diphyllodes respublica* excluded) suggests that the ancestors of these two genera hybridized after they split from all other core bird-of-paradise lineages (Figures S36 and S37). Similarly, all *Ptiloris/Lophorina* species carry alleles that are distinct from other core bird-of-paradise species and likely introgressed from a (possibly extinct) ghost lineage (Figures S36 and S37). *Epimachus*, a genus with only two species, shares more alleles with both *Drepanornis* species than with *Astrapia*; its well-supported sister group (Figure S45). Genus-wide concordance in Patterson’s *D* is a strong indicator of ancestral hybridization, but some erratic species-specific patterns suggest that more recent hybridization may have also taken place.

Patterson’s *D*-statistic was introduced to identify allele sharing between closely related populations, but instances of hybridization between bird-of-paradise genera include species that diverged 5–15 Mya.^16^^,^^17^ Recurring mutations may have a negative effect on the identifiability of introgression and may even increase the false-positive rate if substitution rates vary between lineages.^29^ At a phylogenetic scale, comparing the relative frequencies of competing four-taxon gene tree topologies provides a promising alternative to identify instances of ancestral hybridization.^30^ Following a similar sampling approach of genomic windows as done for the phylogenomic analyses, we estimated the topological frequencies of quartet trees (for convenience referred to as ABBA, BABA, and BBAA topologies) for each Cartesian combination of individuals between focal sets of four genera. Four genera combinations were selected to investigate putative instances of hybridization if indicated by Patterson’s *D*-statistic, mitonuclear discordance, or discordance between Z/A (Z-chromosome vs. autosome)-based phylogenies. The topological frequency results are largely concordant with Patterson’s *D*; ancestral hybridization between progenitors of contemporary genera has led to a consistent imbalance in ABBA/BABA topologies (Figure 2; Figures S49–S107). For example, regardless of combination, *Cicinnurus/Diphyllodes* consistently has a higher proportion of window trees where it is sister to *Parotia* (Figures S49–S59). The imbalance in *Ptiloris/Lophorina* topologies is likely caused by hybridization with an unsampled ghost lineage (Figures S59–S73), and *Epimachus* has an excess of topologies where it is more closely related to *Drepanornis* (Figures S101–S107). Moreover, quantifying window-tree frequencies also highlights putative instances of hybridization that were not readily detectable based on Patterson’s *D*-statistic (Figures S74–S100) and illustrates more broadly the challenge of resolving intergeneric diversification order based on autosomal loci alone. For some combinations (e.g., *Epimachus/Astrapia*, *Paradisaea*, and *Cicinnurus*), there is effectively equal support for each of the three competing topologies, even though there is strong support for a single topology on the Z chromosome (Figures S81 and S82). The observed discordance in site and topological frequencies strengthen the notion that incomplete lineage sorting (ILS) is not solely responsible for incongruent coalescent patterns, and that interspecific hybridization has been a recurring process throughout the Paradisaeidae radiation.

### Introgression scales with expected recombination rate

Patterson’s *D*-statistic or four-taxon gene tree frequencies provide a qualitative estimate indicating whether introgressive hybridization has taken place, but is a poor predictor of the amount or localization of introgressed regions across the genome.^31^ However, theoretical models^32^ and empirical work^33^^,^^34^^,^^35^ have both highlighted that the magnitude and spatial distribution of introgression is non-random across the genome and that it often scales with local recombination rate. The avian recombination landscape is highly heterogeneous along and between chromosomes^36^ and this has also been empirically observed in Corvoidea,^37^ the avian superfamily to which birds-of-paradise belong. While local rates of recombination can rapidly change between avian species,^36^ broad-scale recombination patterns at a genome-wide scale are more conserved such as an increase in recombination frequency toward telomeres.^38^^,^^39^ Thus, in concert with a relatively conserved karyotype and genome synteny among birds,^40^ predictions can be made about the rate of introgression at a genome wide scale; even in the absence of a bird-of-paradise-specific recombination map. If introgression scales positively with recombination rate, topologies matching introgression should be more frequently found toward the telomeres and less frequently on the low-recombining Z chromosome.

For all conspicuous examples of intergeneric hybridization among birds-of-paradise, high-recombination regions indeed have a higher proportion of introgressed topologies than low-recombination regions (Figures S49–S73, S83–S91, and S101–S107). Summed across the 10 macrochromosomes, there is a skew in topologies supporting introgression in 20% of the chromosome that is located closest to the telomeres. For example, the number of topologies supporting introgression between *Cicinnurus/Diphyllodes* and *Parotia* is twice as high as the competing alternative (ABBA vs. BABA) toward the telomeres, whereas this difference is negligible in the middle of chromosomes (Figure 2; Figures S49, S53, S56, and S58). This pattern is even more pronounced for combinations that include *Ptiloris/Lophorina*, likely due to additional introgression from a ghost lineage into *Ptiloris/Lophorina* (Figure 2; Figure S59). In parallel, support for introgressed topologies is largely absent on the low-recombining Z chromosome. While a skew in the ratio of ABBA to BABA topologies is caused by introgression, there is also an overall increase in the number of ABBA/BABA relative to BBAA topologies in high-recombination regions. These latter findings are consistent with the theoretical prediction that recombination rate shapes local Ne, and that genome-wide heterogeneity in Ne simultaneously leads to variation in the magnitude of ILS across the genome.^41^

In contrast to the prominent instances of ancestral hybridization, the positive correlation between expected recombination rate and introgression or ILS is erratic for various 4-taxon combinations that span the most contentious region of the phylogeny (Figures S64–S72, S74–S82, and S92–S100). The four bipartitions at the base of the core bird-of-paradise radiation (excluding *Parotia*) have the lowest wCF and sCF scores across the phylogeny (Figure S7B); they coincide with strong incongruence between Z/A topologies and the frequency of competing topologies remains relatively stable along the 10 macrochromosomes. Variation in the tempo and mode of differentiation between Z/A is common in birds^42^ and both the “fast-Z” and “large-Z” effect are often invoked as possible explanations. However, elevated differentiation on the Z chromosome is not necessarily driven by “adaptation” or “reproductive isolation,” and can also be the outcome of accelerated drift due to low Ne.^43^ Moreover, the Z:A ratio of Ne may be even lower than 3/4^44^ in birds-of-paradise since lekking behavior in many species can increase the reproductive variance among males (ZZ) relative to females (ZW). However, not only do the Z/A chromosomes differ in the magnitude of support for a given topology (e.g., Figure S67), in some cases they even strongly support competing topologies (e.g., Figures S64–S66, S80–S82, S92, S97, and S99). A phylogenetic history that includes periods of rapid diversification can result in widespread topological conflict across autosomes,^45^^,^^46^ but drift alone is not expected to result in strong support for competing phylogenetic histories between Z/A. Instead, these patterns are most consistent with a history of extensive hybridization between incipient ancestral lineages and relatively weak selection against introgressed autosomal alleles.^47^ This may also explain why the correlation between recombination rate and introgression/ILS is absent for some of these comparisons. The role of the Z chromosome in facilitating diversification (e.g., via the large/fast-Z effect) throughout the bird-of-paradise radiation should be further investigated.^48^ Most importantly, altogether, these patterns provide further support for the hypothesis that repeated hybridization has led to introgression across the bird-of-paradise radiation and confirm that introgression is non-randomly distributed across the genome.

### Hybridization dynamics and evolutionary implications

Our study produces strong evidence that sexual isolation among lekking species does not prohibit contemporary intergeneric hybridization. We sampled 10 putative intergeneric hybrids and all individuals are F1 combinations. While they confirm that contemporary hybridization indeed still takes place, we cannot exclude the possibility that contemporary hybrids are sterile or have a lower fitness due to a mix of less desirable phenotypic traits relative to the parental species. However, the current sampling is biased, since we only included males that were already flagged as putative hybrids. If backcrossing does take place, the resulting offspring would be phenotypically similar to one of the parental species and forthcoming males may not have been flagged as hybrids. Moreover, female hybrids are even harder to detect since interspecific differences between females are less conspicuous. Either way, these results call for a more extensive survey of (female) hybrids in historical collections and re-emphasize the question why strong sexual selection does not prevent interspecific hybridization between contemporary lek-mating species. Due to the small number of bird-of-paradise species with pair-bonding, it remains challenging to directly test Mayr’s hypothesis^7^ and a comparative study is needed that contrasts hybridization rates within lekking and non-lekking clades. However, there are many birds-of-paradise species that engage in lek-mating, and species from different genera frequently have overlapping contemporary distributions.^18^ Yet, we found no signal of admixture between species from different genera (Figure 1A; Figure S25B) and there is strong genome-wide support (sCF/wCF) for the monophyletic grouping of each genus (Figure 1A; Figures S6 and S7), which collectively suggests that contemporary intergeneric hybridization is rare and/or does not result in introgression. Mayr’s theory on pair-bonding does not provide a prediction regarding the frequency of interspecific hybridization, nor the plausibility of introgression, and perhaps the seemingly haphazard frequency of hybridization is determined by external factors instead. A recent survey quantifying hybridization dynamics across suboscine birds (1,300+ species) identified several factors that predict the degree of introgression.^49^ They found a positive association between introgression signal and variables such as geographic overlap and climate stability. With a dynamic climatic history and heterogeneous landscape, including alpine highlands and tropical lowlands, New Guinea has played an important role in the demographic history of many species.^50^^,^^51^ This includes the birds-of-paradise as well and many can only be found within a specific altitudinal range. We hypothesize that while sexual isolation can put an effective brake on hybridization during neutral conditions, these isolating mechanisms may take a step back during periods of environmental change and consequently modulate hybridization probability.^52^ For now, this remains to be tested, but the dynamic geological and climatic history of New Guinea in combination with its rich biodiversity, presents an excellent framework to investigate this further across avian and non-avian taxa.

Irrespective of the determinants of hybridization probability, ancestral hybridization has repeatedly resulted in shared genetic variation and has left imprints in the genomes of contemporary bird-of-paradise species. Lek-mating is a conserved trait shared among all core birds-of-paradise,^9^ and it is unlikely that such a distinct mating strategy independently evolved many times. It is therefore reasonable to postulate that ancestral hybridization and backcrossing took place between species that also engaged in lek-mating. Backcrossing between lekking species ultimately led to introgressed variants that are non-randomly distributed across the genome, and signatures of introgression are more pronounced in regions where the expected recombination rate is elevated. These findings indicate that (many) introduced variants were not deleterious enough to be directly purged via natural or sexual selection. Furthermore, the positive correlation between expected recombination rate and introgression suggests that recombination has been able to decouple mildly deleterious haplotype tracts from other introgressed variants.^32^ A major outstanding question is whether these remaining variants are mostly (nearly) neutral with respect to phenotype or whether ancestral hybridization could have resulted in adaptive introgression.^53^ If the latter is true, ancestral hybridization may have directly contributed to phenotypic diversification by bringing together haplotype blocks from distinct evolutionary lineages^3^^,^^54^^,^^55^; thereby creating a new genetic background for sexual selection to act upon. Thus, our present findings highlight that interspecific hybridization and introgression can still take place despite the presence of strong sexual isolation mechanisms and provide strong impetus to further investigate the evolutionary implications of introgression among lekking species.

### Limitations of the study

Differentiating between introgression and deep-coalescence is challenging,^53^ particularly with the sampling of the present study that includes only one to a few representatives for each species. We therefore did not attempt to explicitly pinpoint introgressed loci, but rather used a quantitative approach where we used several lines of evidence to demonstrate that ancestral hybridization resulted in introgression. For example, by characterizing how the distribution of competing four-taxon topologies differs within and between chromosomes, and how this scales to the expected recombination rate. While this is a reasonable approach to establish whether introgression has taken place, it does not allow us to confidently identify genes or regulatory regions that have been introduced and whether they have persisted due to subsequent selection. More detailed sampling at the population level is needed to identify possible instances of adaptive introgression, and this would also allow a more detailed look at the prevalence of interspecific hybridization within genera (rather than between).

## STAR★Methods

### Key resources table

**Table:** 

REAGENT or RESOURCE	SOURCE	IDENTIFIER
**Deposited data**		

*Lycocorax obiensis* reference genome	Peona et al.^56^	GCA_014706295.1
Whole genome resequencing data	Prost et al.^17^	PRJNA491255
Ultra-conserved elements	Jarvis et al.^25^	UCE-probes-used.fasta.gz
Whole genome resequencing data	This study	PRJEB64275

**Software and algorithms**		

nf-polish v1.0	https://github.com/MozesBlom/nf-polish	https://github.com/MozesBlom/nf-polish
FastQC v.0.11.5	Andrews^57^	https://www.bioinformatics.babraham.ac.uk/projects/fastqc/
SuperDeduper v.1.4	Petersen et al.^58^	https://github.com/dstreett/Super-Deduper
Trimmomatic v.0.36	Bolger et al.^59^	http://www.usadellab.org/cms/?page=trimmomatic
PEAR v.0.9.10	Zhang et al.^60^	https://cme.h-its.org/exelixis/web/software/pear/
BWA-mem v.0.7.17	Li & durbin^61^	https://github.com/lh3/bwa
Picard v.2.10.13	Picard Toolkit^62^	https://broadinstitute.github.io/picard/
Qualimap v.2.2.1	Okonechnikov et al.^63^	http://qualimap.conesalab.org/
ANGSD v.0.9.2	Korneliussen et al.^64^	https://github.com/ANGSD/angsd
Freebayes v.1.1.0	Garrison et al.^65^	https://github.com/freebayes/freebayes
VCFtools v.0.1.16	Danecek et al.^66^	https://vcftools.github.io/index.html
VCFlib v.1.0.0	Garrison et al.^67^	https://github.com/vcflib/vcflib
BLAST v.2.6	Camacho et al.^68^	https://blast.ncbi.nlm.nih.gov/Blast.cgi
Mitobim v.1.8	Hahn et al.^69^	https://github.com/chrishah/MITObim
SeqTK v1.3	https://github.com/lh3/seqtk	https://github.com/lh3/seqtk
BCFtools v1.12	Li et al.^70^	https://github.com/samtools/bcftools
MAFFT v7.4	Katoh et al.^71^	https://mafft.cbrc.jp/alignment/software/
Geneious Pro vR11	http://www.geneious.com/	http://www.geneious.com/
RAxML-ng v0.7	Kozlov et al.^72^	https://github.com/amkozlov/raxml-ng
Nextflow v19.04	https://www.nextflow.io/	https://www.nextflow.io/
nf-phylo	https://github.com/MozesBlom/nf-phylo	https://github.com/MozesBlom/nf-phylo
Samtools v1.12	Li et al.^70^	https://github.com/samtools/bcftools
IQtree2 v2.1.2	Minh et al.^20^	http://www.iqtree.org/
ASTRAL-III	Zhang et al.^21^	https://github.com/smirarab/ASTRAL
PCAngsd v0.981	Meisner & Albrechtsen^73^	https://github.com/Rosemeis/pcangsd
Numpy v1.21.5	https://numpy.org/	https://numpy.org/
Plotly v5.6	https://github.com/plotly/plotly.py	https://github.com/plotly/plotly.py
PLINK v1.9	Purcel et al.^74^	https://www.cog-genomics.org/plink/
CLUMPAK	Kopelman et al.^75^	http://clumpak.tau.ac.il/
Dsuite v0.4	Malinsky et al.^28^	https://github.com/millanek/Dsuite
ete3 v3.1.2	Huerta-Cepas et al.^76^	https://etetoolkit.org/
Admixture v1.3	Alexander et al.^22^	https://dalalexander.github.io/admixture/

### Resource availability

#### Lead contact

Further information and requests for resources and reagents should be directed to and will be fulfilled by the Lead Contact, Mozes P.K. Blom (mozes.blom@gmail.com).

#### Materials availability

This study did not generate new unique reagents.

#### Data and code availability

• Raw whole-genome resequencing data produced for this study have been deposited in the European Nucleotide Archive and can be publicly accessed via project number: PRJEB64275.
• This paper also utilises existing, publicly available data, that we have previously generated. These accession numbers are listed in the key resources table.
• All original code for data processing and analysis are deposited to GitHub: https://github.com/MozesBlom/.
• Any additional information required to reanalyse the data reported in this paper is available from the lead contact, Mozes P.K. Blom *(*mozes.blom@gmail.com*)*, upon request.

### Experimental model and study participant details

#### Animal materials

To study patterns of hybridization among Birds-of-Paradise (Paradisaeidae), we sequenced the genomes of 57 samples; including 1-3 individuals per species and ten intergeneric hybrids (Table S1). In accordance with the IOC World Bird List (https://www.worldbirdnames.org/new/), last accessed June 1st 2023, the taxon sampling covers 40 out of 45 species. Two species missing belong to the monomorphic/monogamous genera outside of the core Birds-of-Paradise (*Lycocorax pyrrhopterus, Manucodia alter*) and three have been recently established due to taxonomic revision of closely related taxa (*Parotia carolae, Lophorina niedda and Lophorina superba*). The majority of genomes in this study have been newly generated, complemented with a few that we have published earlier^17^^,^^48^^,^^56^ including the *Lycocorax obiensis* reference genome. Metadata for all samples (such as Study ID, specimen ID, tissue type, sex, coordinates/locality) are listed in Tables S1 and S2.

Birds-of-Paradise are CITES-listed and exchange of material from Birds-of-Paradise are therefore subject to CITES regulations. However, between CITES institutions, CITES material can be freely exchanged. The Swedish Museum of Natural History is a CITES registered entity and all genetic material in this project are on loan from other CITES institutions (Tables S1 and S2). The material is also legal in relation to the Nagoya Protocol and EU’s ABS Regulation. For historical samples, these regulations do not apply as they have been collected well before 2014. Fresh tissue samples were either collected prior to the ratification of the Nagoya Protocol or are from Papua New Guinea that is no party of the Nagoya Protocol. For fresh samples all required research and ethical permits from national and local authorities are in place and can be requested from the institutions from which these samples have been borrowed.

### Method details

#### Reference genome

Using a combination of long-, linked- and short-read sequencing technologies, we have previously assembled a high-quality reference genome for *Lycocorax obiensis* (LycPyr7.4^56^); one of the monomorphic and monogamous species that together with *Phonygammus* and *Manucodia* form a well-supported sister clade to all other Birds-of-Paradise.^16^ In brief, the resulting contigs were scaffolded into chromosome models using proximity ligation maps and annotated by comparison to the chicken genome. Throughout the present study, we used the entire *Lycocorax obiensis* assembly as a reference and then subsetted the chromosome models for downstream analyses. The unknown contigs and scaffolds were not included because they are all relatively short and repetitive, and therefore error prone particularly when mapping relatively short (historical DNA) fragments. For similar reasons, we also excluded the W chromosome and only considered the Z chromosome for all analyses focused on the sex chromosomes.

#### DNA extraction and sequencing

For fresh tissue samples (n = 24), genomic DNA was extracted with a KingFisher Duo magnetic particle processor (ThermoFisher Scientific) using the KingFisher Cell and Tissue DNA Kit. Library preparation and sequencing of fresh tissue samples was conducted by the Science for Life Laboratory, Stockholm, using the Illumina TruSeq DNA Library Preparation Kit and sequenced on Illumina platforms (HiSeq2500 and HiSeq X). For the historical specimens (n = 33), Genomic DNA was extracted from footpad samples with the QIAamp DNA Micro Kit (Qiagen) following manufacturer instructions. For library preparation of DNA from historical specimens, we followed the protocol of Meyer & Kircher.^77^ In short, library preparation included blunt-end repair, adapter ligation, and adapter fill-in, followed by four independent index PCRs per sample. For more detailed descriptions of laboratory procedures from degraded historical DNA samples see Irestedt et al.^78^ Sequencing libraries from museum samples were pooled and sequenced on Illumina platforms (HiSeq2500 and HiSeq X) by SciLifeLab, Stockholm.

#### Read processing and alignment

We have developed a custom Nextflow pipeline to process and polish sequencing libraries from both modern and historical specimens (nf-polish, v1.0; https://github.com/MozesBlom/nf-polish). Each library was processed by itself, rather than by individual, and evaluated prior to any modification for quality and contamination using FastQC (v.0.11.5^57^). Historical DNA is often fragmented and adapter contamination ('read through') is therefore a common phenomenon. Following quality control, we used SuperDeduper (v.1.4^58^) to remove PCR duplicates and Trimmomatic (v.0.36^59^) to remove adapter sequences. Overlapping read pairs were subsequently merged with PEAR (v.0.9.10^60^), if reads were longer than 30 basepairs (bp) and had at least 20 bp overlap between forward and reverse reads. Moreover, quality scores of the merged reads were updated and followed by a second round of Trimmomatic where we removed 5 bp leading and trailing a 4 bp window if the average PHRED quality of that 4 bp window was below 15. Processed reads shorter than 30 bp were removed. Finally, statistics during each cleaning step (number and length of forward, reverse and merged reads) were calculated.

With a polished read set in place, we then mapped each library independently to the complete *Lycocorax obiensis* reference genome using BWA-mem (v.0.7.17^61^). Read pairs and single-end (e.g. merged) reads were mapped separately for each library and the resulting SAM files sorted by position using Picard (v.2.10.13^62^). We then used Picard to merge all libraries that belonged to the same individual, converted to BAM format and added readgroup information by individual. For each individual, we then calculated mapping statistics using Qualimap (v2.2.1^63^; Table S1).

#### Variant calling & filtering

##### Genotype Likelihoods

We took two independent approaches to variant calling and generated different call sets; each specifically tailored to mimimise biases in downstream analyses and allow for an evaluation of consistency in biological patterns. First, we used a probabilistic approach and calculated Genotype Likelihoods using ANGSD (v0.9.2^64^). While the individuals sequenced in this study tend to have medium- to high-coverage (Tables S1 and S2), we used GL to minimise any possible biases due to DNA damage; assuming that damage patterns are randomly distributed and have a low GL given that it's unlikely that the same site is affected in many DNA molecules (within one individual) and across individuals. Moreover, in contrast to joint hard-calling with a species prior (see below), GL were called independent of our a-priori expectation of species assignment per individual. The GL were used to conduct a Principal Component Analysis (PCA) and to infer admixture proportions. Equivalent analyses were repeated using hard-called variants and we interpreted the concordance between distinct approaches as support that patterns of population structure and admixture proportions are not biased by genotyping method, coverage differences, a-priori species assignment or tissue type.

To calculate genotype likelihoods, we used the indexed and sorted BAM files for all core Birds-of-Paradise (i.e. excl. the outgroups: *Lycocorax*, *Manucodia* and *Phonygammus*) and the GATK genotype likelihood model. Only reads with a mapping quality above 20, sites with a base quality above 10 and a minimum minor allele frequency of 0.05 were considered. Moreover, the minimum number of individuals present at a given site needed to be above 45 (out of 50 core Bird-of-Paradise individuals total), the minimum depth per individual was set at 1x and the minimum overall depth for a given site when summing over all individuals needed to surpass 20x. Only biallelic sites were retained.

##### Hard-calling

The PCA and admixture analyses (Figures S19–S26) based on GL confirmed our a-priori expectation (based on phenotype) of species assignment per individual. Supported by this independent observation, we used Freebayes (v1.1.0^65^) to call variants for each chromosome model of the *L. obiensis* reference genome and used a 'species prior' for joint genotyping. The species prior enables Freebayes to improve variant calling per individual by utilising read information across all samples that belong to the same 'species'. We ran Freebayes on all individuals simultaneously, pure species and putative hybrids, and assigned individuals to either the outgroup or the core Bird-of-Paradise group. All reads were included except for those with a mapping quality below 10. The resulting multi-sample VCF file was filtered using VCFtools (0.1.16^66^) and filtering settings were adjusted based on the downstream analyses. Independent filtering iterations were done depending on the dataset needed (i.e. all individuals, excluding hybrids, etc.) and the filtering settings used are described below per respective analysis. The joint-genotype calls were used to infer PCA's, admixture, phylogenetic and introgression analyses.

In addition to joint-genotype calling with a species prior, we also hard-called variants by individual for the phylogenetic analyses. We used Freebayes to call variants for each individual and all chromosome models of the *L. obiensis* reference genome. All reads were included except for those with a mapping quality below 10. During a first round of filtering, we excluded variants with an allelic balance between 0 and 0.2 and a quality score below 20. This initial round aims to exclude variants with low support and those that likely represent Illumina sequencing errors or DNA damage. We then deconstructed multi-nucleotide polymorphisms ('MNPs') into their respective single-nucleotide polymorphism sets with VCFlib (v1.0.0^67^) and removed indels from the VCF file. In addition to the filtered VCF file, we also created a Mask file in VCF format which includes sites that should be masked during consensus calling. The Mask file includes all sites with an allele frequency below 1 and an allelic balance below 0.8 (heterozygous sites), and a coverage below 3. The coverage was calculated across all sites and monomorphic sites with a coverage below 3 were therefore also excluded. The main objective for variant calling per individual and the filtering settings employed, was to generate a variant call set that is minimally biased by read depth differences between individuals, number of species between genera, while retaining unique substitutions between lineages.

### Quantification and statistical analysis

#### Species tree estimation

##### Mitochondrial genome

We used an iterative baiting and mapping approach to assemble complete mitochondrial genomes (mitogenomes) and inferred a maximum-likelihood phylogeny that included all individuals. We first used BLAST (v2.6^68^) to identify the mitogenome scaffold in the *L. obiensis* genome assembly and used this as a reference to directly map the reads for each individual. However, the resulting consensus sequences were highly fragmented, which is likely caused due to the degree of divergence between the core Bird-of-Paradise species and *L. obiensis*. To retrieve complete mitogenomes, we then opted for a two-step approach and only used the *L. obiensis* mitogenome as a reference seed for a Mitobim analysis. Mitobim (v1.8^69^) is an iterative baiting and mapping strategy where reads for focal individuals are first mapped against a distant reference. While reads will successfully align in more conserved regions, the divergent regions in between are then subsequently reconstructed by multiple additional rounds of mapping. During the latter rounds of mapping, the initial reference seed is no longer considered but regions of overlap between reads and conserved regions are used to extend sequences and close gaps. We used Mitobim to create a draft consensus sequence for each individual using only a subset of 16 million reads, randomly selected using SeqTK (v1.3; https://github.com/lh3/seqtk), due to the computational challenges of using entire readsets. We then used BWA-mem to map the complete read dataset for each individual against its own Mitobim consensus sequence, called variants with Freebayes (to identify possible issues due to read subsetting) and called consensus sequences with BCFtools (v1.12^66^). For variant calling with Freebayes, the ploidy level was set to 1 and only reads with a mapping score higher than 20 were used. Variants with a quality score below 20 and an allelic balance lower than 0.2 were excluded. Consensus sequences were masked at positions with a coverage below 20x or where the coverage exceeded 3 times the mitogenome wide mean. A multiple sequence alignment was subsequently created using MAFFT (v7.4^71^) G-INS-i at default settings. The resulting mitogenome alignment was visually inspected in Geneious Pro (vR11; http://www.geneious.com/). We then inferred a maximum likelihood phylogeny using RAxML-ng (v0.7^72^), with a GTR+G substitution model, and calculated support values using 100 bootstrap iterations (Figure S1).

##### Window-based

To reconstruct phylogenetic history and quantify genome-wide heterogeneity in inheritance patterns, we used Nextflow (v19.04; https://www.nextflow.io/) to create a custom automated and reproducible workflow for phylogenomic inference and evaluation. nf-phylo (https://github.com/MozesBlom/nf-phylo) uses BCFtools (v1.12) and Samtools (v1.12^70^) to call masked consensus sequences per individual, for each scaffold or chromosome, or can directly use consensus sequences that have been called a-priori. Here, we used the hard-called variants by individual and the corresponding mask files. With indel variation removed during filtering, consensus sequences were aligned per chromosome and the resulting chromosome alignments formed the basis for phylogenetic reconstruction. All analyses were run twice, once for the complete dataset (i.e. including the ten recent hybrids) and once for a dataset that only included pure representatives for the currently recognized species (i.e. excluding recent hybrids).

nf-phylo is specifically designed to construct the data matrices required for a range of phylogenomic analyses, can be used to filter alignments for a range of parameters and is flexible with respect to genome size and the computational infrastructure available. We used nf-phylo to subset and filter different sized genomic windows (10, 20, 30 and 40 kilobases (kb)) and sampled genomic windows with 100 kb intervals (resulting in roughly 10,000 autosomal window segments). These windows were filtered and used for species tree inference based on concatenation or individual window trees. Any given window only passed filtering if more than 50% of the alignment columns had at least 50% of the individuals represented, and at least 80% of the alignment rows (individuals) had less than 40% missing data. If a given window did not fulfil these requirements, an adjacent window of the same size was evaluated until a suitable window was identified. The filtering was then repeated at a distance of 100 kb from the previous window. This sampling and filtering strategy resulted in the following alignments (with window length x ranging from 10 - 40 kb): i) filtered window alignments of length x, ii) a filtered concatenated alignment (concatenated windows of length x) for each chromosome and iii) a filtered concatenated alignment (concatenated windows of length x) of all autosomes.

For the concatenated alignments, we used IQtree2 (v2.1.2^20^) to infer a maximum-likelihood species tree using the GTR+I+G substitution model. Finding the optimal substitution model for concatenated data matrices is computationally exhaustive and in most situations does not improve the accuracy of inference much in comparison to the parameter rich GTR+I+G model.^79^ The tree with the highest likelihood score out of 20 tree searches was selected and ultrafast bootstrap support approximation (N=1000) was used to calculate bipartition support.^80^ In addition to concatenated data matrices that combined windows from all autosomes (Figures S2, S3, S6, and S7), the same analyses were repeated for each autosome and the Z chromosome independently (Figures S4, S5, S8, and S9). All phylogenies were inferred based on datasets with or without the recent hybrids.

Maximum likelihood inference based on concatenated data matrices provides a robust estimation of the predominant phylogenetic signal in the alignment but does not account nor accommodate for any underlying discordance between gene and species tree. All maximum-likelihood species trees based on concatenated windows indicated that deep coalescence may be rampant, given the short internal branches between genera, and we therefore inferred a maximum-likelihood window tree for each of the filtered window alignments independently (i.e the same window alignments before they were concatenated). Thus, using a sampling density of one window per 100 kb, we inferred around 10.000 autosomal window trees for each dataset with window length x (x ranging from 10 - 40 kb). Individual window trees were inferred using IQtree2, but ModelFinder Plus^81^ was used to identify the most optimal substitution model for each window alignment. The tree with the highest likelihood score, out of 20 tree searches, was selected. For each window size, the corresponding window trees were then gathered and ASTRAL-III^21^ used to infer a summary-coalescent species tree (incl. local posterior support^82^). This resulted in eight summary-coalescent species trees, each based on window trees with a different underlying alignment length and including (Figures S10 and S11) or excluding (Figures S12 and S13) the recent hybrids.

Finally, using the same window alignments and window trees, nf-phylo integrates IQtree2 to calculate "gene" (in this case, window) and site concordance factors. Gene and site concordance factors summarise the frequency with which the inferred bipartition is recovered in the underlying collection of gene trees or supported by informative site patterns in each alignment. They therefore represent a more nuanced proxy for the underlying discordance than bootstrap support values alone.^26^ Window concordance factors were calculated based on all inferred windows trees and site concordance factors by sampling 100 random quartets around each internal branch. We calculated concordance factors for all different window lengths and annotated those on the corresponding concatenated maximum-likelihood and summary-coalescent species trees (Figures S2–S13). All above-mentioned analyses have been integrated in the nf-phylo workflow and both pipeline and documentation can be found at https://github.com/MozesBlom/nf-phylo.

##### Ultra-conserved elements

Phylogenomic analyses based on windows, provide an exhaustive overview of inheritance patterns across the genome but do not differentiate between coding and non-coding regions. Such windows can therefore include sequences that have been under selection or other constraining processes. We complemented our window-based analyses by also inferring species trees based on a putatively neutrally evolving dataset of ultra-conserved elements (UCEs). 3769 single-copy loci have been previously described as widely shared across birds^25^ and we used BLAST (v2.6.0) to identify the orthologous loci in the *L. obiensis* reference genome. The resulting BLAST hits were filtered using a custom Jupyter Notebook where we excluded loci with more than one well-supported BLAST hit (Identity score > 90%, length > 100 bp and gaps < 10) and retained maximum 1 UCE per gene. We then used samtools (v1.9) and bcftools (v1.8) to call a consensus sequence for each individual, based on the variant and mask call set that were created using Freebayes (with variant calling done per individual) for the window-based analyses (above), and each consensus included 1000 bp. of flanking region on each side of the UCE. Only UCE's that mapped to one of the *L. obiensis* primary chromosome models were included (i.e. those belonging to unplaced scaffolds excluded). With start and end positions anchored to the reference genome, the consensus sequences were combined in multiple sequence alignments per UCE locus and loci belonging to either autosomes (N = 3549) or the Z chromosome (N = 73) concatenated independently. We used IQtree2 to infer a maximum-likelihood tree for each UCE locus, with ModelFinder Plus to identify the most optimal substitution model, and maximum-likelihood species trees based on the concatenated alignments (GTR+I+G) of autosomal or Z linked UCE loci. Summary-coalescent trees based on all autosomal or Z linked UCE trees were inferred using ASTRAL-III. All analyses were run twice, once with (Figures S14 and S15) and without the 10 recent hybrids (Figures S16 and S17), and all trees were annotated with site- and concordance factors using the corresponding UCE datasets. The high degree of congruence between the window- and UCE-based analyses suggests that the possible inclusion of non-neutrally evolving regions in the window-based phylogenetic analyses do not lead to qualitative differences in interpretation of evolutionary history.

#### Introgression analyses

##### Principal component analyses

To explore the major axes of genetic diversity among species within the core Birds-of-Paradise, we ran a number of different Principal Component Analyses (PCA); once for a dataset including the recent hybrids and once for a dataset excluding recent hybrids. Moreover, a PCA for each dataset was inferred based on genotype likelihoods, as well as hard-called variants with a species prior.

Based on the genotype likelihoods calculated with ANGSD, we used PCAngsd (v0.981^73^) to infer a covariance matrix and ascertain the main axes of genetic variation among individuals and species. PCAngsd uses an iterative approach to estimate individual allele frequencies based on genotype likelihoods and therefore takes the uncertainty surrounding low-coverage genotypes into account when estimating the covariance matrix. We created a custom Jupyter Notebook to convert the covariance matrix and plot each individual along the major Principal Component axes. The eigen decomposition function in Numpy (v1.21.5) was used to estimate eigenvalues and scaled so all eigenvalues combined summed up to 100. The first 10 eigenvalues captured well over 80% of the genetic variation for both the analyses including and excluding the 10 recent hybrids (Figure S19). We used Plotly (v5.6; Plotly Technologies Inc.) to visualise the distribution of species along several of the major axes of genetic diversity (Figures S20 and S21).

We subsampled the pre-filtered multi-sample VCF files generated with Freebayes and a species prior. Using VCFtools, non-focal individuals (incl. or excl. recent hybrids) for a given PCA analysis were excluded and the resulting dataset filtered for the following criteria. Sites were removed with more than two alleles, a minor allele frequency below 0.03, a quality cut-off below 20 or when containing indels. Moreover, we employed a missing data cut-off of 0.9, a minimum coverage level per individual between 2 and 150 and a mean depth per site across all individuals between 10 and 50. We then used PLINK (v1.9^74^) to account for linkage disequilibrium between sites and used a window size of 100 kb, a 20 bp step size and an R^2^ threshold of 0.4 for linkage pruning. The pruned dataset was subsequently used to calculate eigenvalues and eigenvectors with PLINK. The first 10 eigenvalues captured well over 70% of the genetic variation for both the analyses including and excluding the 10 recent hybrids (Figure S22). We used Plotly to visualise the distribution of species along several of the major axes of genetic variation (Figures S23 and S24).

##### Admixture analyses

Using the same datasets as generated for the PCA analyses, we employed genetic clustering algorithms to identify the optimal number of clusters and quantify the genetic composition of each individual. Similar as to the PCA analyses, we excluded the species belonging to the non- core Bird-of-Paradise genera (i.e. *Lycocorax, Phonygammus, Manucodia*), and ran each analysis with and without the recent hybrids.

NGSadmix (within ANGSD v.0.921^83^) used the genotype-likelihood call set to estimate both K and ancestry proportions. For each possible value of K (2-10), we ran 10 replicates and gathered the likelihood values from each output file. We then ran CLUMPAK (http://clumpak.tau.ac.il/ ^75^) to determine the optimal value for K. Similarly, we used Admixture (v1.3^22^) and the linkage-pruned variant call set based on hard-called genotypes with a species prior, to identify the optimal number of K and estimate ancestry proportions. We used 10-fold cross-validation error estimation and ran 10 replicates for each possible value of K (2-10). Summed across all replicates, we visualised the ancestry proportions inferred with NGSadmix (Figure S25) and Admixture (Figure S26) for each K using a custom Python script.

##### Ancestry Informative Markers

The 10 hybrid specimens included in this study were explicitly selected because they are putative combinations between parental species from different genera based on morphology. The phylogenetic and clustering approaches (PCA & admixture) suggest that these individuals are indeed the outcome of recent hybridization between the parental species as originally proposed based on phenotypic characteristics (except for *EpiAst118*, which is a combination between *Paradigalla* and *Astrapia* instead). However, based on the PCA and admixture analyses alone, it remains difficult to establish whether these are F1 hybrids or whether backcrossing may have taken place.^84^ Benefiting from the phylogenetic scale of divergence between parental species, we further investigated each combination by genotyping hybrid specimens at sites that are Ancestry Informative.^23^ Ancestry Informative Markers (AIMs) are frequently used in a population context where loci are selected for which parental populations substantially differentiate in allele frequency and the origin of individuals can be determined by focusing on the distribution of genotypes at these informative sites. In a phylogenetic context this can be extended by specifically searching for sites with substitutions where two genera are completely fixed for an alternative allele. At such sites, F1 hybrids should be completely heterozygous, unless AIMs were not completely fixed for alternate alleles, and backcrossing of any sort should directly lead to a skew in the distribution of genotype frequencies.

We first identified AIMs for each putative combination by comparing all individuals between parental genera. The *Lophorina* x *Ptiloris* specimen (*LopPti116*) was excluded from this analysis due to the paraphyletic nature of the *Lophorina - Ptiloris* clade (Figure 1). Hence, identifying AIMs by comparing the two genera would not be phylogenetically appropriate. For the 9 other specimens, we iteratively asked which sites had fixed differences by calculating Fst values for each polymorphic site, across all autosomes, between genera using vcftools (v0.1.1.16). Since *Seleucidis* is a monotypic genus and we only have one individual sequenced, we included the two *Drepanornis* individuals to identify AIMs for each hybrid combination involving *Seleucidis* since it was frequently recovered as belonging to the same cluster (Figures S25 and S26) or identified as sister group. With AIMs identified, we 'genotyped' each of the 9 hybrid specimens by inferring Fst values between the hybrid and each of its parental species. Using a custom Python script, we selected sites with fixed differences between genera and quantified the genotype for the hybrid specimens at each of those sites (Figures S27–S35).

##### Incomplete lineage sorting vs. Introgression

Deep coalescence across the genome, heterogeneity in coalescent histories between sites, is primarily caused by incomplete lineage sorting (ILS) or interspecific gene flow. To differentiate between both hypotheses, we calculated Patterson's *D* (also known as the *D*-statistic or the ABBA-BABA test^27^) for each possible triad given the inferred species tree. In other words only triads where the (((P1, P2),P3),O) topology was compatible with the species tree were assessed. Here we followed the topology recovered based on the maximum-likelihood species tree of the concatenated dataset (Figure S7B). Note, the uncertainty surrounding the exact branching order of the polytomy at the base of the core Bird-of-Paradise radiation (i.e. the placement of *Cicinnurus/Diphyllodes*) is not critical but should be taken into account when interpreting signatures of putative introgression.

To calculate Patterson’s *D*, we first subsetted and filtered the hard-called genotypes (Freebayes, species prior) using VCFtools (v0.1.16). We excluded the 10 contemporary hybrids and only retained sites that were biallelic, a minor allele frequency of 0.03 (i.e. present in more than 1 individual), a missing data cut-off of 0.9, a minimum quality cut-off of 20, a minimum coverage per individual between 2 and 160 and a mean depth across all individuals between 10 and 50. We used Dsuite (v0.4^28^) to calculate Patterson's D between all possible combinations given the inferred species tree. Using a custom Python script, for each possible P3 per genus, we generated a heatmap for all pairwise combinations of P1 and P2 (Figures S36–S48). For a given P3, each combination of P1 and P2 was only tested once since the reverse combination of the same individuals for P1 and P2 will yield the same value for Patterson's *D* (but opposite direction from 0). Nonetheless, each combination of P1 and P2 was plotted in the heatmap to improve the clarity of shared patterns across species.

##### Gene-tree frequency distributions

Patterson's D-statistic is a parsimony method that aims to differentiate between ILS and introgression using site patterns across four individuals. It was initially developed to identify instances of recent hybridization (between human populations), but has also been frequently used to recover ancestral hybridization patterns at deeper evolutionary time scales.^85^ While simulations^86^ suggest that Patterson's D-statistic can still be used to detect intergeneric hybridization among Birds-of-Paradise (<5% nuclear divergence), back mutations and/or lineage specific substitution rates may introduce noise that has an effect on the detectability of past hybridization. In such instances, quantifying the relative frequencies of rooted four-taxon topologies may be more suited since substitution models have specifically been developed to account for variation in substitution rates and back mutations. Under a neutral model of ILS only, the two possible topologies alternative to the species tree should be recovered in equal frequencies and a skew towards one of the two topologies (similar to ABBA or BABA) may be an indication of past hybridization.^30^

Following a similar pipeline design and implementation as nf-phylo, we developed a custom workflow that automates the quantification of four-taxon window topologies across all possible combinations of P1, P2, P3 and an outgroup. In other words, nf-GTs (https://github.com/MozesBlom/nf-GTs) takes a similar input as nf-phylo, estimates consensus sequences, filters alignments and infers trees, but does so for every four-taxon combination of individuals by taking the Cartesian product between four different groups (P1, P2, P3 and an outgroup). nf-GTs can handle the computational complexity and load of such an endeavor by parallelizing each task across nodes in a High-Performance Computing cluster environment and uses custom python scripts to estimate the gene-tree frequencies (BBAA, ABBA & BABA) for each four-taxon combination. For example, using two individuals as outgroup, asking whether *Parotia* (P3; 7 indivs) has an excess of gene trees where it is sister to *Cicinnurus/Diphyllodes* (P1; 5 indivs) in comparison to *Epimachus* (P2; 2 indivs), results in 140 possible Cartesian combinations of individuals. Given a 1.3 Gb genome and a sampling density of 10 kb every 100 kb, nf-GTs will filter over 13,000 alignments for each combination and infers a total of 1.8 million window trees for this specific comparison (exact numbers dependent on filtering and data quality).

We estimated the window-tree frequency distributions for multiple instances of past hybridization or to further evaluate discordances observed between phylogenies (i.e. between the summary-coalescent vs. concatenated maximum-likelihood vs. mitogenome vs. Z chromosome). For each comparison, we used the same VCF and Mask files as generated for the complete phylogeny (see 1.4.2.2), used similar alignment filtering settings and used a window size of 10 kb sampled every 100 kb. IQtree2 was used to infer maximum-likelihood window trees using the most optimal substitution model as identified with ModelfinderPlus. The resulting quartet trees were then parsed, rooted by the known outgroup and the topology scored with ete3 (v3.1.2^76^). For each Cartesian combination, we calculated the (relative) frequency of window trees with either an ABBA, BABA or BBAA topology and we scored autosomes and the Z chromosome independently (Figures S49–S107). Moreover, to visualize the distribution of topologies across chromosomes, we divided chromosomes in bins 1 megabase in length and calculated the relative frequencies of the three possible topologies across all Cartesian combinations.

The spatial distribution of ILS and introgression is shaped by the interaction between drift, selection and linkage between sites. ILS is dependent on the maintenance of polymorphisms between speciation events and inversely proportional to τ/2Ne: The relative time between speciation events (τ) and the effective population size (Ne).^41^ With τ being equal across the genome, the expected distribution of ILS across the genome correlates with local Ne. Local Ne is dependent on both genome-wide Ne, the strength of selection on a given site and/or the extent of linkage to a selected site in its vicinity.^87^ Since the extent of linkage scales with recombination rate, the prevalence of ILS should be lower in regions of low recombination and can therefore vary within and between chromosomes. Similarly, recombination also plays an important role in modulating the expected distribution of introgressed tracts. If species barriers are polygenic and made up of many loci with small effect, hybridization will more often lead to introgression in high-recombination regions where clusters of introduced deleterious alleles can be broken up and largely neutral or beneficial variation are more likely to persist.^88^ Genome architecture and recombination are therefore important predictors of coalescent patterns and can provide a null hypothesis about the expected frequencies of window tree topologies across and between chromosomes. While there is unfortunately no empirical estimate of the recombination landscape for any Bird-of-Paradise species, the avian karyotype and genome synteny among birds tends to be relatively conserved.^40^ The fine scale recombination landscape can rapidly change between species, but broad scale patterns at a genome wide scale are more conserved.^36^^,^^38^ For instance, recombination rate on average tends to be higher away from the centromeres, vary between micro- and macrochromosomes and between autosomes and sex chromosomes.^39^ Indeed, an empirical evaluation of the recombination landscape in the hooded crow (*Corvus corone*^37^) seems to confirm that this is also the case for Corvoidea, the super family to which Birds-of-Paradise also belong. To evaluate whether the expected recombination rate correlates with the spatial distribution of window tree topologies, we focused on 10 macrochromosomes (Chromosome 1 - 10) and scaled each chromosome in bins that correspond to 5% of the chromosome length. We calculated the mid-point of the assembled macrochromosomes and each 5% bin includes a 2,5% chromosomal segment equidistant (left and right) to the center of the chromosome. Across all Cartesian combinations, we then counted the frequency of the four taxon topologies and calculated the proportion of each (ABBA, BABA, BBAA) topology per bin (Figures S49–S107).
