# Supplementary material for: Hybridization in birds-of-paradise: Widespread ancestral gene flow despite strong sexual selection in a lek-mating system
Source: iScience. 2024 Jun 19;27(7):110300. doi: 10.1016/j.isci.2024.110300 (PMC11269930; doi:10.1016/j.isci.2024.110300)
Supplement: Document S1. Figures S1–S107 and Tables S1 and S2 [file mmc1.pdf]

iScience, Volume *27*

Supplemental information

Hybridization in birds-of-paradise: Widespread ancestral gene flow despite strong sexual selection in a lek-mating system

Mozes P.K. Blom, Valentina Peona, Stefan Prost, Les Christidis, Brett W. Benz, Knud A. Jønsson, Alexander Suh, and Martin Irestedt

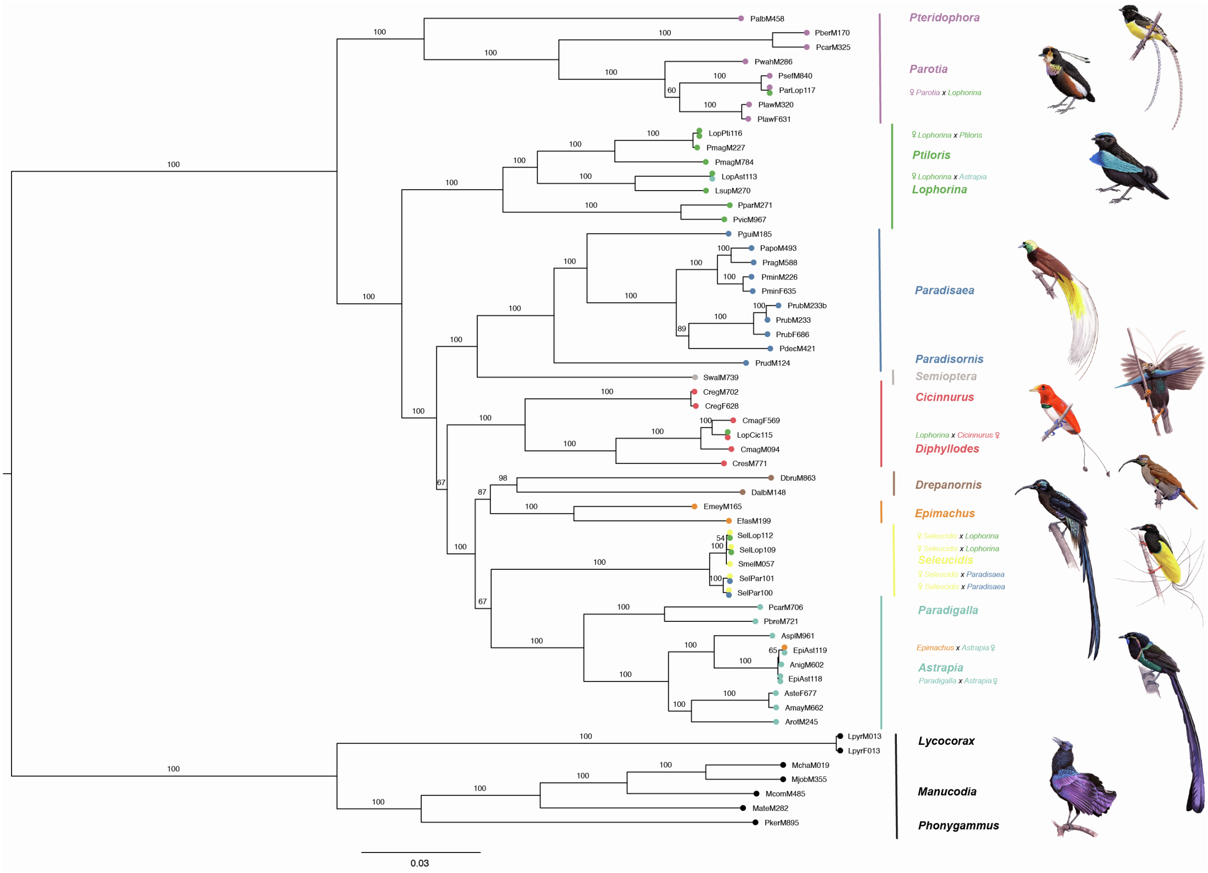

Figure S1. Maximum-likelihood species tree, with bootstrap support, based on whole mitochondrial genomes for all individuals. Related to Figure 1. The 10 recent hybrids have been highlighted with double orbs, with colour fills representing the predicted parental genera based on phenotype.

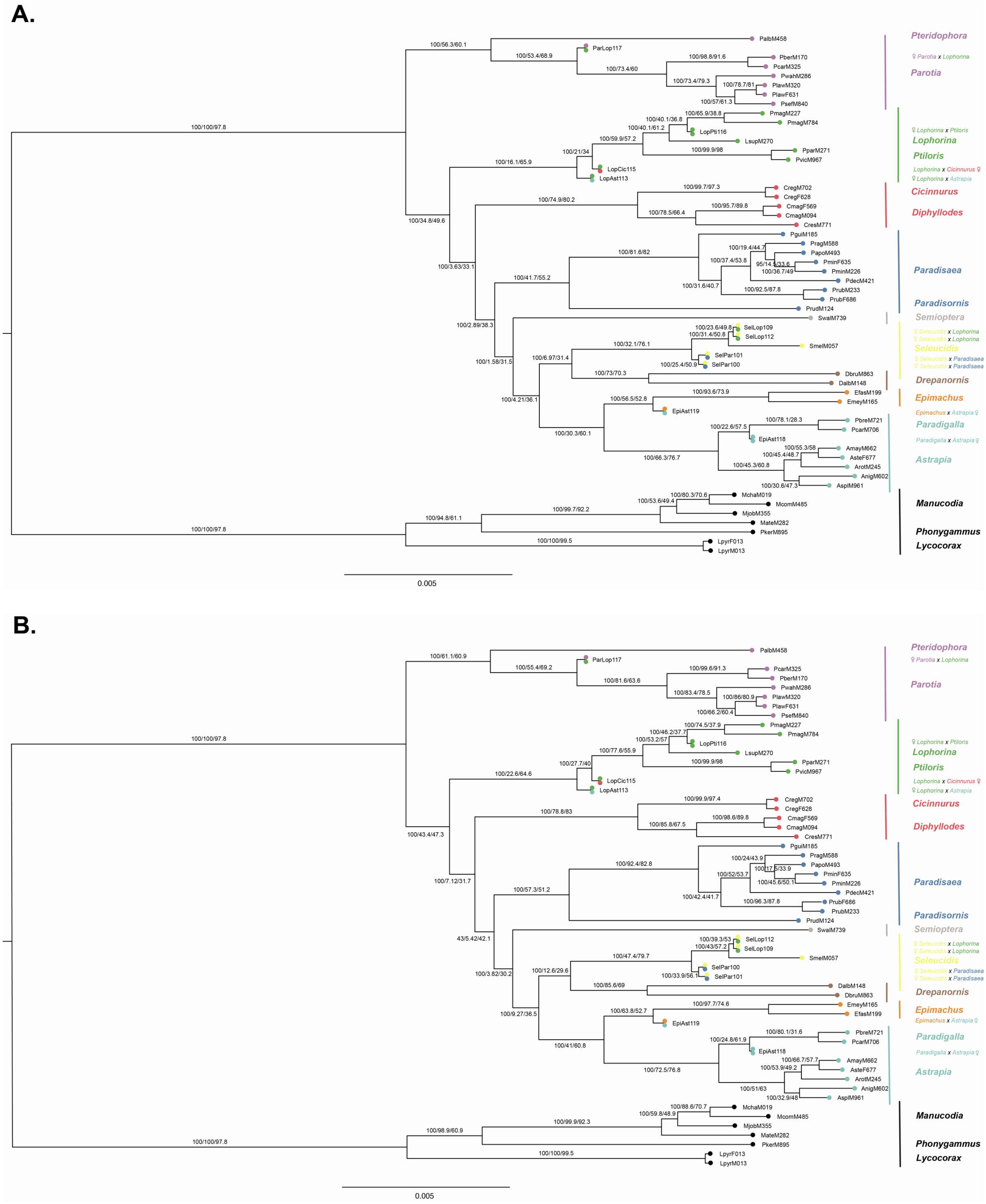

Figure S2. Maximum-likelihood species trees for all individuals, *including* the 10 recent hybrids, based on concatenation of filtered *autosomal* window alignments of length X (sampled every 100 kb). Related to Figure 1. A.) Window alignments of length 10 kb. B.) Window alignments of length 20 kb. All bipartitions are annotated with ultra-fast bootstrap support/window concordance factors/site concordance factors. The 10 recent hybrids have been highlighted with double orbs, with colour fills representing the predicted parental genera based on phenotype, and are often placed as a sister taxon to one of the predicted parental genera.

A.

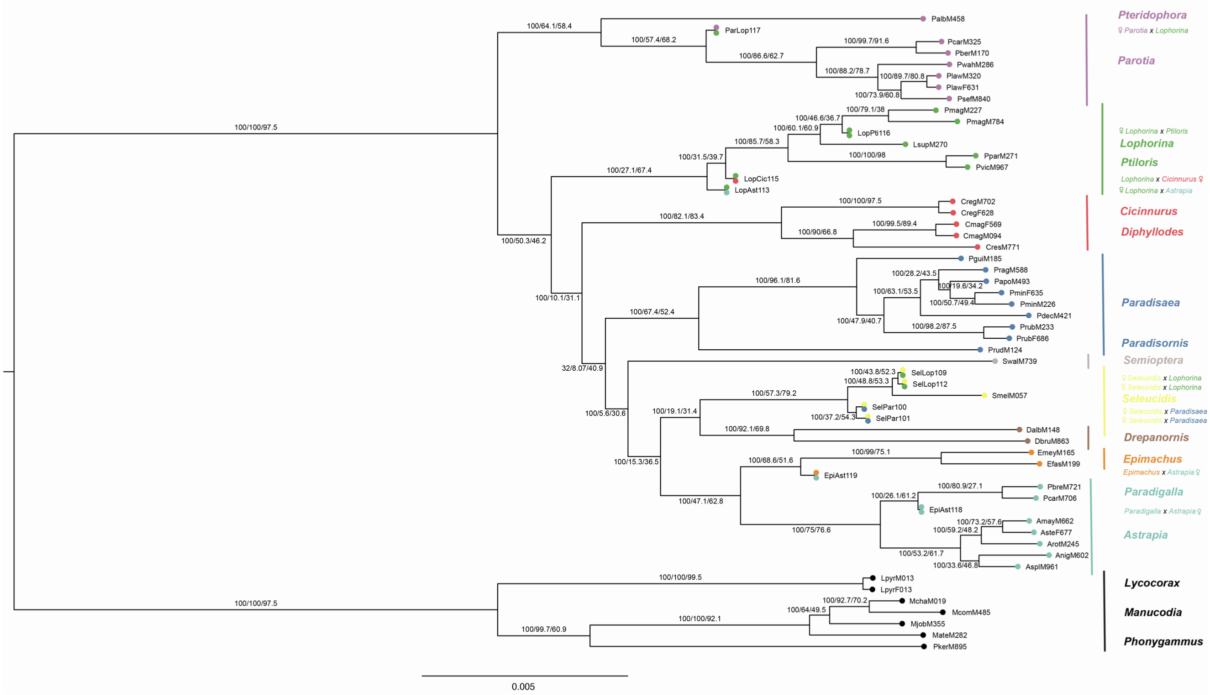

B.

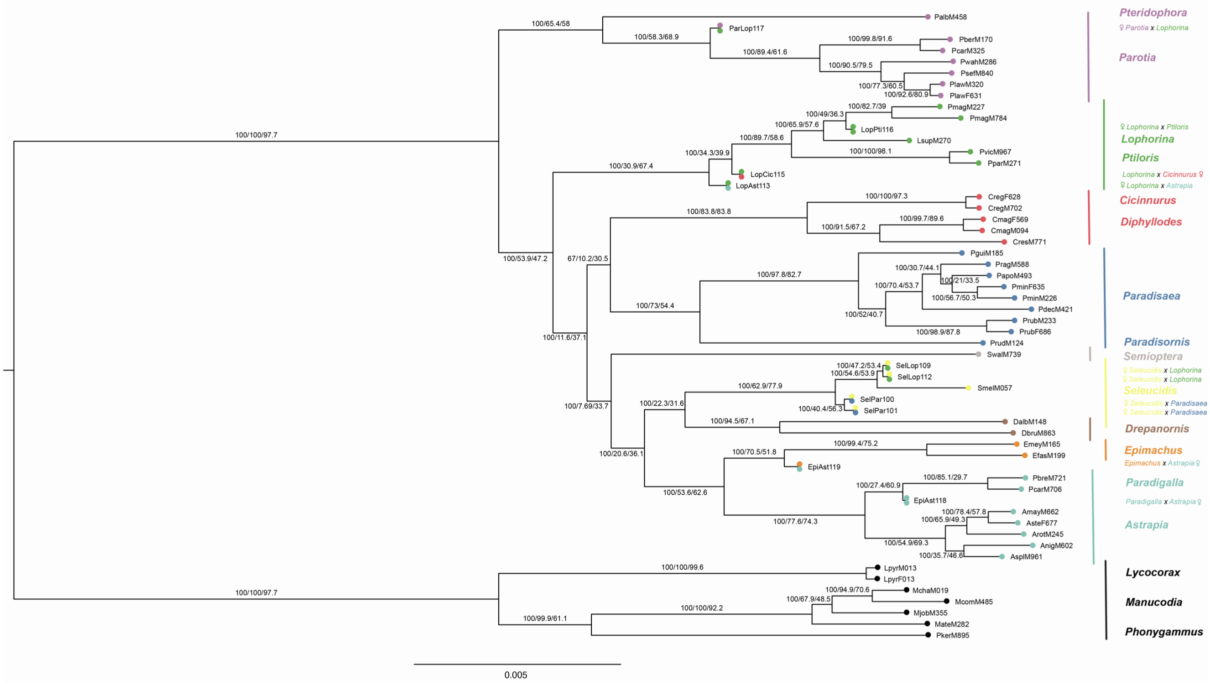

Figure S3. Maximum-likelihood species trees for all individuals, *including* the 10 recent hybrids, based on concatenation of filtered *autosomal* window alignments of length X (sampled every 100 kb). Related to Figure 1. A.) Window alignments of length 30 kb. B.) Window alignments of length 40 kb. All bipartitions are annotated with ultra-fast bootstrap support/window concordance factors/site concordance factors. The 10 recent hybrids have been highlighted with double orbs, with colour fills representing the predicted parental genera based on phenotype, and are often placed as a sister taxon to one of the predicted parental genera.

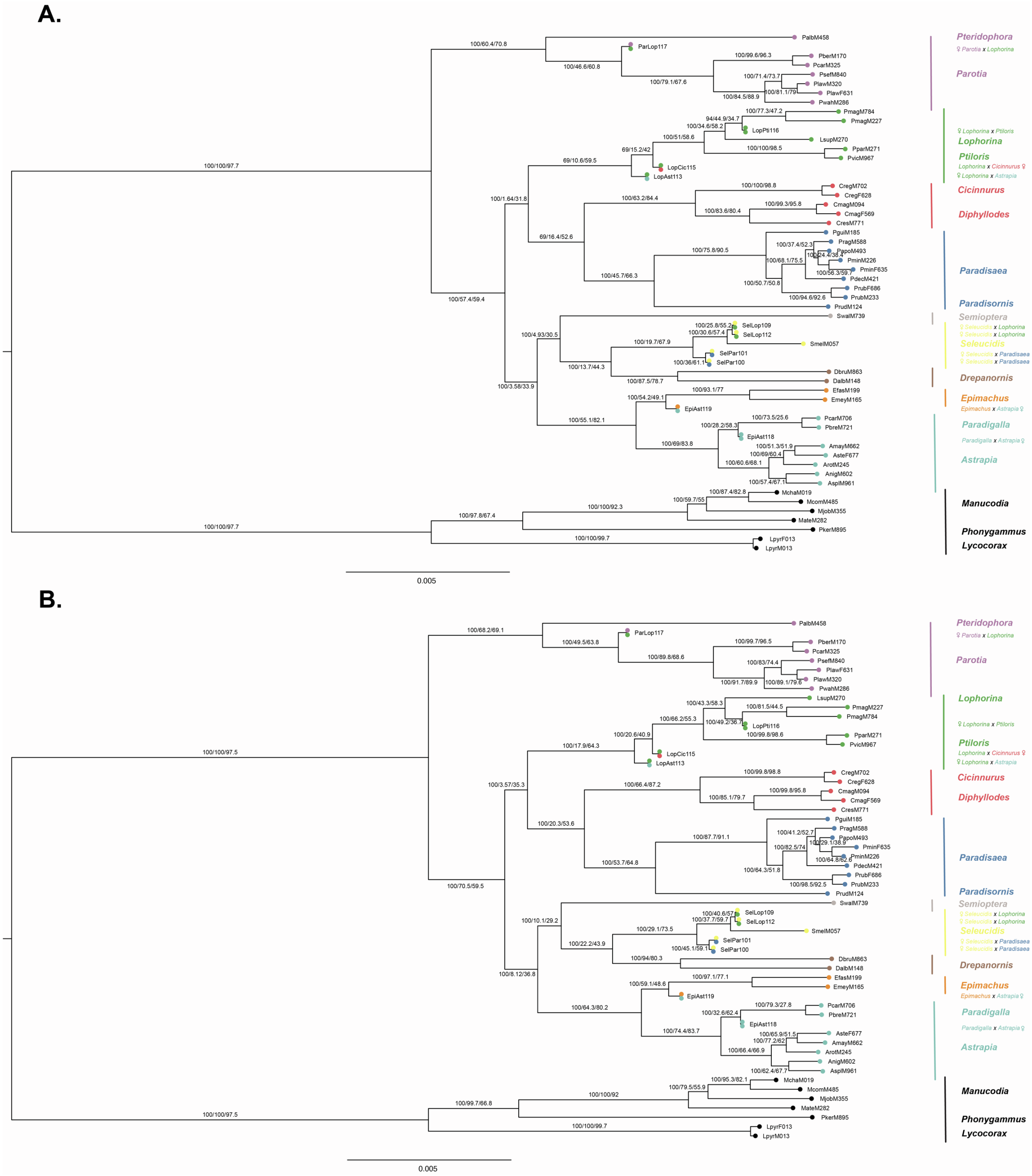

Figure S4. Maximum-likelihood species trees for all individuals, *including* the 10 recent hybrids, based on concatenation of filtered *Z chromosomal* window alignments of length X (sampled every 100 kb). Related to Figure 1. A.) Window alignments of length 10 kb. B.) Window alignments of length 20 kb. All bipartitions are annotated with ultra-fast bootstrap support/window concordance factors/site concordance factors. The 10 recent hybrids have been highlighted with double orbs, with colour fills representing the predicted parental genera based on phenotype, and are often placed as a sister taxon to one of the predicted parental genera.

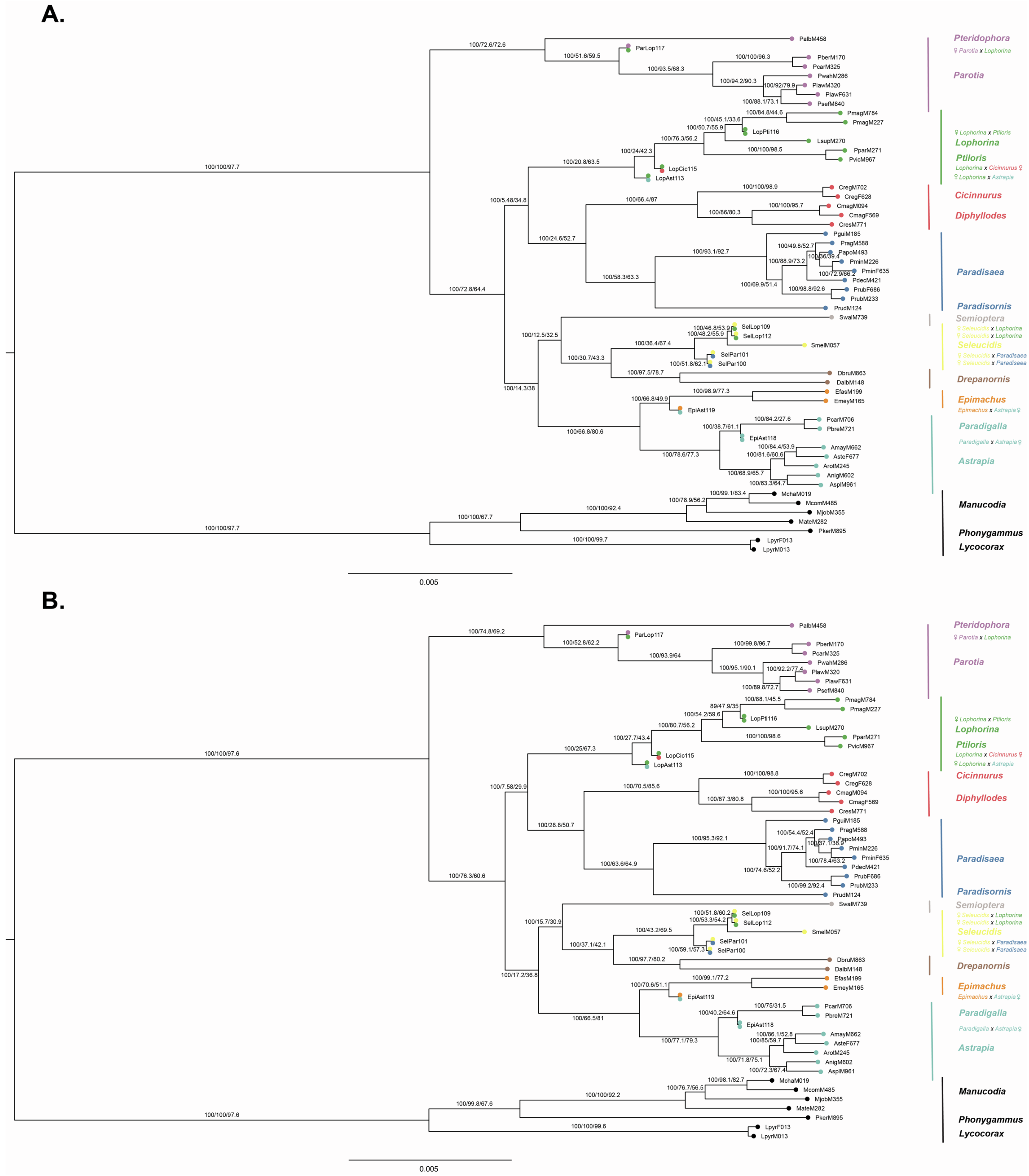

Figure S5. Maximum-likelihood species trees for all individuals, *including* the 10 recent hybrids, based on concatenation of filtered *Z chromosomal* window alignments of length X (sampled every 100 kb). Related to Figure 1. A.) Window alignments of length 30 kb. B.) Window alignments of length 40 kb. All bipartitions are annotated with ultra-fast bootstrap support/window concordance factors/site concordance factors. The 10 recent hybrids have been highlighted with double orbs, with colour fills representing the predicted parental genera based on phenotype, and are often placed as a sister taxon to one of the predicted parental genera.

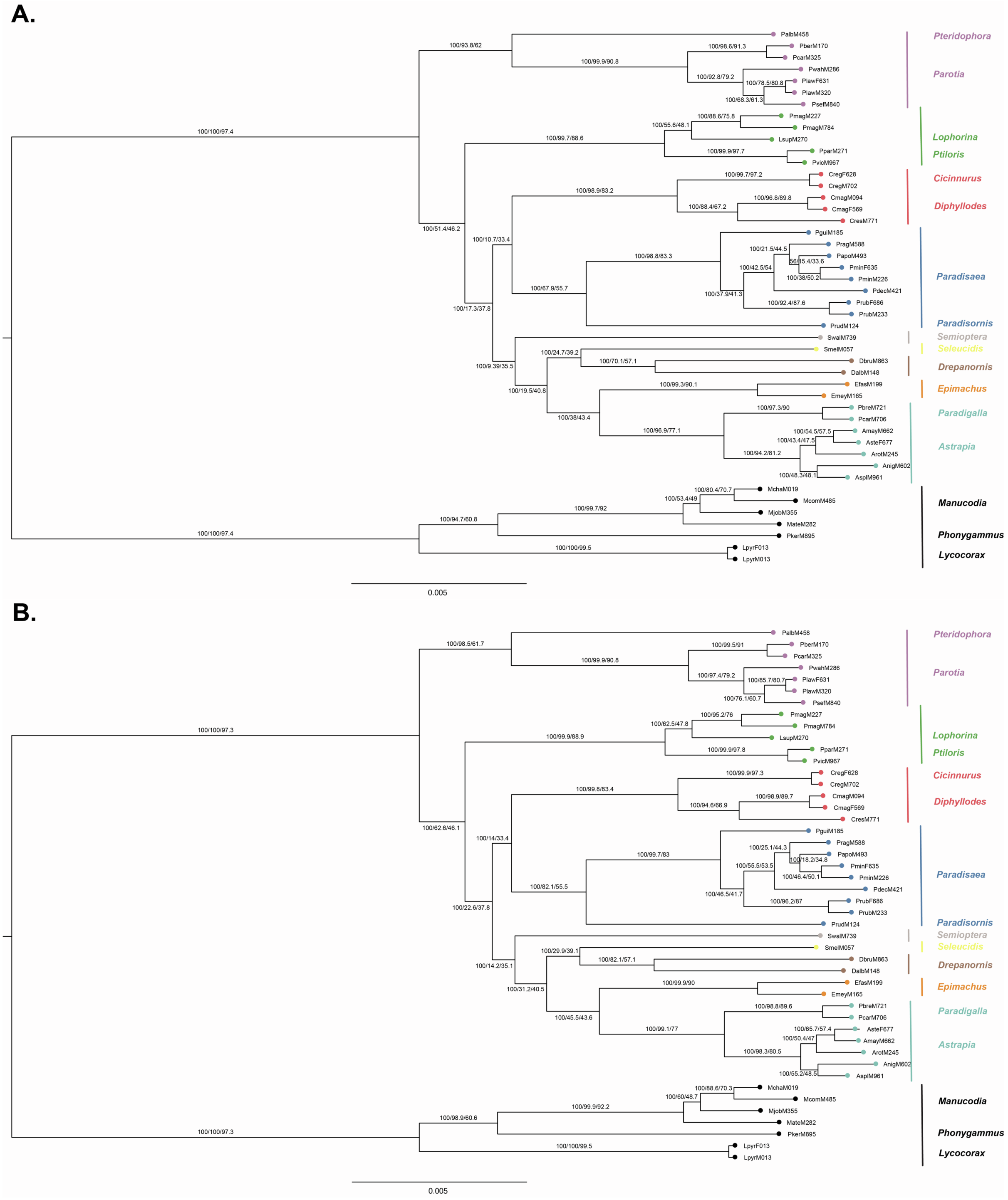

Figure S6: Maximum-Likelihood species trees, *excluding the 10 recent hybrids*, based on concatenation of filtered *autosomal* window alignments of length X (sampled every 100 kb). Related to Figure 1. A.) Window alignments of length 10 kb. B.) Window alignments of length 20 kb. All bipartitions are annotated with ultra-fast bootstrap support/window concordance factors/site concordance factors. All major genera are monophyletic and highly supported, but intergeneric relationships remain largely unresolved.

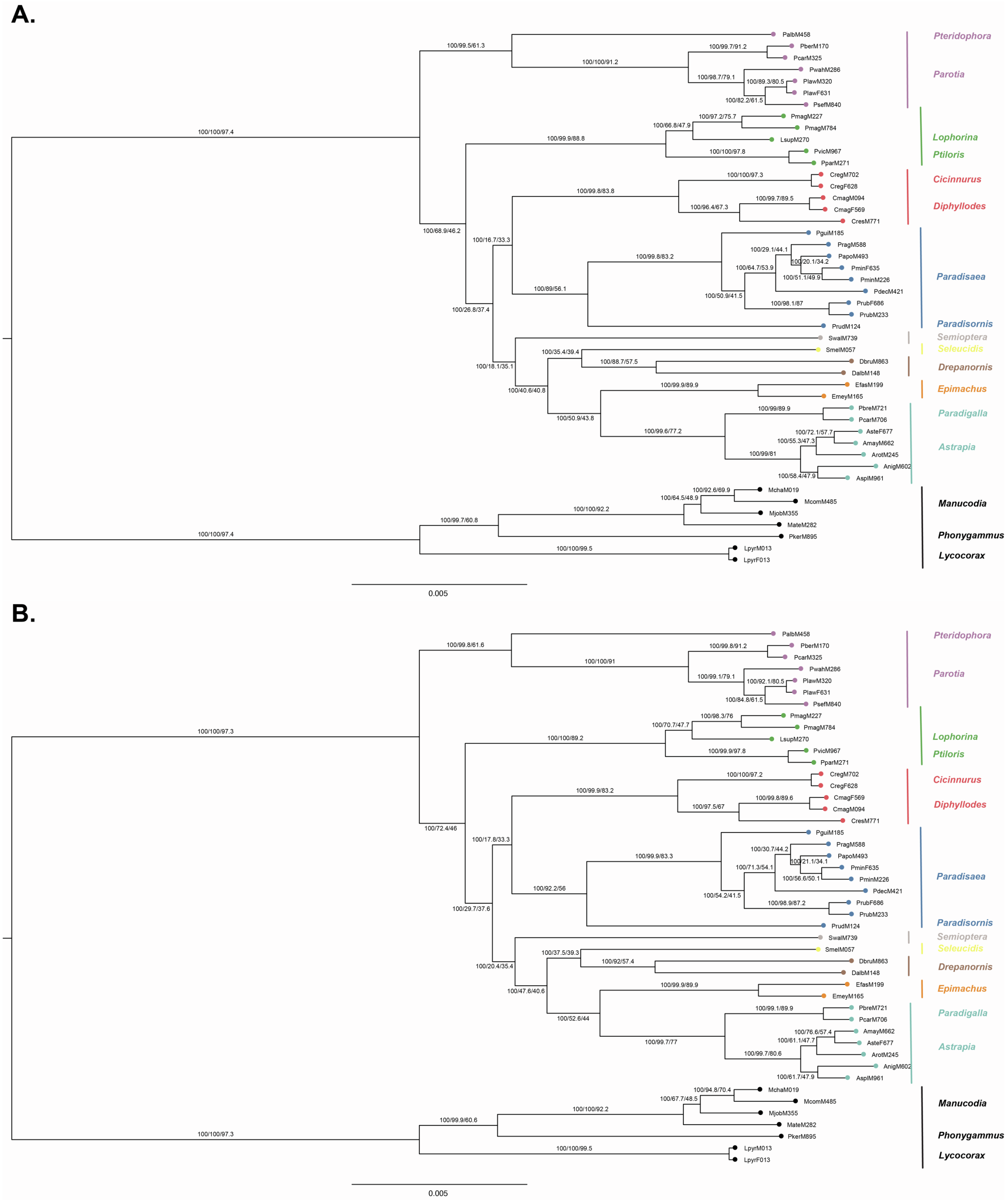

Figure S7: Maximum-Likelihood species trees, *excluding the 10 recent hybrids*, based on concatenation of filtered *autosomal* window alignments of length X (sampled every 100 kb). Related to Figure 1. A.) Window alignments of length 30 kb. B.) Window alignments of length 40 kb. All bipartitions are annotated with ultra-fast bootstrap support/window concordance factors/site concordance factors. All major genera are monophyletic and highly supported, but intergeneric relationships remain largely unresolved.

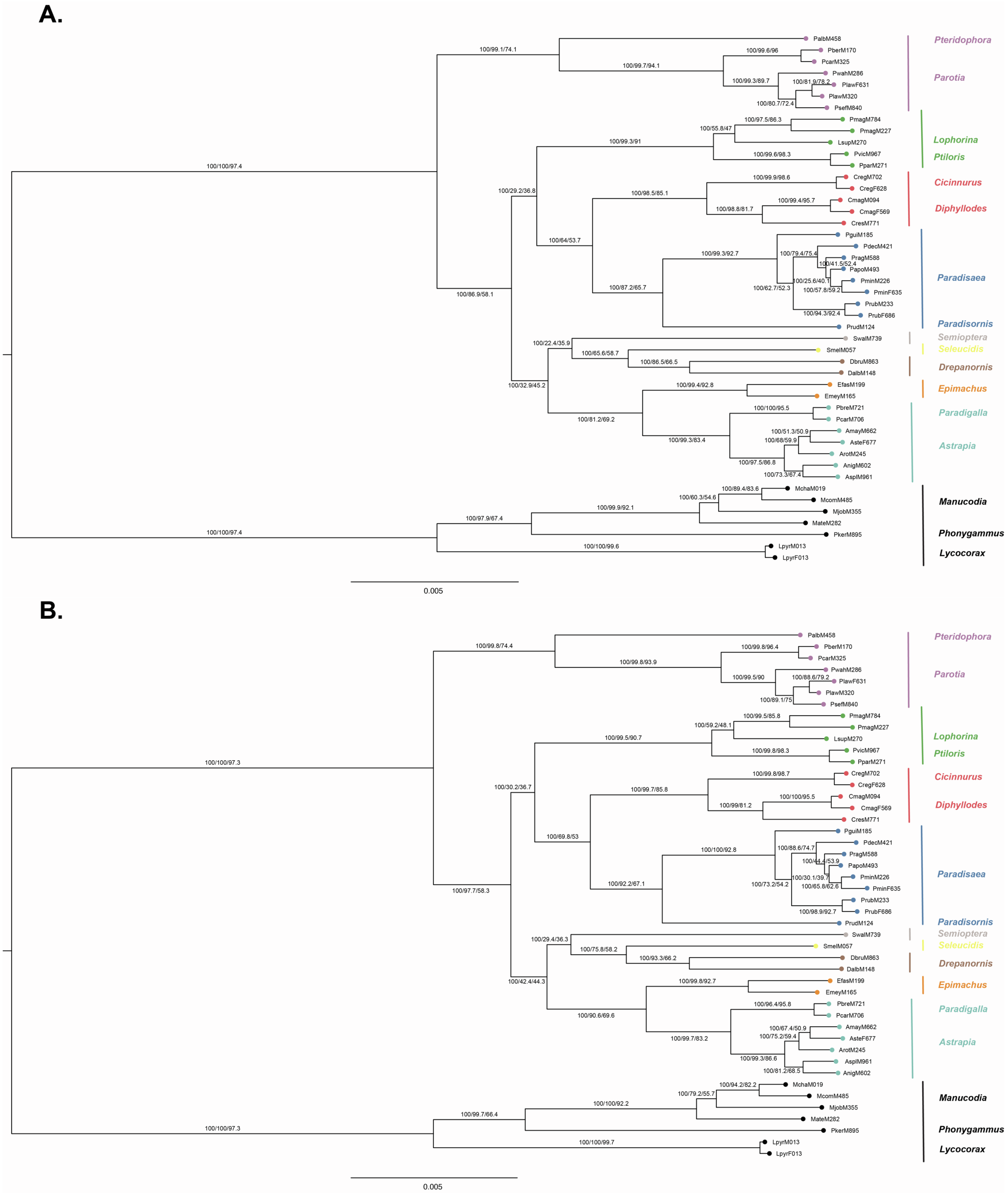

Figure S8. Maximum-Likelihood species trees, *excluding the 10 recent hybrids*, based on concatenation of filtered *Z chromosomal* window alignments of length X (sampled every 100 kb). Related to Figure 1. A.) Window alignments of length 10 kb. B.) Window alignments of length 20 kb. All bipartitions are annotated with ultra-fast bootstrap support/window concordance factors/site concordance factors. All genera are monophyletic and highly supported. In comparison to the species trees based on autosomal loci, the intergeneric relationships are more resolved and most notably, there is strong support for a sister-relationship between *Cicinnurus*/*Diphyllodes* and *Paradisaea/Paradisornis*.

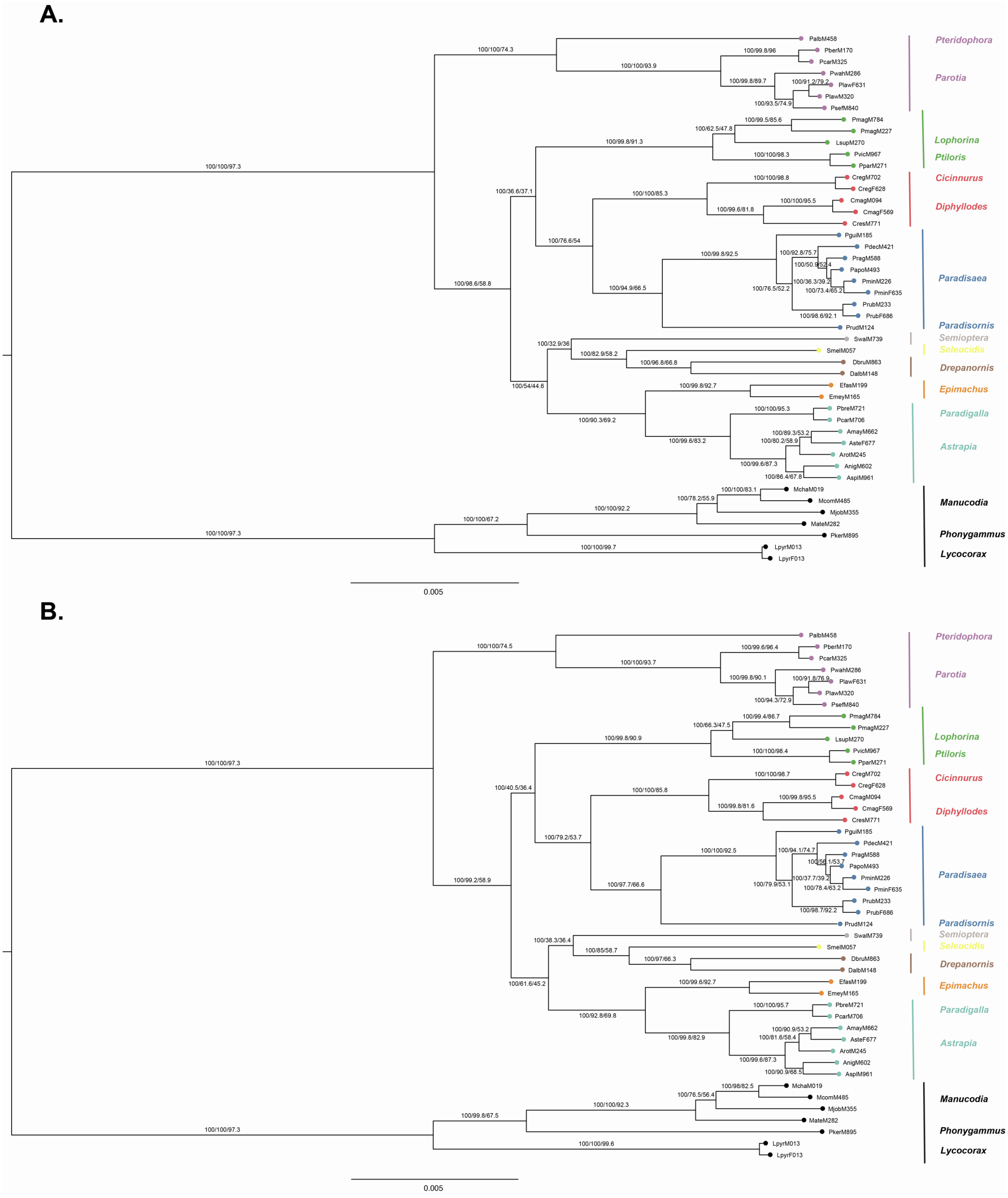

Figure S9. Maximum-Likelihood species trees, *excluding the 10 recent hybrids*, based on concatenation of filtered *Z chromosomal* window alignments of length X (sampled every 100 kb). Related to Figure 1. A.) Window alignments of length 30 kb. B.) Window alignments of length 40 kb. All bipartitions are annotated with ultra-fast bootstrap support/window concordance factors/site concordance factors. All genera are monophyletic and highly supported. In comparison to the species trees based on autosomal loci, the intergeneric relationships are more resolved and most notably, there is strong support for a sister-relationship between *Cicinnurus*/*Diphyllodes* and *Paradisaea/Paradisornis*.

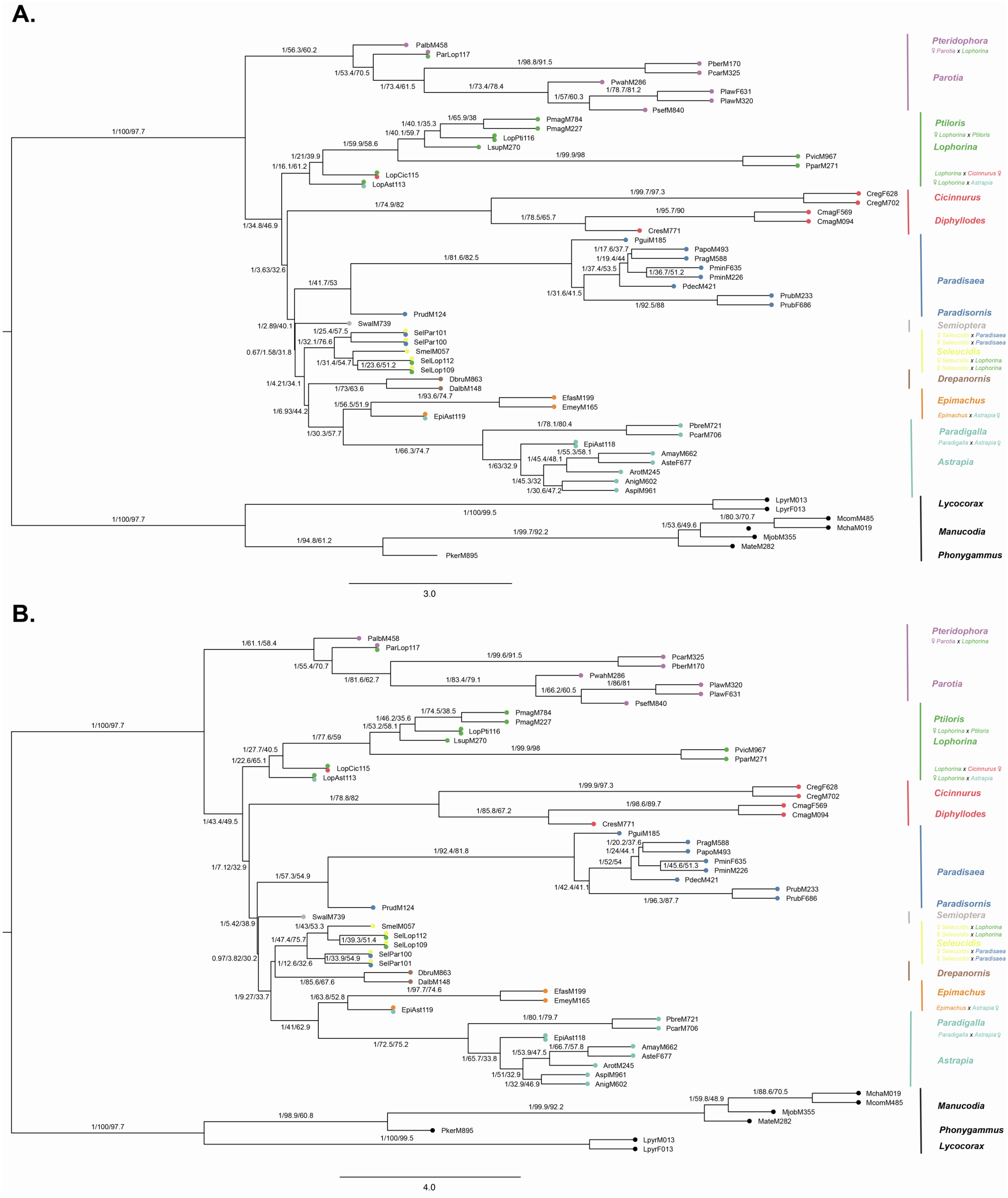

Figure S10. Summary-coalescent species trees for all individuals, *including* the 10 recent hybrids, based on filtered *autosomal* window alignments of length X (sampled every 100 kb). Related to Figure 1. A.) Window alignments of length 10 kb. B.) Window alignments of length 20 kb. All bipartitions are annotated with posterior support/window concordance factors/site concordance factors. The 10 recent hybrids have been highlighted with double orbs, with colour fills representing the predicted parental genera based on phenotype, and are often placed as a sister taxon to one of the predicted parental genera. Terminal branches are all scaled to 1 coalescent unit and are non-informative.

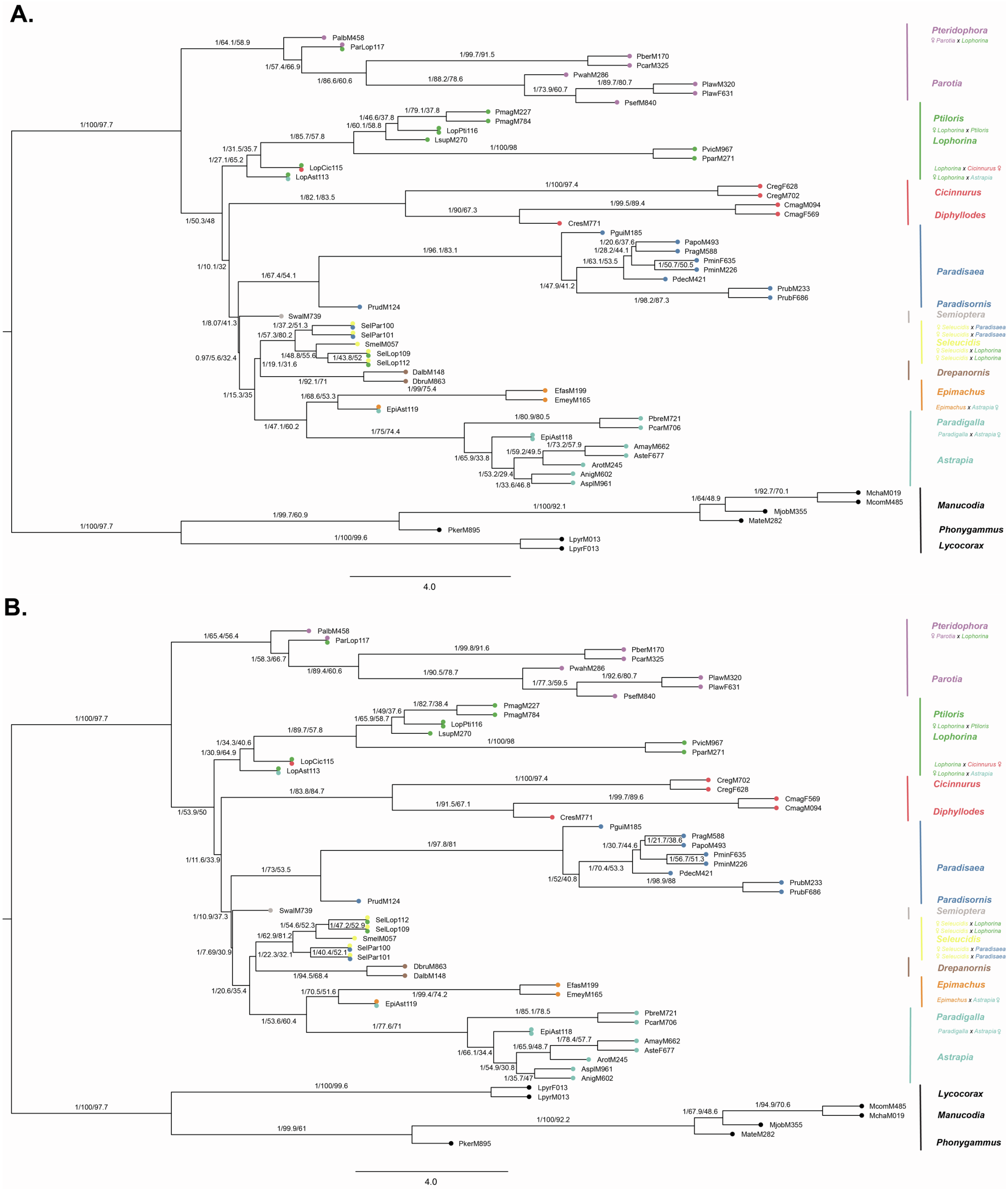

Figure S11. Summary-coalescent species trees for all individuals, *including* the 10 recent hybrids, based on filtered *autosomal* window alignments of length X (sampled every 100 kb). Related to Figure 1. A.) Window alignments of length 30 kb. B.) Window alignments of length 40 kb. All bipartitions are annotated with posterior support/window concordance factors/site concordance factors. The 10 recent hybrids have been highlighted with double orbs, with colour fills representing the predicted parental genera based on phenotype, and are often placed as a sister taxon to one of the predicted parental genera. Terminal branches are all scaled to 1 coalescent unit and are non-informative.

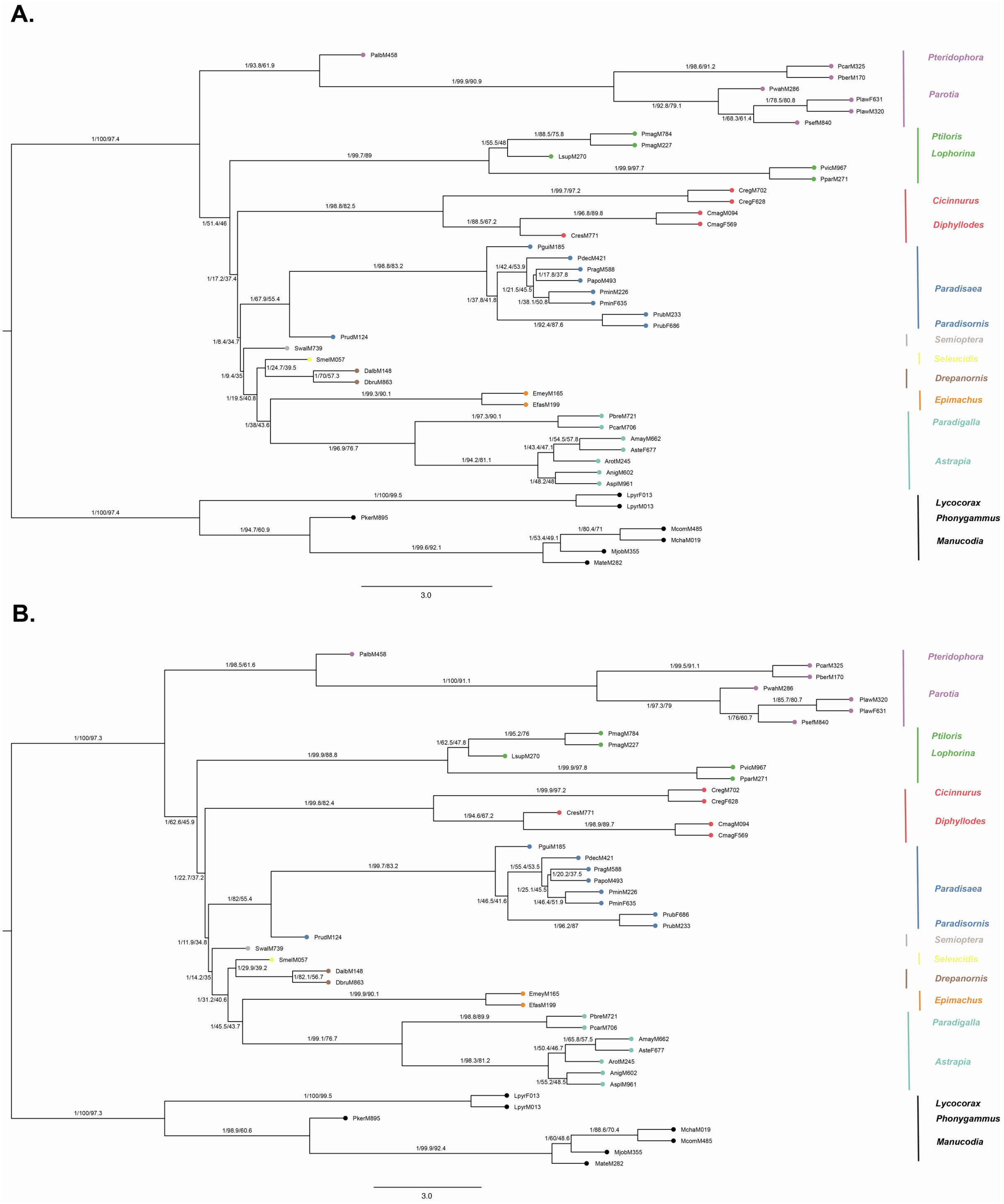

Figure S12. Summary-Coalescent species trees, *excluding* the 10 recent hybrids, based on filtered *autosomal* window alignments of length X (sampled every 100 kb). Related to Figure 1. A.) Window alignments of length 10 kb. B.) Window alignments of length 20 kb. All bipartitions are annotated with posterior support/window concordance factors/site concordance factors. Terminal branches are all scaled to 1 coalescent unit and are non-informative. All major genera are monophyletic and highly supported, but intergeneric relationships remain largely unresolved.

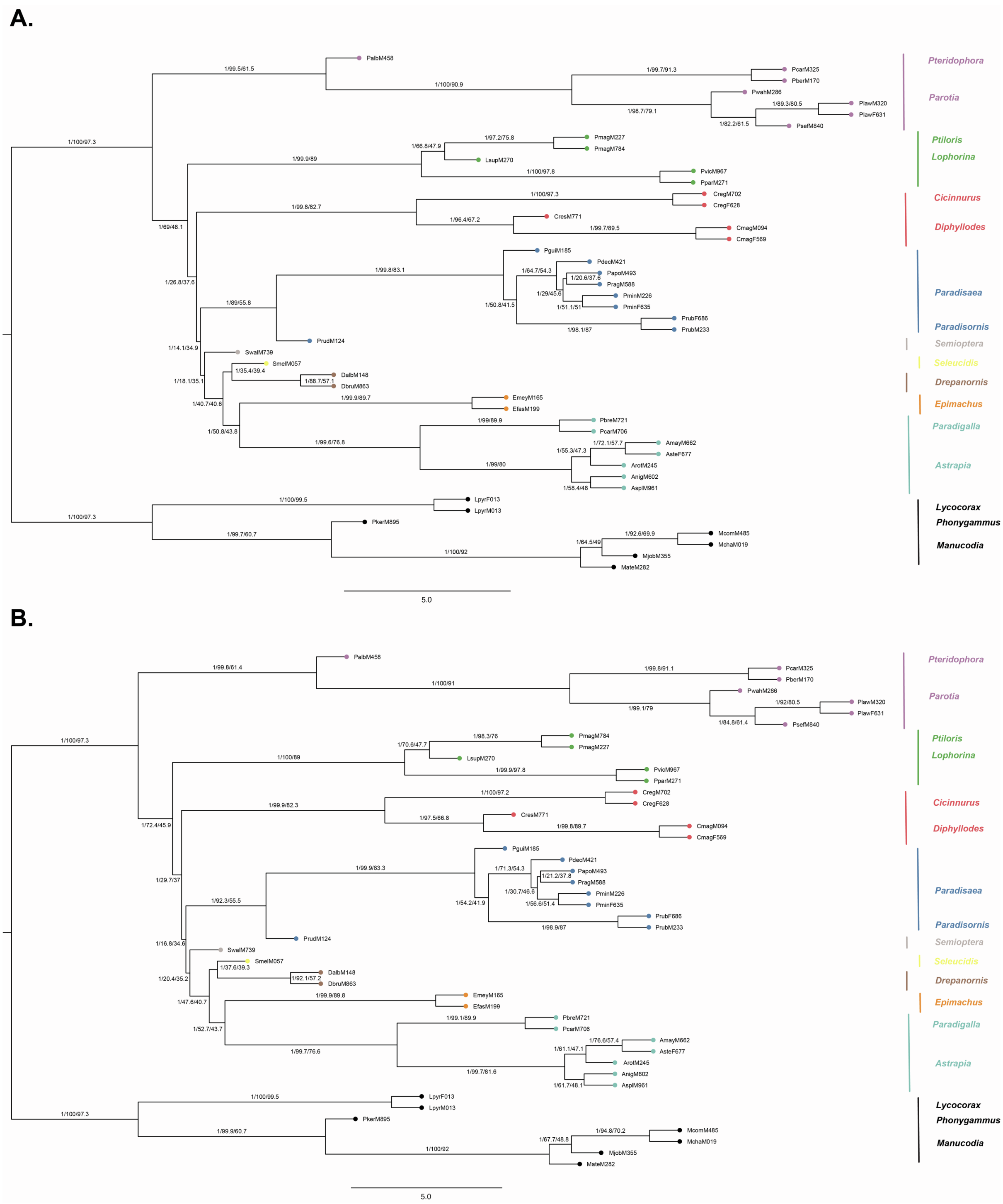

Figure S13. Summary-Coalescent species trees, *excluding* the 10 recent hybrids, based on filtered *autosomal* window alignments of length X (sampled every 100 kb). Related to Figure 1. A.) Window alignments of length 30 kb. B.) Window alignments of length 40 kb. All bipartitions are annotated with posterior support/window concordance factors/site concordance factors. Terminal branches are all scaled to 1 coalescent unit and are non-informative. All major genera are monophyletic and highly supported, but intergeneric relationships remain largely unresolved.

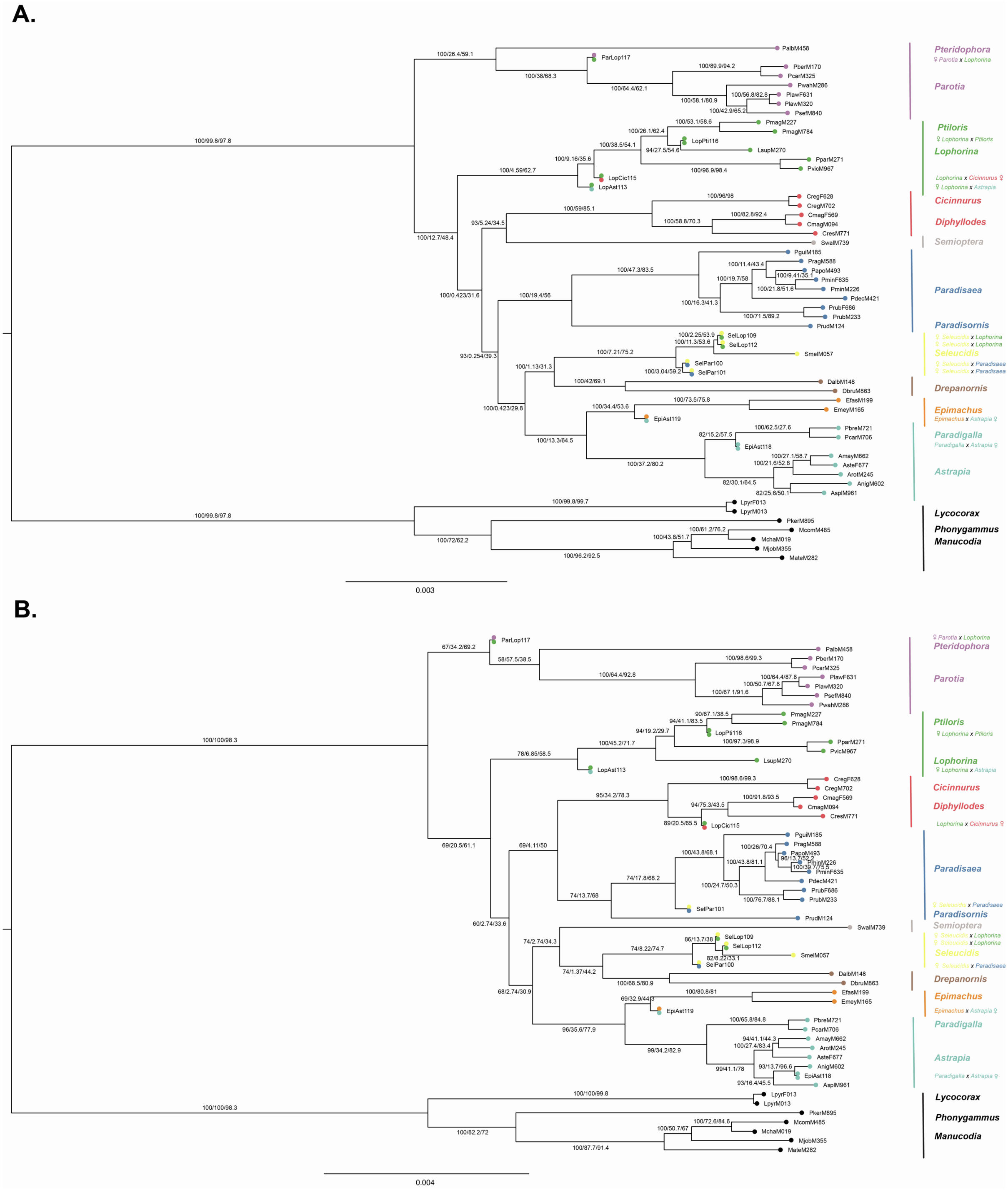

Figure S14. Maximum-likelihood species trees for all individuals, *including* the 10 recent hybrids, based on *Ultra-Conserved Element* loci. Related to Figure 1. A.) UCE loci located on autosomes. B.) UCE loci located on Z-chromosome. All bipartitions are annotated with ultra-fast bootstrap support or posterior support/window concordance factors/site concordance factors. The 10 recent hybrids have been highlighted with double orbs, with colour fills representing the predicted parental genera based on phenotype. Species trees are largely concordant with the window-tree based analyses suggesting that the possible inclusion of some non-neutrally evolving regions have no demonstrable effect on the qualitative interpretation of evolutionary history.

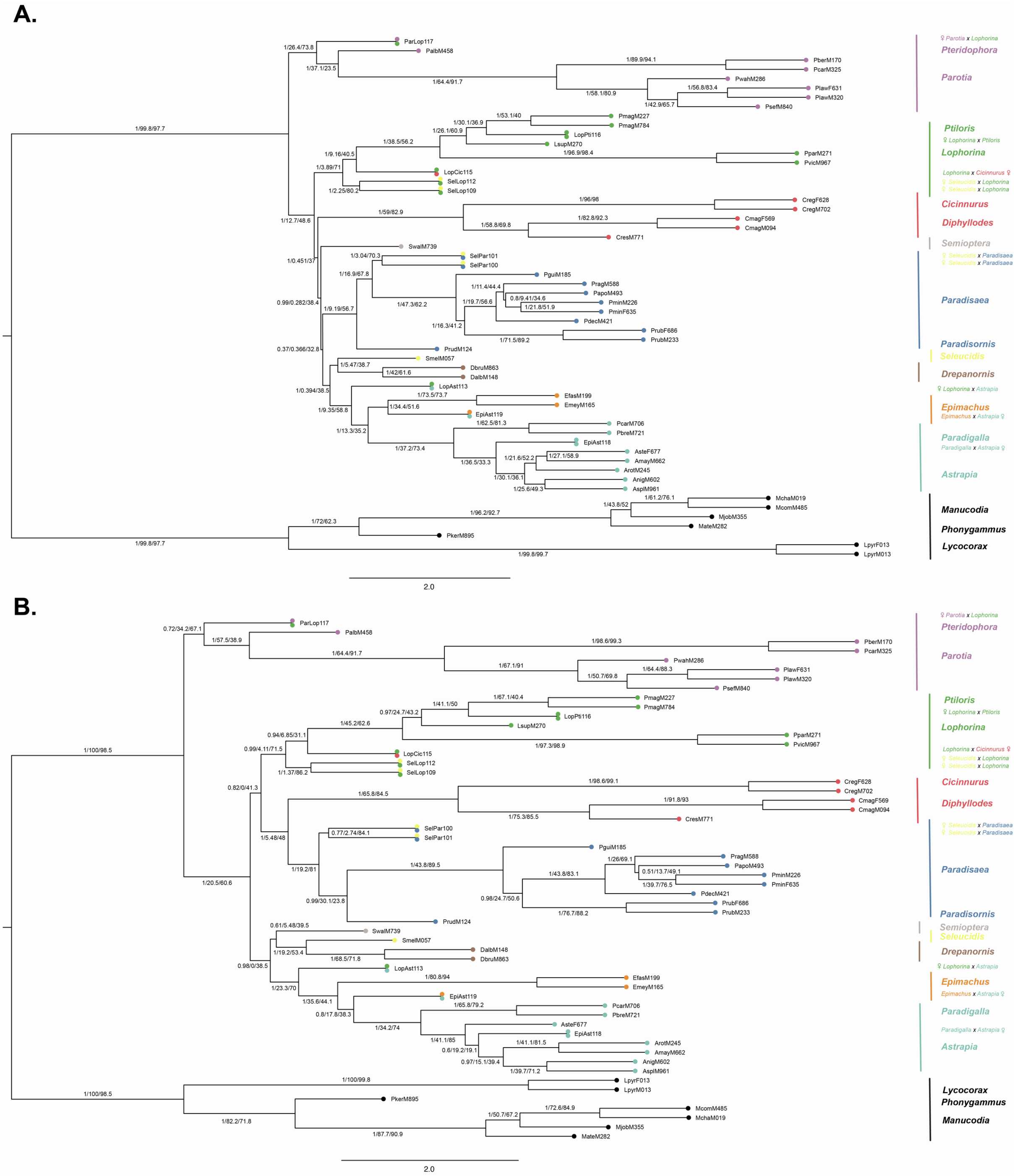

Figure S15. Summary-coalescent species trees for all individuals, *including* the 10 recent hybrids, based on *Ultra-Conserved Element* loci. Related to Figure 1. A.) UCE loci located on autosomes. B.) UCE loci located on Z-chromosome. All bipartitions are annotated with ultra-fast bootstrap support or posterior support/window concordance factors/site concordance factors. The 10 recent hybrids have been highlighted with double orbs, with colour fills representing the predicted parental genera based on phenotype. Species trees are largely concordant with the window-tree based analyses suggesting that the possible inclusion of some non-neutrally evolving regions have no demonstrable effect on the qualitative interpretation of evolutionary history.

A.

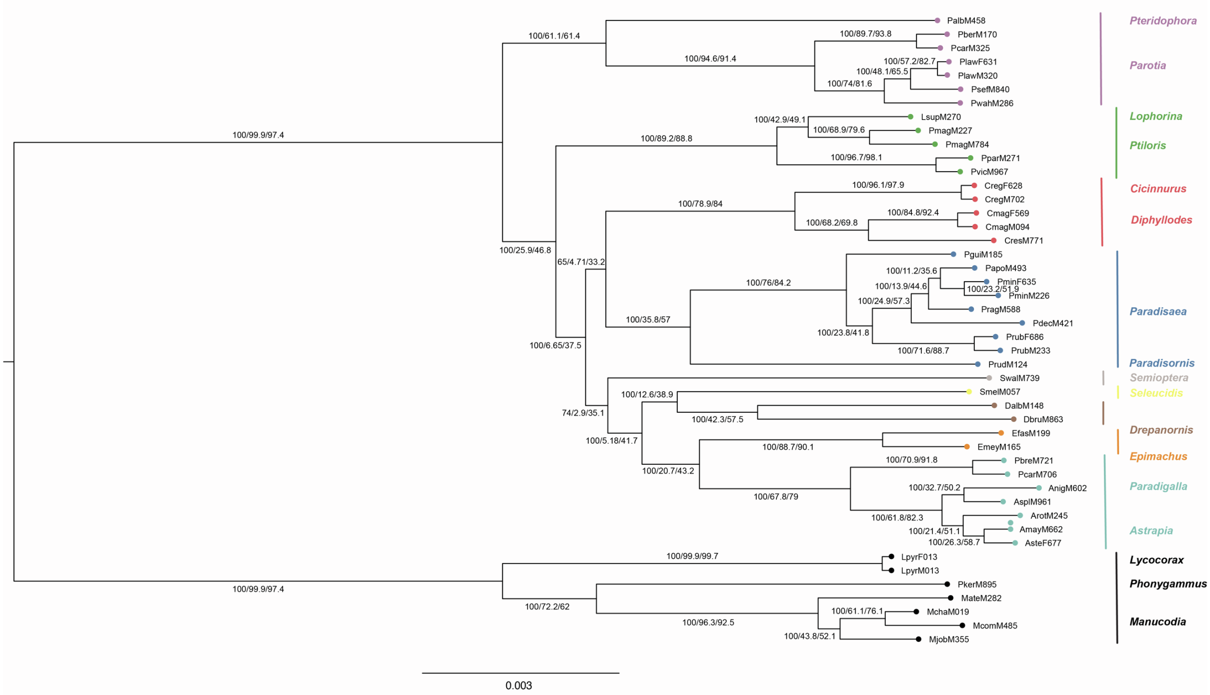

B.

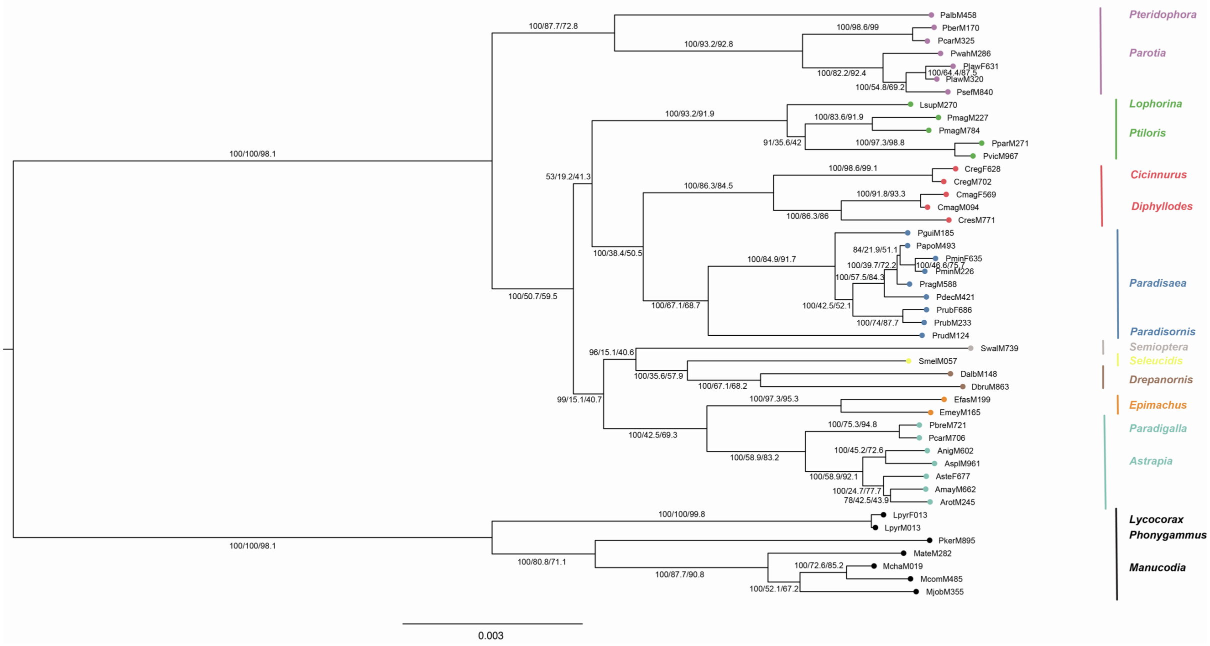

Figure S16. Maximum-likelihood species trees for all individuals, *excluding* the 10 recent hybrids, based on *Ultra-Conserved Element* loci. Related to Figure 1. A.) UCE loci located on autosomes. B.) UCE loci located on Z-chromosome. All bipartitions are annotated with ultra-fast bootstrap support or posterior support/window concordance factors/site concordance factors. The 10 recent hybrids have been highlighted with double orbs, with colour fills representing the predicted parental genera based on phenotype. Species trees are largely concordant with the window-tree based analyses suggesting that the possible inclusion of some non-neutrally evolving regions have no demonstrable effect on the qualitative interpretation of evolutionary history.

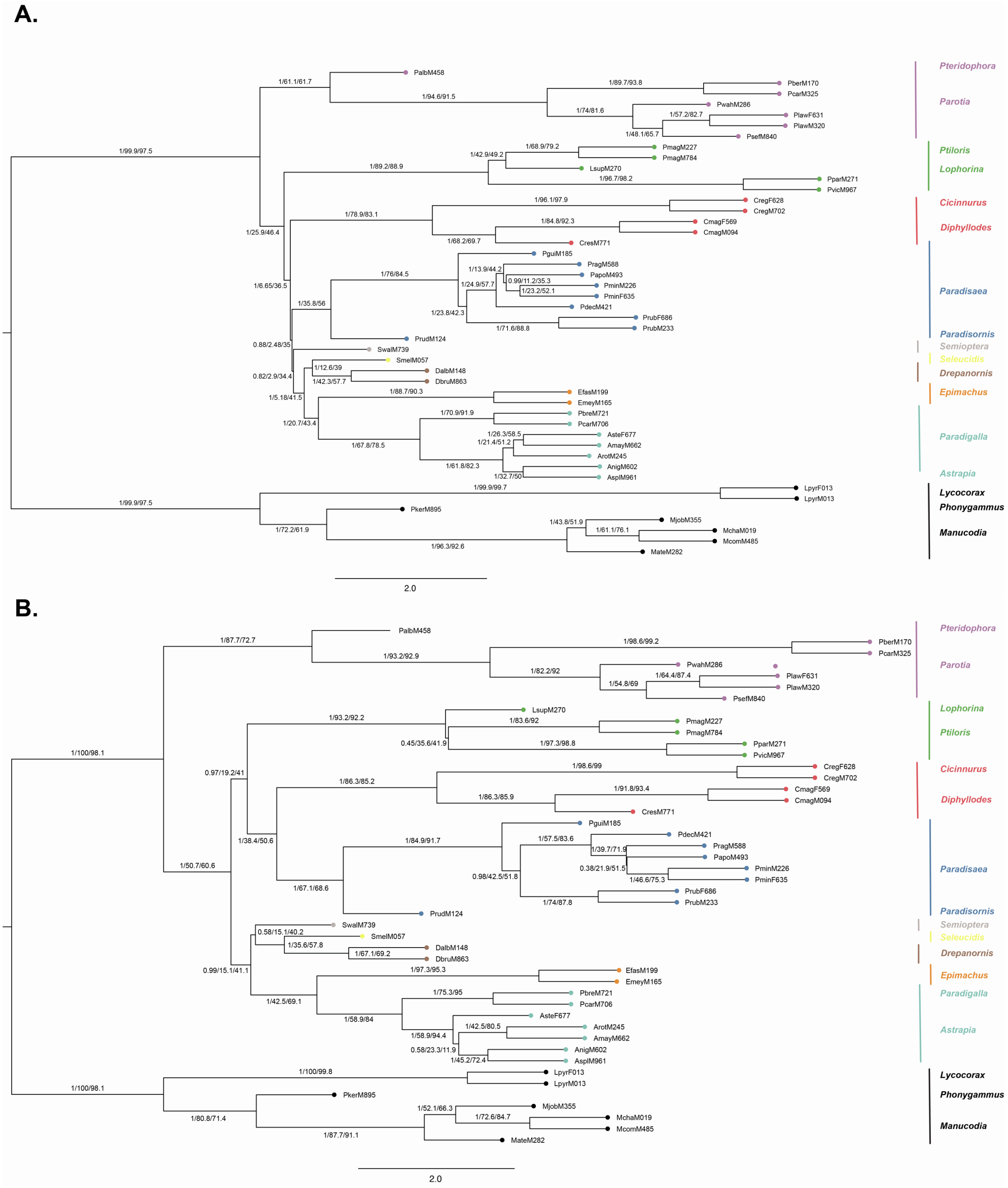

Figure S17. Summary-coalescent species trees for all individuals, *excluding* the 10 recent hybrids, based on *Ultra-Conserved Element* loci. Related to Figure 1. A.) UCE loci located on autosomes. B.) UCE loci located on Z-chromosome. All bipartitions are annotated with ultra-fast bootstrap support or posterior support/window concordance factors/site concordance factors. The 10 recent hybrids have been highlighted with double orbs, with colour fills representing the predicted parental genera based on phenotype. Species trees are largely concordant with the window-tree based analyses suggesting that the possible inclusion of some non-neutrally evolving regions have no demonstrable effect on the qualitative interpretation of evolutionary history.

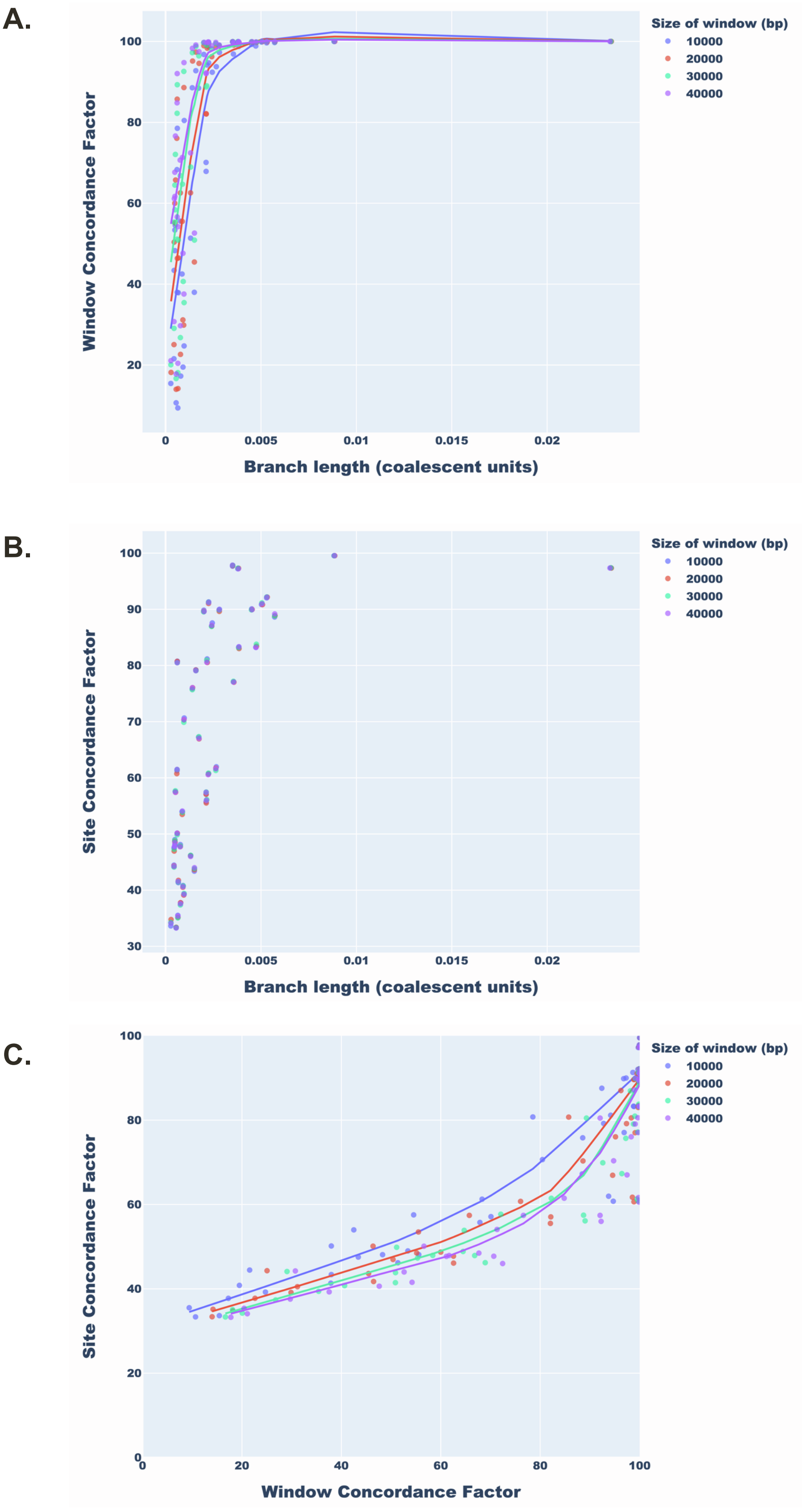

Figure S18. Correlation between window-, site-concordance factors (CF) and branch lengths of the summary-coalescent species trees (excluding recent hybrids). Related to Figure 1. A.) Longer branches correlate with higher window CF's. B.) Longer branches correlate with higher site CF's, but is more noisy relative to window CF's. C.) Correlation between sCF and wCF at different window alignment lengths.

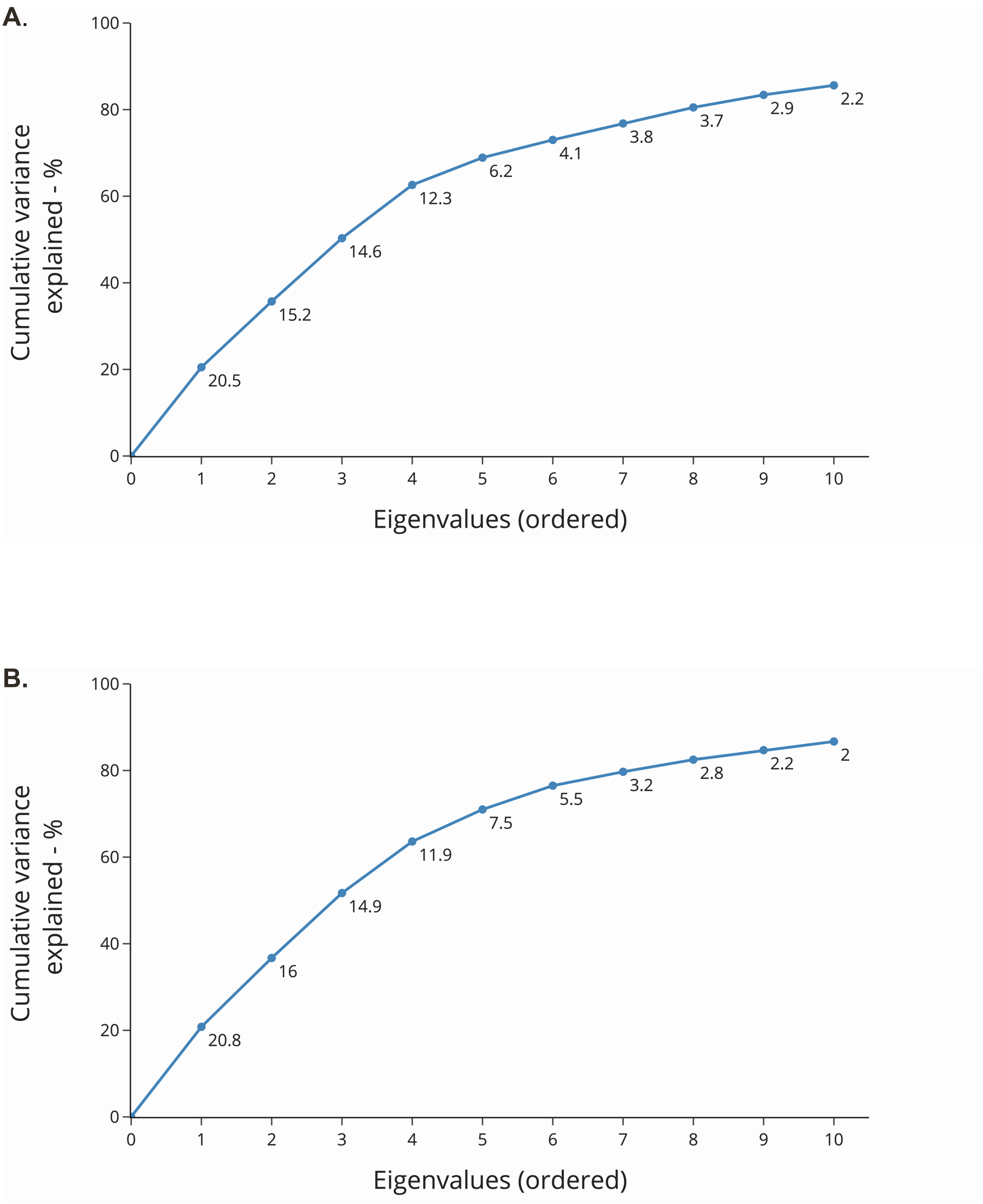

Figure S19. The (cumulative) amount of variation explained by each Principal Component axis, for the Principal Component Analyses based on *Genotype Likelihoods*. Related to Figure 1. A.) Excluding the 10 recent hybrids. B.) Including the 10 recent hybrids. Amount of variation explained by each eigenvalue listed by each data point.

A.

B.

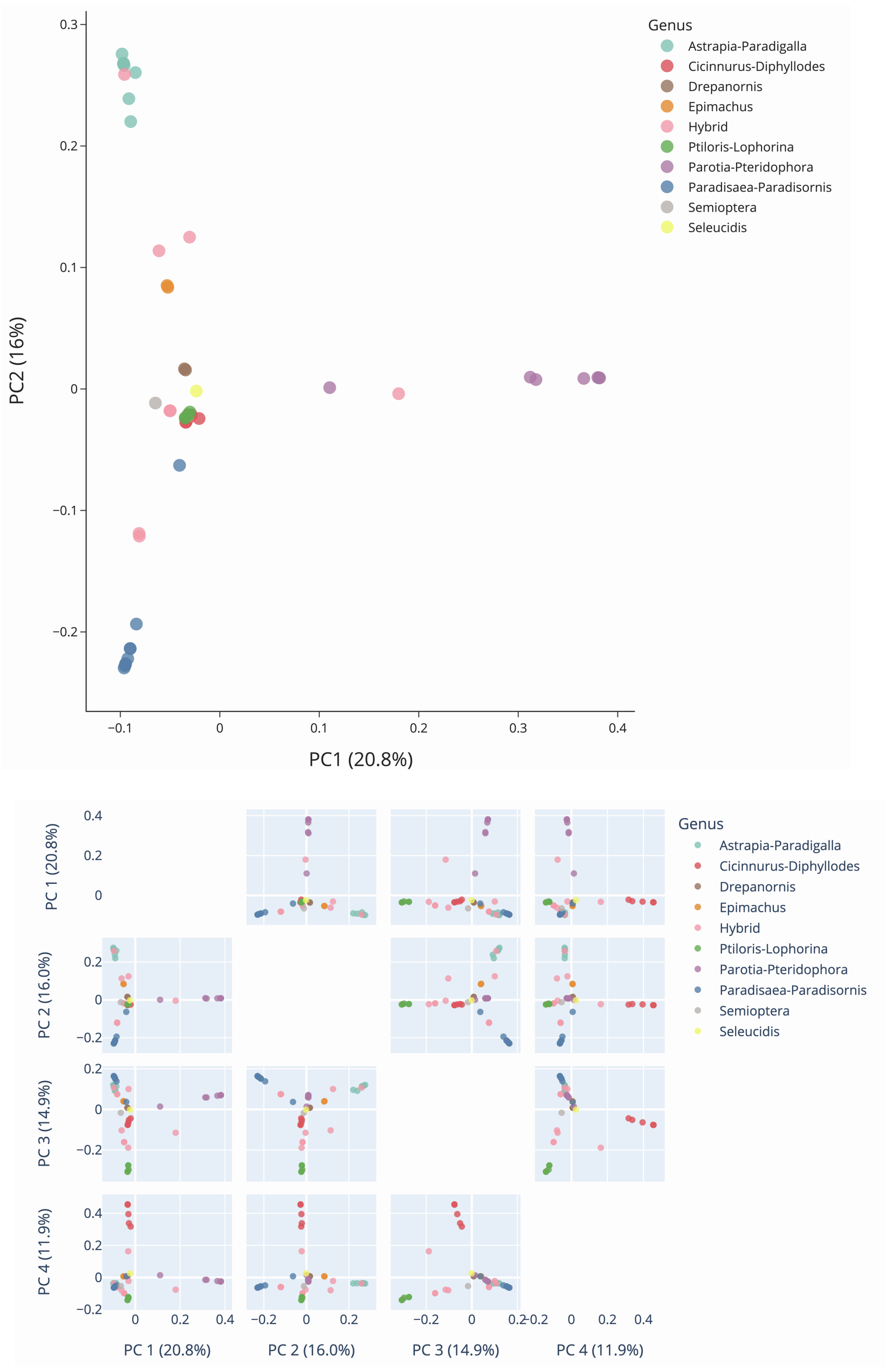

Figure S20. Principal Component Analyses (PCA) based on genotype likelihoods and including the 10 recent hybrids. Related to Figure 1. A.) PCA plot for first two major axes of variation. B.) Pairwise plot for first four major axes of variation. Individuals belonging to the same genus cluster relatively closely and hybrids are placed intermediately between putative parental genera.

A.

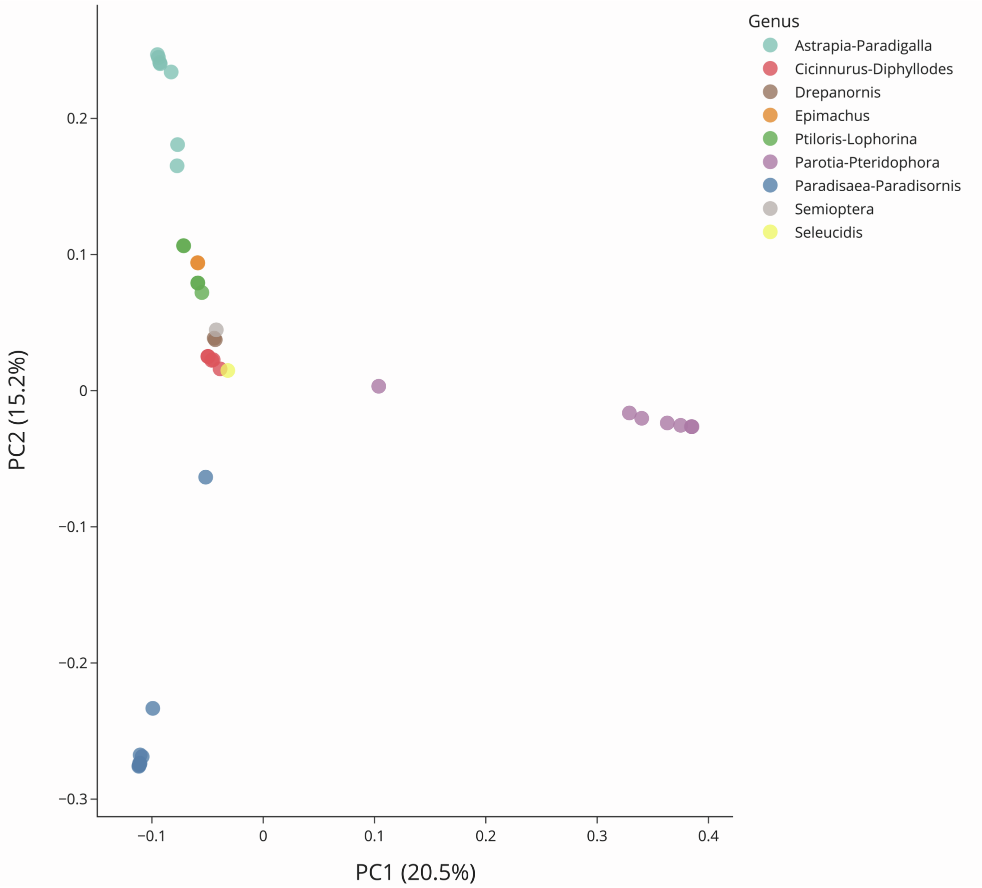

B.

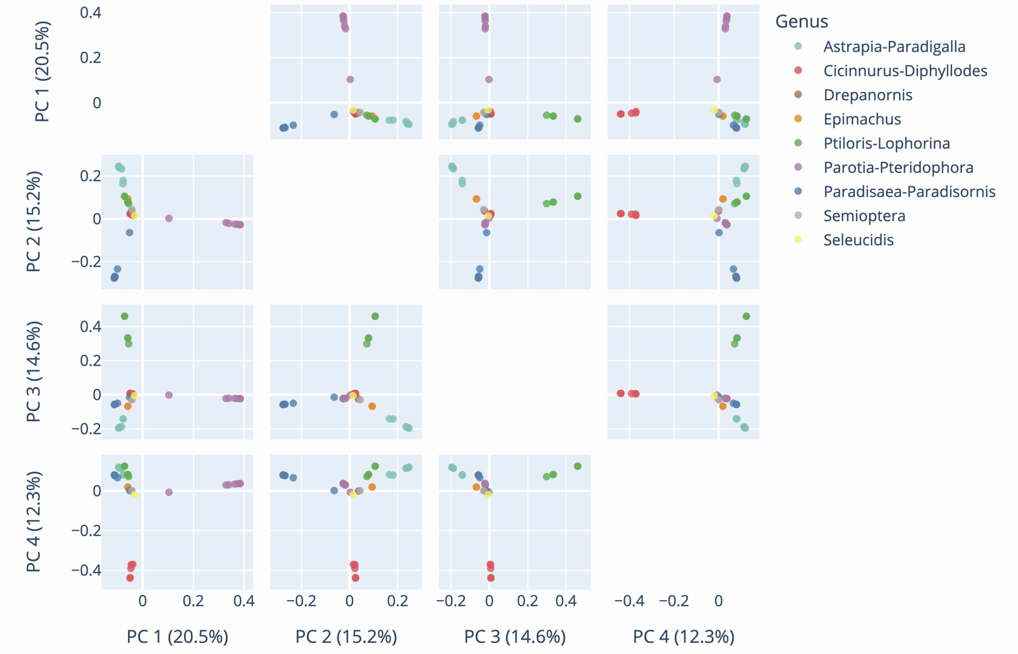

Figure S21. Principal Component Analyses (PCA) based on genotype likelihoods and excluding the 10 recent hybrids. Related to Figure 1. A.) PCA plot for first two major axes of variation. B.) Pairwise plot for first four major axes of variation. Individuals belonging to the same genus cluster relatively closely.

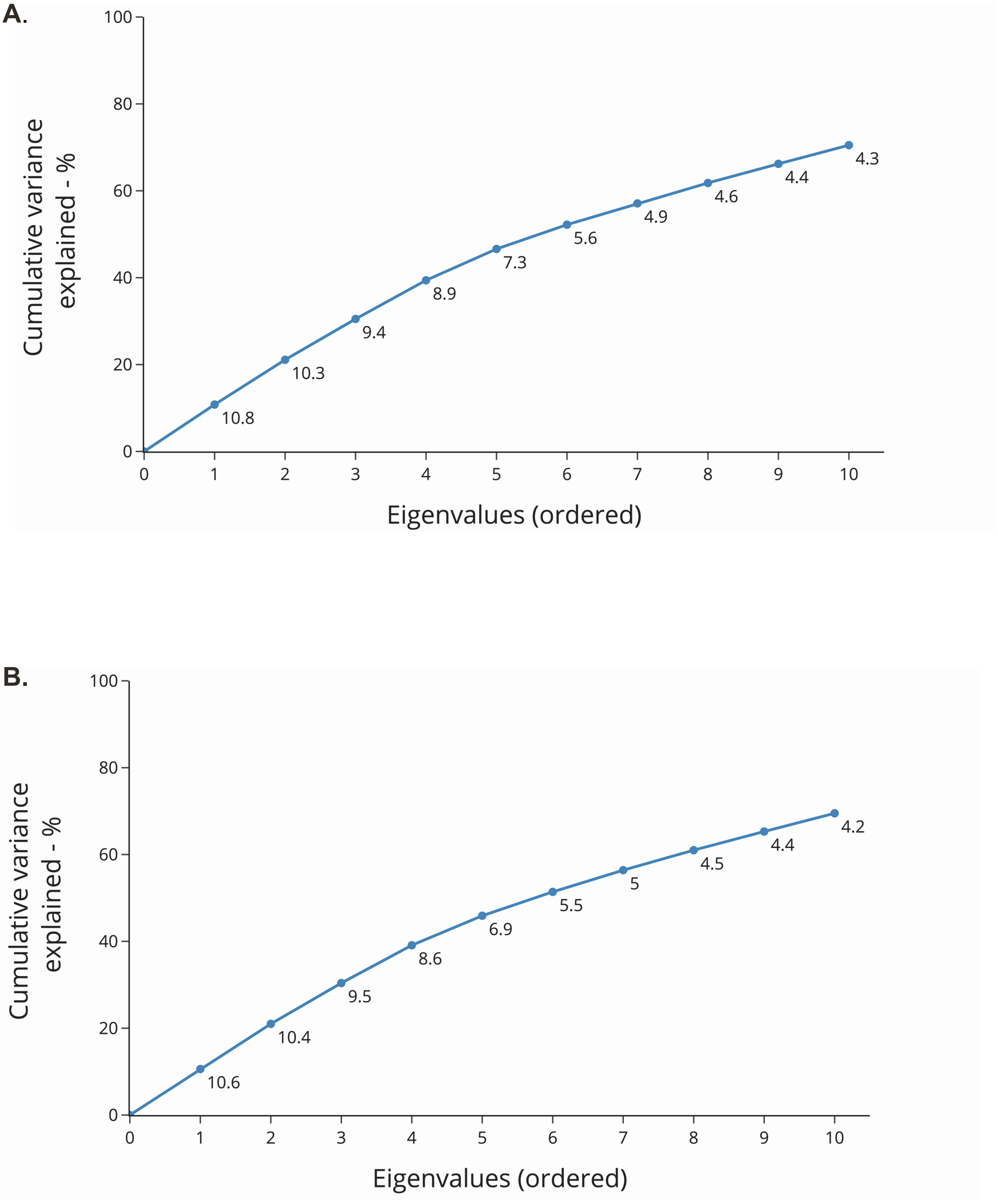

Figure S22. The (cumulative) amount of variation explained by each Principal Component axis, for the Principal Component Analyses based on *hard-called genotypes*. Related to Figure 1. A.) Excluding the 10 recent hybrids. B.) Including the 10 recent hybrids. Amount of variation explained by each eigenvalue listed by each data point.

A.

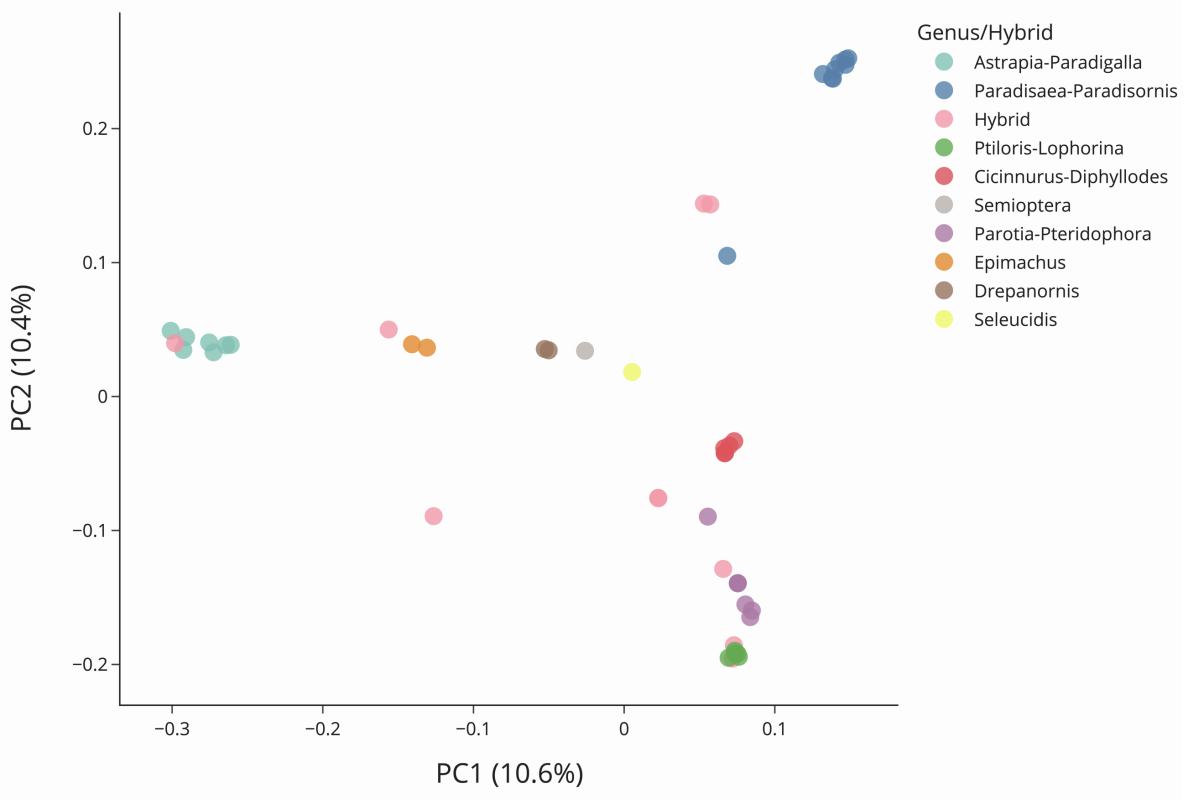

B.

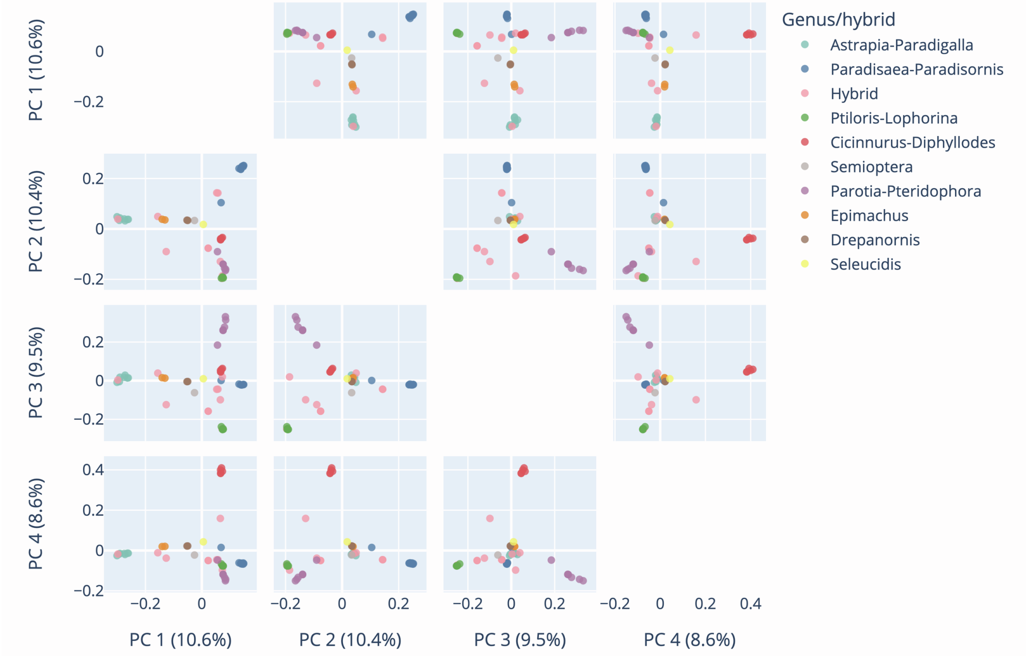

Figure S23. Principal Component Analyses (PCA) based on hard-called genotypes and including the 10 recent hybrids. Related to Figure 1. A.) PCA plot for first two major axes of variation. B.) Pairwise plot for first four major axes of variation. Individuals belonging to the same genus cluster relatively closely and hybrids are placed intermediately between putative parental genera.

A.

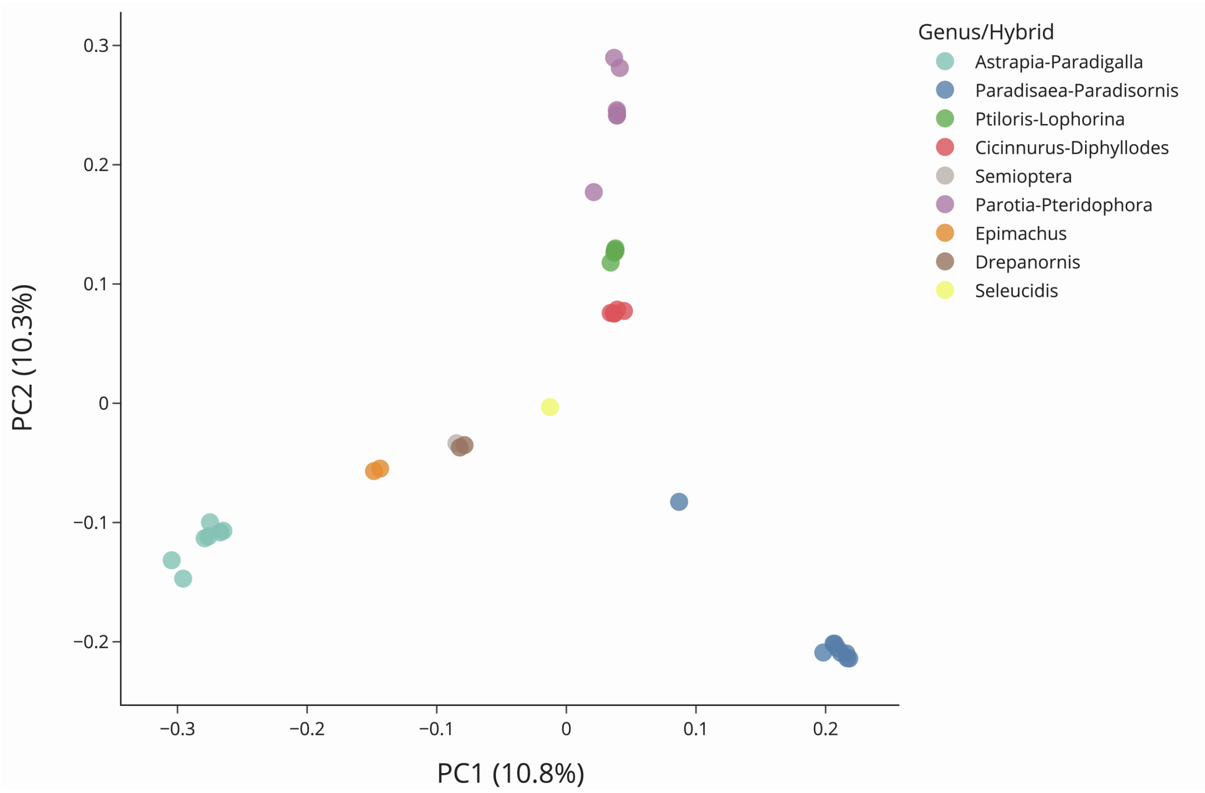

B.

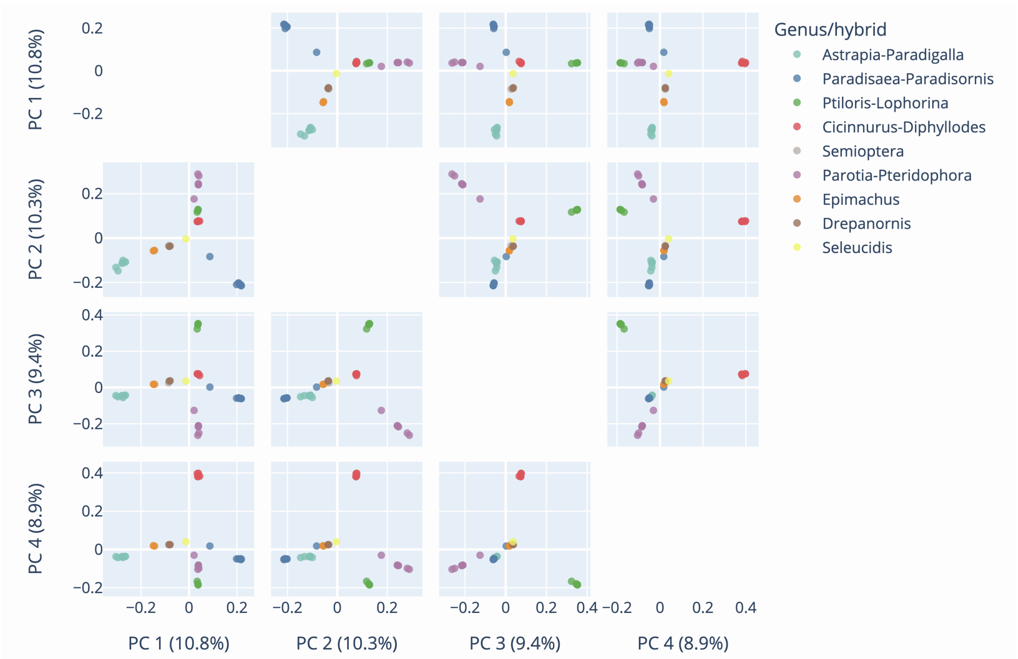

Figure S24. Principal Component Analyses (PCA) based on hard-called genotypes and excluding the 10 recent hybrids. Related to Figure 1. A.) PCA plot for first two major axes of variation. B.) Pairwise plot for first four major axes of variation. Individuals belonging to the same genus cluster relatively closely.

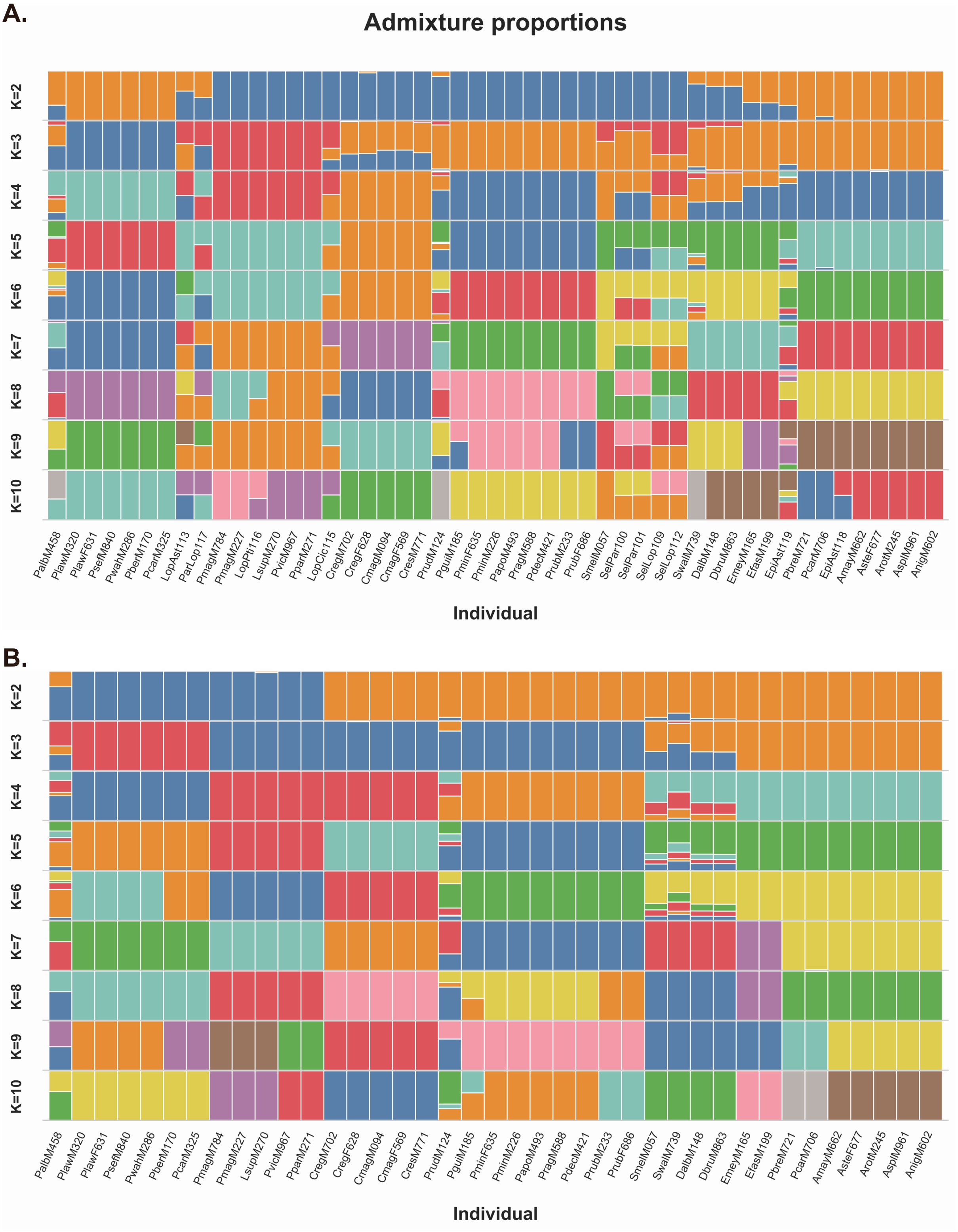

Figure S25. Admixture analyses based on genotype likelihoods. Related to Figure 1. A.) Including the 10 recent hybrids. B.) Excluding the 10 recent hybrids. Evanno's K prediction for the optimal K, as implemented in Clumpak, suggests K=6 for the analysis including hybrids and K=5 for the analysis excluding hybrids, but higher values of K generally recover genera affiliation well. Colour scheme at each K randomly assigned and does not correspond to the genus assignment used elsewhere. Individuals tend to cluster by genus at the higher values for K and hybrids exhibit a highly admixed origin.

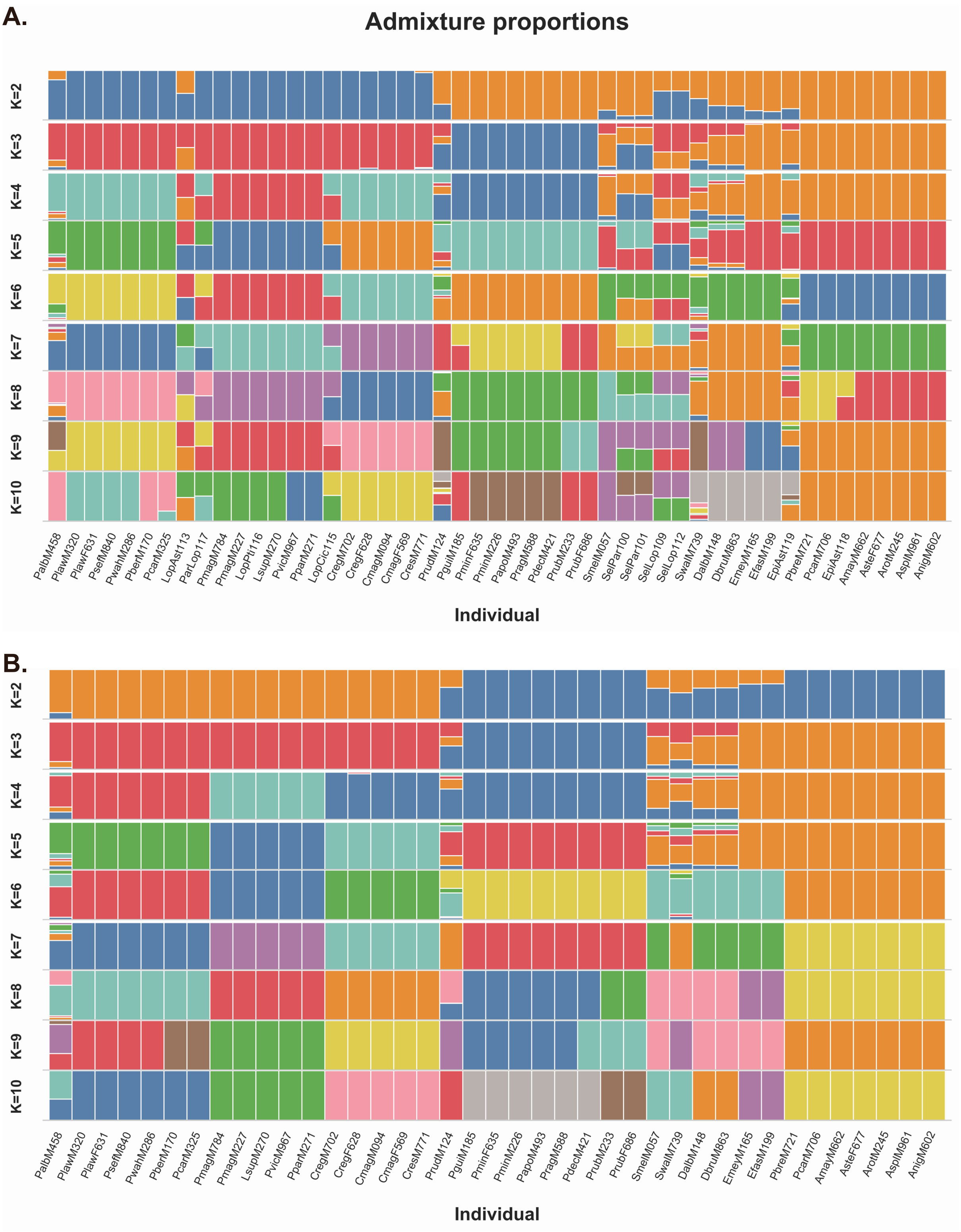

Figure S26. Admixture analyses based on hard-called genotypes. Related to Figure 1. A.) Including the 10 recent hybrids. B.) Excluding the 10 recent hybrids. Cross validation error estimation suggest K=5 as the most optimal value for K, in both analyses, but higher values of K generally recover genera affiliation well. Colour scheme at each K randomly assigned and does not correspond to the genus assignment used elsewhere. Individuals tend to cluster by genus at the higher values for K and hybrids exhibit a highly admixed origin.

A.

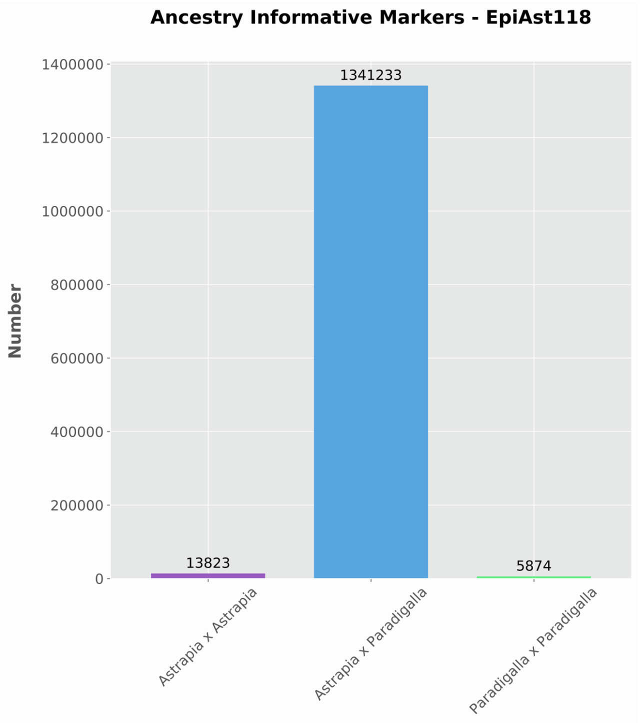

B.

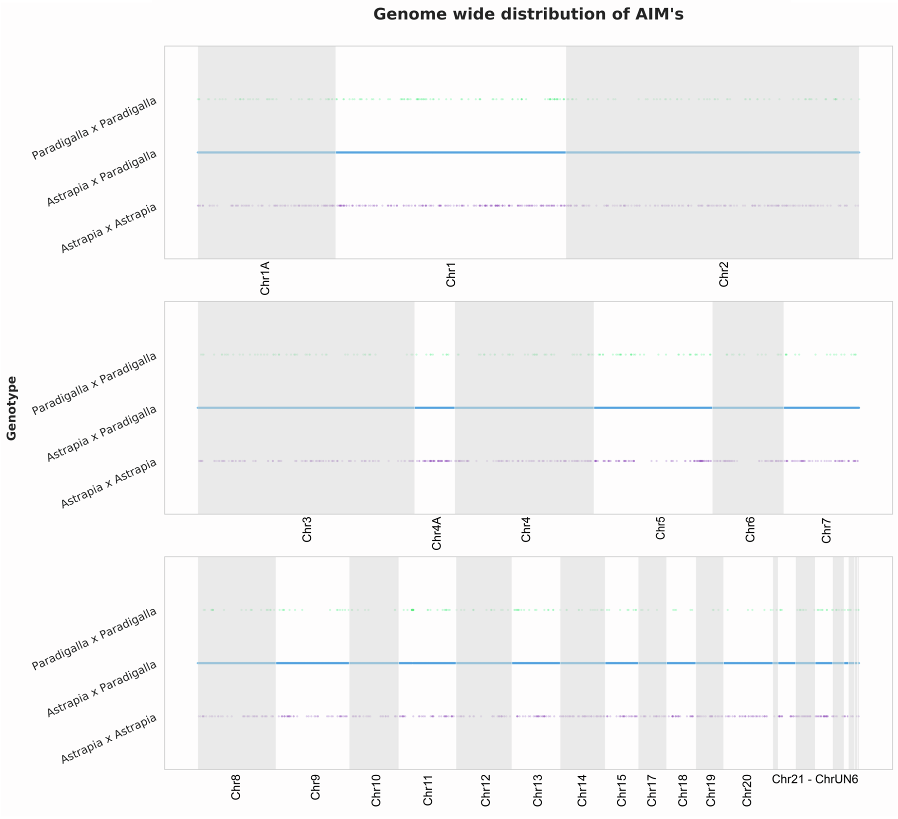

Figure S27. Genotype frequencies and genome-wide distribution of genotypes at Ancestry Informative Marker sites (AIMs) for hybrid individual EpiAst118. Related to Figure 1. A.) At AIMs, this individual is almost unequivocally heterozygous suggesting that it is an F1 hybrid. B.) The distribution of homozygous AIMs is relatively random across the genome; suggesting that these are most likely sites that were not fully fixed between parental genera, i.e. not true AIMs, and represent background noise.

A.

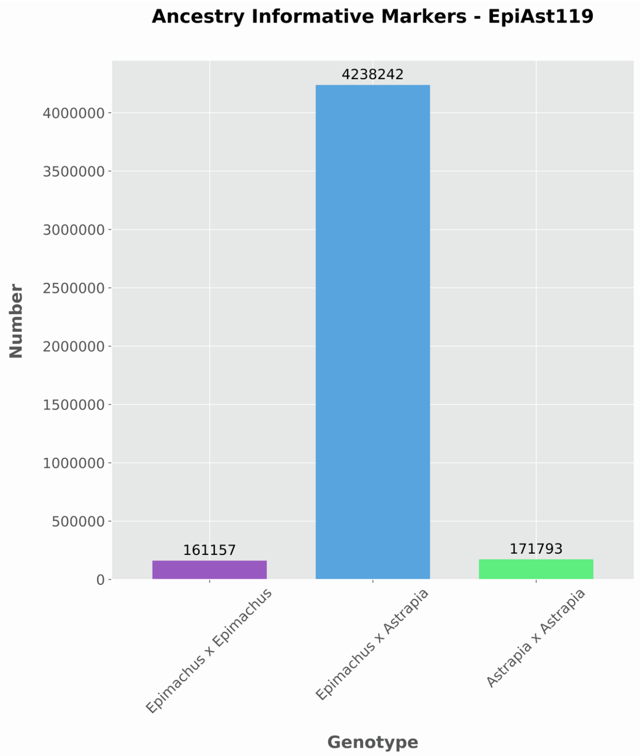

B.

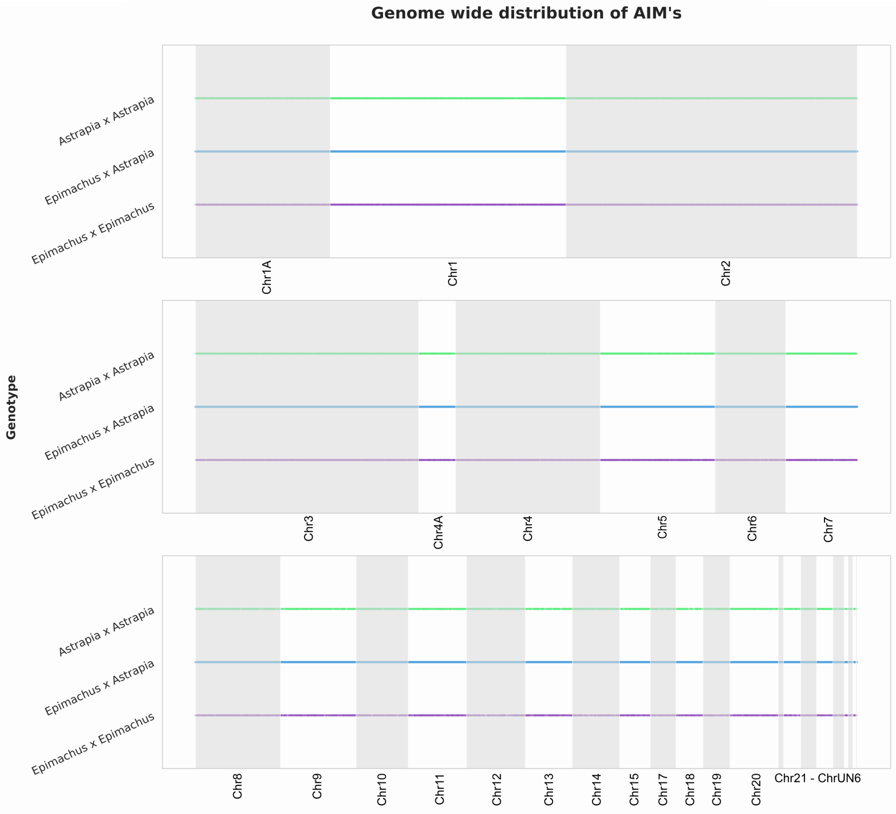

Figure S28. Genotype frequencies and genome-wide distributions of genotypes at Ancestry Informative Marker sites (AIMs) for hybrid individual EpiAst119. Related to Figure 1. A.) At AIMs, this individual is almost unequivocally heterozygous suggesting that it is an F1 hybrid. B.) The distribution of homozygous AIMs is relatively random across the genome; suggesting that these are most likely sites that were not fully fixed between parental genera, i.e. not true AIMs, and represent background noise.

A.

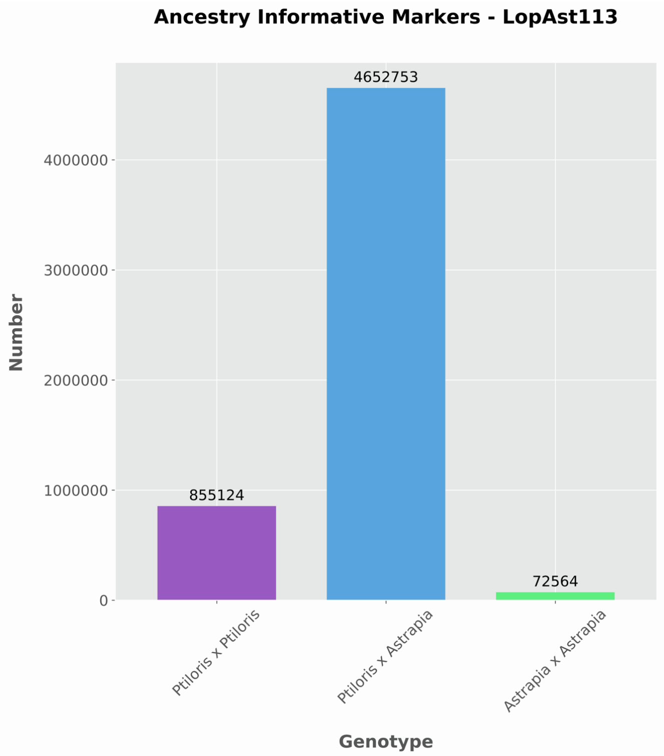

B.

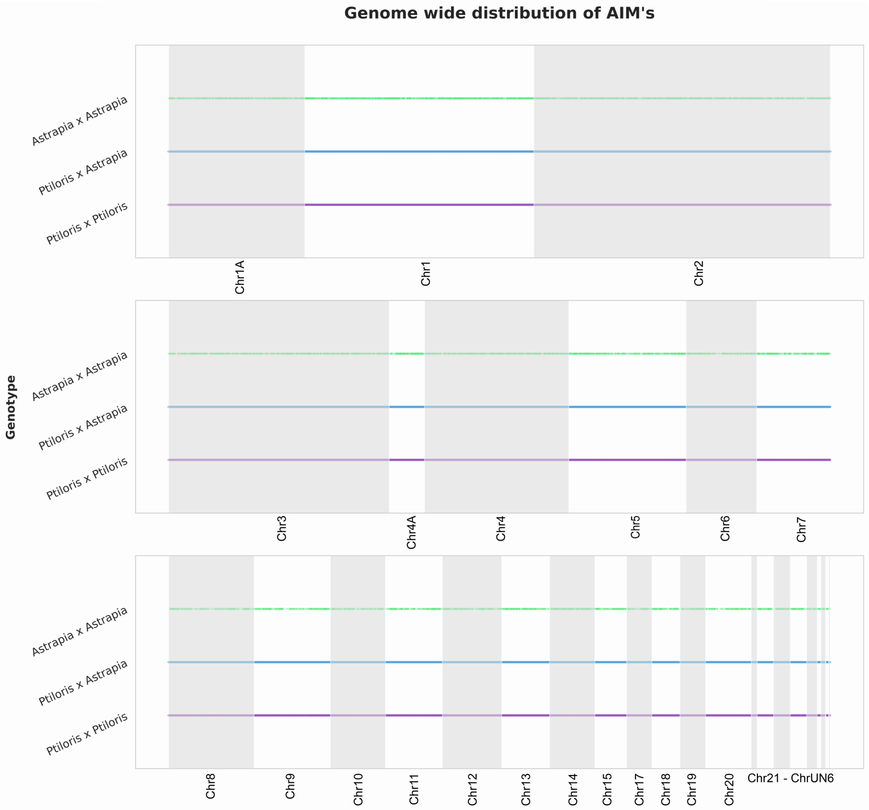

Figure S29. Genotype frequencies and genome-wide distributions of genotypes at Ancestry Informative Marker sites (AIMs) for hybrid individual LopAst113. Related to Figure 1. A.) At AIMs, this individual is highly heterozygous suggesting that it is most likely an F1 hybrid. B.) The distribution of homozygous AIMs is relatively random across the genome; suggesting that these are most likely sites that were not fully fixed between parental genera, i.e. not true AIMs, and represent background noise.

A.

B.

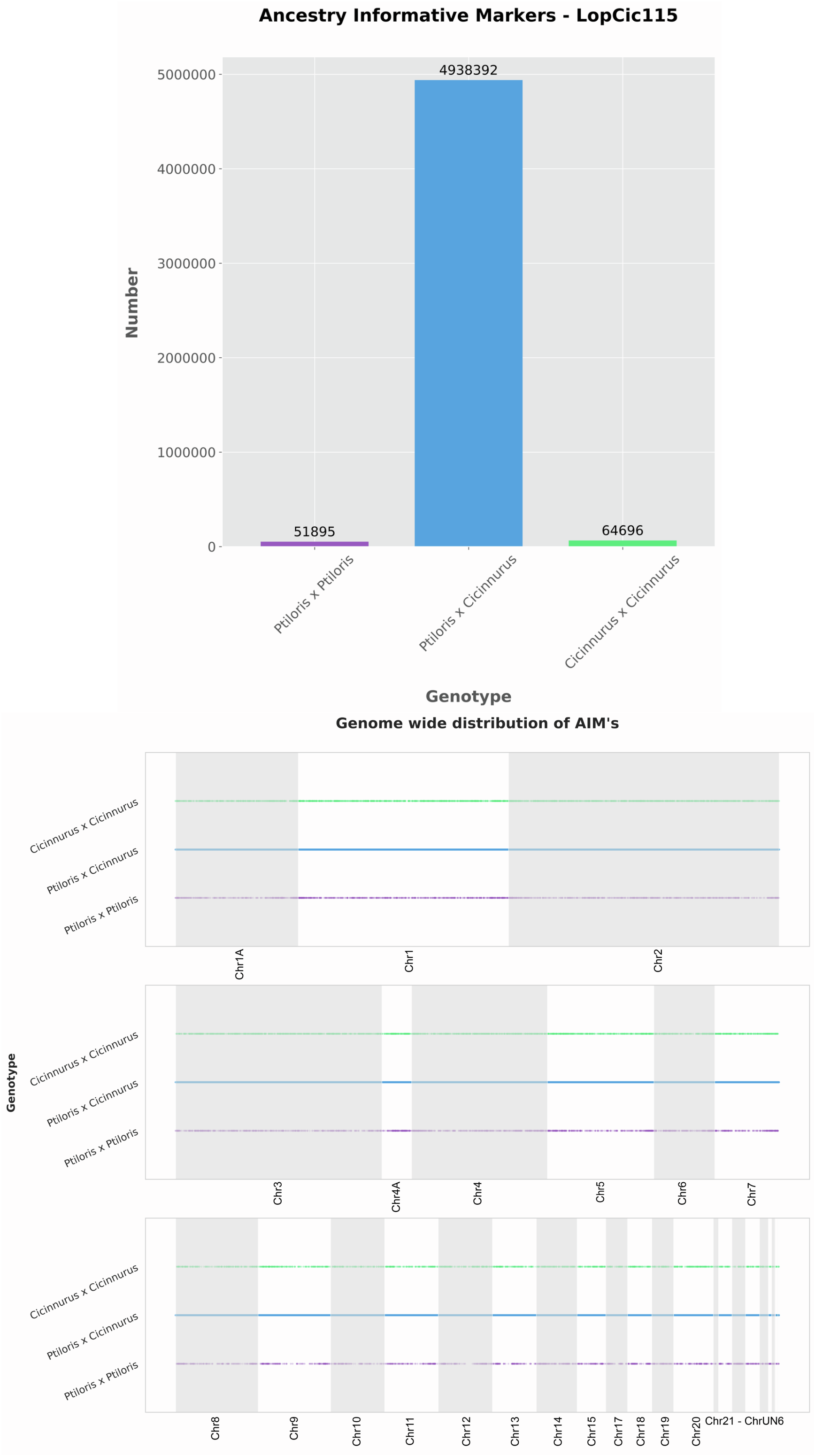

Figure S30. Genotype frequencies and genome-wide distributions of genotypes at Ancestry Informative Marker sites (AIMs) for hybrid individual LopCic115. Related to Figure 1. A.) At AIMs, this individual is almost unequivocally heterozygous suggesting that it is an F1 hybrid. B.) The distribution of homozygous AIMs is relatively random across the genome; suggesting that these are most likely sites that were not fully fixed between parental genera, i.e. not true AIMs, and represent background noise.

A.

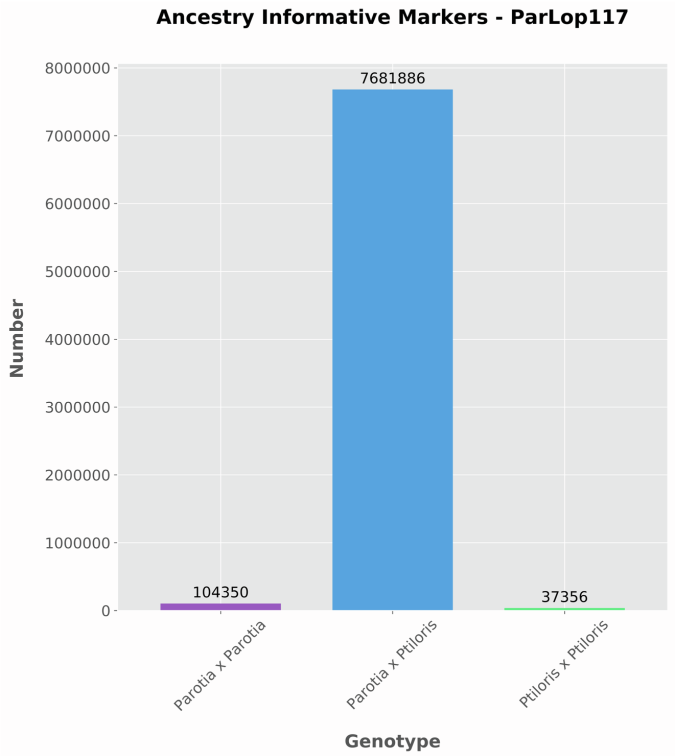

B.

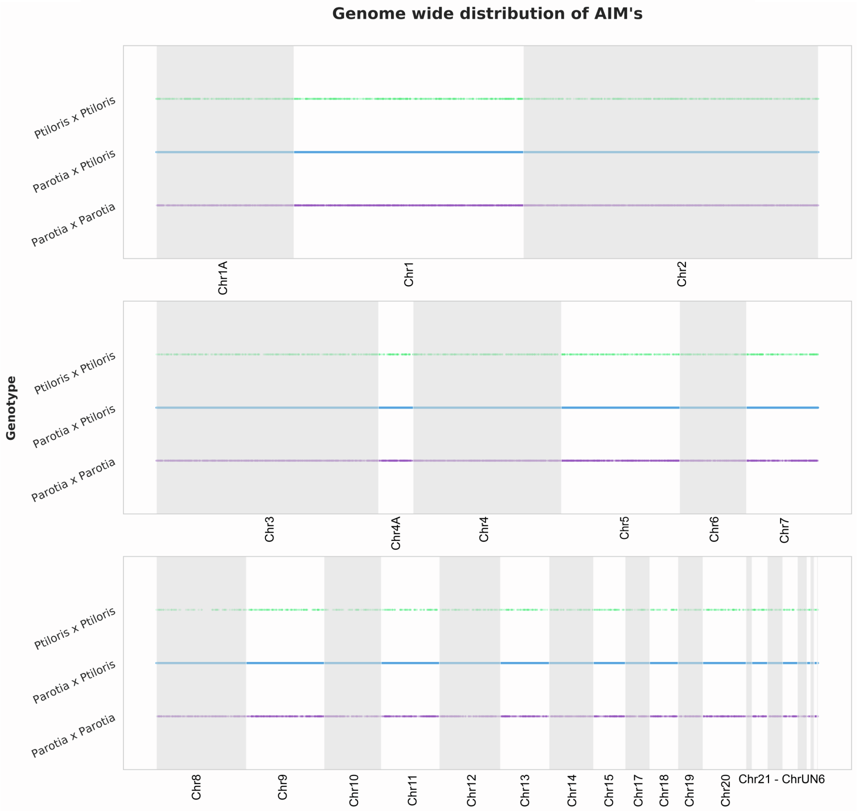

Figure S31. Genotype frequencies and genome-wide distributions of genotypes at Ancestry Informative Marker sites (AIMs) for hybrid individual ParLop117. Related to Figure 1. A.) At AIMs, this individual is almost unequivocally heterozygous suggesting that it is an F1 hybrid. B.) The distribution of homozygous AIMs is relatively random across the genome; suggesting that these are most likely sites that were not fully fixed between parental genera, i.e. not true AIMs, and represent background noise.

A.

B.

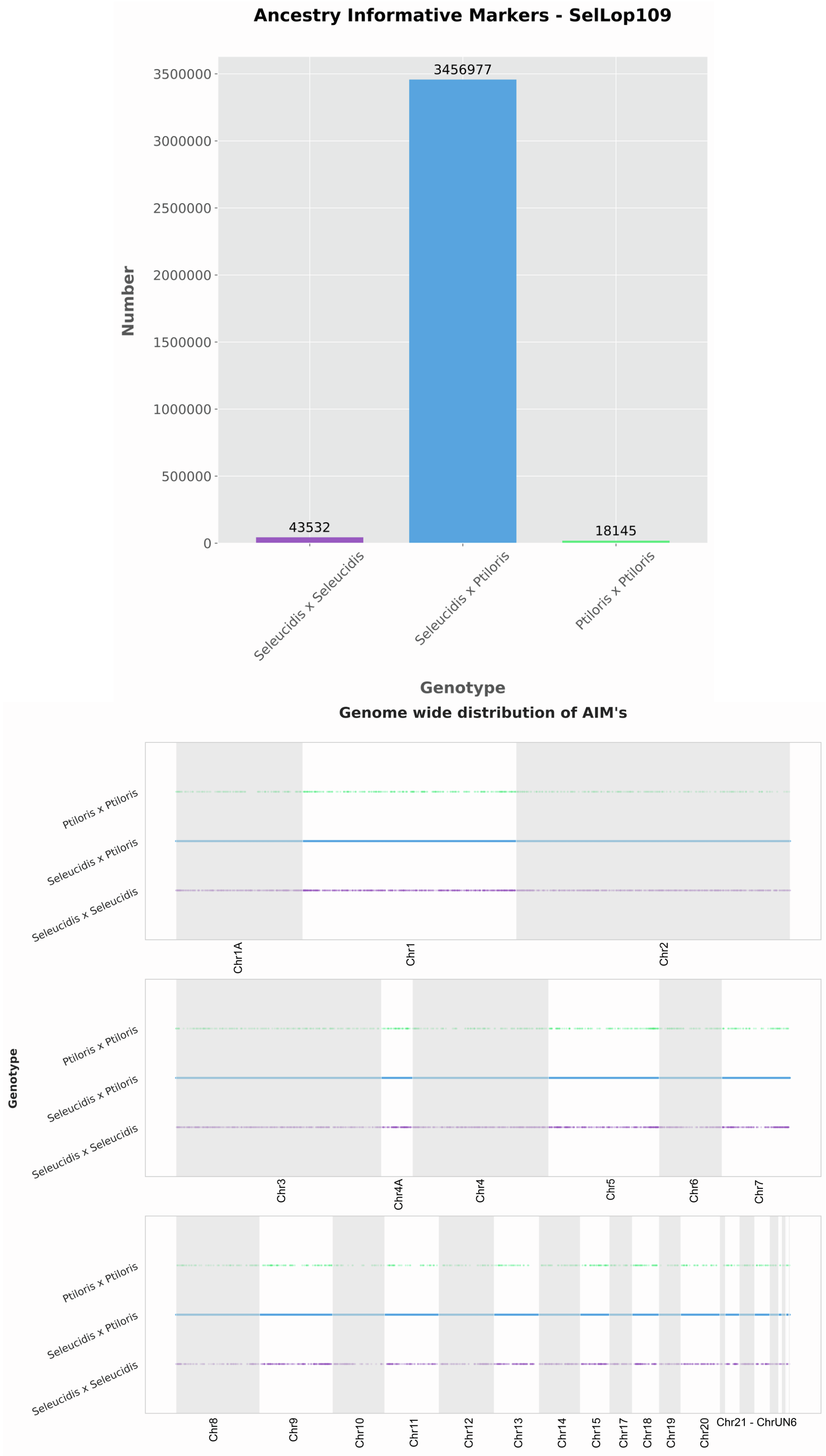

Figure S32. Genotype frequencies and genome-wide distributions of genotypes at Ancestry Informative Marker sites (AIMs) for hybrid individual SelLop109. Related to Figure 1. A.) At AIMs, this individual is almost unequivocally heterozygous suggesting that it is an F1 hybrid. B.) The distribution of homozygous AIMs is relatively random across the genome; suggesting that these are most likely sites that were not fully fixed between parental genera, i.e. not true AIMs, and represent background noise.

A.

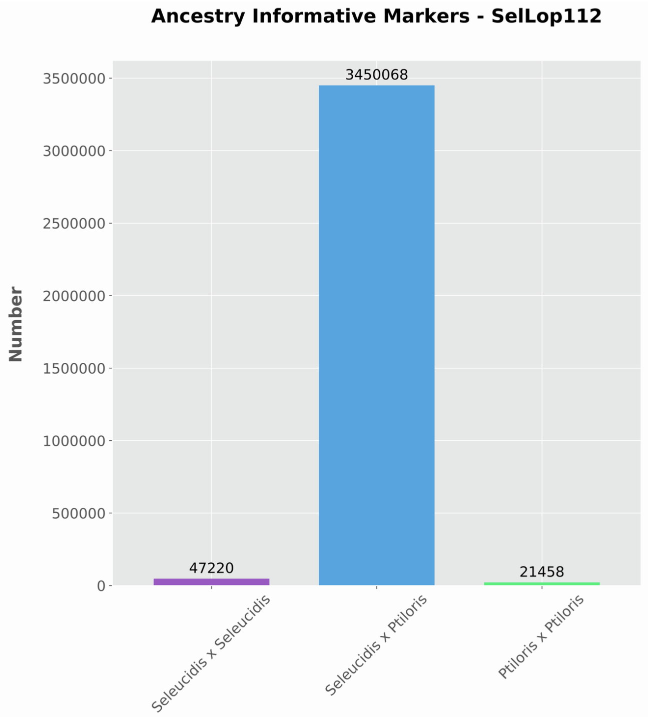

B.

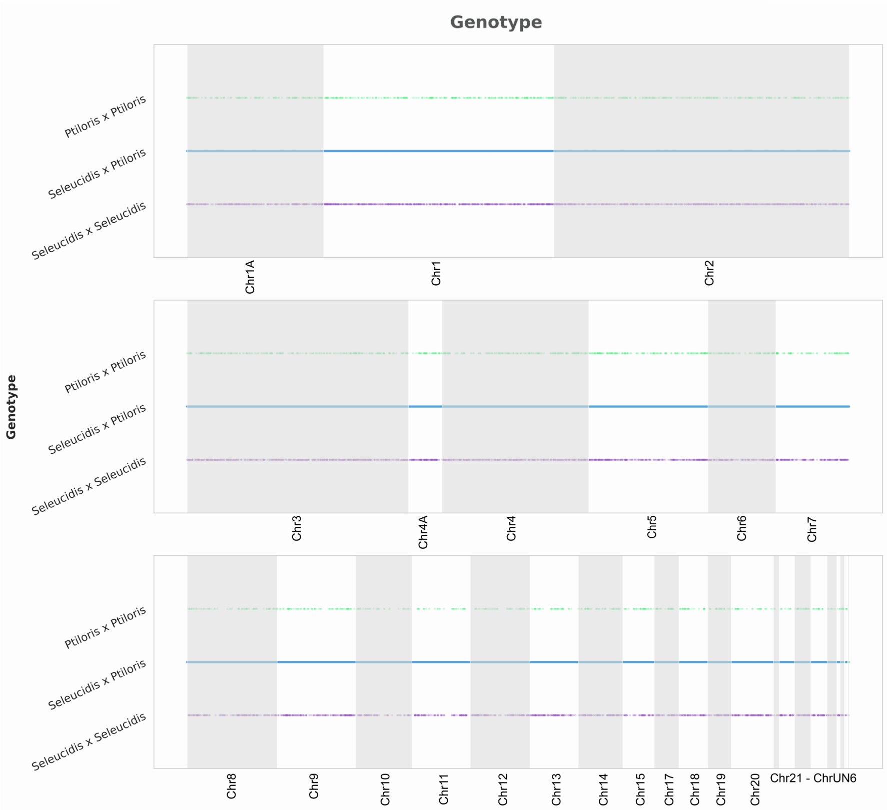

Figure S33. Genotype frequencies and genome-wide distributions of genotypes at Ancestry Informative Marker sites (AIMs) for hybrid individual SelLop112. Related to Figure 1. A.) At AIMs, this individual is almost unequivocally heterozygous suggesting that it is an F1 hybrid. B.) The distribution of homozygous AIMs is relatively random across the genome; suggesting that these are most likely sites that were not fully fixed between parental genera, i.e. not true AIMs, and represent background noise.

A.

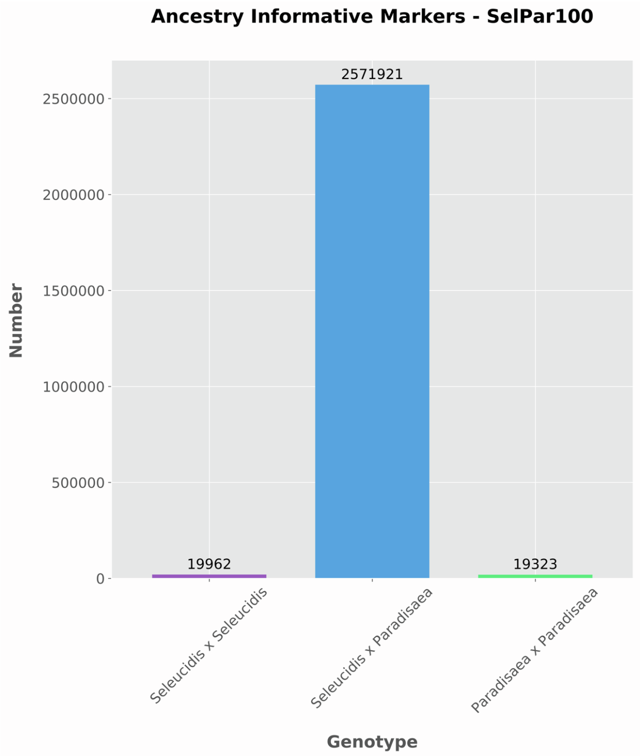

B.

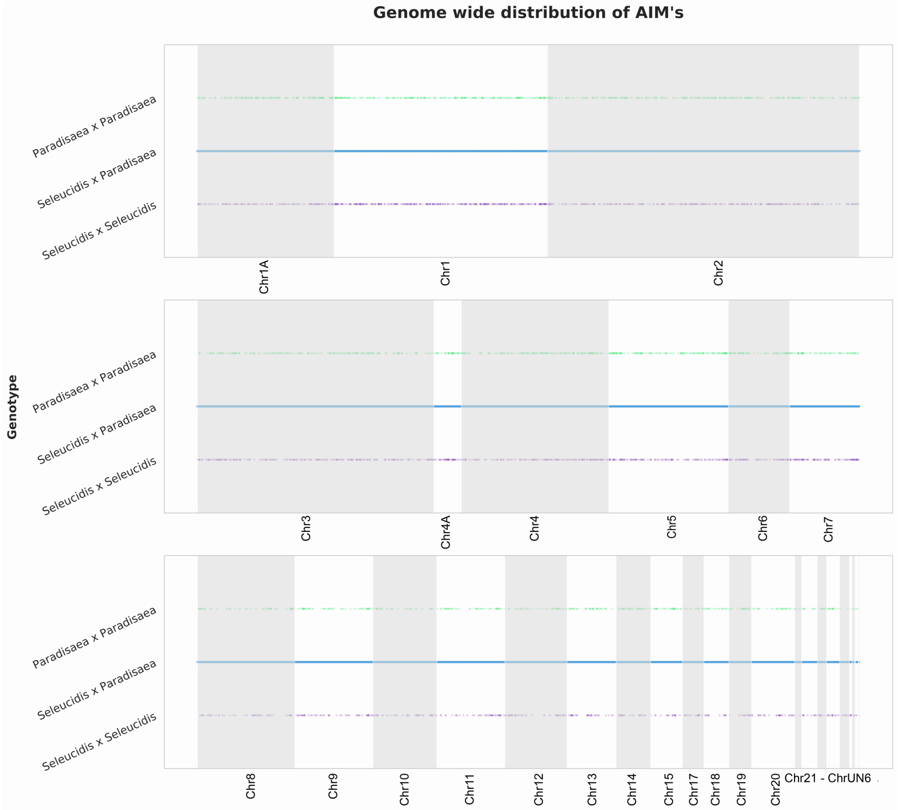

Figure S34. Genotype frequencies and genome-wide distributions of genotypes at Ancestry Informative Marker sites (AIMs) for hybrid individual SelPar100. Related to Figure 1. A.) At AIMs, this individual is almost unequivocally heterozygous suggesting that it is an F1 hybrid. B.) The distribution of homozygous AIMs is relatively random across the genome; suggesting that these are most likely sites that were not fully fixed between parental genera, i.e. not true AIMs, and represent background noise.

A.

B.

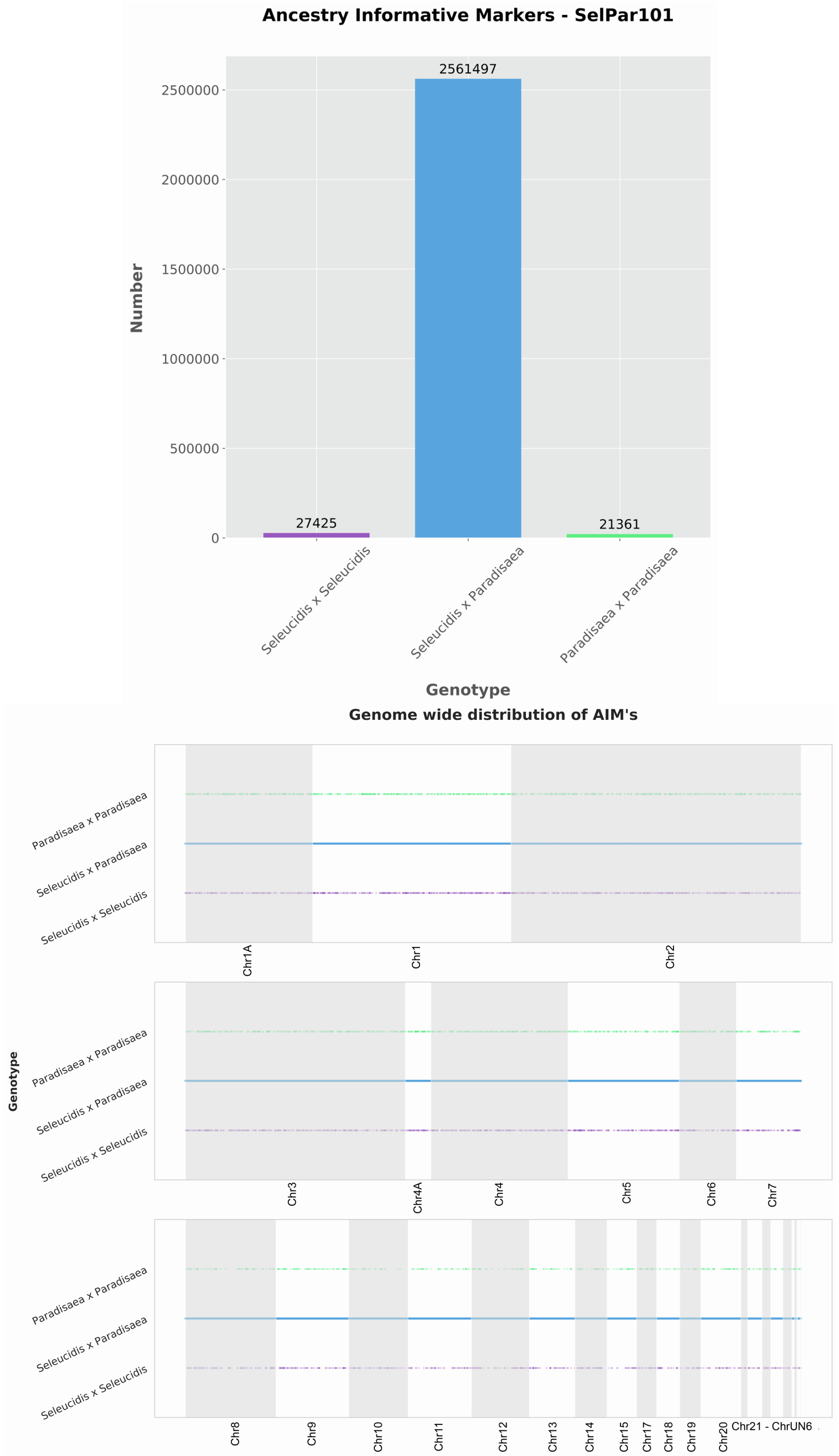

Figure S35. Genotype frequencies and genome-wide distributions of genotypes at Ancestry Informative Marker sites (AIMs) for hybrid individual SelPar101. Related to Figure 1. A.) At AIMs, this individual is almost unequivocally heterozygous suggesting that it is an F1 hybrid. B.) The distribution of homozygous AIMs is relatively random across the genome; suggesting that these are most likely sites that were not fully fixed between parental genera, i.e. not true AIMs, and represent background noise.

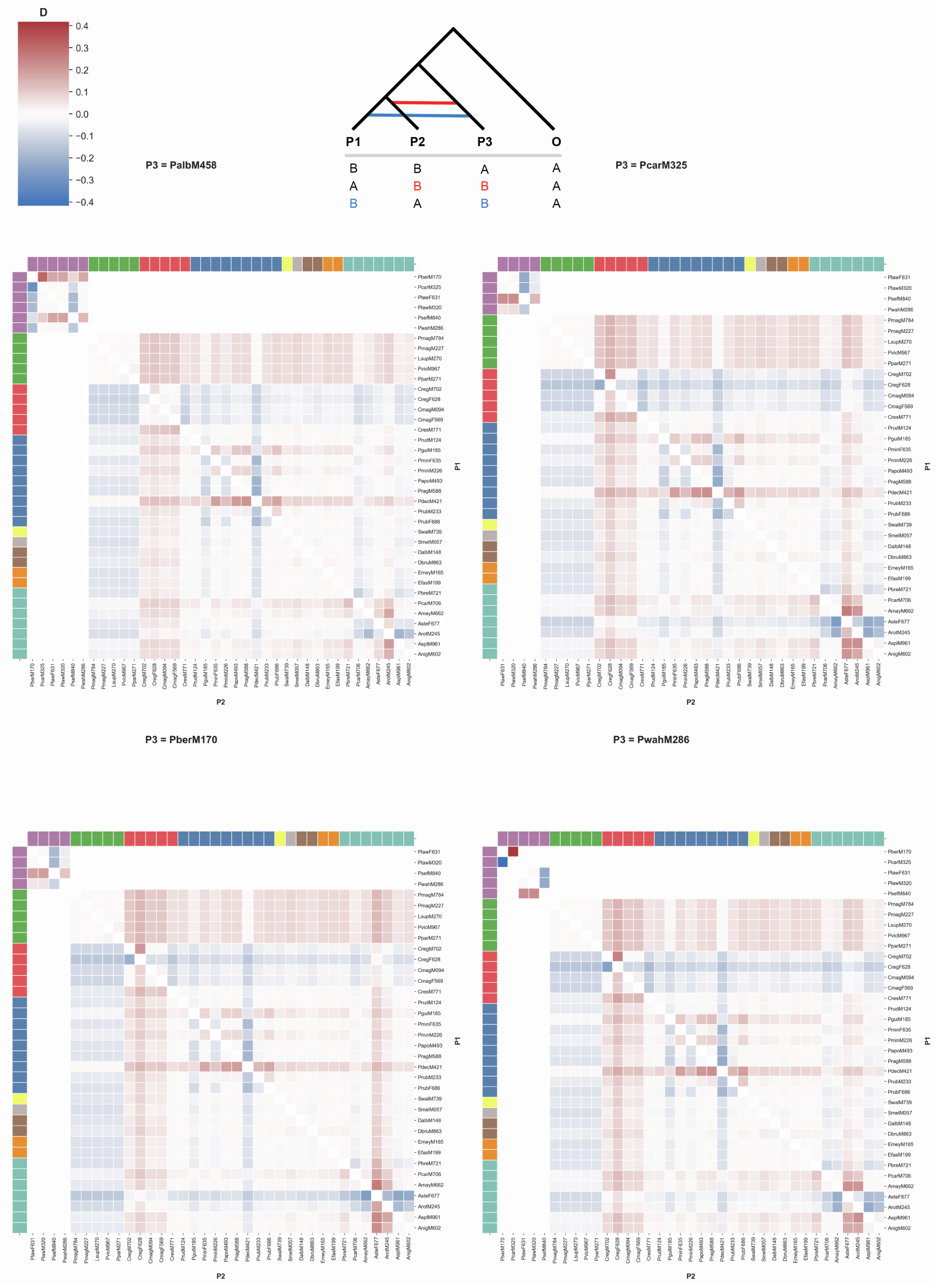

Figure S36. Pairwise Patterson's *D* as a means to identify putative instances of introgression between PalbM458, PcarM325, PberM170 & PwahM286 (P3) individuals and other core Birds-of-Paradise (P1, P2). Related to Figure 2 and S37. The maximum-likelihood species tree based on concatenated 40 kb windows was used to identify phylogenetically relevant comparisons ((P1, P2),P3) and only those comparisons are presented in the matrix. The colour labels on the side of the matrix match the colour coding used for genera throughout the study. A consistent pattern of excess allele sharing observed for all four individuals, supports a history involving ancestral hybridization between *Parotia/Pteridophora* and *Cicinurrus*/*Diphyllodes* and is most pronounced in triads that also include *Ptiloris/Lophorina* (as P1 or P2).

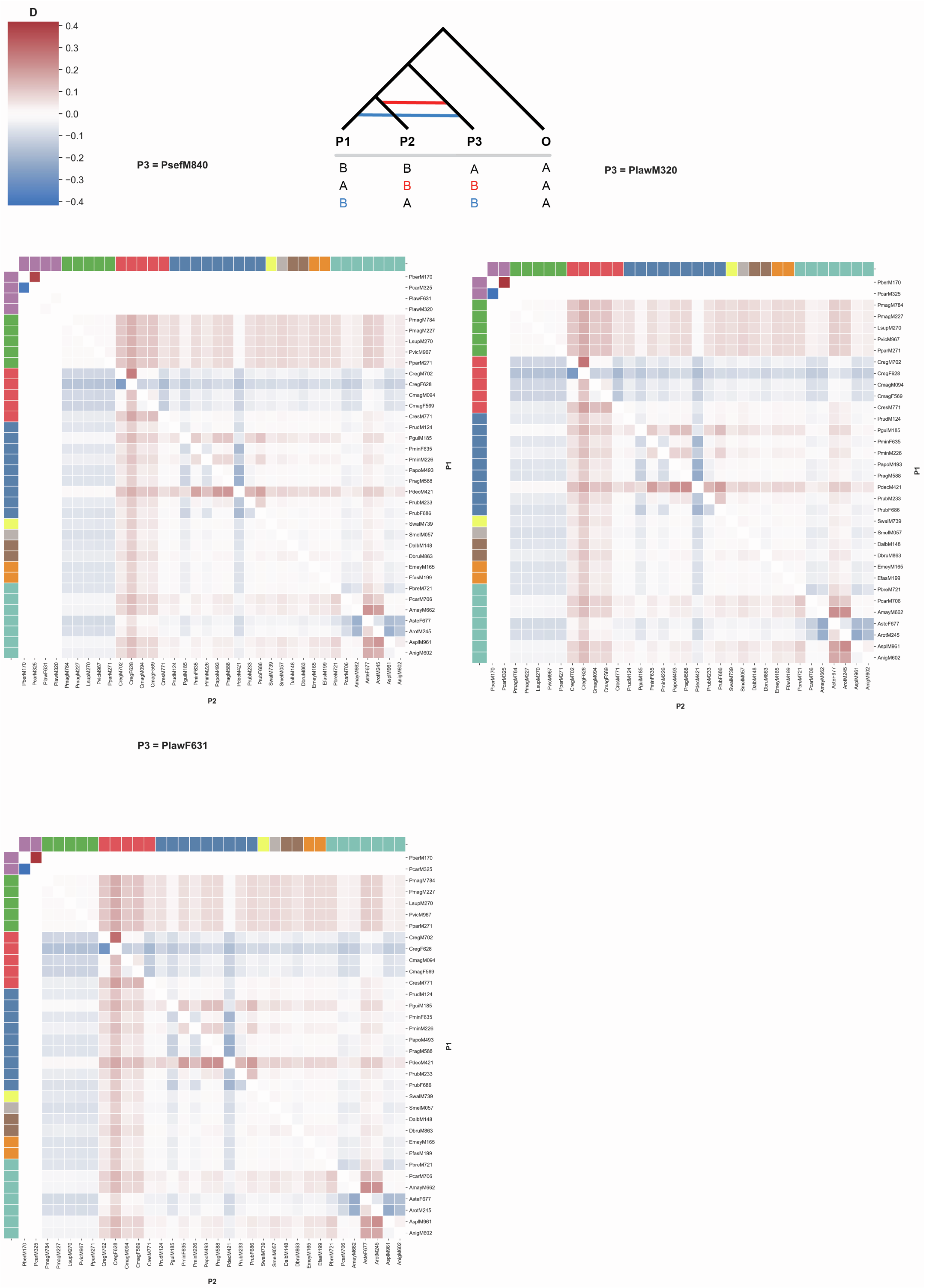

Figure S37. Pairwise Patterson's *D* as a means to identify putative instances of introgression between PsefM840, PlawM320 & PlawF631 (P3) individuals and other core Birds-of-Paradise (P1, P2). Related to Figure 2 and S36. The maximum-likelihood species tree based on concatenated 40 kb windows was used to identify phylogenetically relevant comparisons ((P1, P2),P3) and only those comparisons are presented in the matrix. The colour labels on the side of the matrix match the colour coding used for genera throughout the study. A consistent pattern of excess allele sharing observed for all three individuals, supports a history involving ancestral hybridization between *Parotia/Pteridophora* and *Cicinurrus/Diphyllodes* and is most pronounced in triads that also include *Ptiloris/Lophorina* (as P1 or P2).

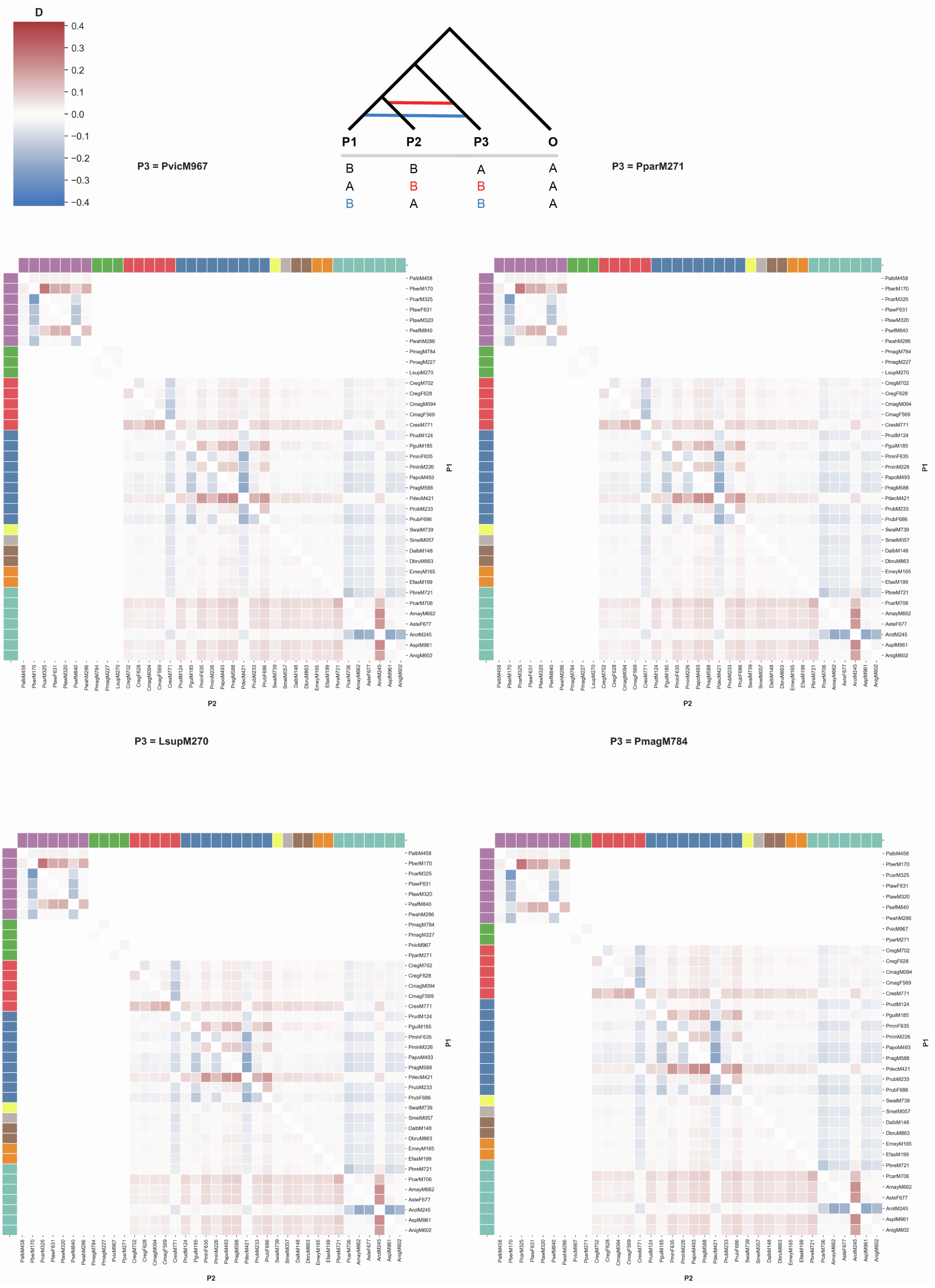

Figure S38. Pairwise Patterson's *D* as a means to identify putative instances of introgression between PvicM967, PparM271, LsupM270 & PmagM784 (P3) individuals and other core Birds-of-Paradise (P1, P2). Related to Figure 2 and S39. The maximum-likelihood species tree based on concatenated 40 kb windows was used as a basis to identify phylogenetically relevant comparisons ((P1, P2),P3) and only those comparisons are presented in the matrix. The colour labels on the side of the matrix match the colour coding used for genera throughout the study. No clear pattern of excess allele sharing between these four individuals and any of the core Bird-of-Paradise lineages apparent. The allele sharing with *Astrapia/Paradigalla carunculata* is discernible in many other combinations (Figure S36-S45) and suggests that *Astrapia/Paradigalla carunculata* may carry some alleles foreign to most other core Bird-of-Paradise lineages.

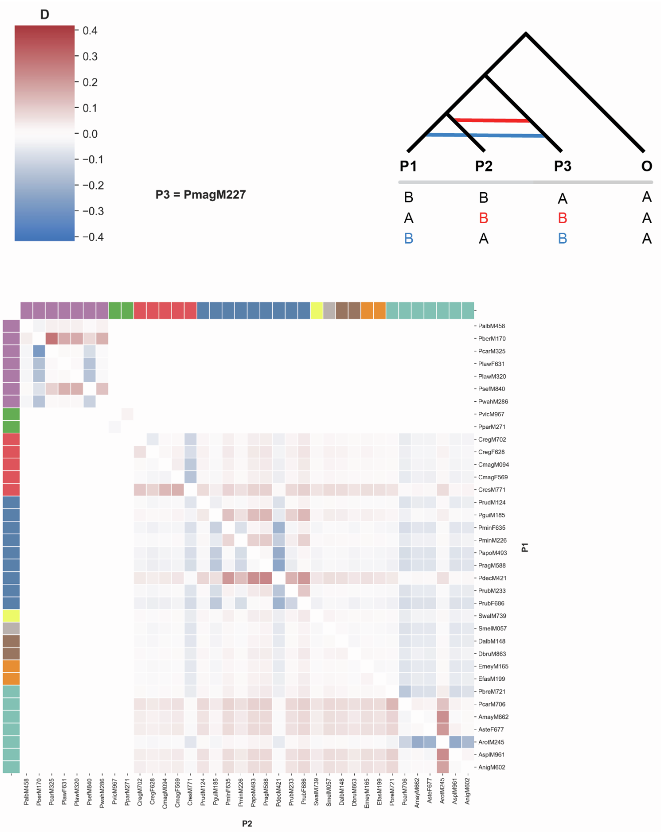

Figure S39. Pairwise Patterson's *D* as a means to identify putative instances of introgression between individual PmagM227 (P3) and other core Birds-of-Paradise (P1, P2). Related to Figure 2 and S38. The maximum-likelihood species tree based on concatenated 40 kb windows was used as a basis to identify phylogenetically relevant comparisons ((P1, P2),P3) and only those comparisons are presented in the matrix. The colour labels on the side of the matrix match the colour coding used for genera throughout the study. No clear pattern of excess allele sharing between this individual and any of the core Bird-of-Paradise lineages apparent. The allele sharing with *Astrapia/Paradigalla carunculata* is discernible in many other combinations (Figure S36-S45) and suggests that *Astrapia/Paradigalla carunculata* may carry some alleles foreign to most other core Bird-of-Paradise lineages.

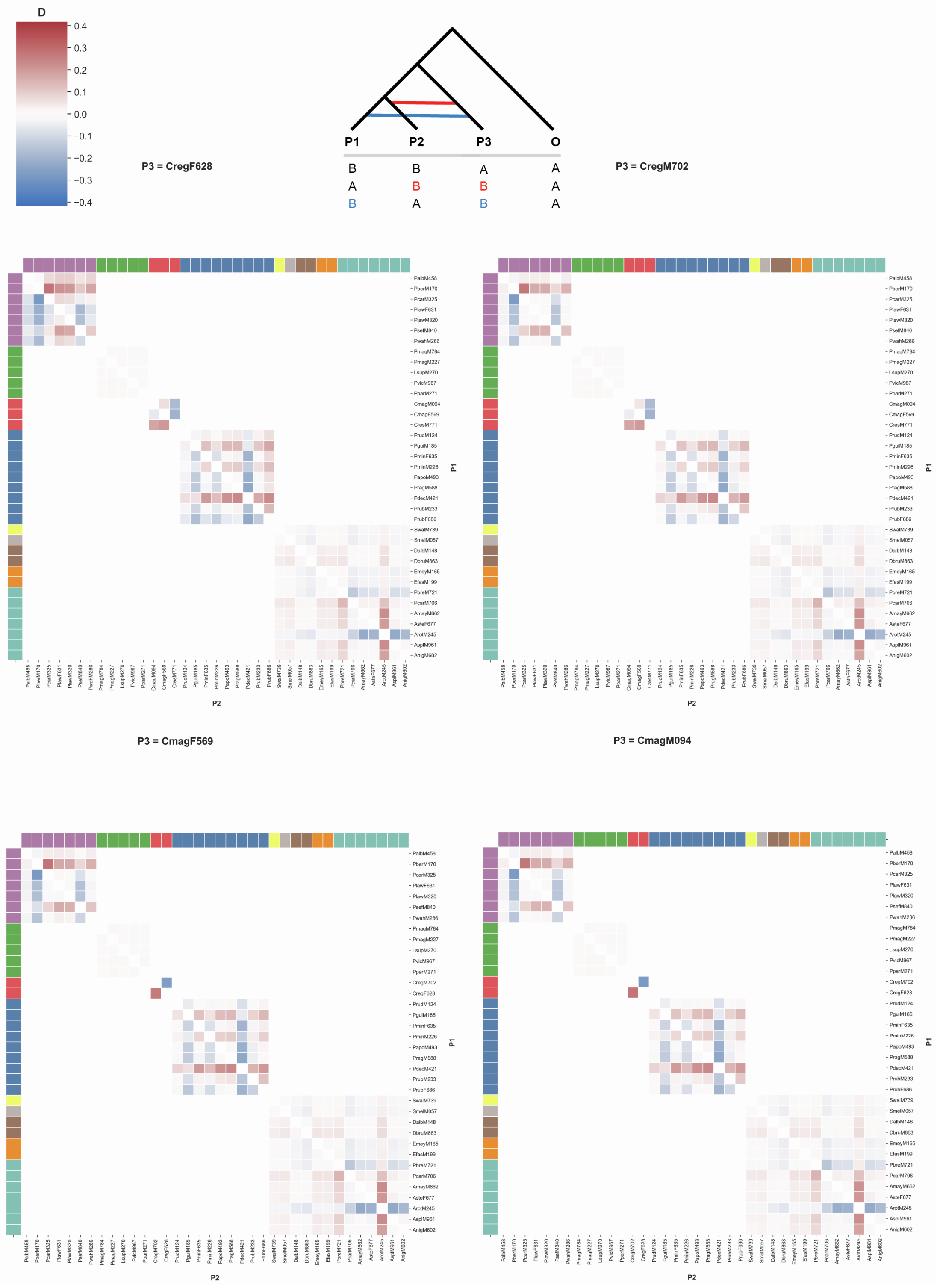

Figure S40. Pairwise Patterson's *D* as a means to identify putative instances of introgression between CregF628, CregM702, CmagF569 & CmagM094 (P3) individuals and other core Birds-of-Paradise (P1, P2). Related to Figure 2 and S41. The maximum-likelihood species tree based on concatenated 40 kb windows was used as a basis to identify phylogenetically relevant comparisons ((P1, P2),P3) and only those comparisons are presented in the matrix. The colour labels on the side of the matrix match the colour coding used for genera throughout the study. No clear pattern of excess allele sharing due to ancestral hybridization between these individuals and any of the core Bird-of-Paradise lineages apparent. In these comparisons, with *Cicinnurus*/*Diphyllodes* as P3, the ancestral hybridization with *Pteridophora/Parotia* is not explicitly tested for given the species tree.

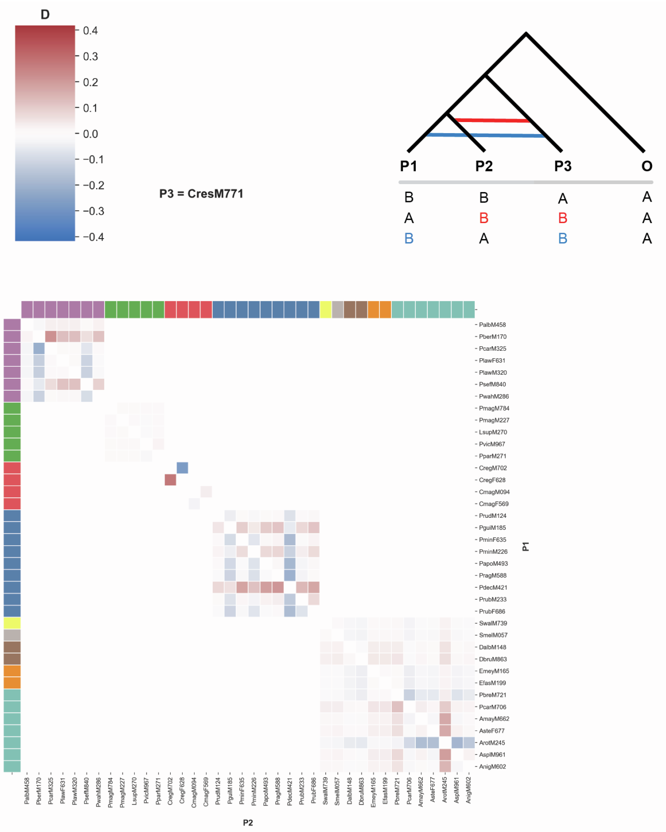

Figure S41. Pairwise Patterson's *D* as a means to identify putative instances of introgression between individual CresM771 (P3) and other core Birds-of-Paradise (P1, P2). Related to Figure 2 and S40. The maximum-likelihood species tree based on concatenated 40 kb windows was used as a basis to identify phylogenetically relevant comparisons ((P1, P2),P3) and only those comparisons are presented in the matrix. The colour labels on the side of the matrix match the colour coding used for genera throughout the study. No clear pattern of excess allele sharing due to ancestral hybridization between these individuals and any of the core Bird-of-Paradise lineages apparent. In these comparisons, with *Cicinnurus*/*Diphyllodes* as P3, the ancestral hybridization with *Pteridophora/Parotia* is not explicitly tested for given the species tree.

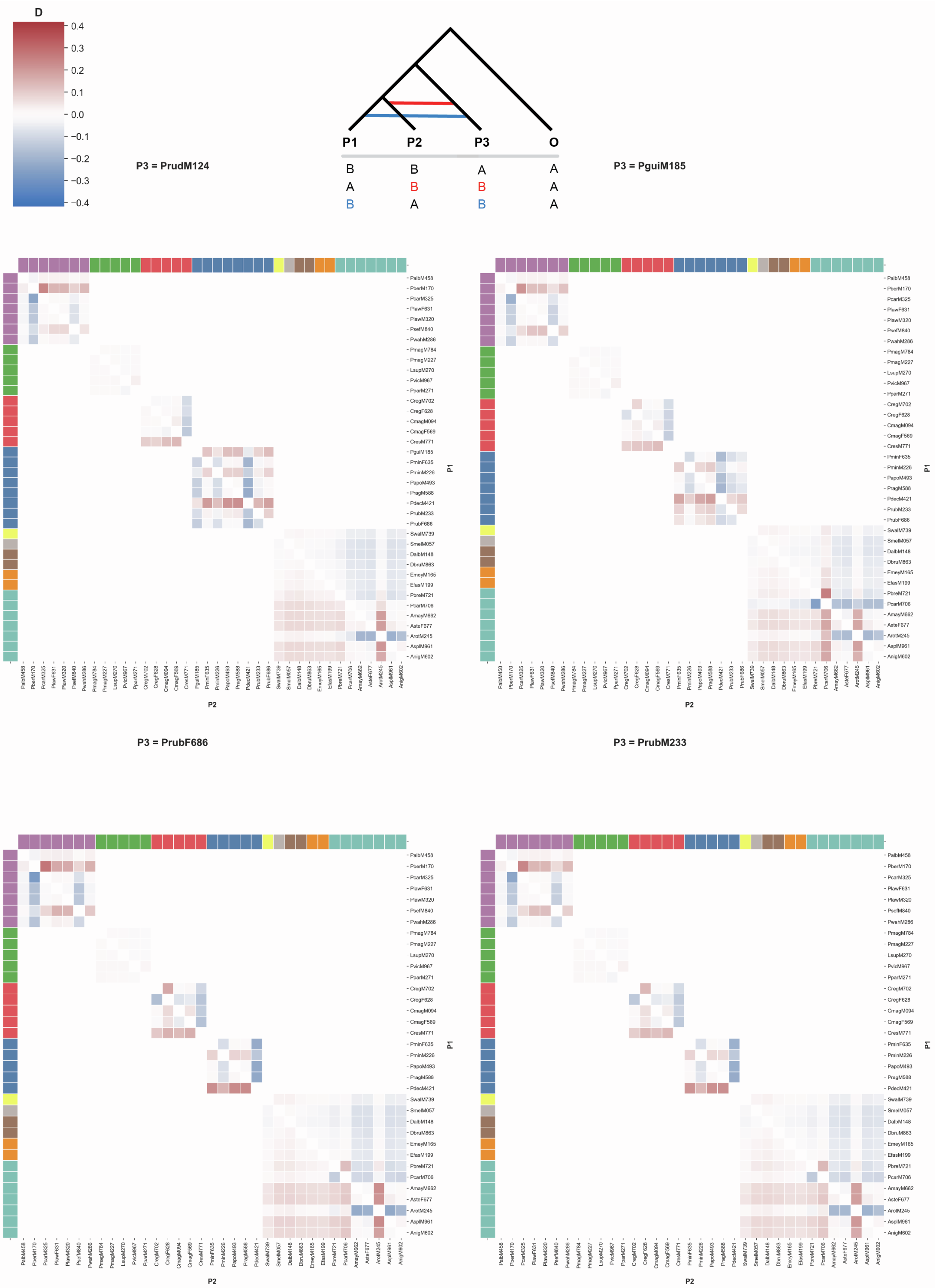

Figure S42. Pairwise Patterson's *D* as a means to identify putative instances of introgression between PrudM124, PguiM185, PrubF686 & PrubM233 (P3) individuals and other core Birds-of-Paradise (P1, P2). Related to Figure 2, S43 & S44. The maximum-likelihood species tree based on concatenated 40 kb windows was used as a basis to identify phylogenetically relevant comparisons ((P1, P2),P3) and only those comparisons are presented in the matrix. The colour labels on the side of the matrix match the colour coding used for genera throughout the study. No clear pattern of excess allele sharing between these individuals and any of the core Bird-of-Paradise lineages apparent. The allele sharing with *Astrapia/Paradigalla carunculata* is discernible in many other combinations and suggests that *Astrapia/Paradigalla carunculata* may carry some alleles foreign to most other core Bird-of-Paradise lineages.

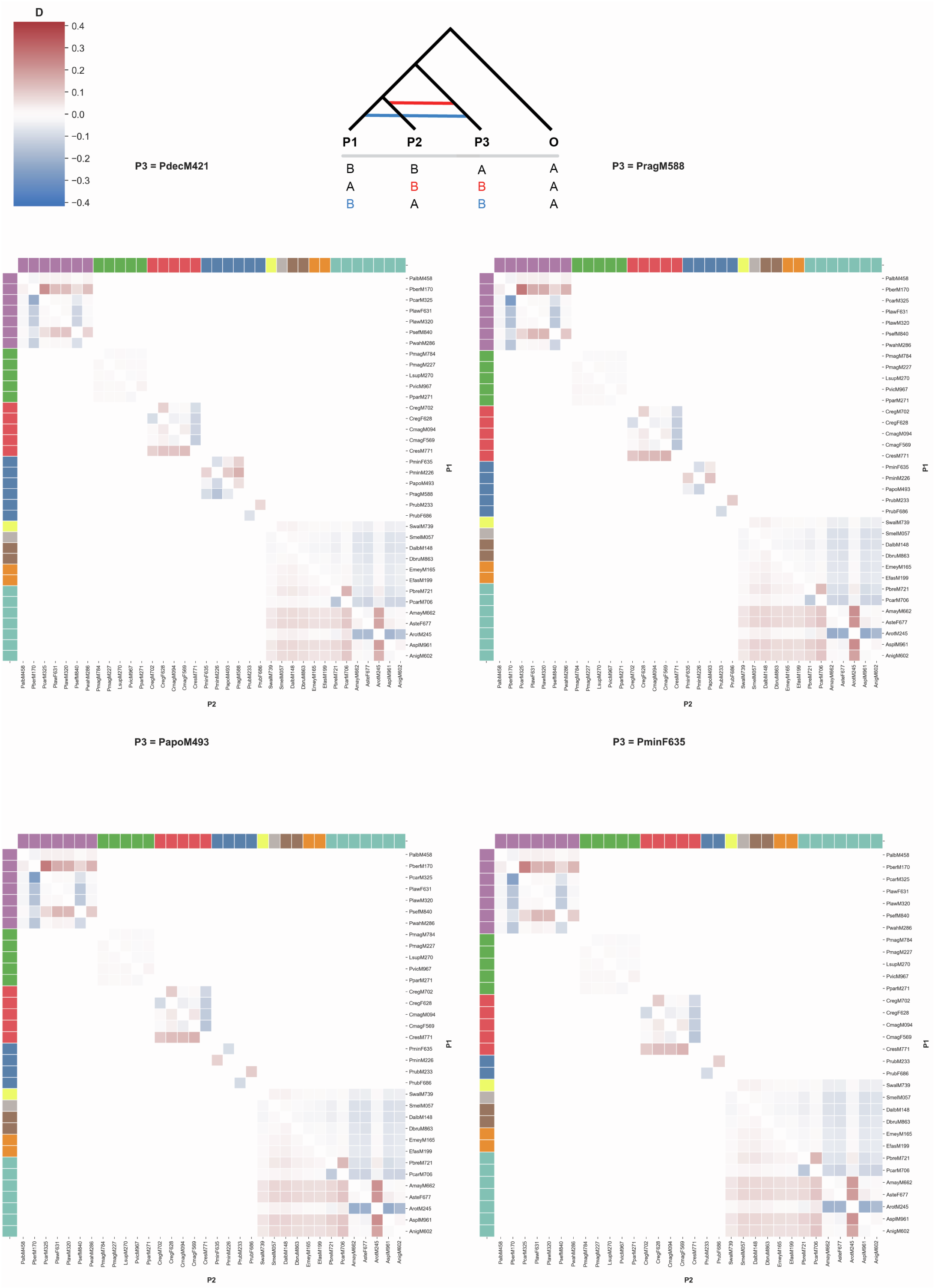

Figure S43. Pairwise Patterson's *D* as a means to identify putative instances of introgression between PdecM421, PragM588, PapoM493 & PminF635 (P3) individuals and other core Birds-of-Paradise (P1, P2). Related to Figure 2, S42 & S44. The maximum-likelihood species tree based on concatenated 40 kb windows was used as a basis to identify phylogenetically relevant comparisons ((P1, P2),P3) and only those comparisons are presented in the matrix. The colour labels on the side of the matrix match the colour coding used for genera throughout the study. No clear pattern of excess allele sharing between these individuals and any of the core Bird-of-Paradise lineages apparent. The allele sharing with *Astrapia/Paradigalla carunculata* is discernible in many other combinations and suggests that *Astrapia/Paradigalla carunculata* may carry some alleles foreign to most other core Bird-of-Paradise lineages.

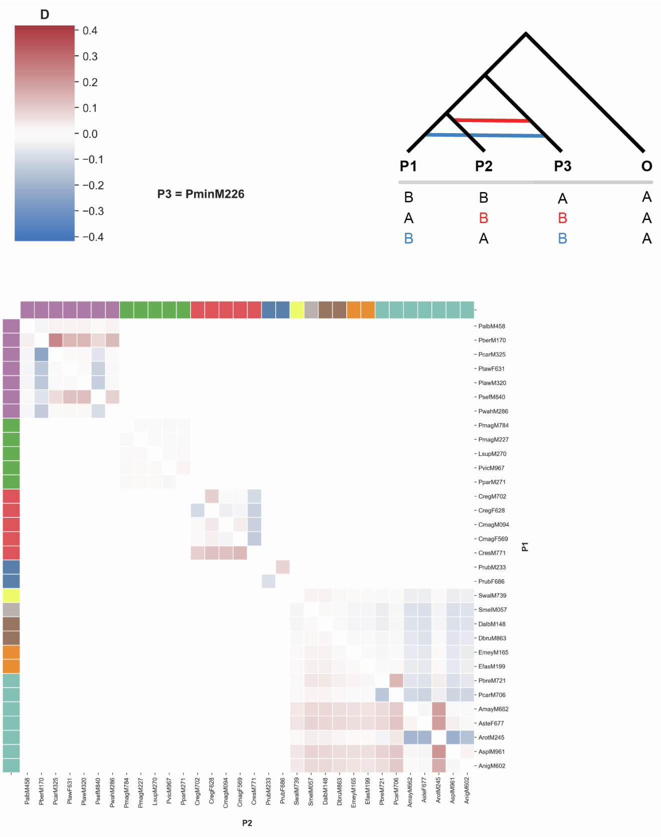

Figure S44. Pairwise Patterson's *D* as a means to identify putative instances of introgression between individual PminM226 (P3) and other core Birds-of-Paradise (P1, P2). Related to Figure 2, S42 & S43. The maximum-likelihood species tree based on concatenated 40 kb windows was used as a basis to identify phylogenetically relevant comparisons ((P1, P2),P3) and only those comparisons are presented in the matrix. The colour labels on the side of the matrix match the colour coding used for genera throughout the study. No clear pattern of excess allele sharing between these PminM226 and any of the core Bird-of-Paradise lineages apparent. The allele sharing with *Astrapia/Paradigalla carunculata* is discernible in many other combinations and suggests that *Astrapia/Paradigalla carunculata* may carry some alleles foreign to most other core Bird-of-Paradise lineages.

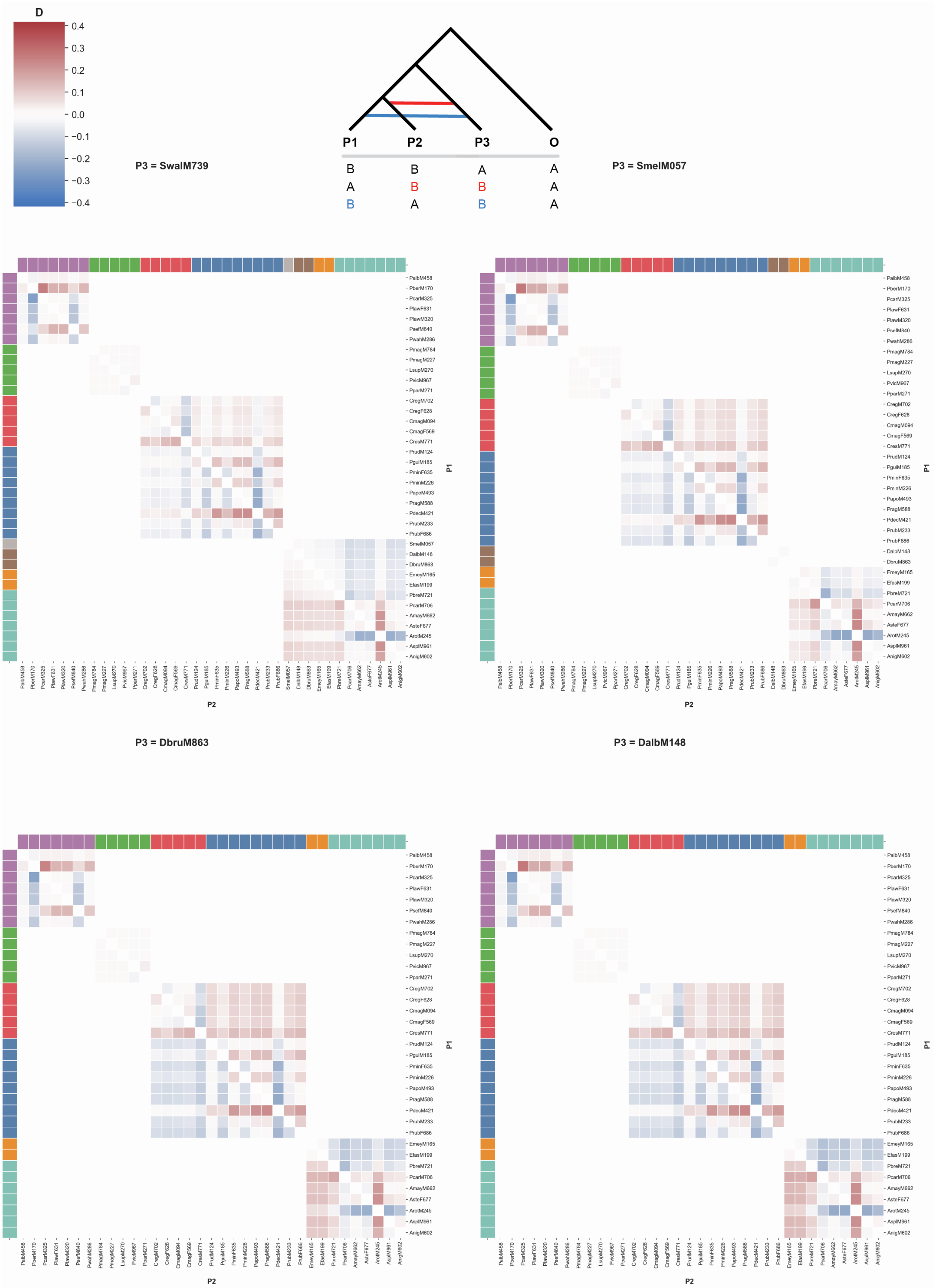

Figure S45. Pairwise Patterson's *D* as a means to identify putative instances of introgression between SwalM739, SmelM057, DbruM863 & DalbM148 (P3) individuals and other core Birds-of-Paradise (P1, P2). Related to Figure 2. The maximum-likelihood species tree based on concatenated 40 kb windows was used as a basis to identify phylogenetically relevant comparisons ((P1, P2),P3) and only those comparisons are presented in the matrix. The colour labels on the side of the matrix match the colour coding used for genera throughout the study. *Cicinurrus*/*Diphyllodes* share an excess of alleles with *Drepanornis* individuals, in particular, supporting a history of ancestral hybridization between these lineages. However the placement of *Cicinurrus*/*Diphyllodes* in the species tree is challenging based on autosomal data alone. *Drepanornis* individuals also share an excess of alleles with *Epimachus* relative to *Paradigalla/Astrapia*, but it is challenging to evaluate this signal given the more broader observed allele sharing between *Astrapia/Paradigalla carunculata* and an unknown taxa.

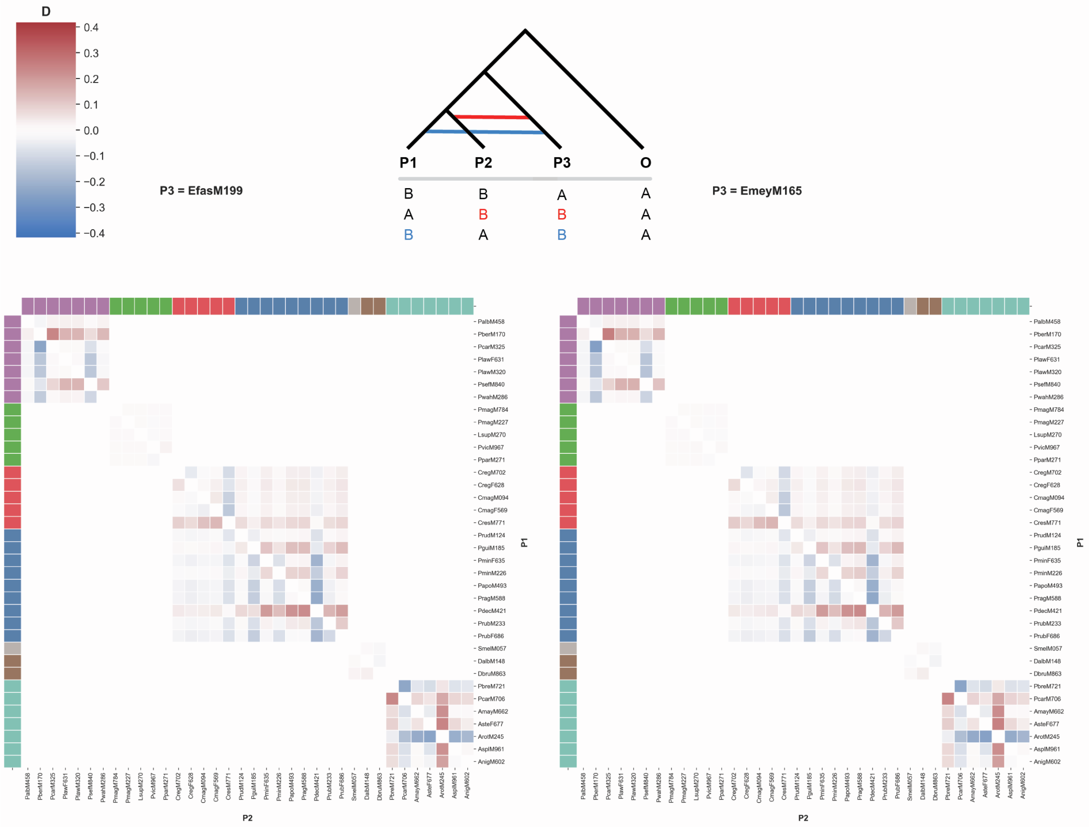

Figure S46. Pairwise Patterson's *D* as a means to identify putative instances of introgression between any of the *Epimachus* (P3) individuals, EfasM199 or EmeyM165, and other core Birds-of-Paradise (P1, P2). Related to Figure 2. The maximum-likelihood species tree based on concatenated 40 kb windows was used as a basis to identify phylogenetically relevant comparisons ((P1, P2),P3) and only those comparisons are presented in the matrix. The colour labels on the side of the matrix match the colour coding used for genera throughout the study. No consistent signal of ancestral hybridization observed.

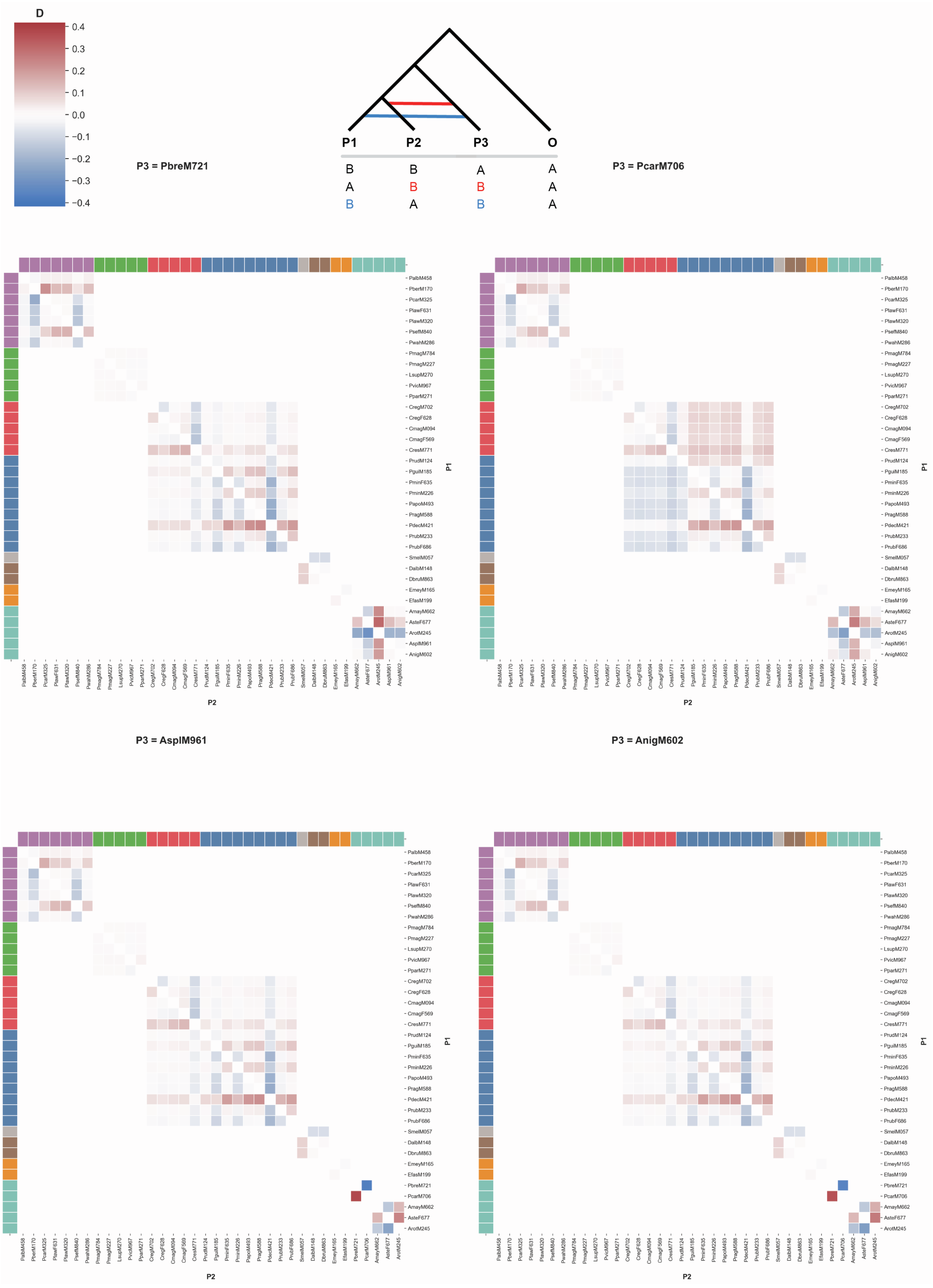

Figure S47. Pairwise Patterson's *D* as a means to identify putative instances of introgression between PbreM721, PcarM706, AsplM961 & AnigM602 (P3) individuals and other core Birds-of-Paradise (P1, P2). Related to Figure 2 & S48. The maximum-likelihood species tree based on concatenated 40 kb windows was used as a basis to identify phylogenetically relevant comparisons ((P1, P2),P3) and only those comparisons are presented in the matrix. The colour labels on the side of the matrix match the colour coding used for genera throughout the study. No clear pattern of excess allele sharing between these individuals and any of the core Bird-of-Paradise lineages apparent, except for *Paradisaea* and *Paradigalla carunculata* (*PcarM706*). Excess allele sharing between *Paradigalla carunculata* and *Paradisaea*, relative to *Cicinurrus/Diphyllodes/Paradisornis*, supports a history involving hybridization between these lineages.

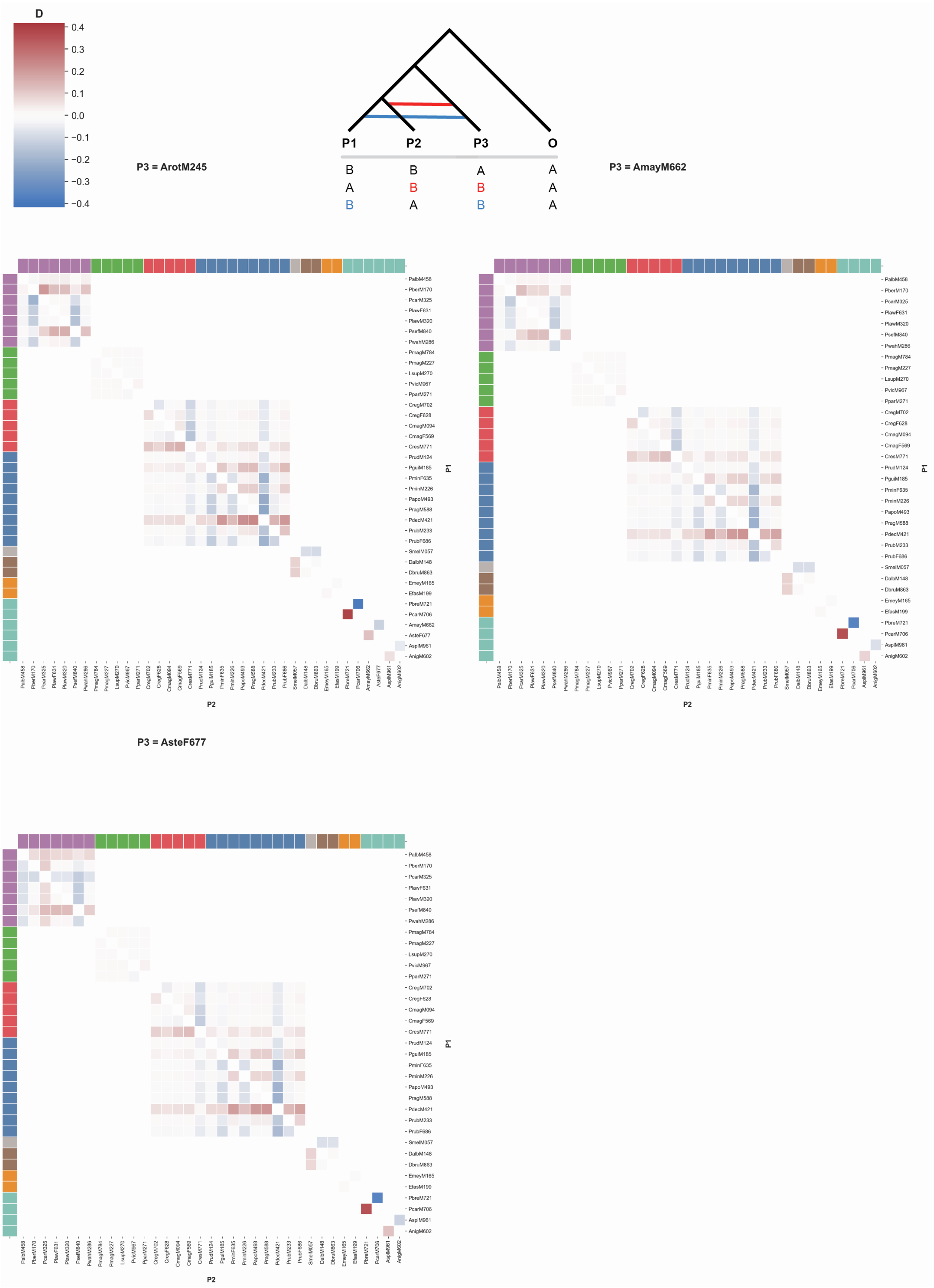

Figure S48. Pairwise Patterson's *D* as a means to identify putative instances of introgression between ArotM245, AmayM662 & AsteF677 (P3) individuals and other core Birds-of-Paradise (P1, P2). Related to Figure 2 & S47. The maximum-likelihood species tree based on concatenated 40 kb windows was used as a basis to identify phylogenetically relevant comparisons ((P1, P2),P3) and only those comparisons are presented in the matrix. The colour labels on the side of the matrix match the colour coding used for genera throughout the study. No consistent signal of ancestral hybridization observed.

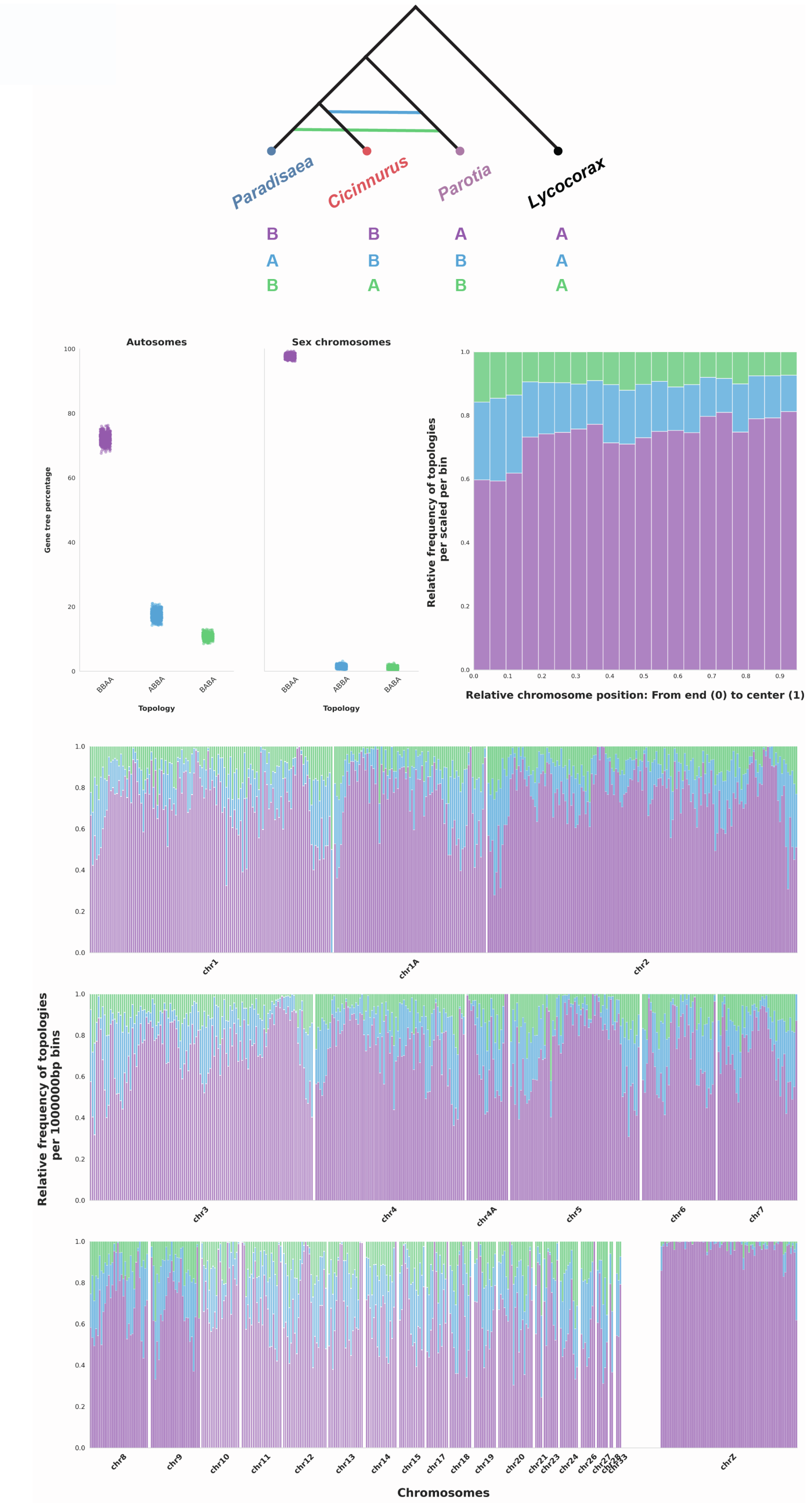

Figure S49. Frequency and genome wide distribution of window tree topologies for each possible Cartesian combination of individuals across four genera. Related to Figure 2 & S50-59. The top-left figure has the relative frequency of ABBA, BABA and BBAA topologies plotted for each of the Cartesian combinations, the top right figure has the distribution of topologies plotted by scaled chromosome position (10 macrochromosomes, summed across all Cartesian combinations) and the bottom plot illustrates the distribution of topologies in bins of 1 Mb. for all chromosomes (summed across all Cartesian combinations). Besides the species tree (BBAA), there is an excess of trees where *Parotia* and *Cicinnurus/Diphyllodes* are clustered (ABBA), relative to trees where *Parotia* and *Cicinnurus/Diphyllodes* are clustered (BABA). ABBA topologies are more frequently found towards the end of chromosomes.

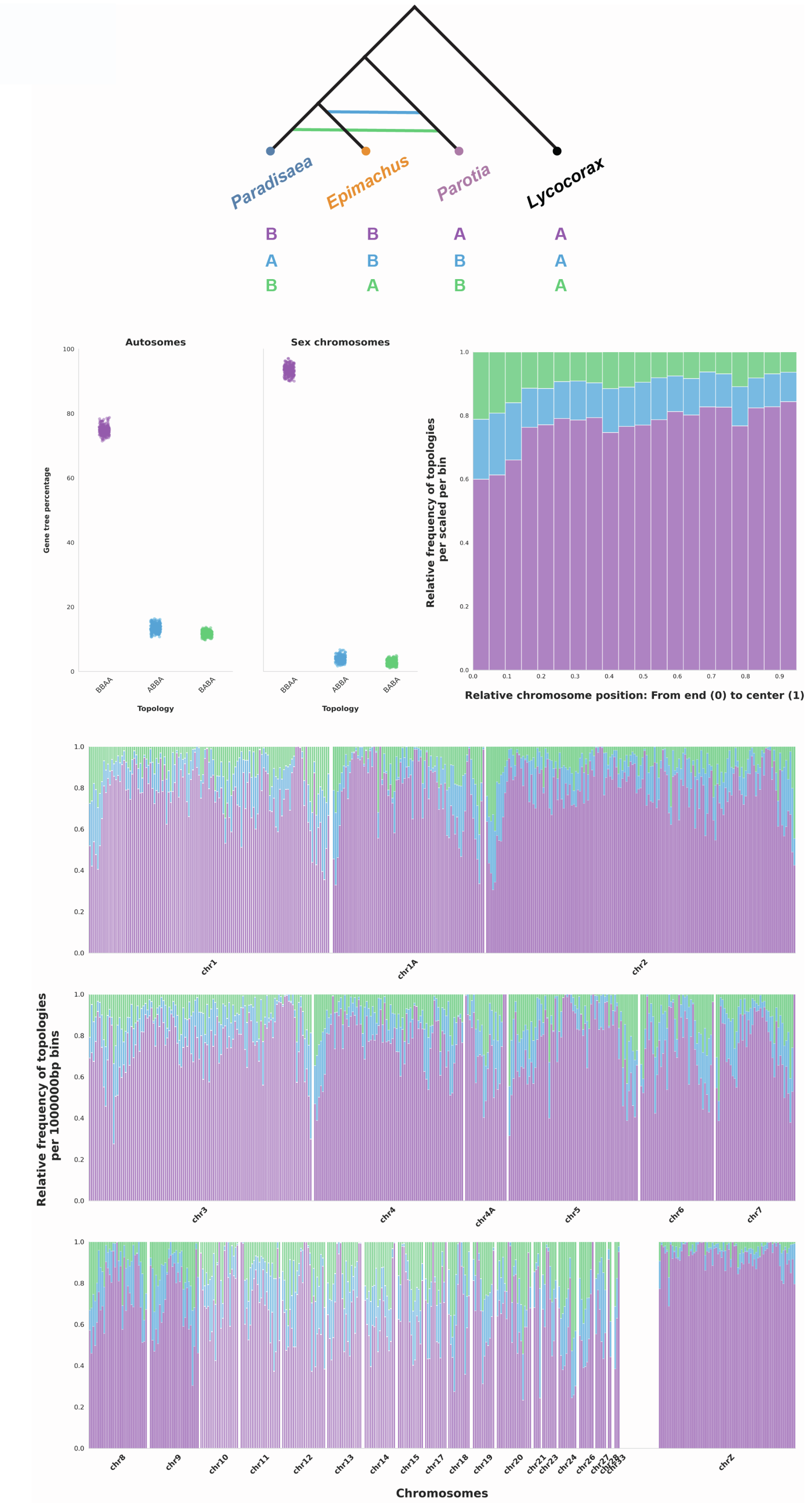

Figure S50. Frequency and genome wide distribution of window tree topologies for each possible Cartesian combination of individuals across four genera. Related to Figure 2 & S49-59. The top-left figure has the relative frequency of ABBA, BABA and BBAA topologies plotted for each of the Cartesian combinations, the top right figure has the distribution of topologies plotted by scaled chromosome position (10 macrochromosomes, summed across all Cartesian combinations) and the bottom plot illustrates the distribution of topologies in bins of 1 Mb. for all chromosomes (summed across all Cartesian combinations). Besides the species tree (BBAA), there is an equal proportion of trees where *Parotia* and *Epimachus* are clustered (ABBA), relative to trees where *Parotia* and *Paradisaea* are clustered (BABA).

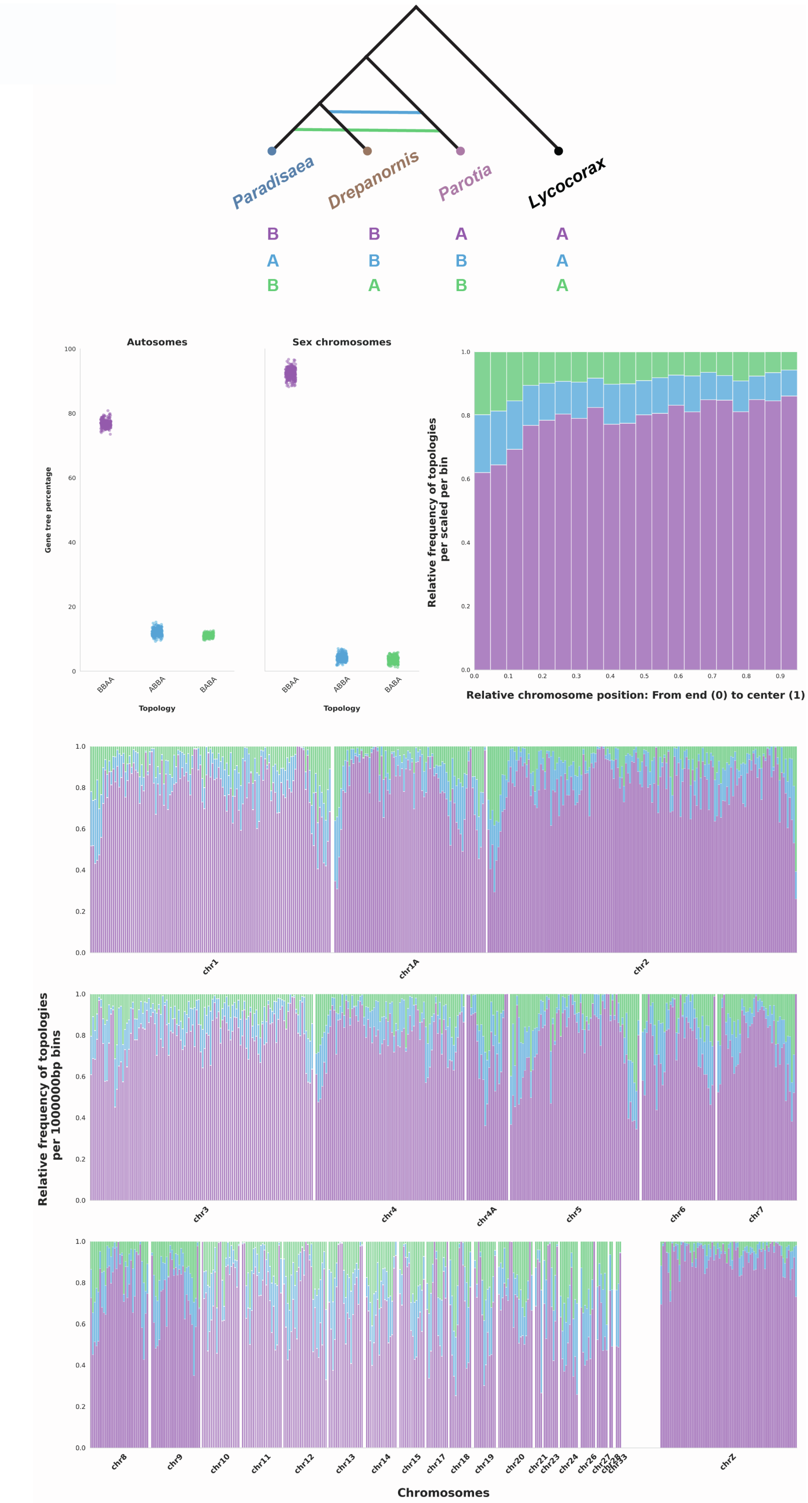

Figure S51. Frequency and genome wide distribution of window tree topologies for each possible Cartesian combination of individuals across four genera. Related to Figure 2 & S49-59. The top-left figure has the relative frequency of ABBA, BABA and BBAA topologies plotted for each of the Cartesian combinations, the top right figure has the distribution of topologies plotted by scaled chromosome position (10 macrochromosomes, summed across all Cartesian combinations) and the bottom plot illustrates the distribution of topologies in bins of 1 Mb. for all chromosomes (summed across all Cartesian combinations). Besides the species tree (BBAA), there is an equal proportion of trees where *Parotia* and *Drepanornis* are clustered (ABBA), relative to trees where *Parotia* and *Paradisaea* are clustered (BABA).

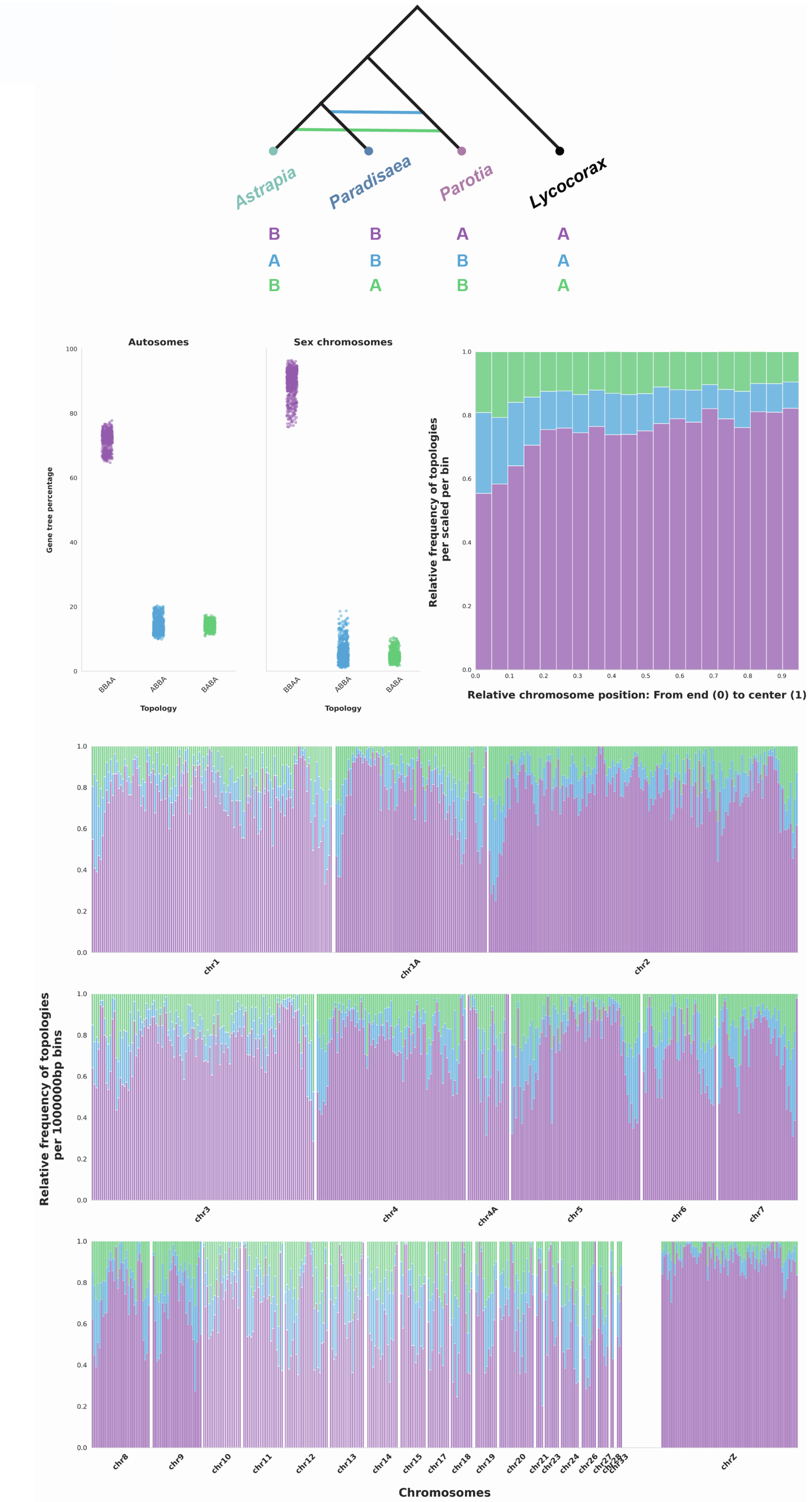

Figure S52. Frequency and genome wide distribution of window tree topologies for each possible Cartesian combination of individuals across four genera. Related to Figure 2 & S49-59. The top-left figure has the relative frequency of ABBA, BABA and BBAA topologies plotted for each of the Cartesian combinations, the top right figure has the distribution of topologies plotted by scaled chromosome position (10 macrochromosomes, summed across all Cartesian combinations) and the bottom plot illustrates the distribution of topologies in bins of 1 Mb. for all chromosomes (summed across all Cartesian combinations). Besides the species tree (BBAA), there is an equal proportion of trees where *Parotia* and *Paradisaea* are clustered (ABBA), relative to trees where *Parotia* and *Astrapia* are clustered (BABA).

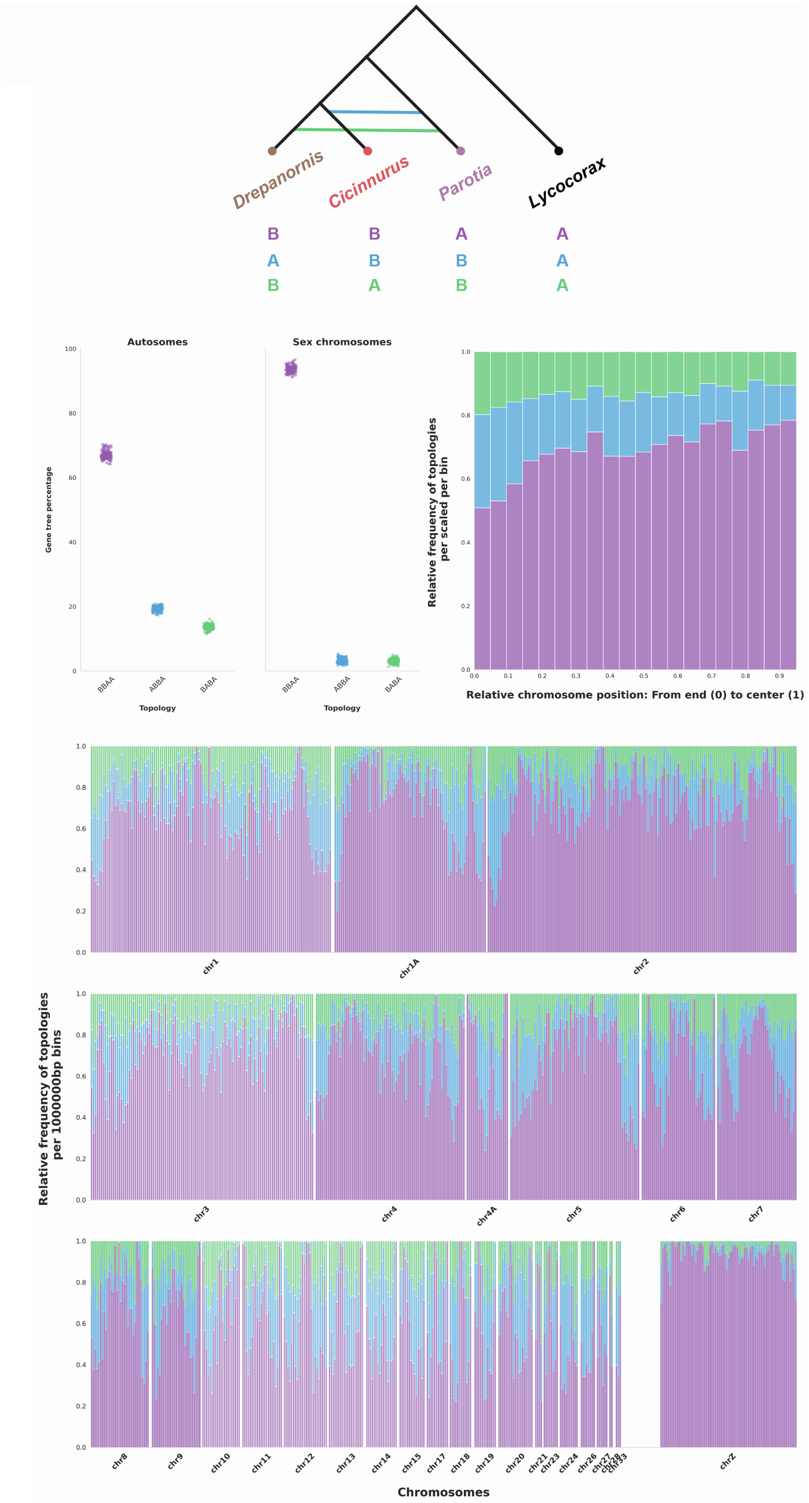

Figure S53. Frequency and genome wide distribution of window tree topologies for each possible Cartesian combination of individuals across four genera. Related to Figure 2 & S49-59. The top-left figure has the relative frequency of ABBA, BABA and BBAA topologies plotted for each of the Cartesian combinations, the top right figure has the distribution of topologies plotted by scaled chromosome position (10 macrochromosomes, summed across all Cartesian combinations) and the bottom plot illustrates the distribution of topologies in bins of 1 Mb. for all chromosomes (summed across all Cartesian combinations). Besides the species tree (BBAA), there is a small excess of trees where *Parotia* and *Cicinnurus/Diphyllodes* are clustered (ABBA), relative to trees where *Parotia* and *Drepanornis* are clustered (BABA). ABBA topologies are more frequently found towards the end of chromosomes.

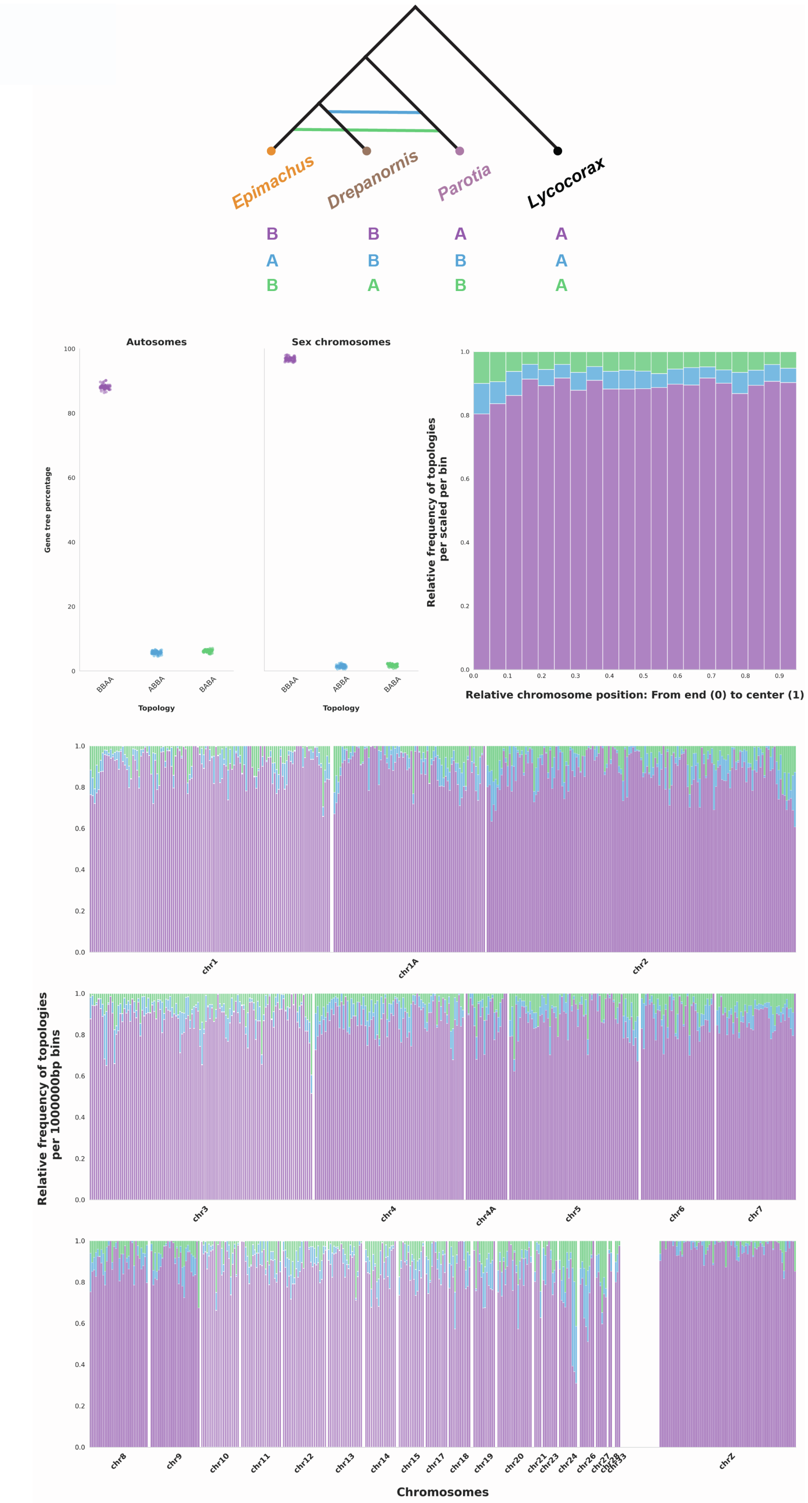

Figure S54. Frequency and genome wide distribution of window tree topologies for each possible Cartesian combination of individuals across four genera. Related to Figure 2 & S49-59. The top-left figure has the relative frequency of ABBA, BABA and BBAA topologies plotted for each of the Cartesian combinations, the top right figure has the distribution of topologies plotted by scaled chromosome position (10 macrochromosomes, summed across all Cartesian combinations) and the bottom plot illustrates the distribution of topologies in bins of 1 Mb. for all chromosomes (summed across all Cartesian combinations). Besides the species tree (BBAA), there is an equal proportion of trees where *Parotia* and *Drepanornis* are clustered (ABBA), relative to trees where *Parotia* and *Epimachus* are clustered (BABA).

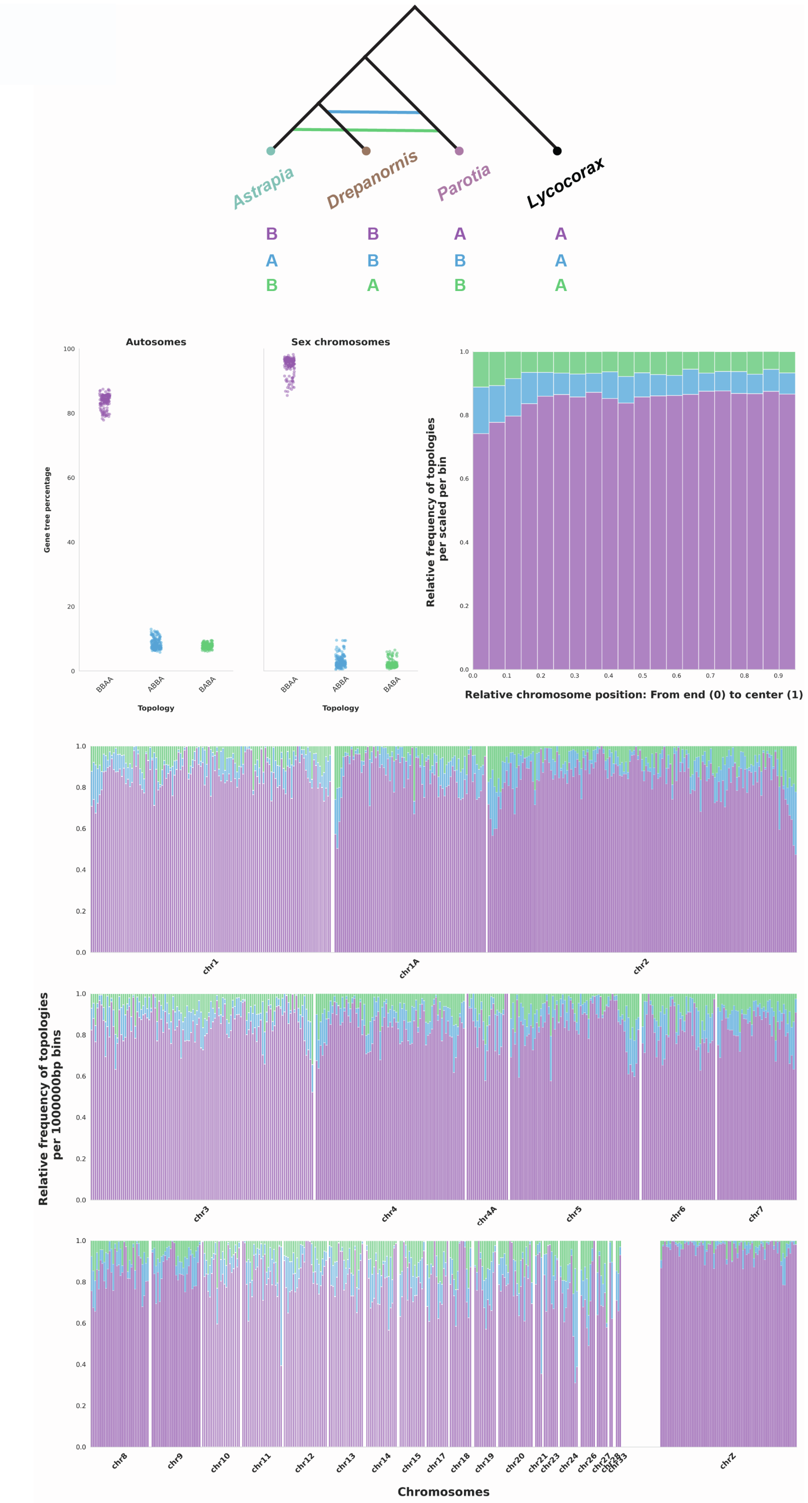

Figure S55. Frequency and genome wide distribution of window tree topologies for each possible Cartesian combination of individuals across four genera. Related to Figure 2 & S49-59. The top-left figure has the relative frequency of ABBA, BABA and BBAA topologies plotted for each of the Cartesian combinations, the top right figure has the distribution of topologies plotted by scaled chromosome position (10 macrochromosomes, summed across all Cartesian combinations) and the bottom plot illustrates the distribution of topologies in bins of 1 Mb. for all chromosomes (summed across all Cartesian combinations). Besides the species tree (BBAA), there is an equal proportion of trees where *Parotia* and *Drepanornis* are clustered (ABBA), relative to trees where *Parotia* and *Astrapia* are clustered (BABA).

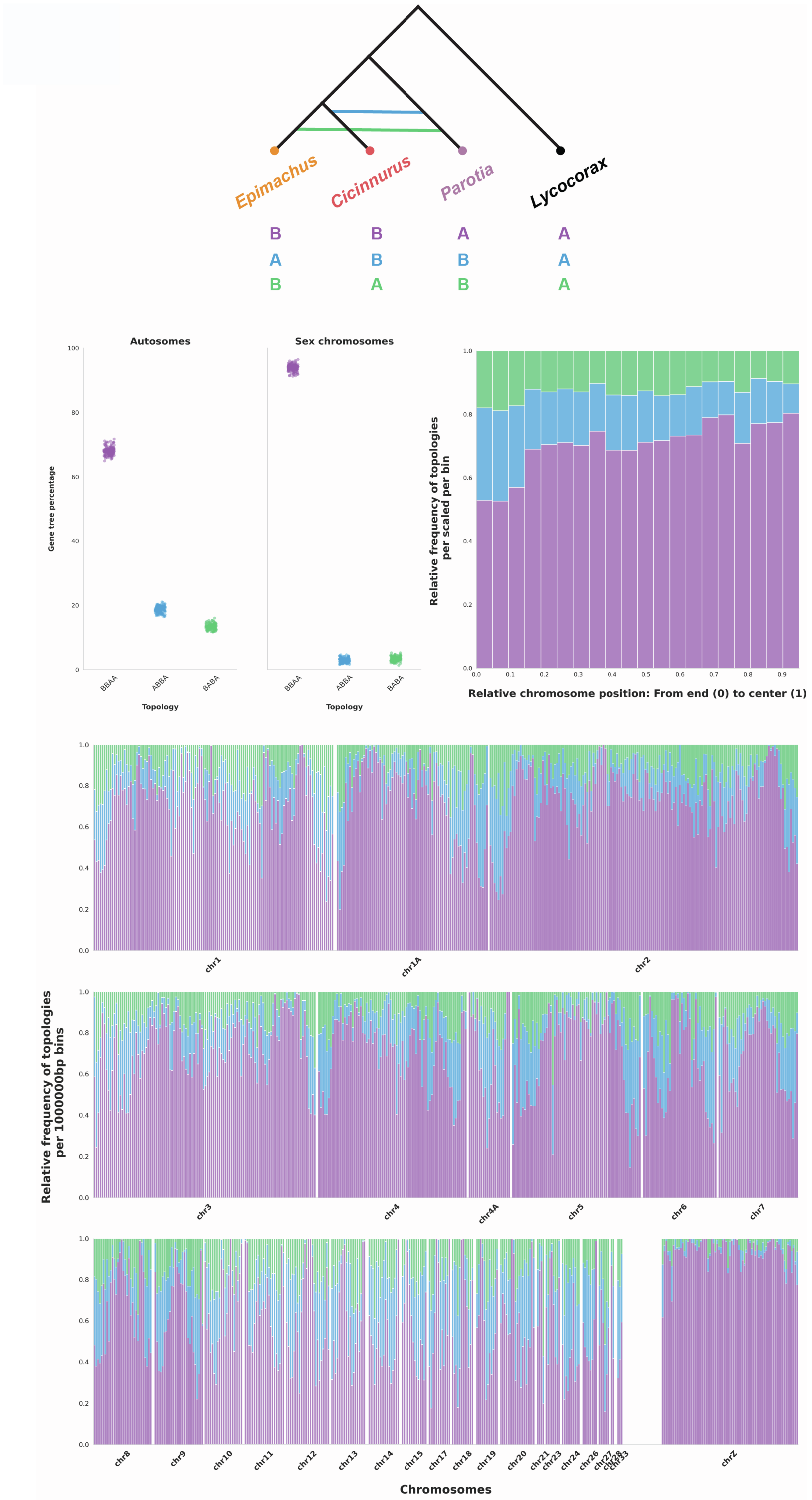

Figure S56. Frequency and genome wide distribution of window tree topologies for each possible Cartesian combination of individuals across four genera. Related to Figure 2 & S49-59. The top-left figure has the relative frequency of ABBA, BABA and BBAA topologies plotted for each of the Cartesian combinations, the top right figure has the distribution of topologies plotted by scaled chromosome position (10 macrochromosomes, summed across all Cartesian combinations) and the bottom plot illustrates the distribution of topologies in bins of 1 Mb. for all chromosomes (summed across all Cartesian combinations). Besides the species tree (BBAA), there is a small excess of trees where *Parotia* and *Cicinnurus/Diphyllodes* are clustered (ABBA), relative to trees where *Parotia* and *Epimachus* are clustered (BABA). ABBA topologies are more frequently found towards the end of chromosomes.

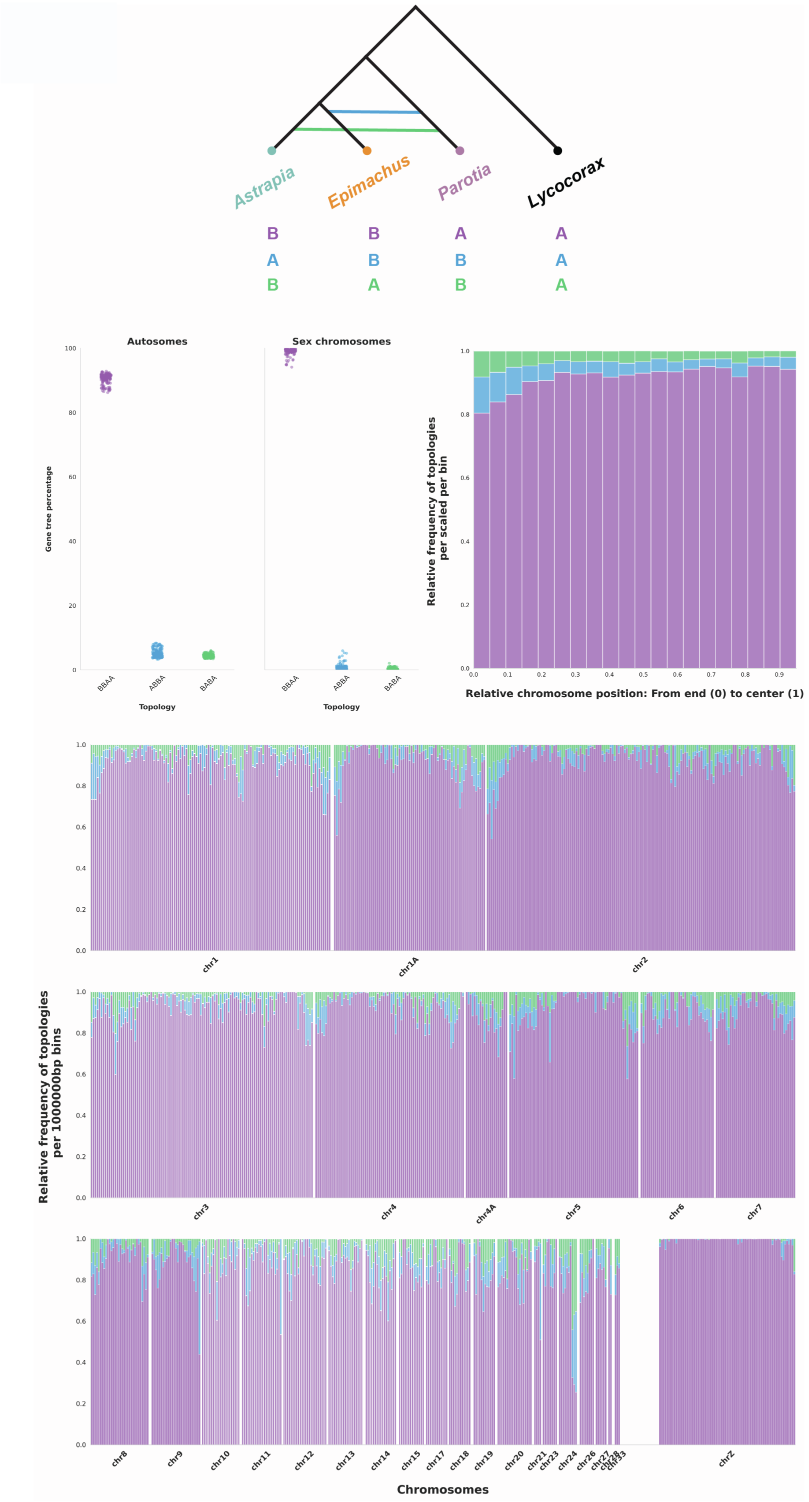

Figure S57. Frequency and genome wide distribution of window tree topologies for each possible Cartesian combination of individuals across four genera. Related to Figure 2 & S49-59. The top-left figure has the relative frequency of ABBA, BABA and BBAA topologies plotted for each of the Cartesian combinations, the top right figure has the distribution of topologies plotted by scaled chromosome position (10 macrochromosomes, summed across all Cartesian combinations) and the bottom plot illustrates the distribution of topologies in bins of 1 Mb. for all chromosomes (summed across all Cartesian combinations). Besides the species tree (BBAA), there is an equal proportion of trees where *Parotia* and *Epimachus* are clustered (ABBA), relative to trees where *Parotia* and *Astrapia* are clustered (BABA).

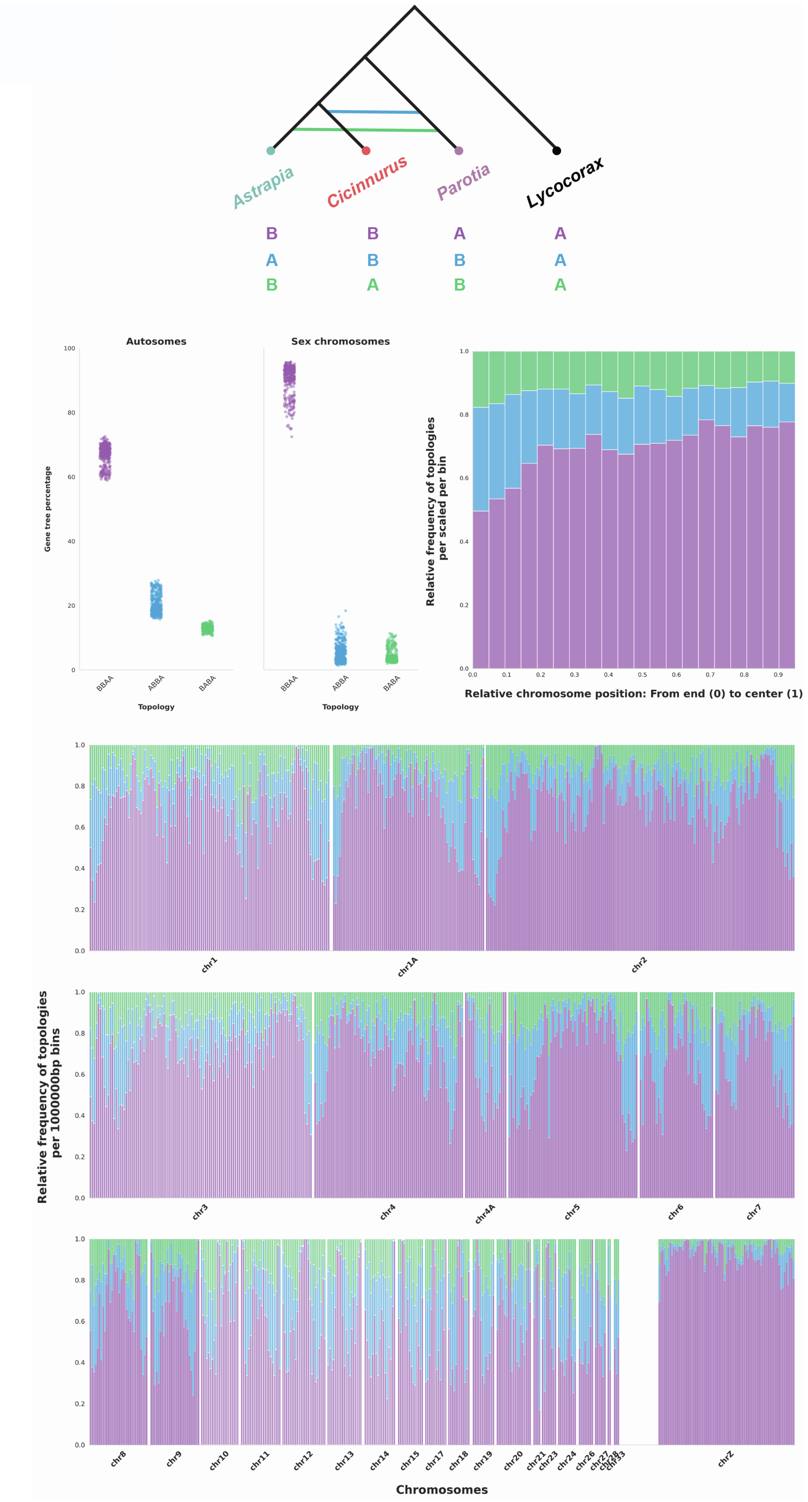

Figure S58. Frequency and genome wide distribution of window tree topologies for each possible Cartesian combination of individuals across four genera. Related to Figure 2 & S49-59. The top-left figure has the relative frequency of ABBA, BABA and BBAA topologies plotted for each of the Cartesian combinations, the top right figure has the distribution of topologies plotted by scaled chromosome position (10 macrochromosomes, summed across all Cartesian combinations) and the bottom plot illustrates the distribution of topologies in bins of 1 Mb. for all chromosomes (summed across all Cartesian combinations). Besides the species tree (BBAA), there is an excess of trees where *Parotia* and *Cicinnurus/Diphyllodes* are clustered (ABBA), relative to trees where *Parotia* and *Astrapia* are clustered (BABA). ABBA topologies are more frequently found towards the end of chromosomes.

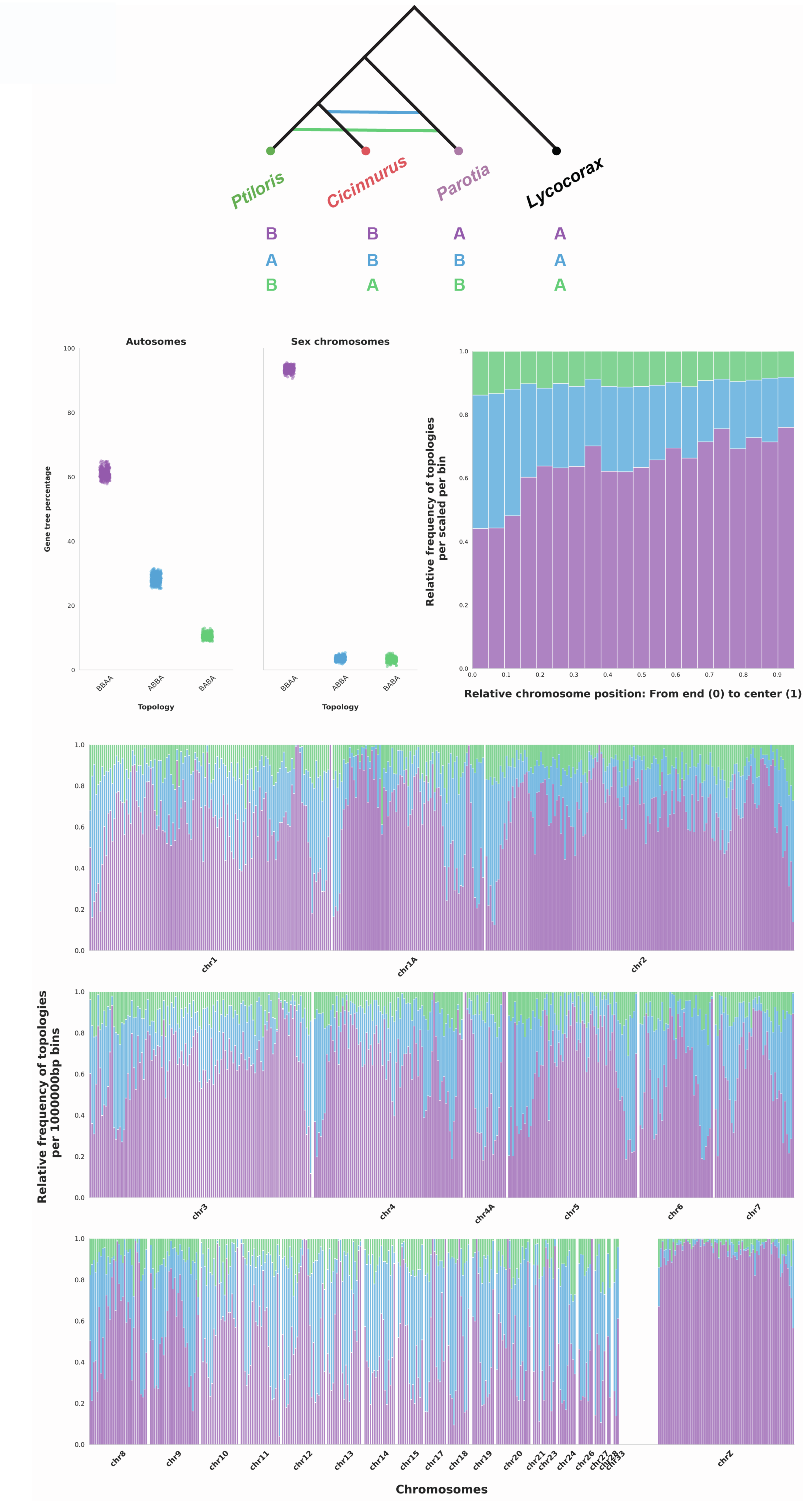

Figure S59. Frequency and genome wide distribution of window tree topologies for each possible Cartesian combination of individuals across four genera. Related to Figure 2 & S49-58. The top-left figure has the relative frequency of ABBA, BABA and BBAA topologies plotted for each of the Cartesian combinations, the top right figure has the distribution of topologies plotted by scaled chromosome position (10 macrochromosomes, summed across all Cartesian combinations) and the bottom plot illustrates the distribution of topologies in bins of 1 Mb. for all chromosomes (summed across all Cartesian combinations). Besides the species tree (BBAA), there is a large excess of trees where *Parotia* and *Cicinnurus/Diphyllodes* are clustered (ABBA), relative to trees where *Parotia* and *Ptiloris/Lophorina* are clustered (BABA). ABBA topologies are more frequently found towards the end of chromosomes.

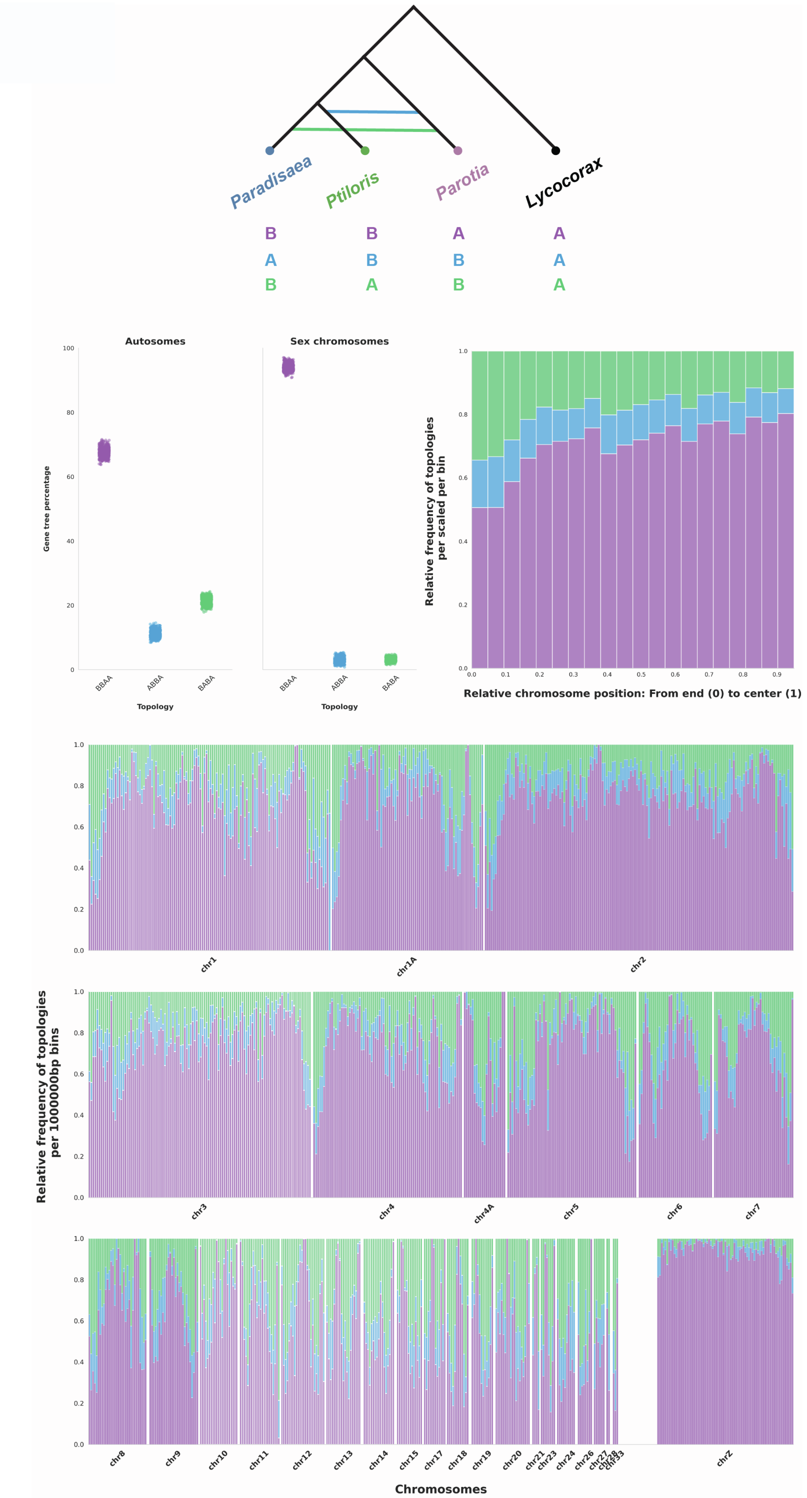

Figure S60. Frequency and genome wide distribution of window tree topologies for each possible Cartesian combination of individuals across four genera. Related to Figure 2 & S59-74. The top-left figure has the relative frequency of ABBA, BABA and BBAA topologies plotted for each of the Cartesian combinations, the top right figure has the distribution of topologies plotted by scaled chromosome position (10 macrochromosomes, summed across all Cartesian combinations) and the bottom plot illustrates the distribution of topologies in bins of 1 Mb. for all chromosomes (summed across all Cartesian combinations). Besides the species tree (BBAA), there is an excess of trees where *Parotia* and *Paradisaea* are clustered (BABA), relative to trees where *Parotia* and *Ptiloris/Lophorina* are clustered (ABBA). BABA topologies are more frequently found towards the end of chromosomes.

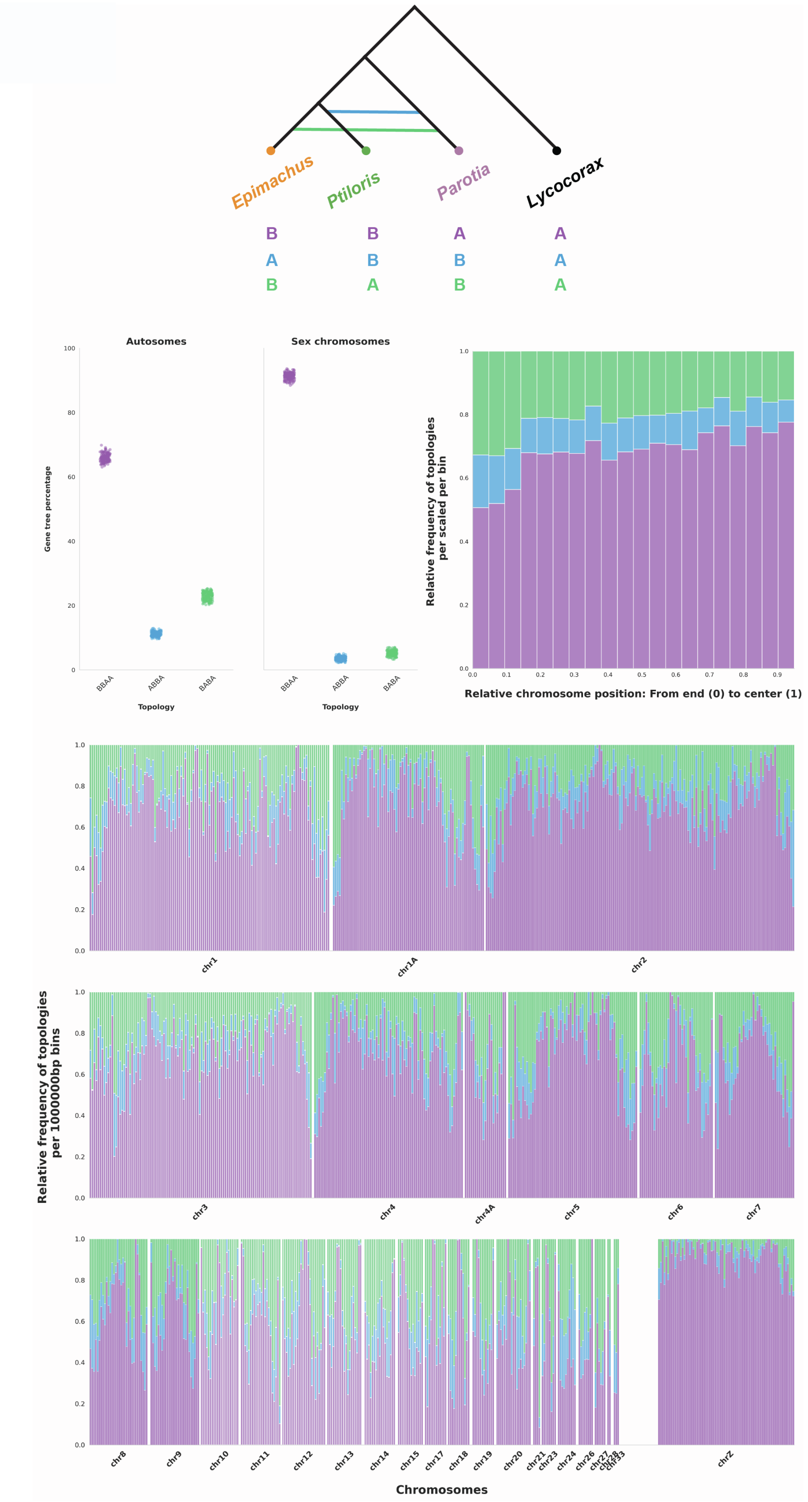

Figure S61. Frequency and genome wide distribution of window tree topologies for each possible Cartesian combination of individuals across four genera. Related to Figure 2 & S59-74. The top-left figure has the relative frequency of ABBA, BABA and BBAA topologies plotted for each of the Cartesian combinations, the top right figure has the distribution of topologies plotted by scaled chromosome position (10 macrochromosomes, summed across all Cartesian combinations) and the bottom plot illustrates the distribution of topologies in bins of 1 Mb. for all chromosomes (summed across all Cartesian combinations). Besides the species tree (BBAA), there is an excess of trees where *Parotia* and *Epimachus* are clustered (BABA), relative to trees where *Parotia* and *Ptiloris/Lophorina* are clustered (ABBA). BABA topologies are more frequently found towards the end of chromosomes.

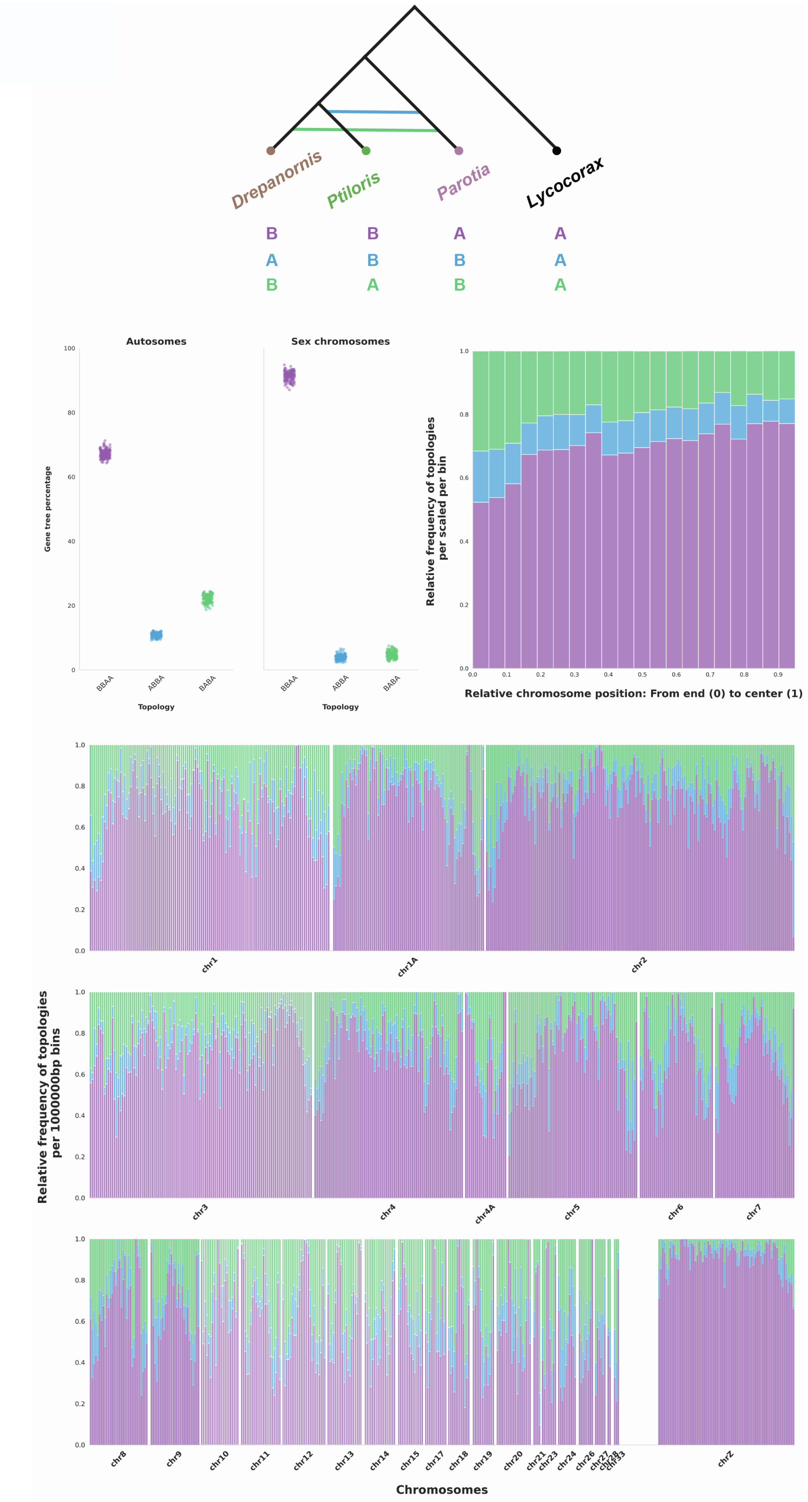

Figure S62. Frequency and genome wide distribution of window tree topologies for each possible Cartesian combination of individuals across four genera. Related to Figure 2 & S59-73. The top-left figure has the relative frequency of ABBA, BABA and BBAA topologies plotted for each of the Cartesian combinations, the top right figure has the distribution of topologies plotted by scaled chromosome position (10 macrochromosomes, summed across all Cartesian combinations) and the bottom plot illustrates the distribution of topologies in bins of 1 Mb. for all chromosomes (summed across all Cartesian combinations). Besides the species tree (BBAA), there is an excess of trees where *Parotia* and *Drepanornis* are clustered (BABA), relative to trees where *Parotia* and *Ptiloris/Lophorina* are clustered (ABBA). BABA topologies are more frequently found towards the end of chromosomes.

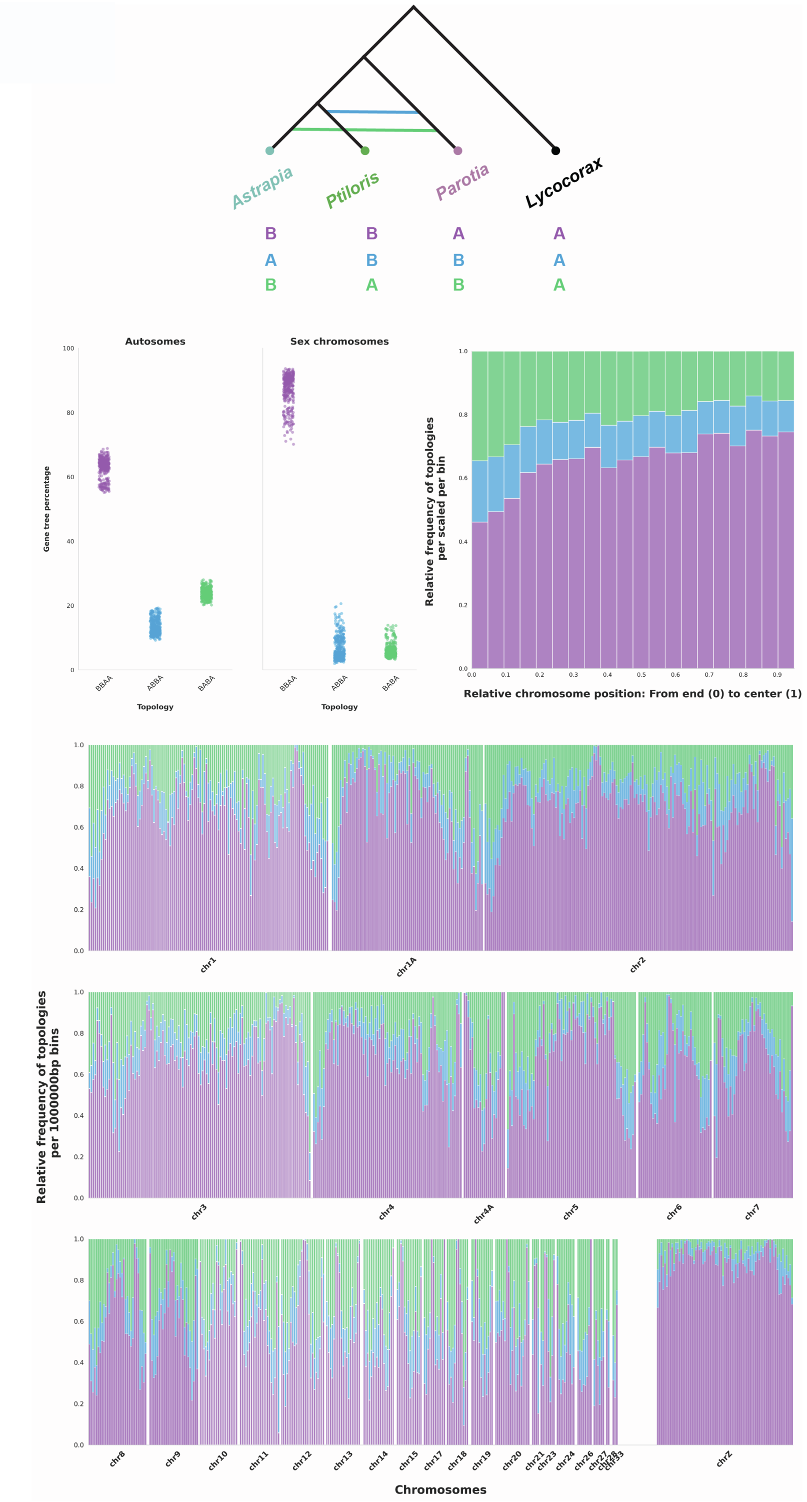

Figure S63. Frequency and genome wide distribution of window tree topologies for each possible Cartesian combination of individuals across four genera. Related to Figure 2 & S59-73. The top-left figure has the relative frequency of ABBA, BABA and BBAA topologies plotted for each of the Cartesian combinations, the top right figure has the distribution of topologies plotted by scaled chromosome position (10 macrochromosomes, summed across all Cartesian combinations) and the bottom plot illustrates the distribution of topologies in bins of 1 Mb. for all chromosomes (summed across all Cartesian combinations). Besides the species tree (BBAA), there is an excess of trees where *Parotia* and *Astrapia* are clustered (BABA), relative to trees where *Parotia* and *Ptiloris/Lophorina* are clustered (ABBA). BABA topologies are more frequently found towards the end of chromosomes.

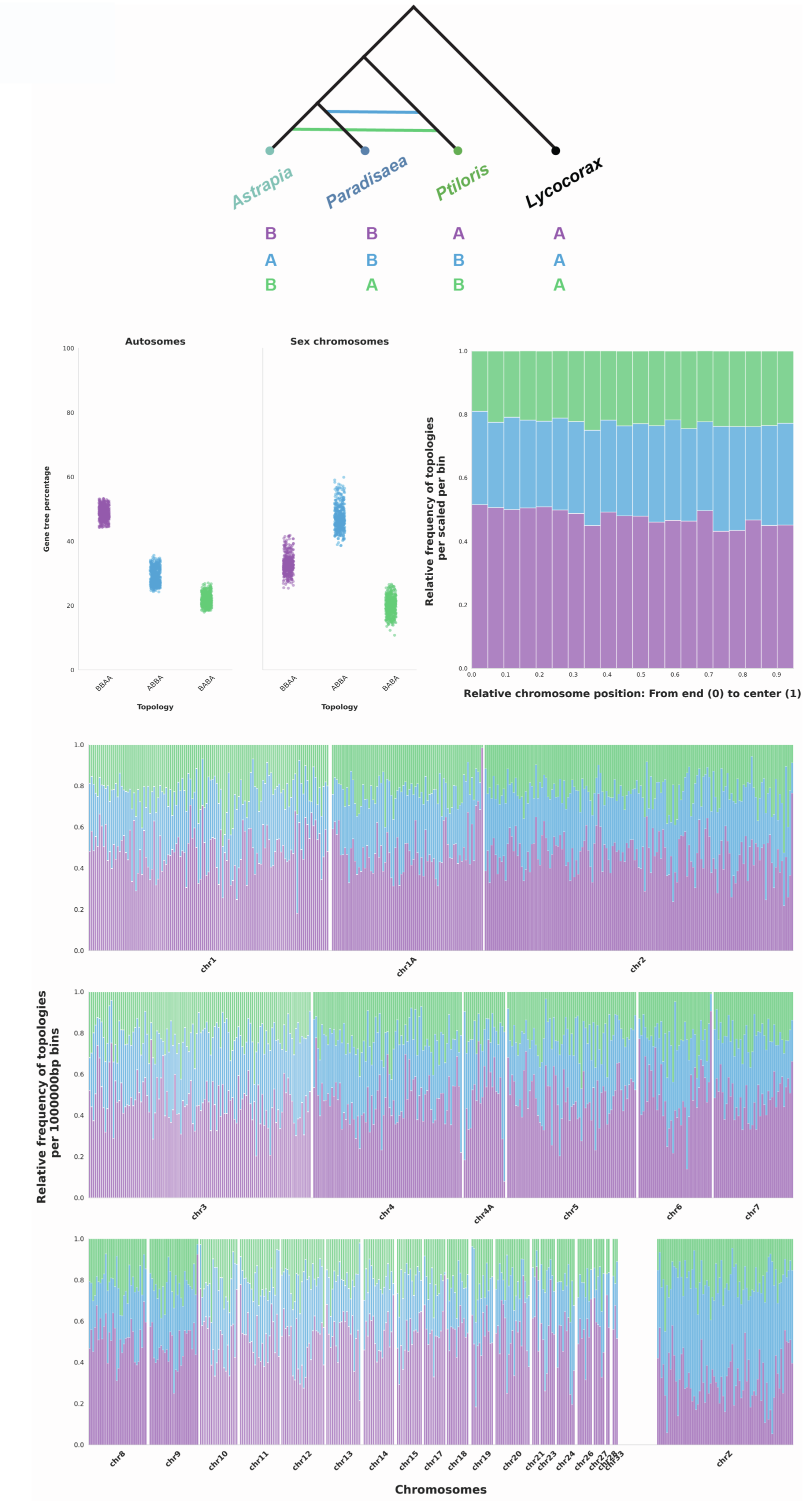

Figure S64. Frequency and genome wide distribution of window tree topologies for each possible Cartesian combination of individuals across four genera. Related to Figure 2 & S59-73. The top-left figure has the relative frequency of ABBA, BABA and BBAA topologies plotted for each of the Cartesian combinations, the top right figure has the distribution of topologies plotted by scaled chromosome position (10 macrochromosomes, summed across all Cartesian combinations) and the bottom plot illustrates the distribution of topologies in bins of 1 Mb. for all chromosomes (summed across all Cartesian combinations). There is discordance between Z and autosomes in the most frequently recovered topology. On the autosomes, *Astrapia* is more closely related to *Paradisaea* (BBAA), whereas *Ptiloris* is more closely related to *Paradisaea* on the Z chromosome (ABBA). There is no clear pattern in the genome-wide distribution of topologies.

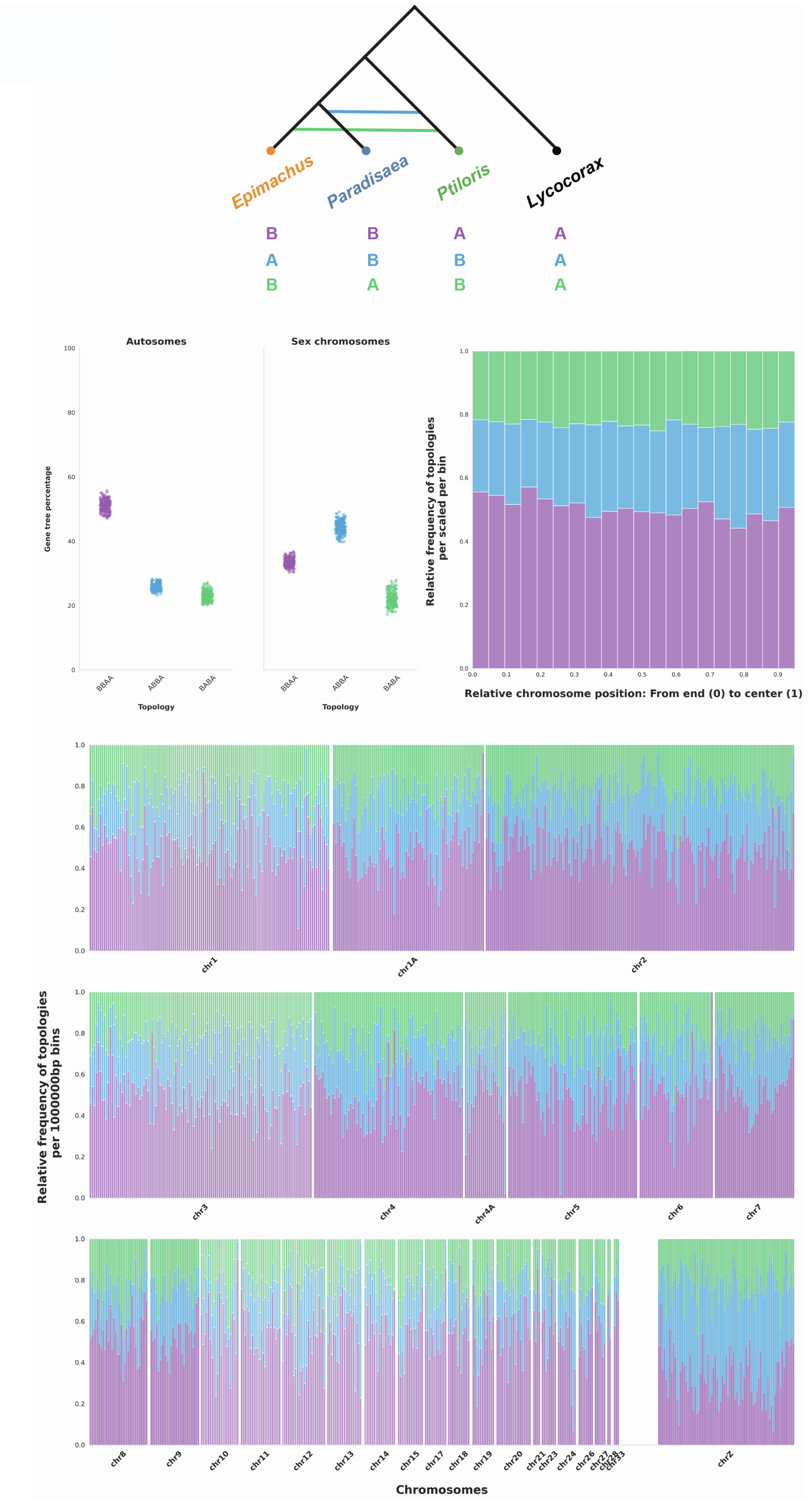

Figure S65. Frequency and genome wide distribution of window tree topologies for each possible Cartesian combination of individuals across four genera. Related to Figure 2 & S59-73. The top-left figure has the relative frequency of ABBA, BABA and BBAA topologies plotted for each of the Cartesian combinations, the top right figure has the distribution of topologies plotted by scaled chromosome position (10 macrochromosomes, summed across all Cartesian combinations) and the bottom plot illustrates the distribution of topologies in bins of 1 Mb. for all chromosomes (summed across all Cartesian combinations). There is discordance between Z and autosomes in the most frequently recovered topology. On the autosomes, *Epimachus* is more closely related to *Paradisaea* (BBAA), whereas *Ptiloris* is more closely related to *Paradisaea* on the Z chromosome (ABBA). There is no clear pattern in the genome-wide distribution of topologies.

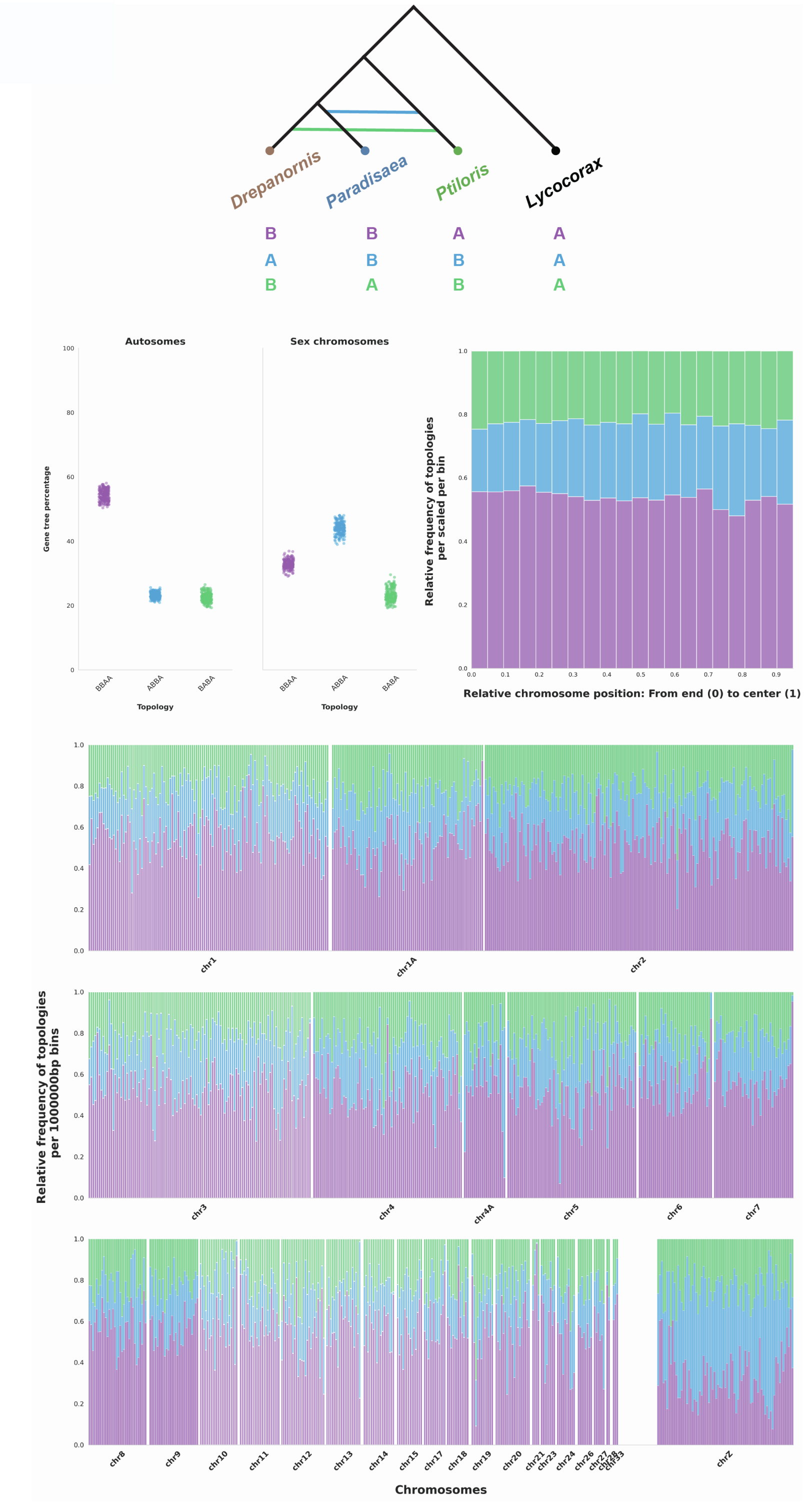

Figure S66. Frequency and genome wide distribution of window tree topologies for each possible Cartesian combination of individuals across four genera. Related to Figure 2 & S59-73. The top-left figure has the relative frequency of ABBA, BABA and BBAA topologies plotted for each of the Cartesian combinations, the top right figure has the distribution of topologies plotted by scaled chromosome position (10 macrochromosomes, summed across all Cartesian combinations) and the bottom plot illustrates the distribution of topologies in bins of 1 Mb. for all chromosomes (summed across all Cartesian combinations). There is discordance between Z and autosomes in the most frequently recovered topology. On the autosomes, *Drepanornis* is more closely related to *Paradisaea* (BBAA), whereas *Ptiloris* is more closely related to *Paradisaea* on the Z chromosome (ABBA). There is no clear pattern in the genome-wide distribution of topologies.

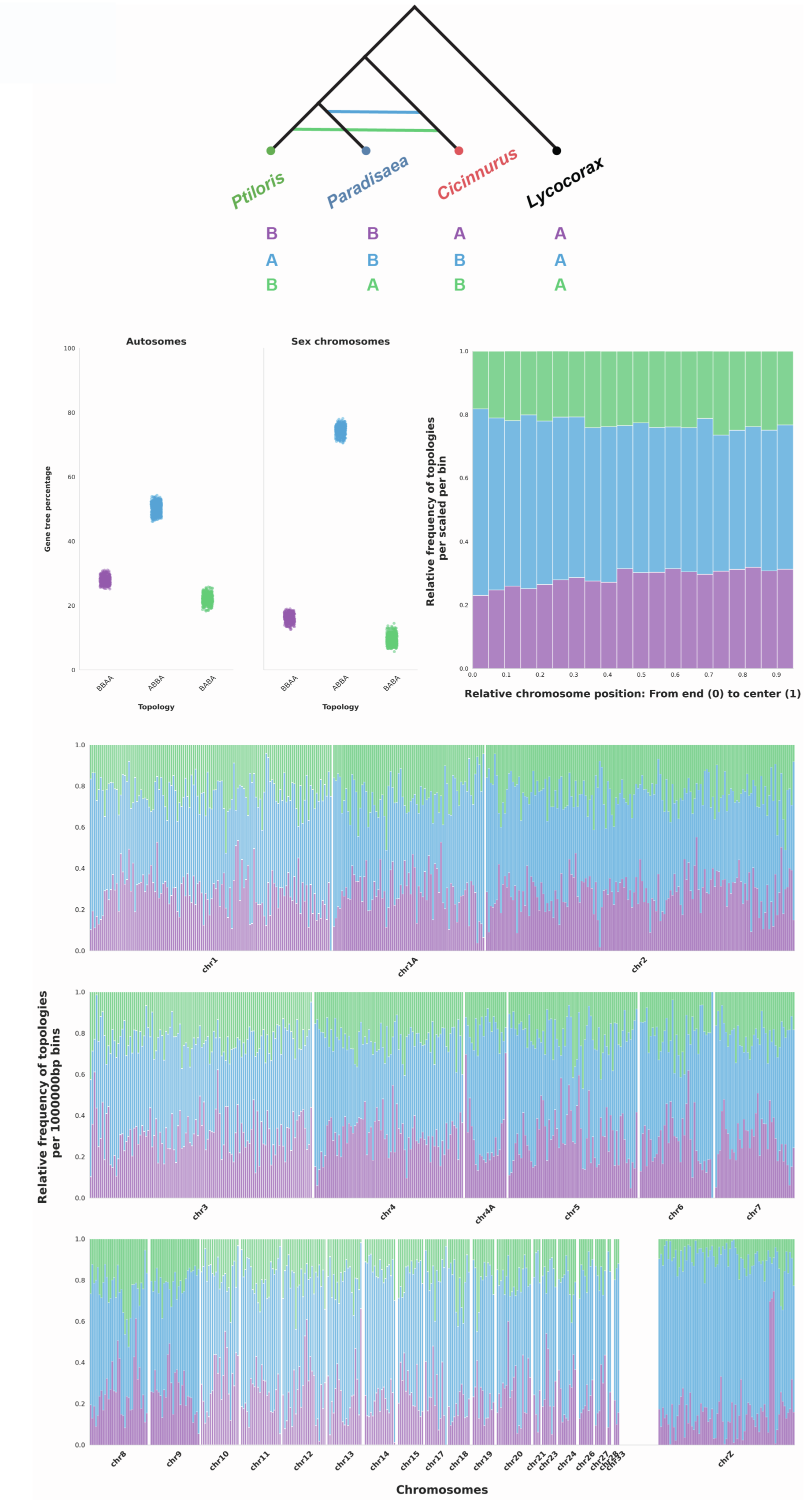

Figure S67. Frequency and genome wide distribution of window tree topologies for each possible Cartesian combination of individuals across four genera. Related to Figure 2 & S59-73. The top-left figure has the relative frequency of ABBA, BABA and BBAA topologies plotted for each of the Cartesian combinations, the top right figure has the distribution of topologies plotted by scaled chromosome position (10 macrochromosomes, summed across all Cartesian combinations) and the bottom plot illustrates the distribution of topologies in bins of 1 Mb. for all chromosomes (summed across all Cartesian combinations). Besides the species tree (ABBA), there is an excess of trees where *Ptiloris* and *Paradisaea* are clustered (BBAA), relative to trees where *Ptiloris* and *Cicinurrus/Diphyllodes* are clustered (BABA). BBAA topologies are more frequently found towards the end of chromosomes.

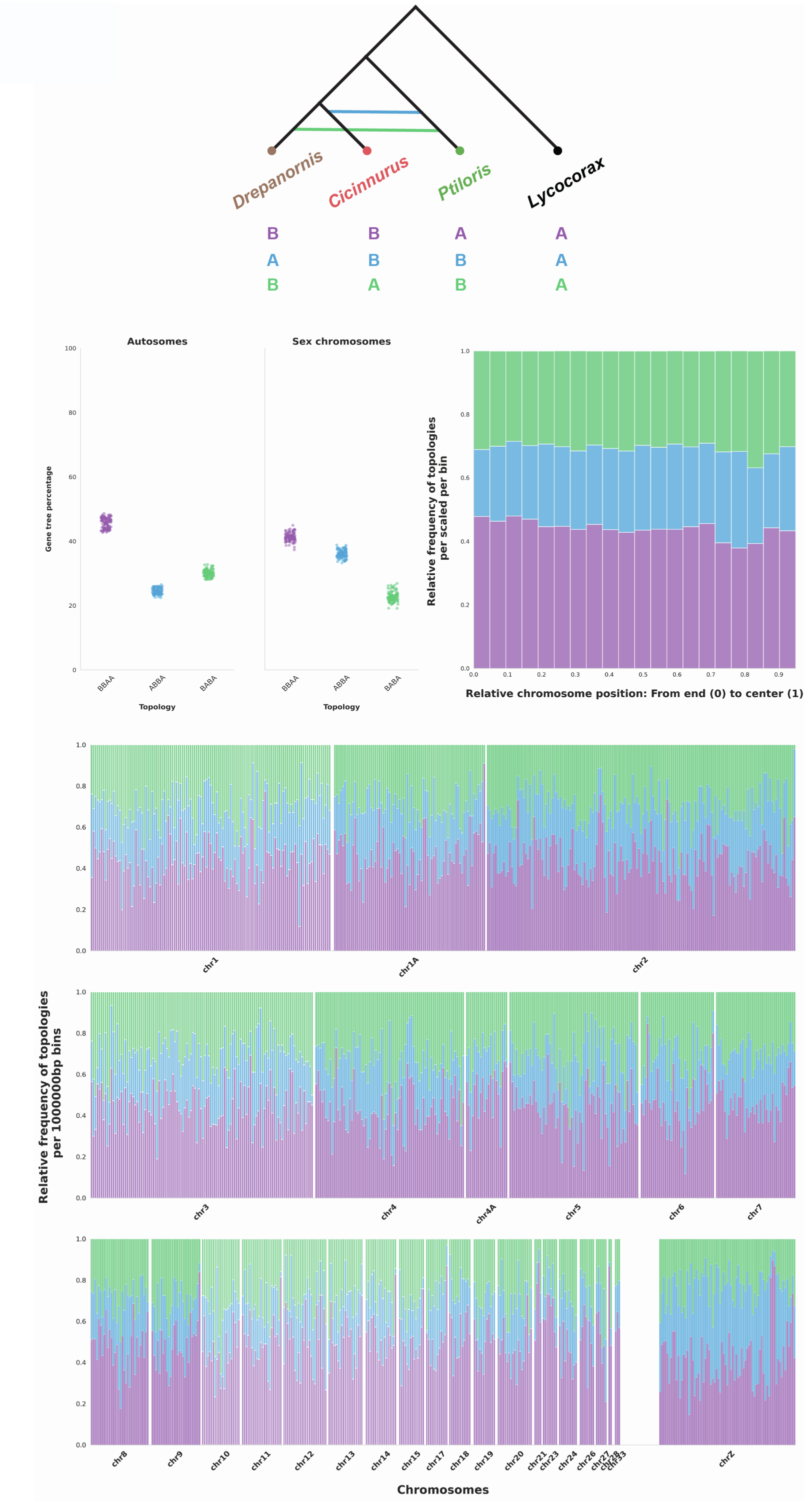

Figure S68. Frequency and genome wide distribution of wind.ow tree topologies for each possible Cartesian combination of individuals across four genera. Related to Figure 2 & S59-73 The top-left figure has the relative frequency of ABBA, BABA and BBAA topologies plotted for each of the Cartesian combinations, the top right figure has the distribution of topologies plotted by scaled chromosome position (10 macrochromosomes, summed across all Cartesian combinations) and the bottom plot illustrates the distribution of topologies in bins of 1 Mb. for all chromosomes (summed across all Cartesian combinations). Besides the species tree (BBAA), there is discordance between Z and autosomes in the alternative topology that is most frequently recovered. On the autosomes, *Ptiloris* is more often closely related to *Drepanornis* (BABA), whereas *Ptiloris* is more often closely related to *Cicinurrus/Diphyllodes* on the Z chromosome (ABBA).

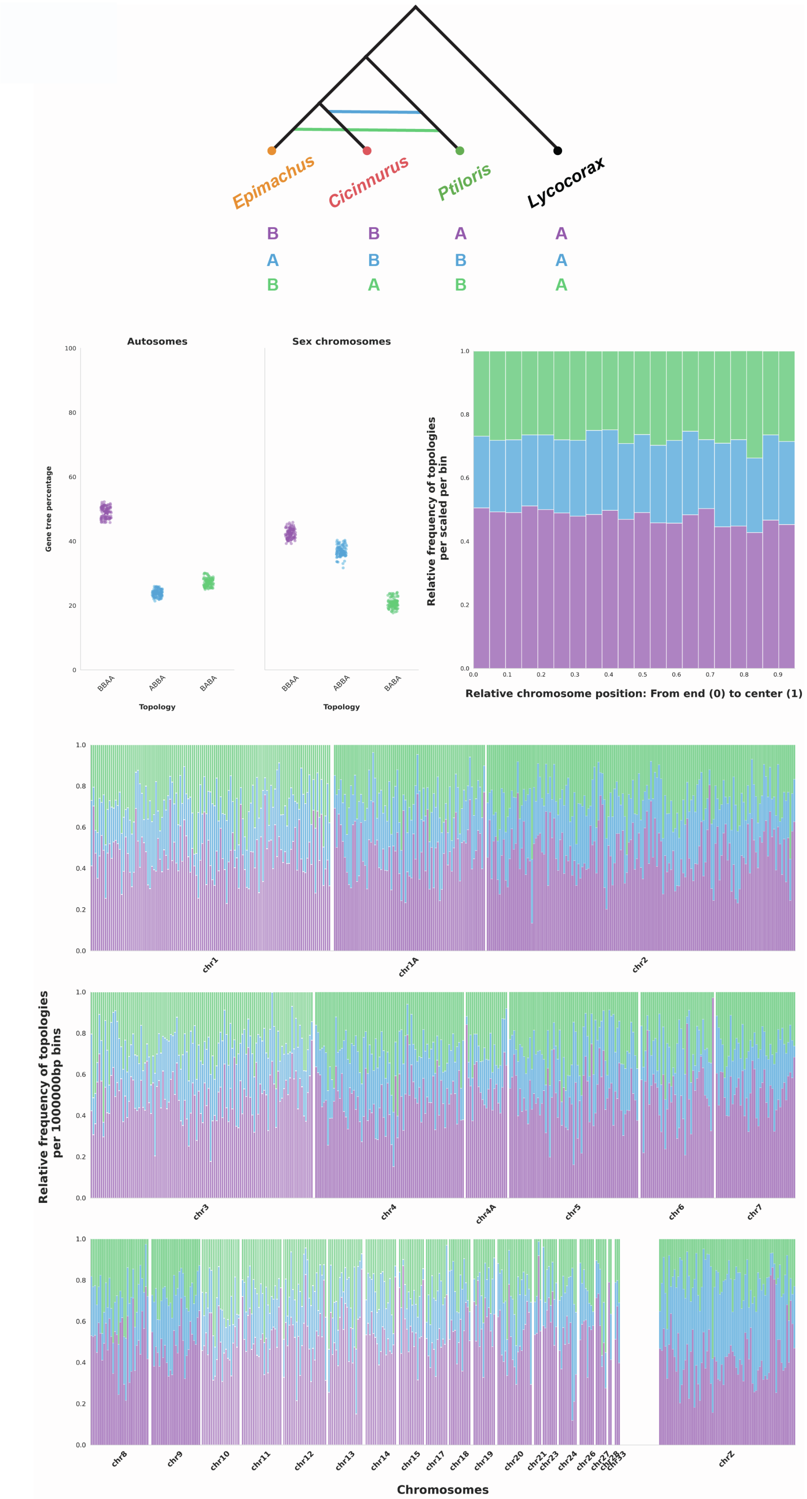

Figure S69. Frequency and genome wide distribution of window tree topologies for each possible Cartesian combination of individuals across four genera. Related to Figure 2 & S59-73. The top-left figure has the relative frequency of ABBA, BABA and BBAA topologies plotted for each of the Cartesian combinations, the top right figure has the distribution of topologies plotted by scaled chromosome position (10 macrochromosomes, summed across all Cartesian combinations) and the bottom plot illustrates the distribution of topologies in bins of 1 Mb. for all chromosomes (summed across all Cartesian combinations). Besides the species tree (BBAA), there is discordance between Z and autosomes in the alternative topology that is most frequently recovered. On the autosomes, *Ptiloris* is more often closely related to *Epimachus* (BABA), whereas *Ptiloris* is more often closely related to *Cicinurrus/Diphyllodes* on the Z chromosome (ABBA).

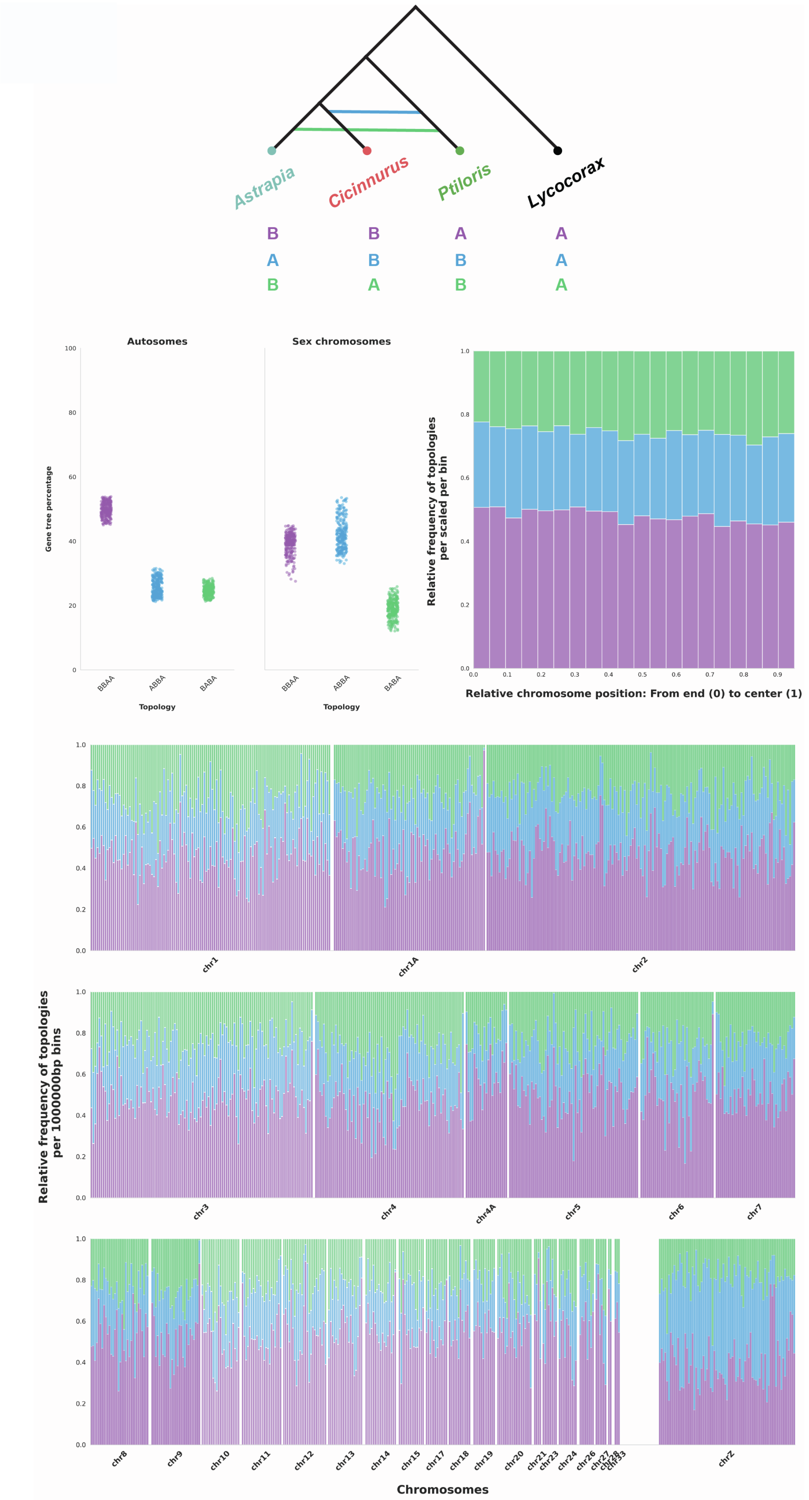

Figure S70. Frequency and genome wide distribution of window tree topologies for each possible Cartesian combination of individuals across four genera. Related to Figure 2 & S59-73. The top-left figure has the relative frequency of ABBA, BABA and BBAA topologies plotted for each of the Cartesian combinations, the top right figure has the distribution of topologies plotted by scaled chromosome position (10 macrochromosomes, summed across all Cartesian combinations) and the bottom plot illustrates the distribution of topologies in bins of 1 Mb. for all chromosomes (summed across all Cartesian combinations). There is discordance between Z and autosomes in the most frequently recovered topology. On the autosomes, *Cicinurrus/Diphyllodes* is more closely related to *Astrapia* (BBAA), *Cicinurrus/Diphyllodes* is more closely related to *Ptiloris* on the Z chromosome (ABBA). There is no clear pattern in the genome-wide distribution of topologies.

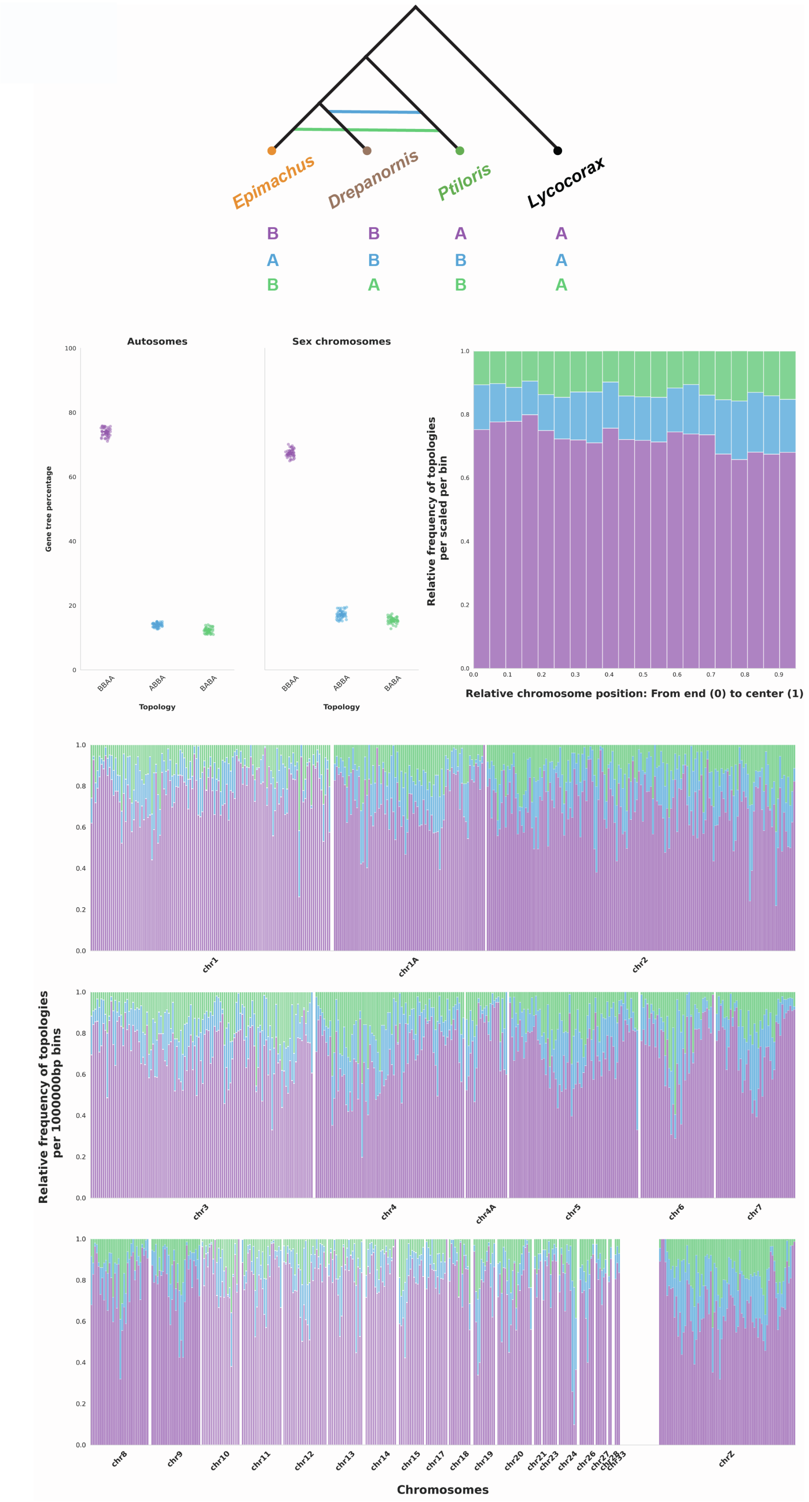

Figure S71. Frequency and genome wide distribution of window tree topologies for each possible Cartesian combination of individuals across four genera. Related to Figure 2 & S59-73. The top-left figure has the relative frequency of ABBA, BABA and BBAA topologies plotted for each of the Cartesian combinations, the top right figure has the distribution of topologies plotted by scaled chromosome position (10 macrochromosomes, summed across all Cartesian combinations) and the bottom plot illustrates the distribution of topologies in bins of 1 Mb. for all chromosomes (summed across all Cartesian combinations). Besides the species tree (BBAA), there is an equal proportion of trees where *Ptiloris* and *Epimachus* are clustered (BABA), relative to trees where *Ptiloris* and *Drepanornis* are clustered (ABBA).

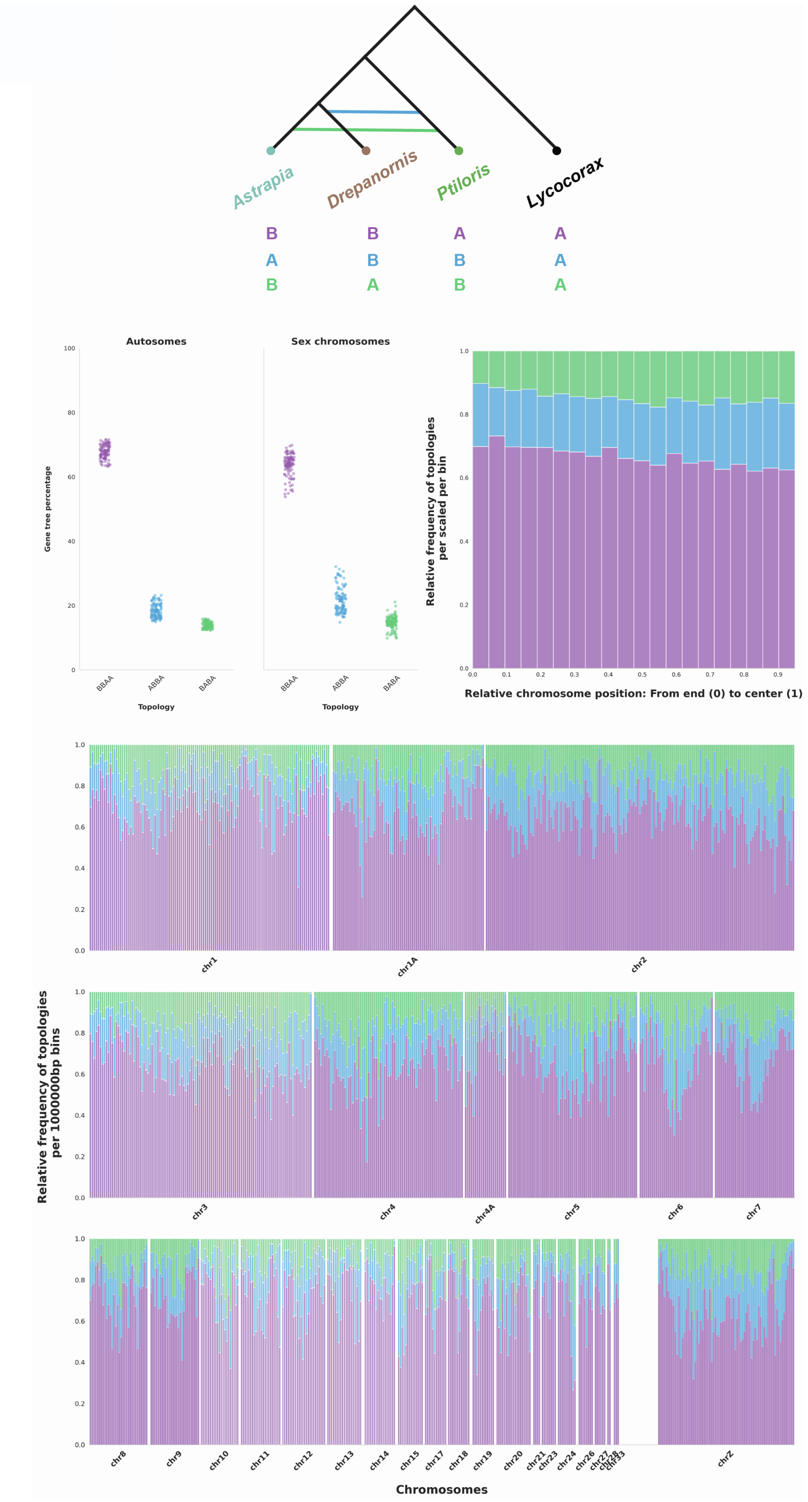

Figure S72. Frequency and genome wide distribution of window tree topologies for each possible Cartesian combination of individuals across four genera. Related to Figure 2 & S59-73. The top-left figure has the relative frequency of ABBA, BABA and BBAA topologies plotted for each of the Cartesian combinations, the top right figure has the distribution of topologies plotted by scaled chromosome position (10 macrochromosomes, summed across all Cartesian combinations) and the bottom plot illustrates the distribution of topologies in bins of 1 Mb. for all chromosomes (summed across all Cartesian combinations). Besides the species tree (BBAA), there is an minute excess of trees where *Ptiloris* and *Drepanornis* are clustered (ABBA), relative to trees where *Astrapia* and *Drepanornis* are clustered (BABA).

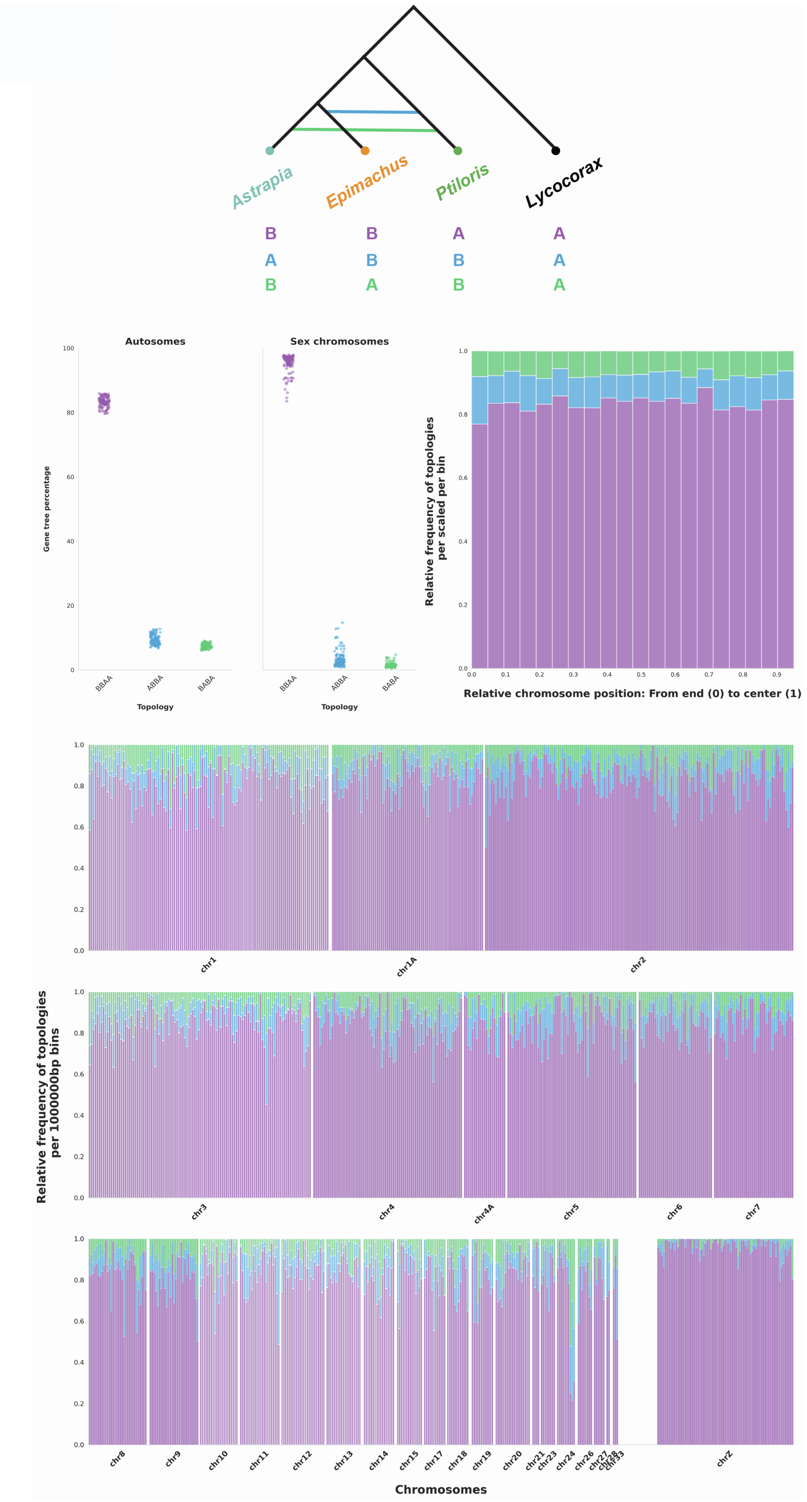

Figure S73. Frequency and genome wide distribution of window tree topologies for each possible Cartesian combination of individuals across four genera. Related to Figure 2 & S59-72. The top-left figure has the relative frequency of ABBA, BABA and BBAA topologies plotted for each of the Cartesian combinations, the top right figure has the distribution of topologies plotted by scaled chromosome position (10 macrochromosomes, summed across all Cartesian combinations) and the bottom plot illustrates the distribution of topologies in bins of 1 Mb. for all chromosomes (summed across all Cartesian combinations). Besides the species tree (BBAA), there is an minute excess of trees where *Ptiloris* and *Epimachus* are clustered (ABBA), relative to trees where *Ptiloris* and *Astrapia* are clustered (BABA).

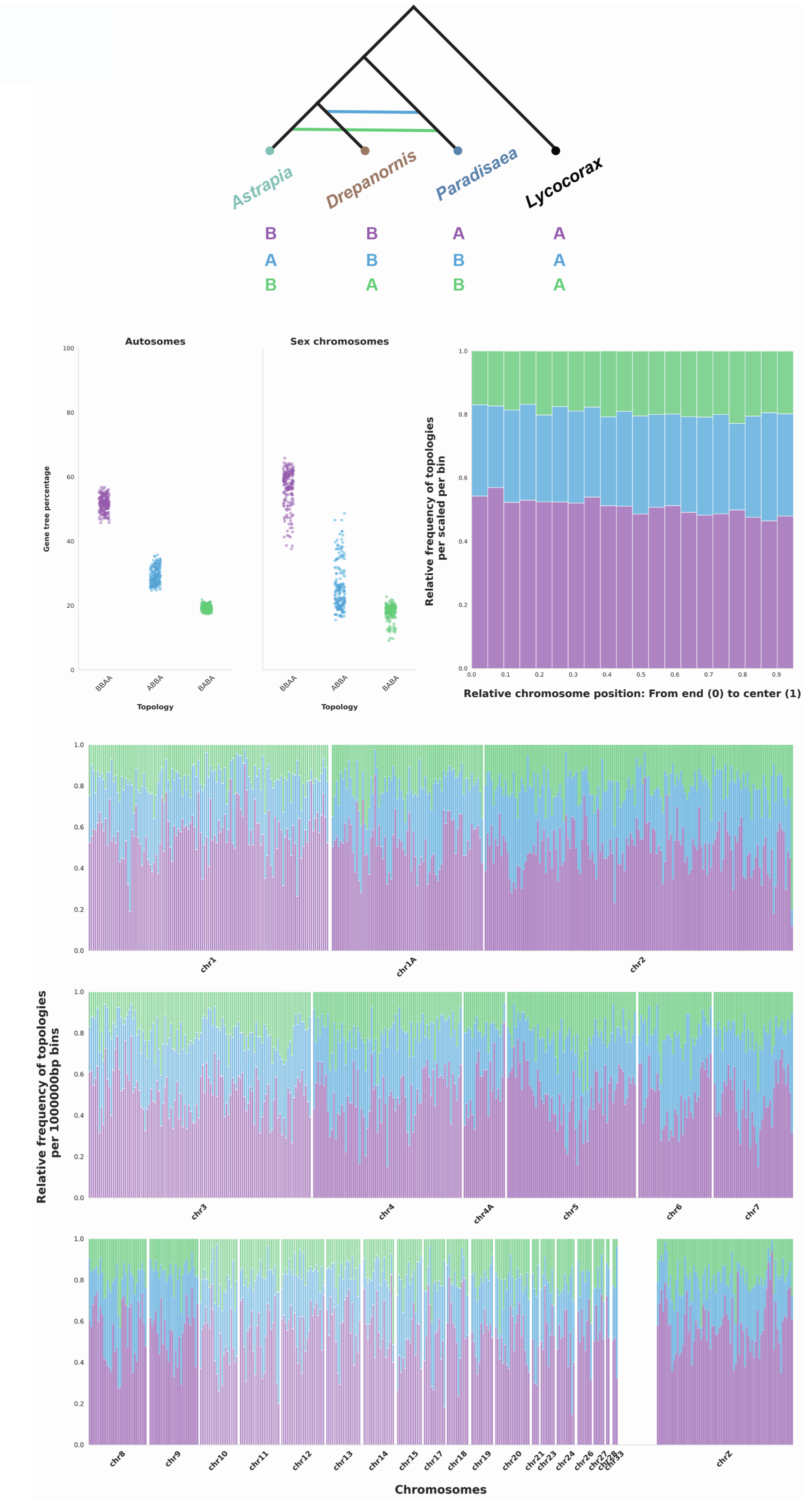

Figure S74. Frequency and genome wide distribution of window tree topologies for each possible Cartesian combination of individuals across four genera. Related to Figure 2 & S75-82. The top-left figure has the relative frequency of ABBA, BABA and BBAA topologies plotted for each of the Cartesian combinations, the top right figure has the distribution of topologies plotted by scaled chromosome position (10 macrochromosomes, summed across all Cartesian combinations) and the bottom plot illustrates the distribution of topologies in bins of 1 Mb. for all chromosomes (summed across all Cartesian combinations). Besides the species tree (BBAA), there is an excess of trees where *Paradisaea* and *Drepanornis* are clustered (ABBA), relative to trees where *Paradisaea* and *Astrapia* are clustered (BABA).

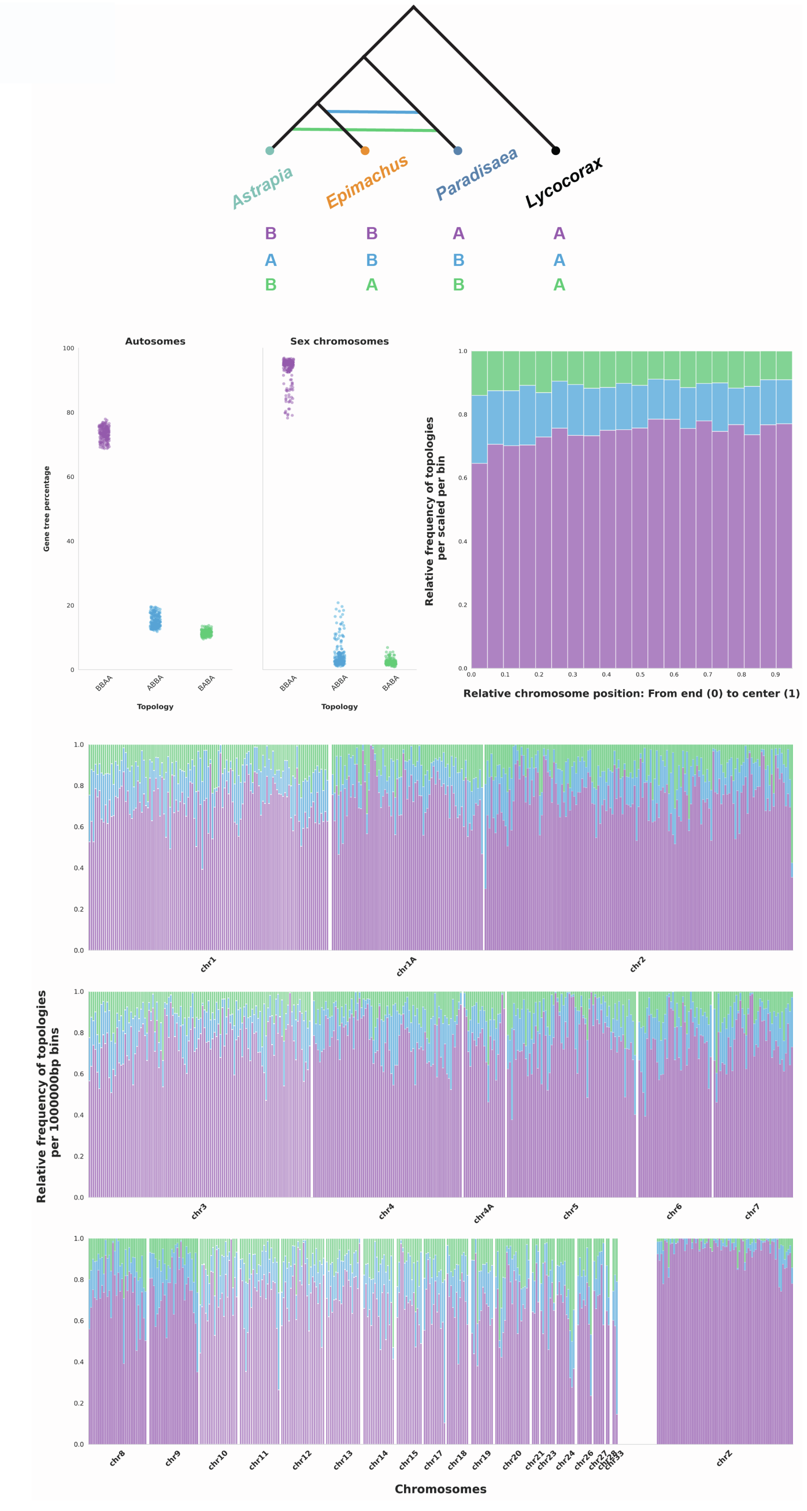

Figure S75. Frequency and genome wide distribution of window tree topologies for each possible Cartesian combination of individuals across four genera. Related to Figure 2 & S74-82. The top-left figure has the relative frequency of ABBA, BABA and BBAA topologies plotted for each of the Cartesian combinations, the top right figure has the distribution of topologies plotted by scaled chromosome position (10 macrochromosomes, summed across all Cartesian combinations) and the bottom plot illustrates the distribution of topologies in bins of 1 Mb. for all chromosomes (summed across all Cartesian combinations). Besides the species tree (BBAA), there is a minute excess of trees where *Paradisaea* and *Epimachus* are clustered (ABBA), relative to trees where *Paradisaea* and *Astrapia* are clustered (BABA).

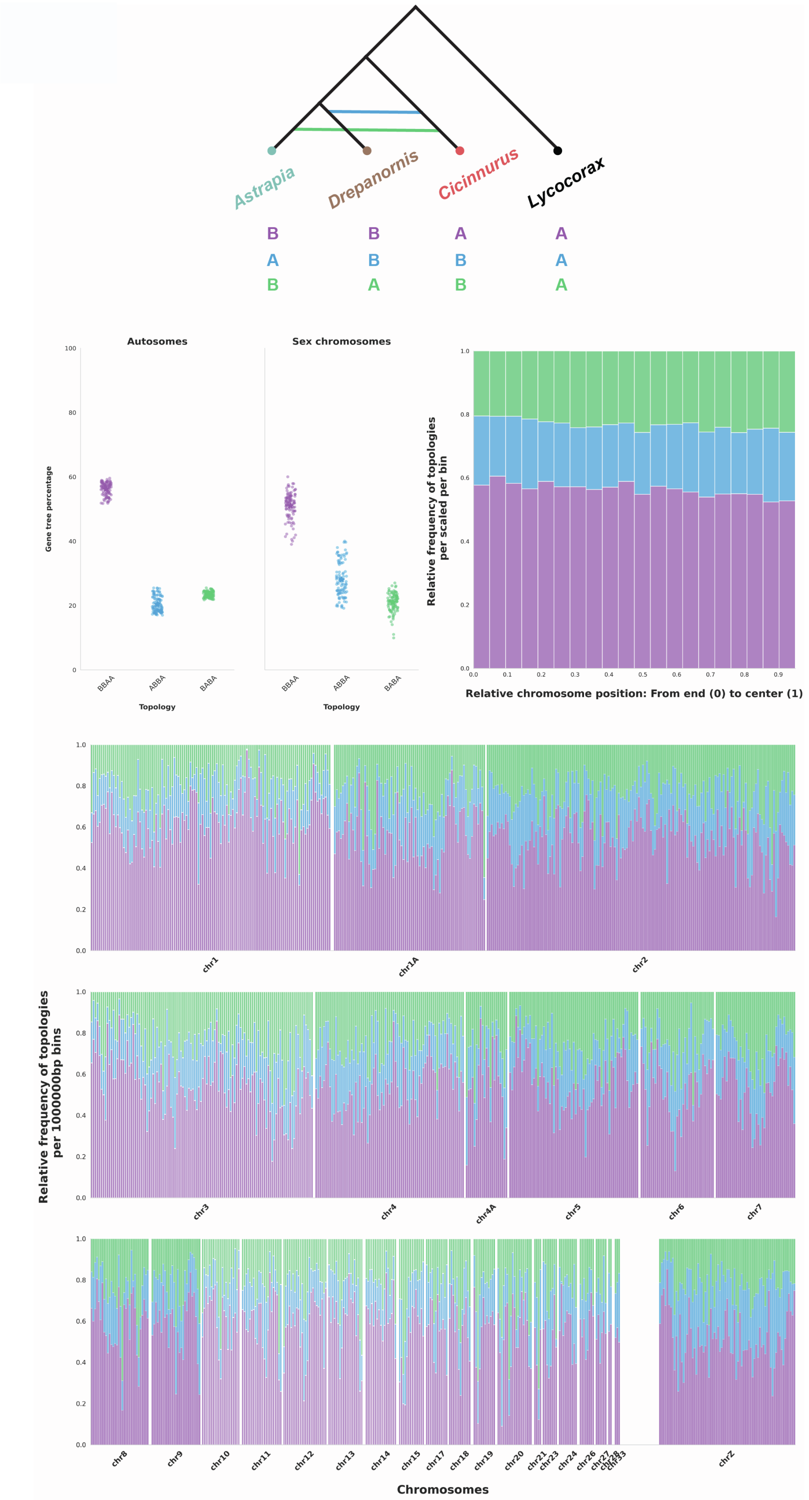

Figure S76. Frequency and genome wide distribution of window tree topologies for each possible Cartesian combination of individuals across four genera. Related to Figure 2 & S74-82. The top-left figure has the relative frequency of ABBA, BABA and BBAA topologies plotted for each of the Cartesian combinations, the top right figure has the distribution of topologies plotted by scaled chromosome position (10 macrochromosomes, summed across all Cartesian combinations) and the bottom plot illustrates the distribution of topologies in bins of 1 Mb. for all chromosomes (summed across all Cartesian combinations). Besides the species tree (BBAA), there is discordance between the Z chromosome and autosomes. ABBA and BABA topologies are equally frequent recovered on the autosomes, whereas there is a higher proportion of trees where *Cicinnurus/Diphyllodes* and *Drepanornis* are clustered on the Z chrosome.

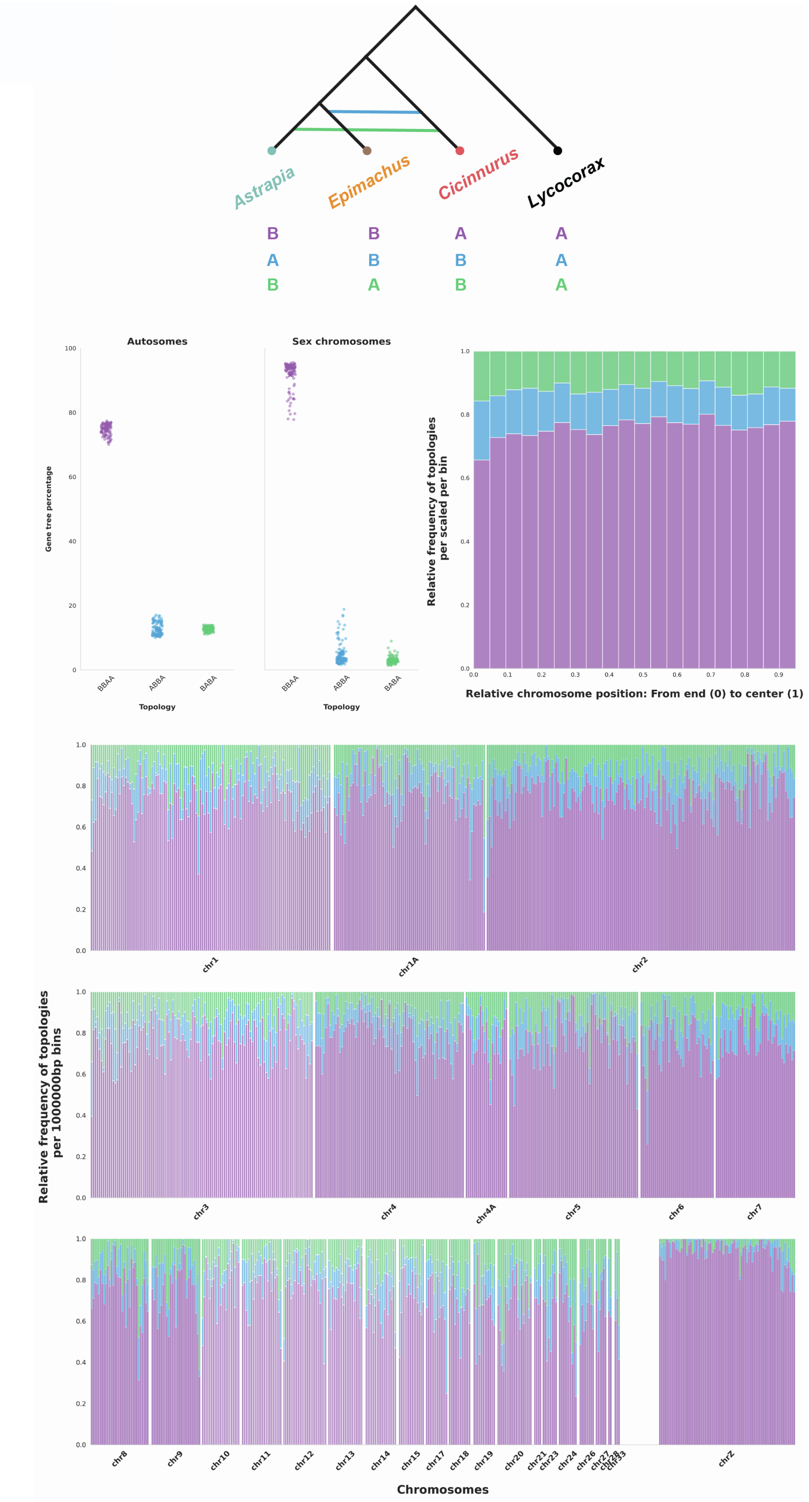

Figure S77. Frequency and genome wide distribution of window tree topologies for each possible Cartesian combination of individuals across four genera. Related to Figure 2 & S74-82. The top-left figure has the relative frequency of ABBA, BABA and BBAA topologies plotted for each of the Cartesian combinations, the top right figure has the distribution of topologies plotted by scaled chromosome position (10 macrochromosomes, summed across all Cartesian combinations) and the bottom plot illustrates the distribution of topologies in bins of 1 Mb. for all chromosomes (summed across all Cartesian combinations). Besides the species tree (BBAA), there is an equal proportion of trees where *Cicinnurus/Diphyllodes* and *Epimachus* are clustered (ABBA), relative to trees where *Cicinnurus/Diphyllodes* and *Astrapia* are clustered (BABA).

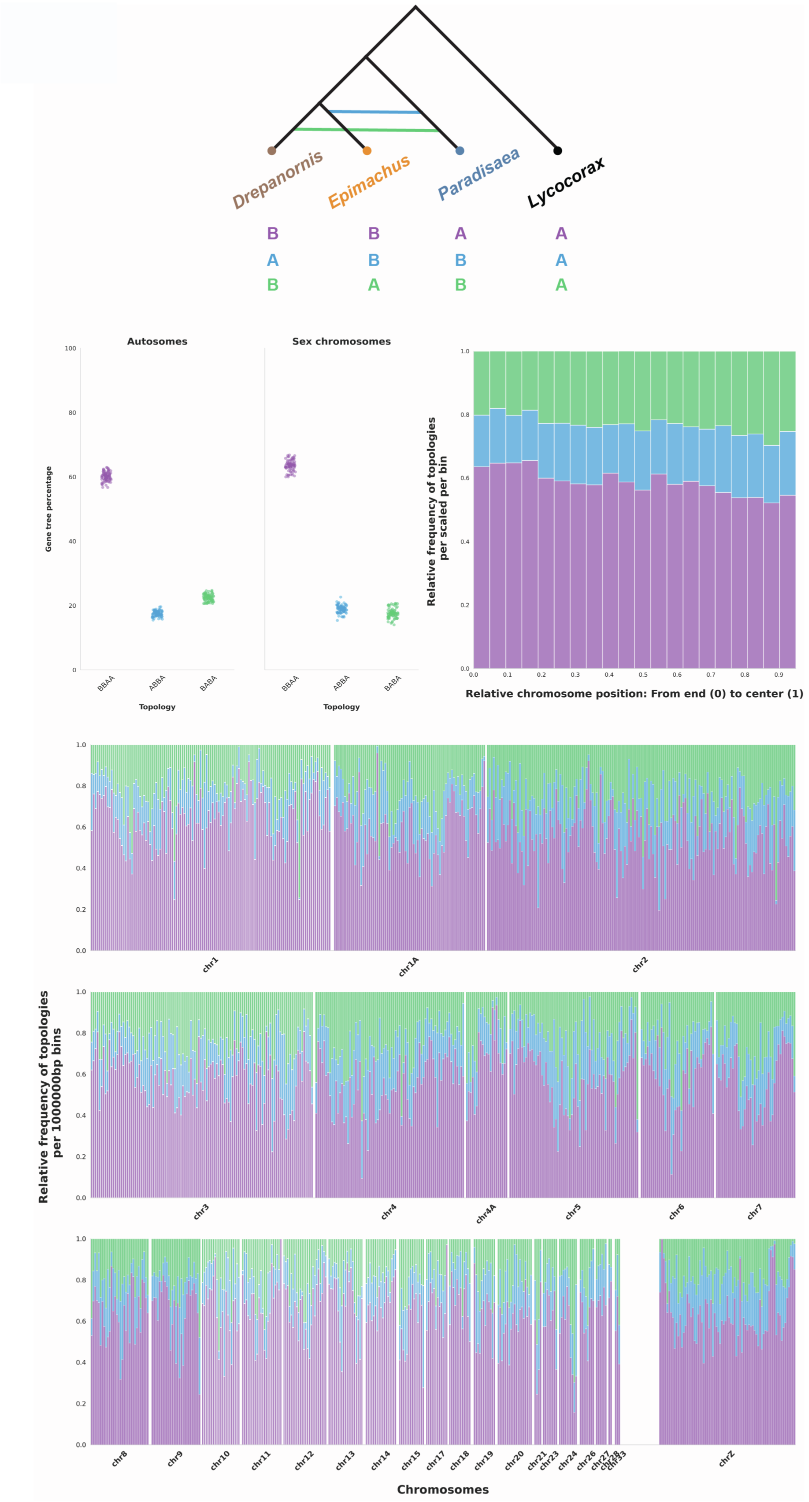

Figure S78. Frequency and genome wide distribution of window tree topologies for each possible Cartesian combination of individuals across four genera. Related to Figure 2 & S74-82. The top-left figure has the relative frequency of ABBA, BABA and BBAA topologies plotted for each of the Cartesian combinations, the top right figure has the distribution of topologies plotted by scaled chromosome position (10 macrochromosomes, summed across all Cartesian combinations) and the bottom plot illustrates the distribution of topologies in bins of 1 Mb. for all chromosomes (summed across all Cartesian combinations). Besides the species tree (BBAA), there is a minute excess of trees where *Paradisaea* and *Drepanornis* are clustered (BABA), relative to trees where *Paradisaea* and *Epimachus* are clustered (ABBA).

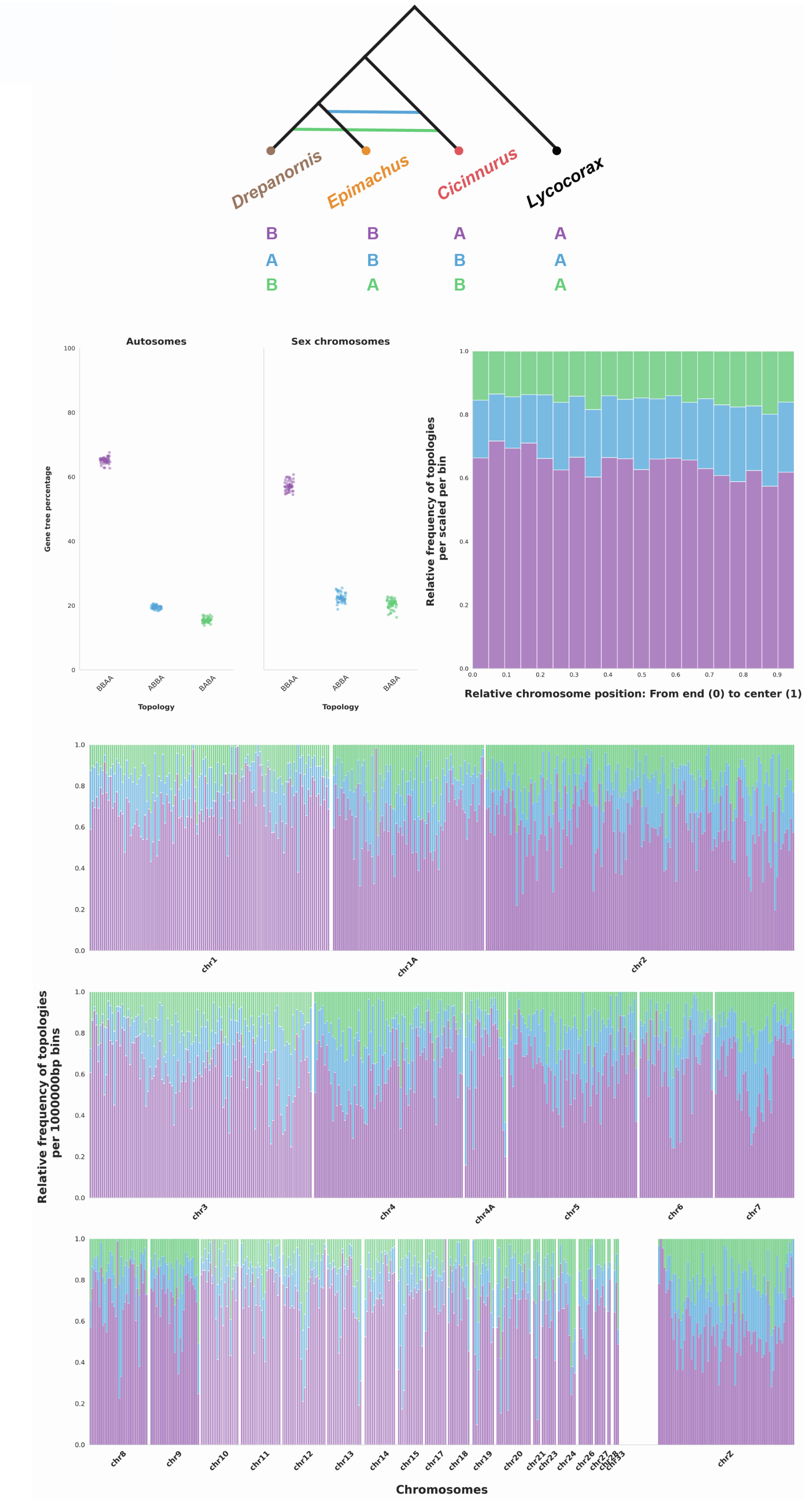

Figure S79. Frequency and genome wide distribution of window tree topologies for each possible Cartesian combination of individuals across four genera. Related to Figure 2 & S74-82. The top-left figure has the relative frequency of ABBA, BABA and BBAA topologies plotted for each of the Cartesian combinations, the top right figure has the distribution of topologies plotted by scaled chromosome position (10 macrochromosomes, summed across all Cartesian combinations) and the bottom plot illustrates the distribution of topologies in bins of 1 Mb. for all chromosomes (summed across all Cartesian combinations). Besides the species tree (BBAA), there is an almost equal proportion of trees where *Cicinnurus/Diphyllodes* and *Epimachus* are clustered (ABBA), relative to trees where *Cicinnurus/Diphyllodes* and *Drepanornis* are clustered (BABA).

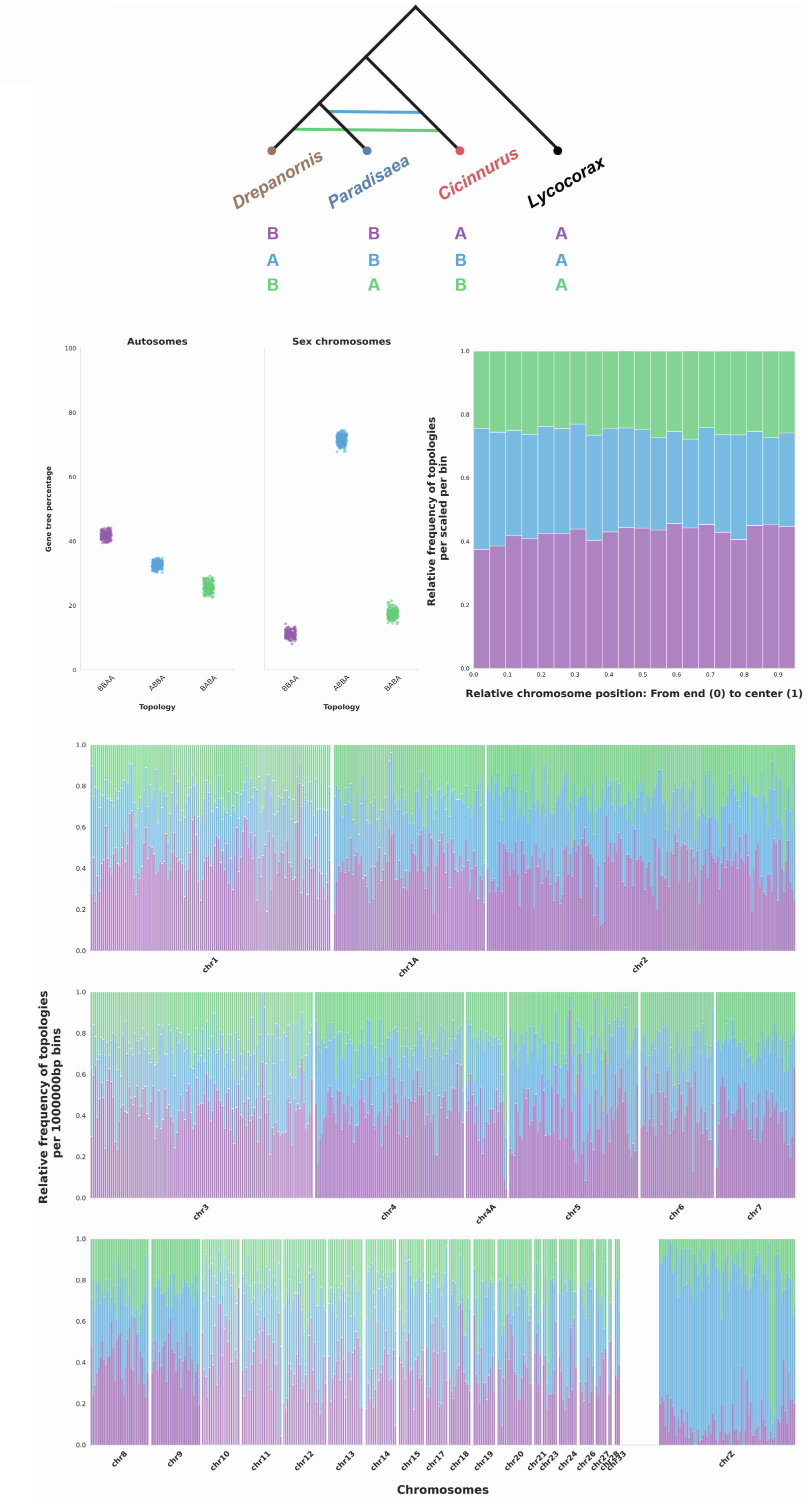

Figure S80. Frequency and genome wide distribution of window tree topologies for each possible Cartesian combination of individuals across four genera. Related to Figure 2 & S74-82. The top-left figure has the relative frequency of ABBA, BABA and BBAA topologies plotted for each of the Cartesian combinations, the top right figure has the distribution of topologies plotted by scaled chromosome position (10 macrochromosomes, summed across all Cartesian combinations) and the bottom plot illustrates the distribution of topologies in bins of 1 Mb. for all chromosomes (summed across all Cartesian combinations). There is discordance between Z and autosomes in the most frequently recovered topology. On the autosomes, *Paradisaea* is more closely related to *Drepanornis* (BBAA), whereas *Paradisaea* is more closely related to *Cicinnurus/Diphyllodes* on the Z chromosome (ABBA).

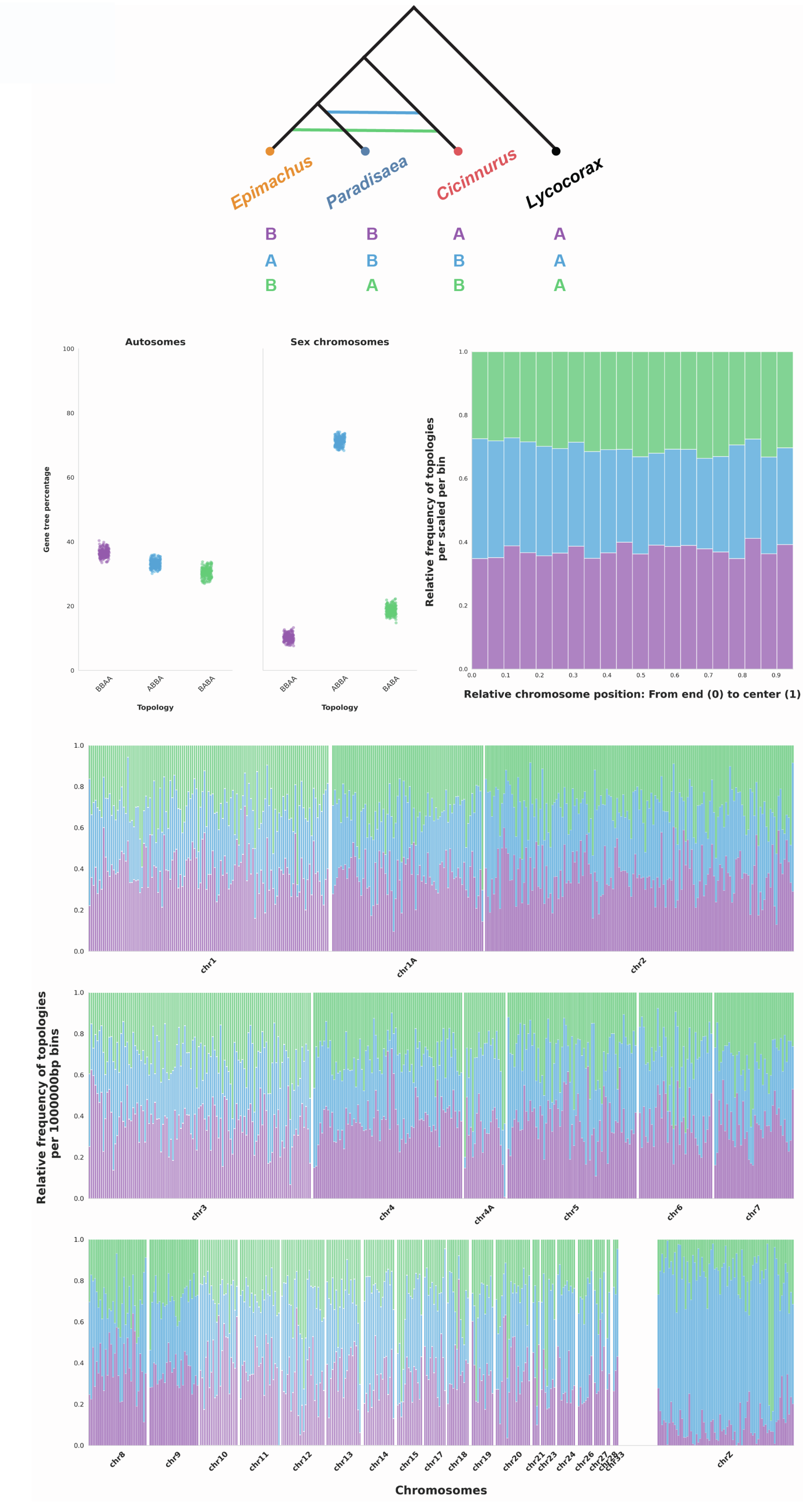

Figure S81. Frequency and genome wide distribution of window tree topologies for each possible Cartesian combination of individuals across four genera. Related to Figure 2 & S74-82. The top-left figure has the relative frequency of ABBA, BABA and BBAA topologies plotted for each of the Cartesian combinations, the top right figure has the distribution of topologies plotted by scaled chromosome position (10 macrochromosomes, summed across all Cartesian combinations) and the bottom plot illustrates the distribution of topologies in bins of 1 Mb. for all chromosomes (summed across all Cartesian combinations). There is discordance between Z and autosomes in the most frequently recovered topology. On the autosomes, *Paradisaea* is more closely related to *Epimachus* (BBAA), whereas *Paradisaea* is more closely related to *Cicinnurus/Diphyllodes* on the Z chromosome (ABBA). However, there is almost equal support for the three different topologies on the autosomes.

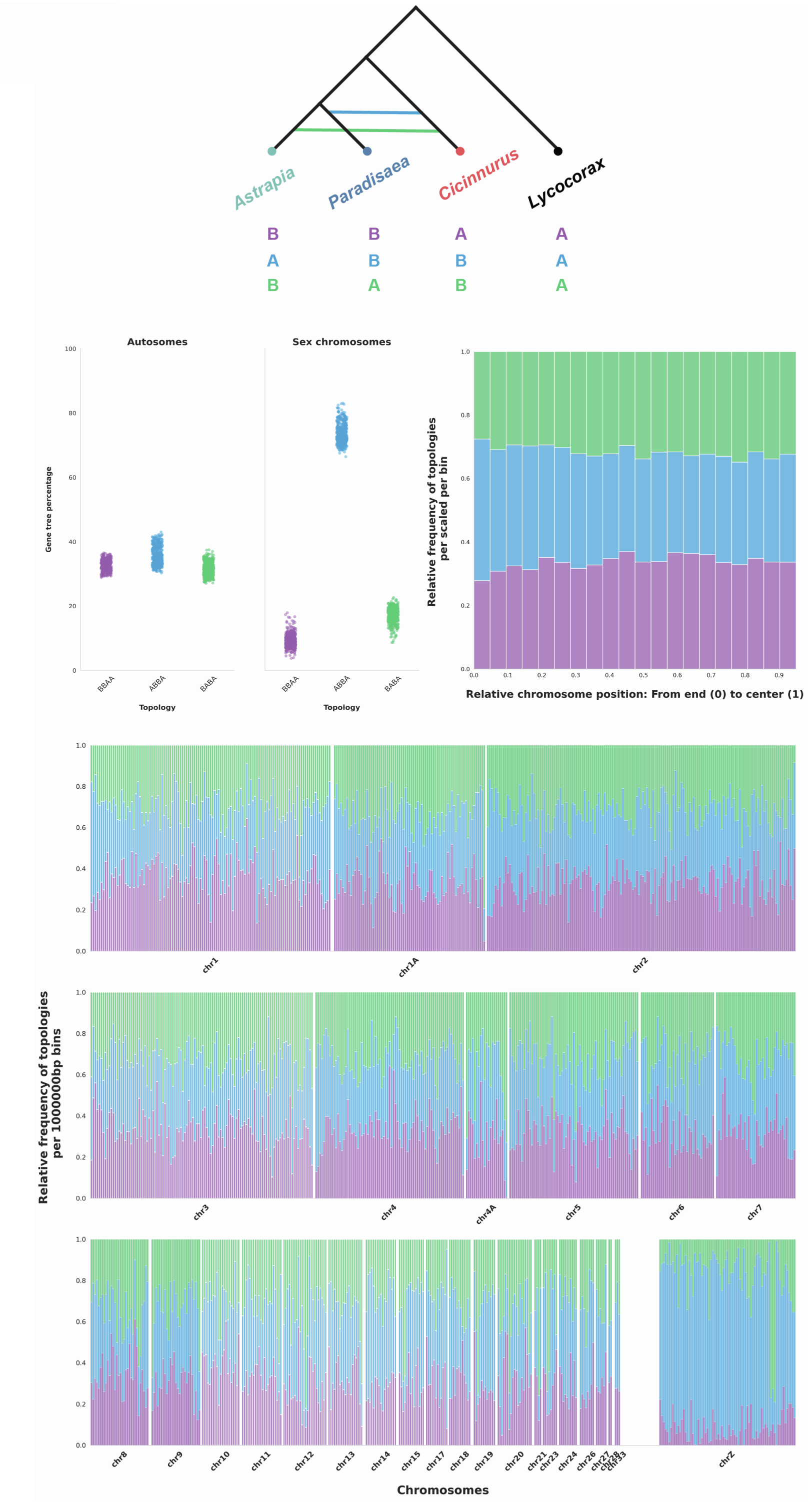

Figure S82. Frequency and genome wide distribution of window tree topologies for each possible Cartesian combination of individuals across four genera. Related to Figure 2 & S74-81. The top-left figure has the relative frequency of ABBA, BABA and BBAA topologies plotted for each of the Cartesian combinations, the top right figure has the distribution of topologies plotted by scaled chromosome position (10 macrochromosomes, summed across all Cartesian combinations) and the bottom plot illustrates the distribution of topologies in bins of 1 Mb. for all chromosomes (summed across all Cartesian combinations). While there is almost equal support for the three possible topologies on the autosomes, on the Z chromosome there is strong support for the topology where *Paradisaea* clusters with *Cicinnurus/Diphyllodes*.

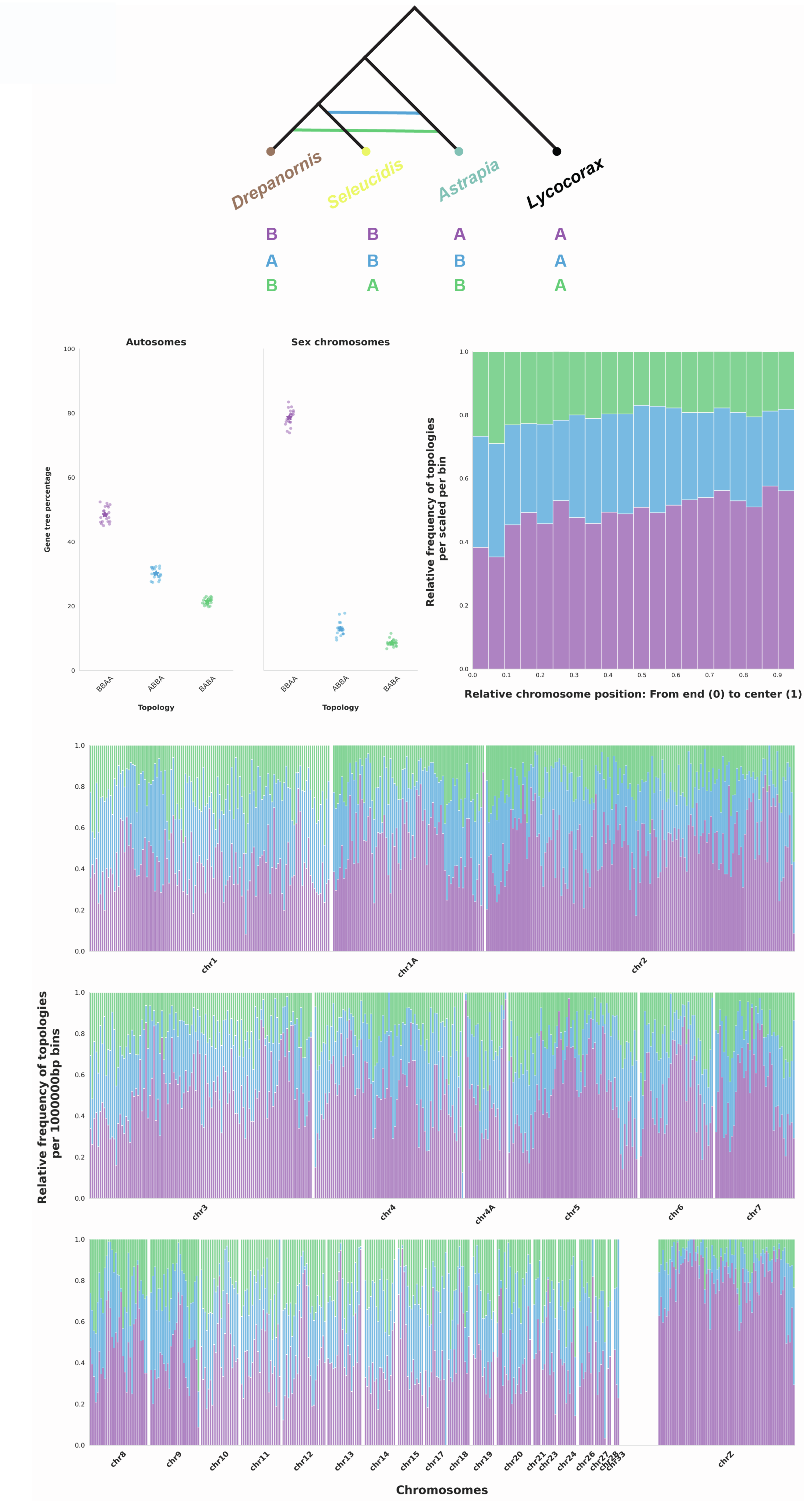

Figure S83. Frequency and genome wide distribution of window tree topologies for each possible Cartesian combination of individuals across four genera. Related to Figure 2 & S84-91. The top-left figure has the relative frequency of ABBA, BABA and BBAA topologies plotted for each of the Cartesian combinations, the top right figure has the distribution of topologies plotted by scaled chromosome position (10 macrochromosomes, summed across all Cartesian combinations) and the bottom plot illustrates the distribution of topologies in bins of 1 Mb. for all chromosomes (summed across all Cartesian combinations). Besides the species tree (BBAA), there is an excess of trees where *Astrapia* and *Seleucidis* are clustered (ABBA), relative to trees where *Astrapia* and *Drepanornis* are clustered (ABBA).

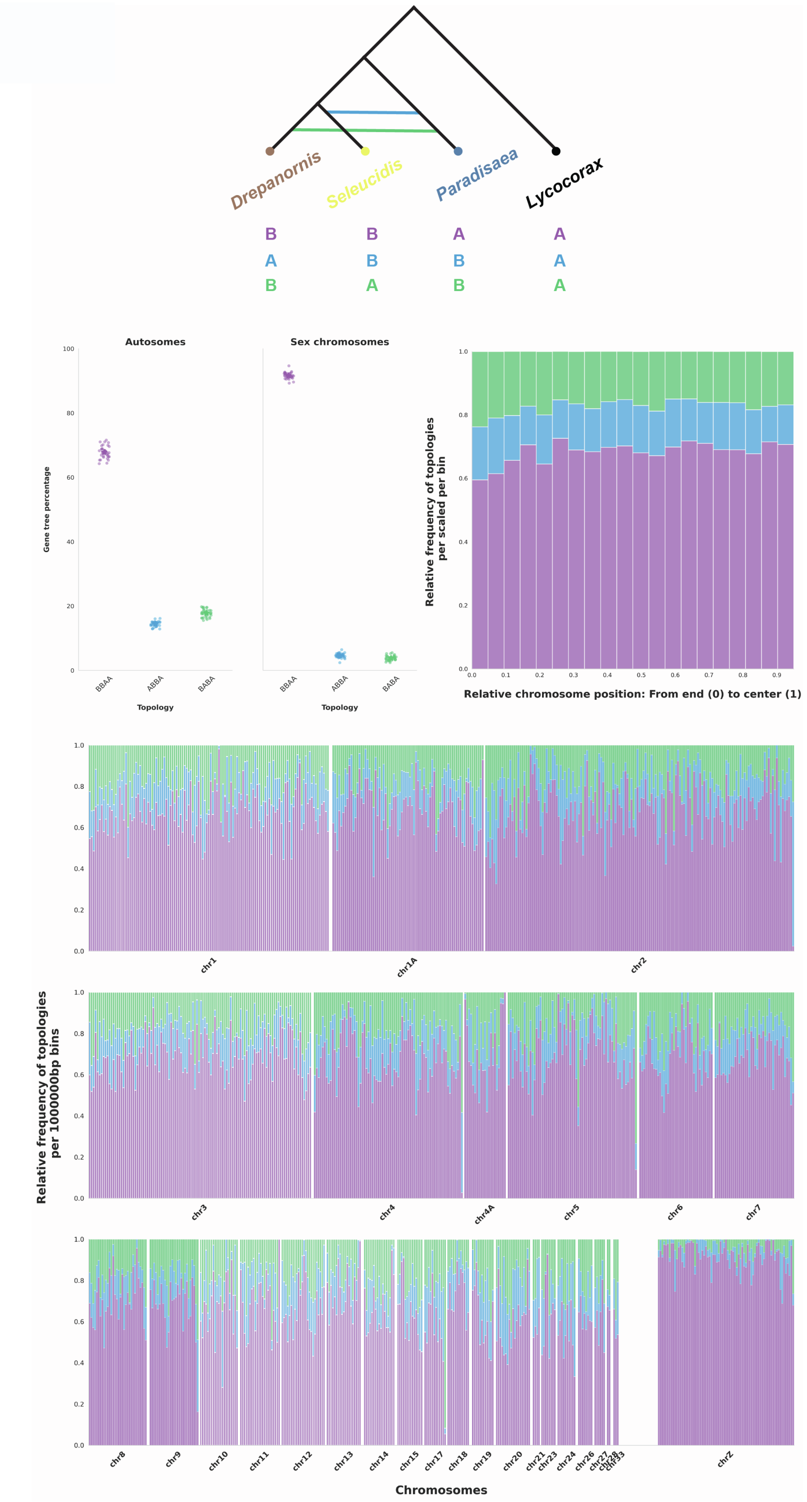

Figure S84. Frequency and genome wide distribution of window tree topologies for each possible Cartesian combination of individuals across four genera. Related to Figure 2 & S83-91. The top-left figure has the relative frequency of ABBA, BABA and BBAA topologies plotted for each of the Cartesian combinations, the top right figure has the distribution of topologies plotted by scaled chromosome position (10 macrochromosomes, summed across all Cartesian combinations) and the bottom plot illustrates the distribution of topologies in bins of 1 Mb. for all chromosomes (summed across all Cartesian combinations). Besides the species tree (BBAA), there is an almost equal proportion of trees where *Paradisaea* and *Drepanornis* are clustered (BABA), relative to trees where *Paradisaea* and *Seleucidis* are clustered (ABBA).

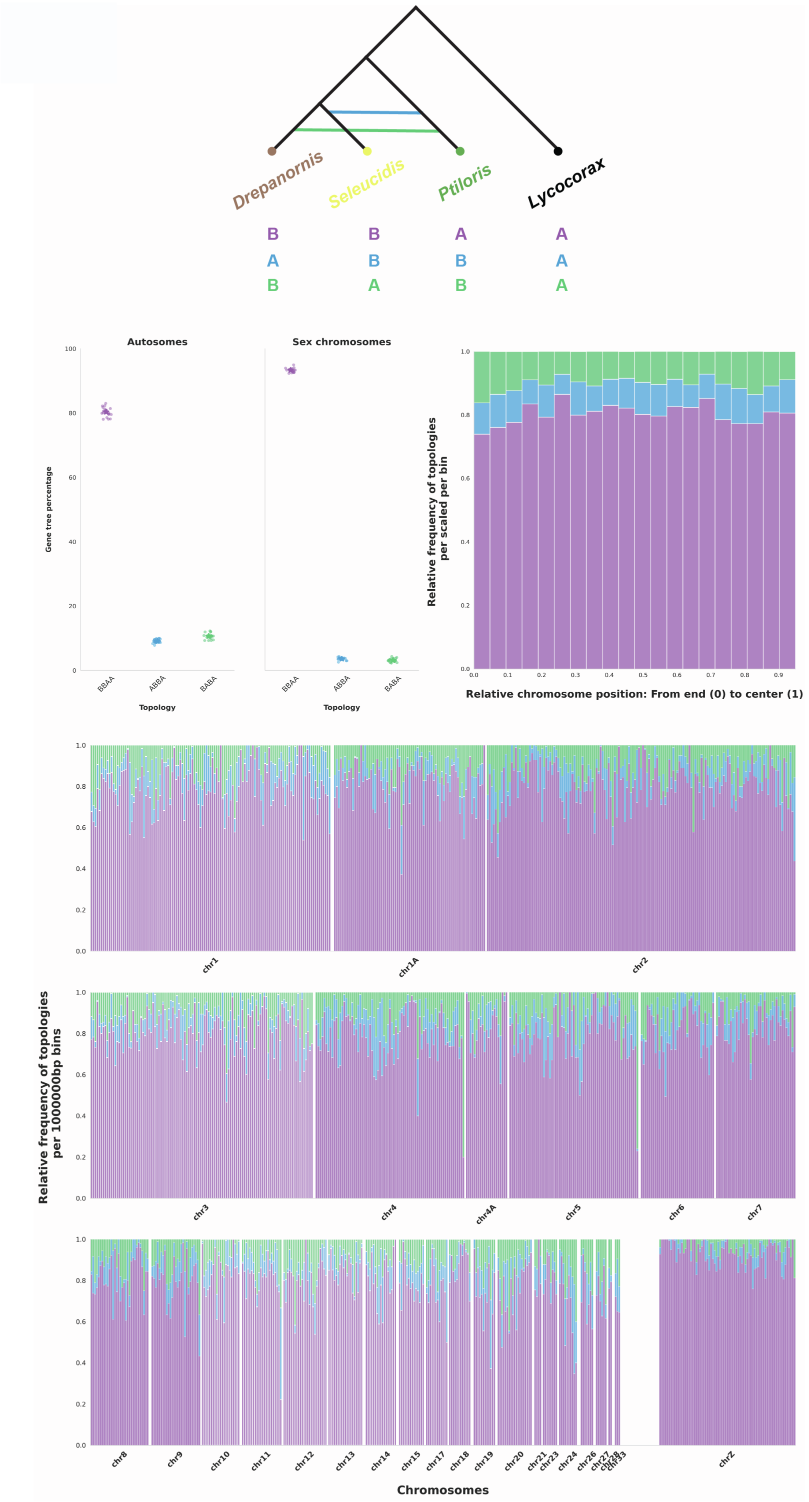

Figure S85. Frequency and genome wide distribution of window tree topologies for each possible Cartesian combination of individuals across four genera. Related to Figure 2 & S83-91. The top-left figure has the relative frequency of ABBA, BABA and BBAA topologies plotted for each of the Cartesian combinations, the top right figure has the distribution of topologies plotted by scaled chromosome position (10 macrochromosomes, summed across all Cartesian combinations) and the bottom plot illustrates the distribution of topologies in bins of 1 Mb. for all chromosomes (summed across all Cartesian combinations). Besides the species tree (BBAA), there is an almost equal proportion of trees where *Ptiloris* and *Drepanornis* are clustered (BABA), relative to trees where *Ptiloris* and *Seleucidis* are clustered (ABBA).

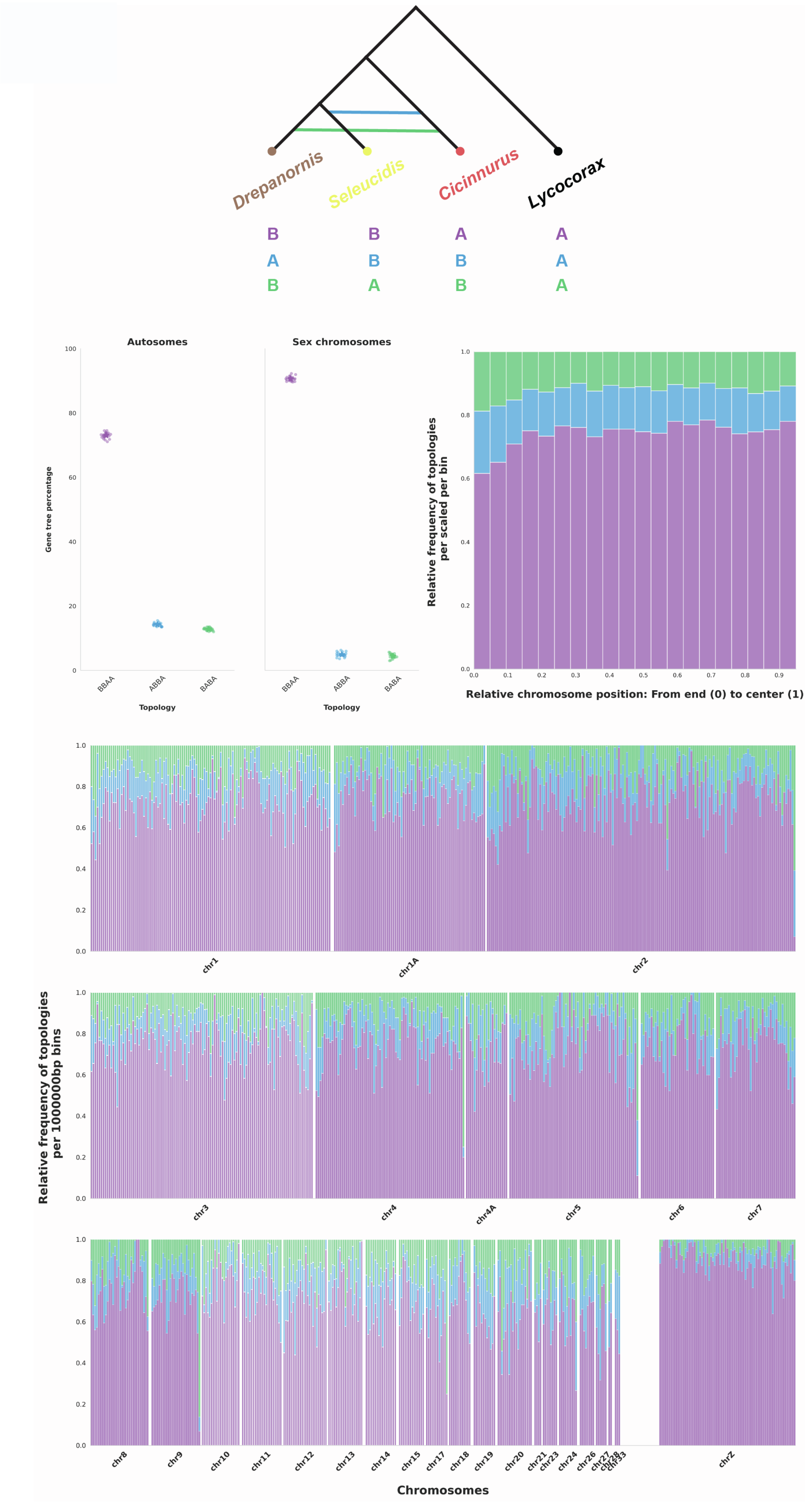

Figure S86. Frequency and genome wide distribution of window tree topologies for each possible Cartesian combination of individuals across four genera. Related to Figure 2 & S83-91. The top-left figure has the relative frequency of ABBA, BABA and BBAA topologies plotted for each of the Cartesian combinations, the top right figure has the distribution of topologies plotted by scaled chromosome position (10 macrochromosomes, summed across all Cartesian combinations) and the bottom plot illustrates the distribution of topologies in bins of 1 Mb. for all chromosomes (summed across all Cartesian combinations). Besides the species tree (BBAA), there is an almost equal proportion of trees where *Cicinnurus/Diphyllodes* and *Drepanornis* are clustered (BABA), relative to trees where *Cicinnurus/Diphyllodes* and *Seleucidis* are clustered (ABBA).

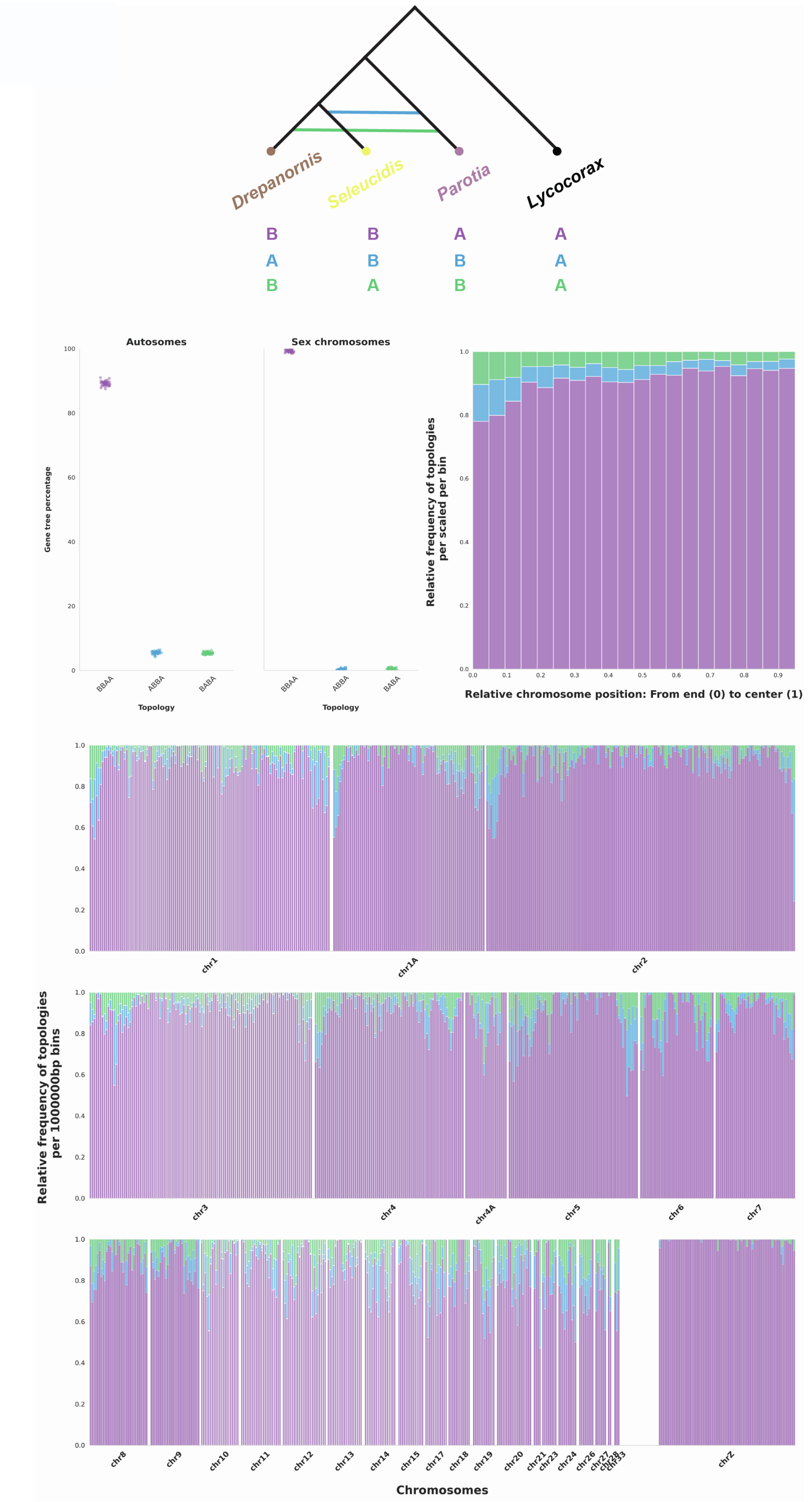

Figure S87. Frequency and genome wide distribution of window tree topologies for each possible Cartesian combination of individuals across four genera. Related to Figure 2 & S83-91. The top-left figure has the relative frequency of ABBA, BABA and BBAA topologies plotted for each of the Cartesian combinations, the top right figure has the distribution of topologies plotted by scaled chromosome position (10 macrochromosomes, summed across all Cartesian combinations) and the bottom plot illustrates the distribution of topologies in bins of 1 Mb. for all chromosomes (summed across all Cartesian combinations). Besides the species tree (BBAA), there is an almost equal proportion of trees where *Parotia* and *Drepanornis* are clustered (BABA), relative to trees where *Parotia* and *Seleucidis* are clustered (ABBA).

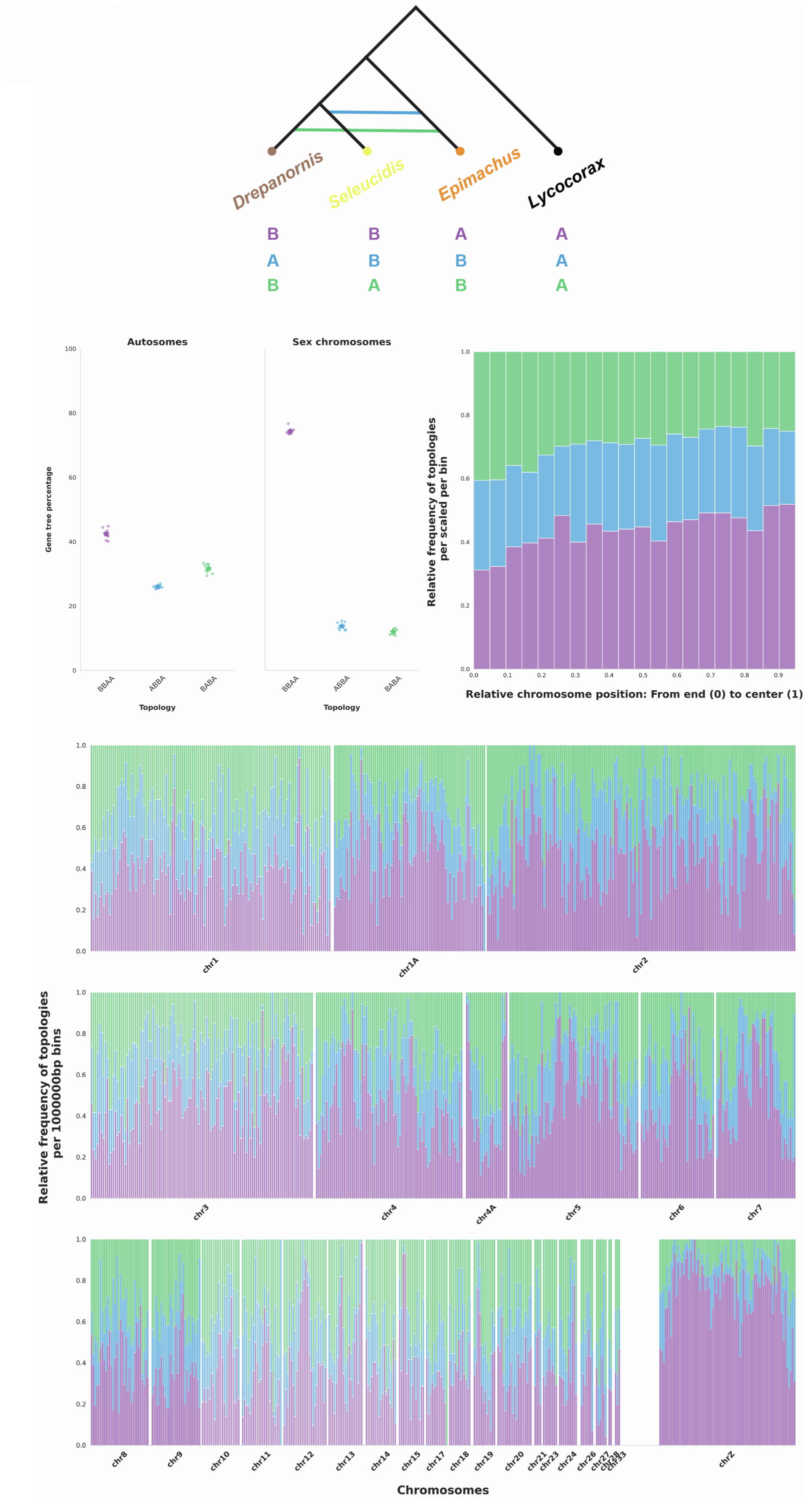

Figure S88. Frequency and genome wide distribution of window tree topologies for each possible Cartesian combination of individuals across four genera. Related to Figure 2 & S83-91. The top-left figure has the relative frequency of ABBA, BABA and BBAA topologies plotted for each of the Cartesian combinations, the top right figure has the distribution of topologies plotted by scaled chromosome position (10 macrochromosomes, summed across all Cartesian combinations) and the bottom plot illustrates the distribution of topologies in bins of 1 Mb. for all chromosomes (summed across all Cartesian combinations). Besides the species tree (BBAA), there is an excess of trees where *Epimachus* and *Drepanornis* are clustered (BABA), relative to trees where *Epimachus* and *Seleucidis* are clustered (ABBA).

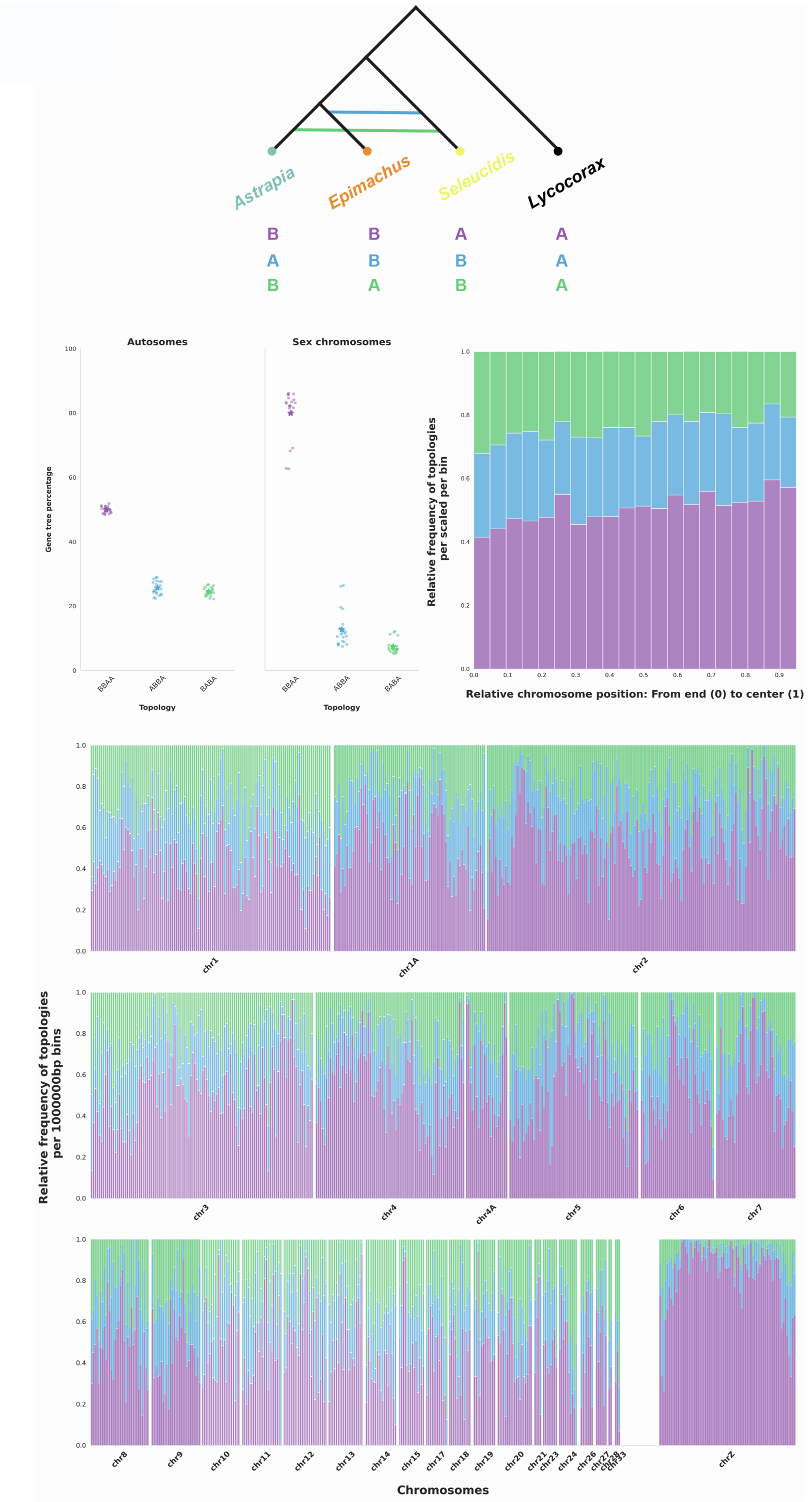

Figure S89. Frequency and genome wide distribution of window tree topologies for each possible Cartesian combination of individuals across four genera. Related to Figure 2 & S83-91. The top-left figure has the relative frequency of ABBA, BABA and BBAA topologies plotted for each of the Cartesian combinations, the top right figure has the distribution of topologies plotted by scaled chromosome position (10 macrochromosomes, summed across all Cartesian combinations) and the bottom plot illustrates the distribution of topologies in bins of 1 Mb. for all chromosomes (summed across all Cartesian combinations). Besides the species tree (BBAA), there is an almost equal proportion of trees where *Seleucidis* and *Epimachus* are clustered (ABBA), relative to trees where *Seleucidis* and *Astrapia* are clustered (BABA).

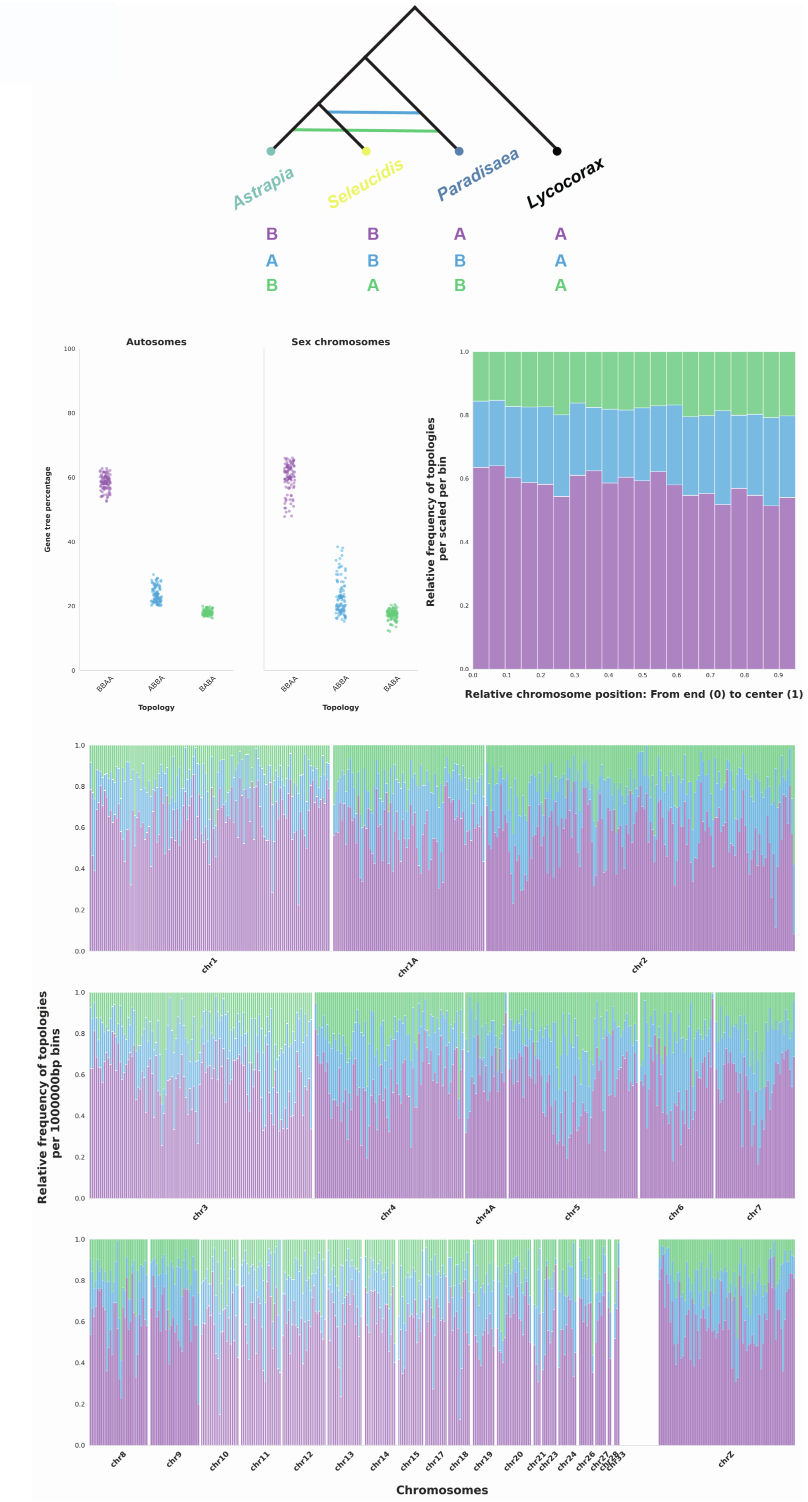

Figure S90. Frequency and genome wide distribution of window tree topologies for each possible Cartesian combination of individuals across four genera. Related to Figure 2 & S83-91. The top-left figure has the relative frequency of ABBA, BABA and BBAA topologies plotted for each of the Cartesian combinations, the top right figure has the distribution of topologies plotted by scaled chromosome position (10 macrochromosomes, summed across all Cartesian combinations) and the bottom plot illustrates the distribution of topologies in bins of 1 Mb. for all chromosomes (summed across all Cartesian combinations). Besides the species tree (BBAA), there is a minute excess of trees where *Paradisaea* and *Seleucidis* are clustered (ABBA), relative to trees where *Paradisaea* and *Astrapia* are clustered (BABA).

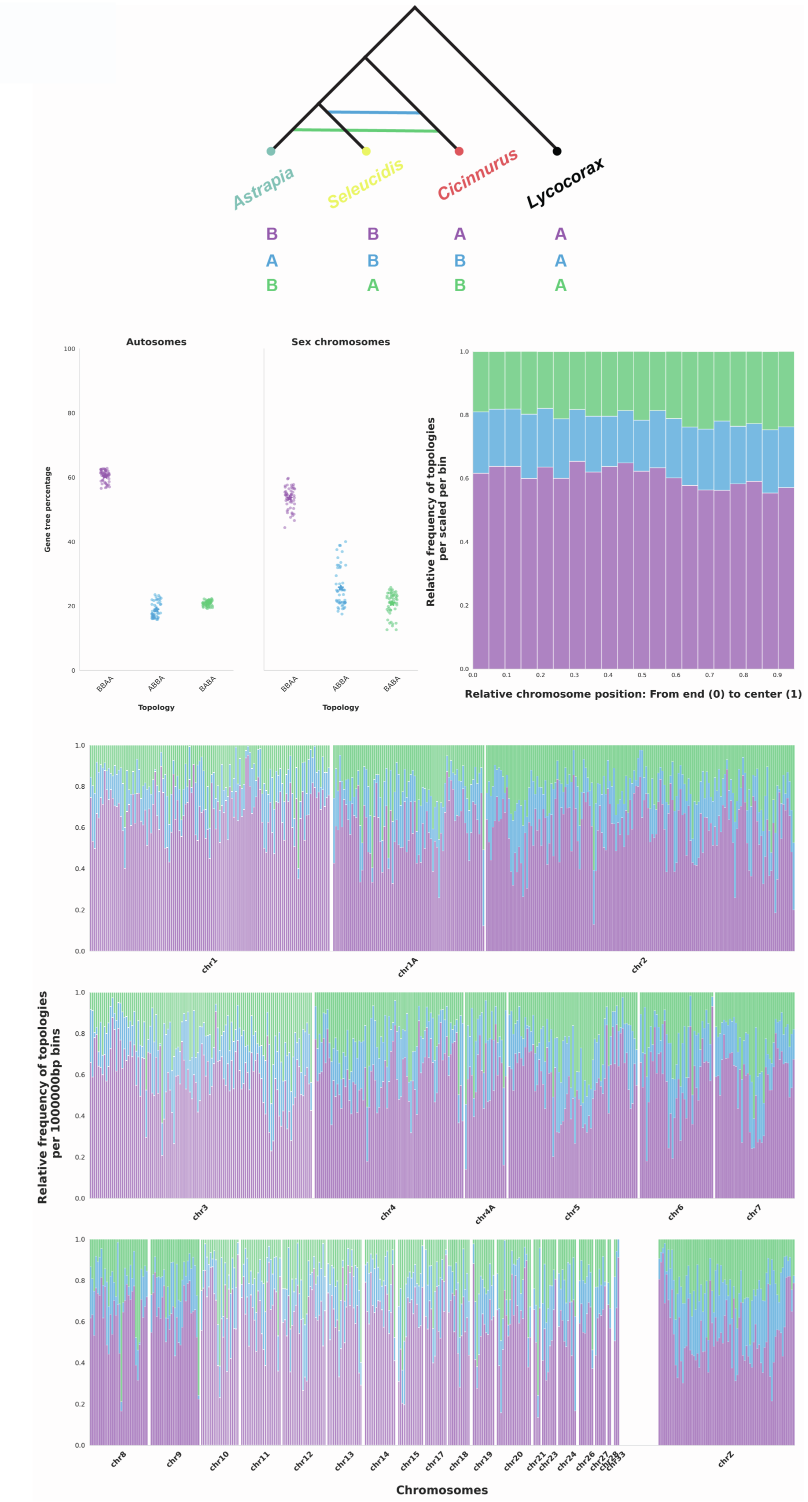

Figure S91. Frequency and genome wide distribution of window tree topologies for each possible Cartesian combination of individuals across four genera. Related to Figure 2 & S83-90. The top-left figure has the relative frequency of ABBA, BABA and BBAA topologies plotted for each of the Cartesian combinations, the top right figure has the distribution of topologies plotted by scaled chromosome position (10 macrochromosomes, summed across all Cartesian combinations) and the bottom plot illustrates the distribution of topologies in bins of 1 Mb. for all chromosomes (summed across all Cartesian combinations). Besides the species tree (BBAA), there is a minute excess of trees where *Cicinnurus/Diphyllodes* and *Seleucidis* are clustered on the Z chromosome (ABBA), relative to trees where *Cicinnurus/Diphyllodes* and *Astrapia* are clustered (BABA). This difference is absent on the autosomes.

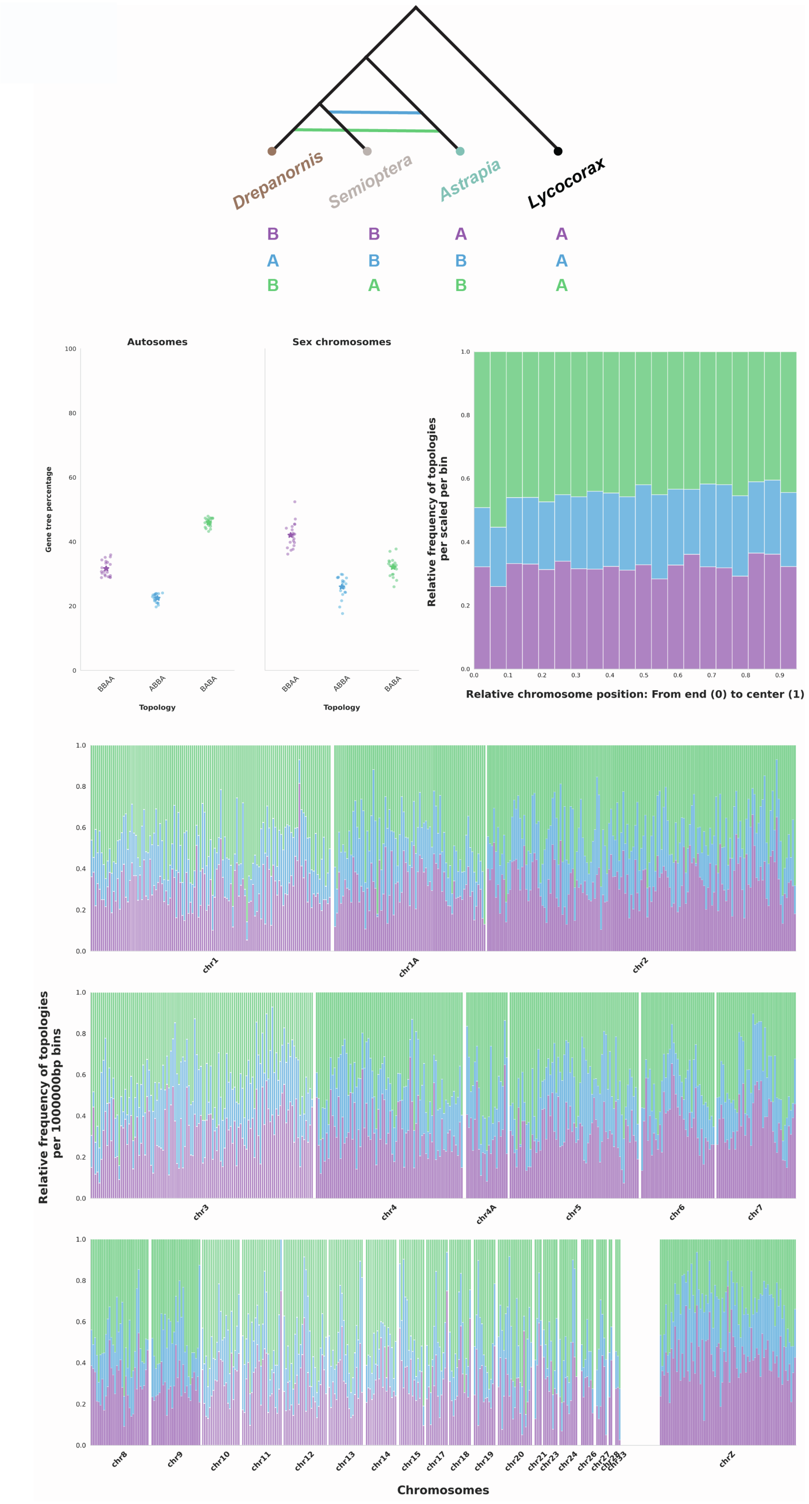

Figure S92. Frequency and genome wide distribution of window tree topologies for each possible Cartesian combination of individuals across four genera. Related to Figure 2 & S93-100. The top-left figure has the relative frequency of ABBA, BABA and BBAA topologies plotted for each of the Cartesian combinations, the top right figure has the distribution of topologies plotted by scaled chromosome position (10 macrochromosomes, summed across all Cartesian combinations) and the bottom plot illustrates the distribution of topologies in bins of 1 Mb. for all chromosomes (summed across all Cartesian combinations). There is discordance between Z and autosomes in the most frequently recovered topology. On the autosomes, *Drepanornis* is more closely related to *Astrapia* (BABA), whereas *Drepanornis* is more closely related to *Semioptera* on the Z chromosome (BBAA).

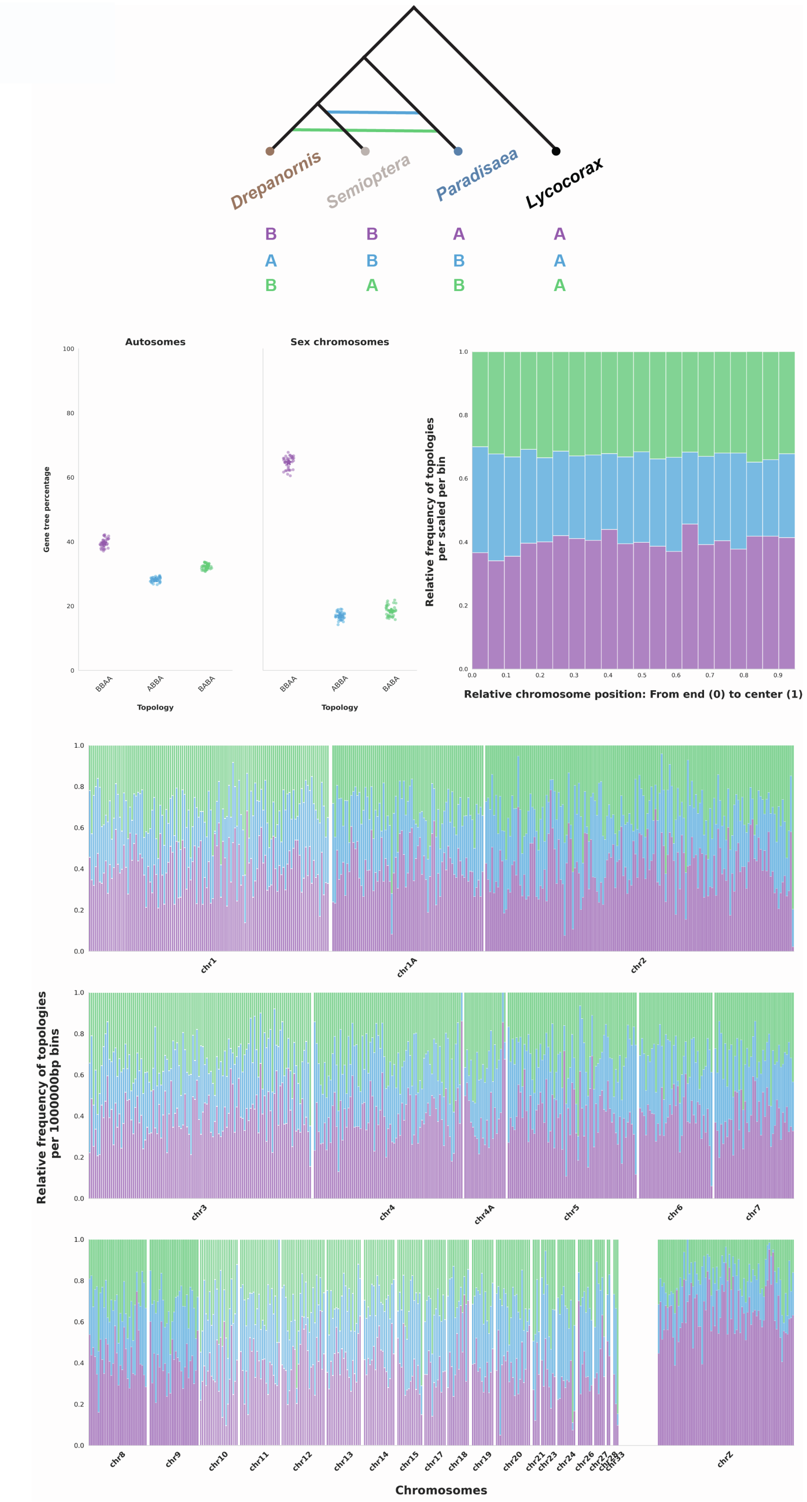

Figure S93. Frequency and genome wide distribution of window tree topologies for each possible Cartesian combination of individuals across four genera. Related to Figure 2 & S92-100. The top-left figure has the relative frequency of ABBA, BABA and BBAA topologies plotted for each of the Cartesian combinations, the top right figure has the distribution of topologies plotted by scaled chromosome position (10 macrochromosomes, summed across all Cartesian combinations) and the bottom plot illustrates the distribution of topologies in bins of 1 Mb. for all chromosomes (summed across all Cartesian combinations). Besides the species tree (BBAA), there is a minute excess of trees where *Drepanornis* and *Paradisaea* are clustered (BABA), relative to trees where *Semioptera* and *Paradisaea* are clustered (ABBA). However, on the autosomes, the frequencies of the observed topologies is relatively equal, whereas the difference between the species tree topology and the alternative topologies is more pronounced on the Z chromosome.

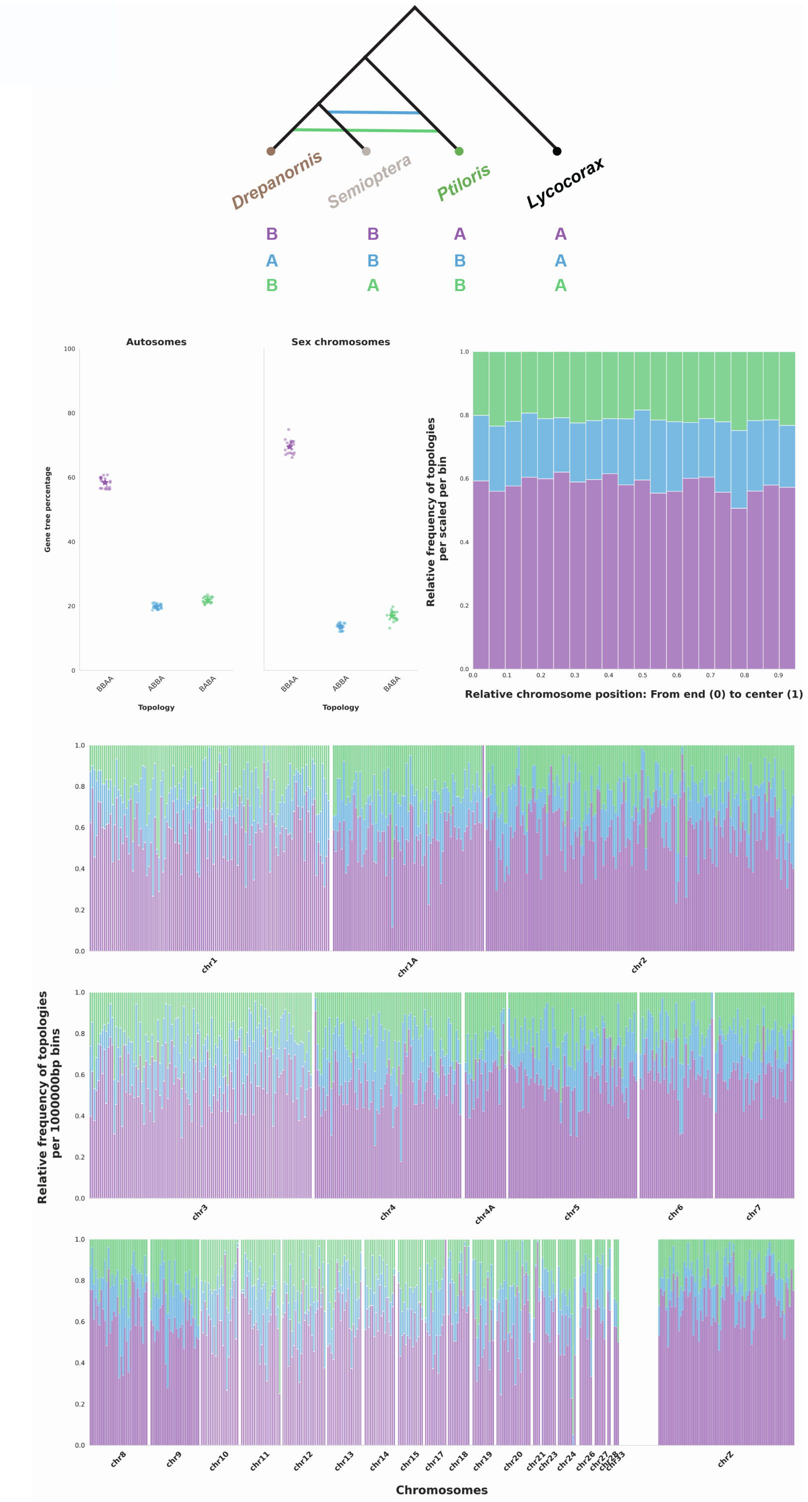

Figure S94. Frequency and genome wide distribution of window tree topologies for each possible Cartesian combination of individuals across four genera. Related to Figure 2 & S92-100. The top-left figure has the relative frequency of ABBA, BABA and BBAA topologies plotted for each of the Cartesian combinations, the top right figure has the distribution of topologies plotted by scaled chromosome position (10 macrochromosomes, summed across all Cartesian combinations) and the bottom plot illustrates the distribution of topologies in bins of 1 Mb. for all chromosomes (summed across all Cartesian combinations). Besides the species tree (BBAA), there is an equal proportion of trees where *Ptiloris* and *Semioptera* are clustered (ABBA), relative to trees where *Ptiloris* and *Drepanornis* are clustered (BABA).

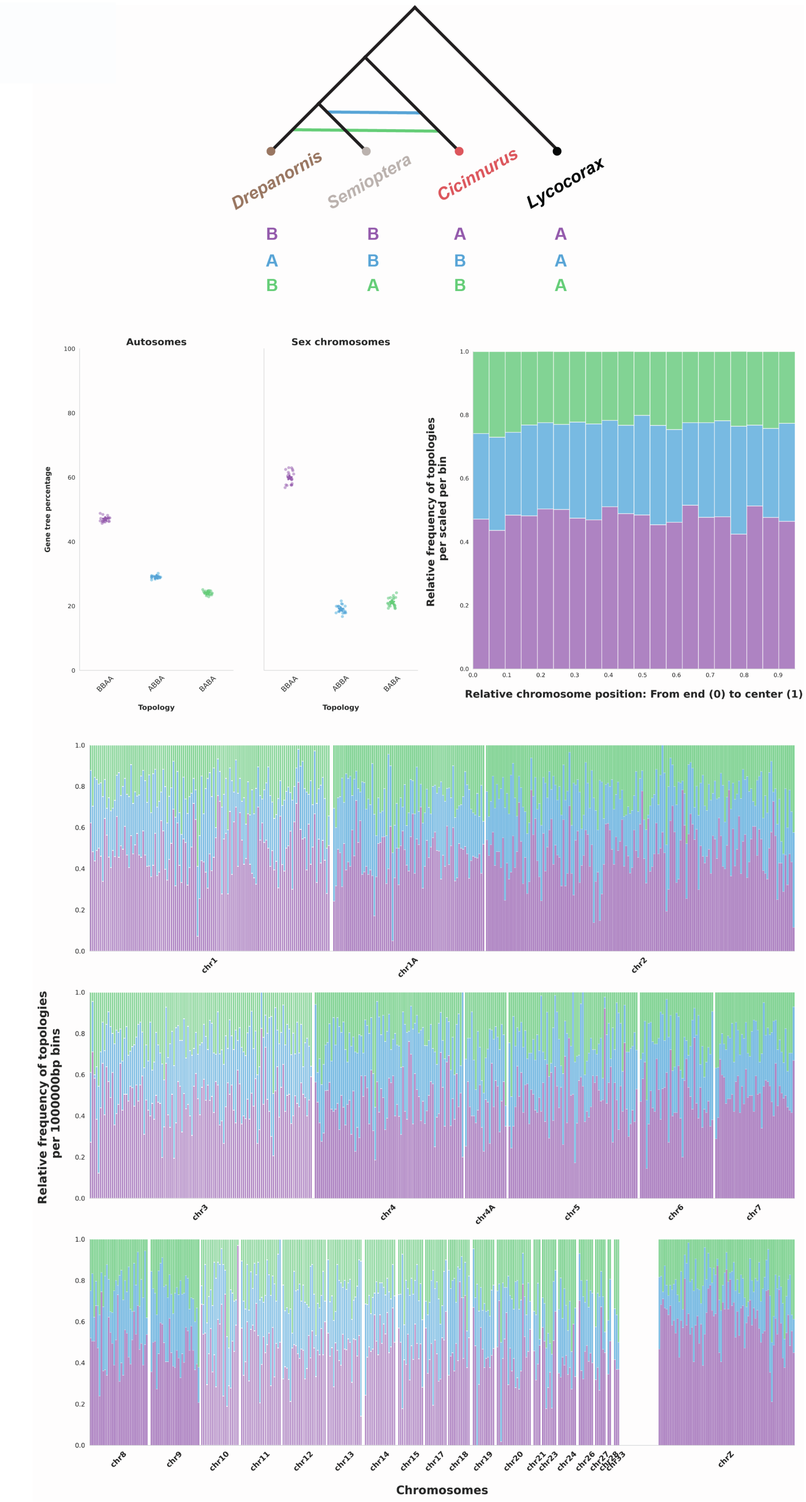

Figure S95. Frequency and genome wide distribution of window tree topologies for each possible Cartesian combination of individuals across four genera. Related to Figure 2 & S92-100. The top-left figure has the relative frequency of ABBA, BABA and BBAA topologies plotted for each of the Cartesian combinations, the top right figure has the distribution of topologies plotted by scaled chromosome position (10 macrochromosomes, summed across all Cartesian combinations) and the bottom plot illustrates the distribution of topologies in bins of 1 Mb. for all chromosomes (summed across all Cartesian combinations). Besides the species tree (BBAA), there is an almost equal proportion of trees where *Cicinnurus/Diphyllodes* and *Semioptera* are clustered (ABBA), relative to trees where *Cicinnurus/Diphyllodes* and *Drepanornis* are clustered (BABA).

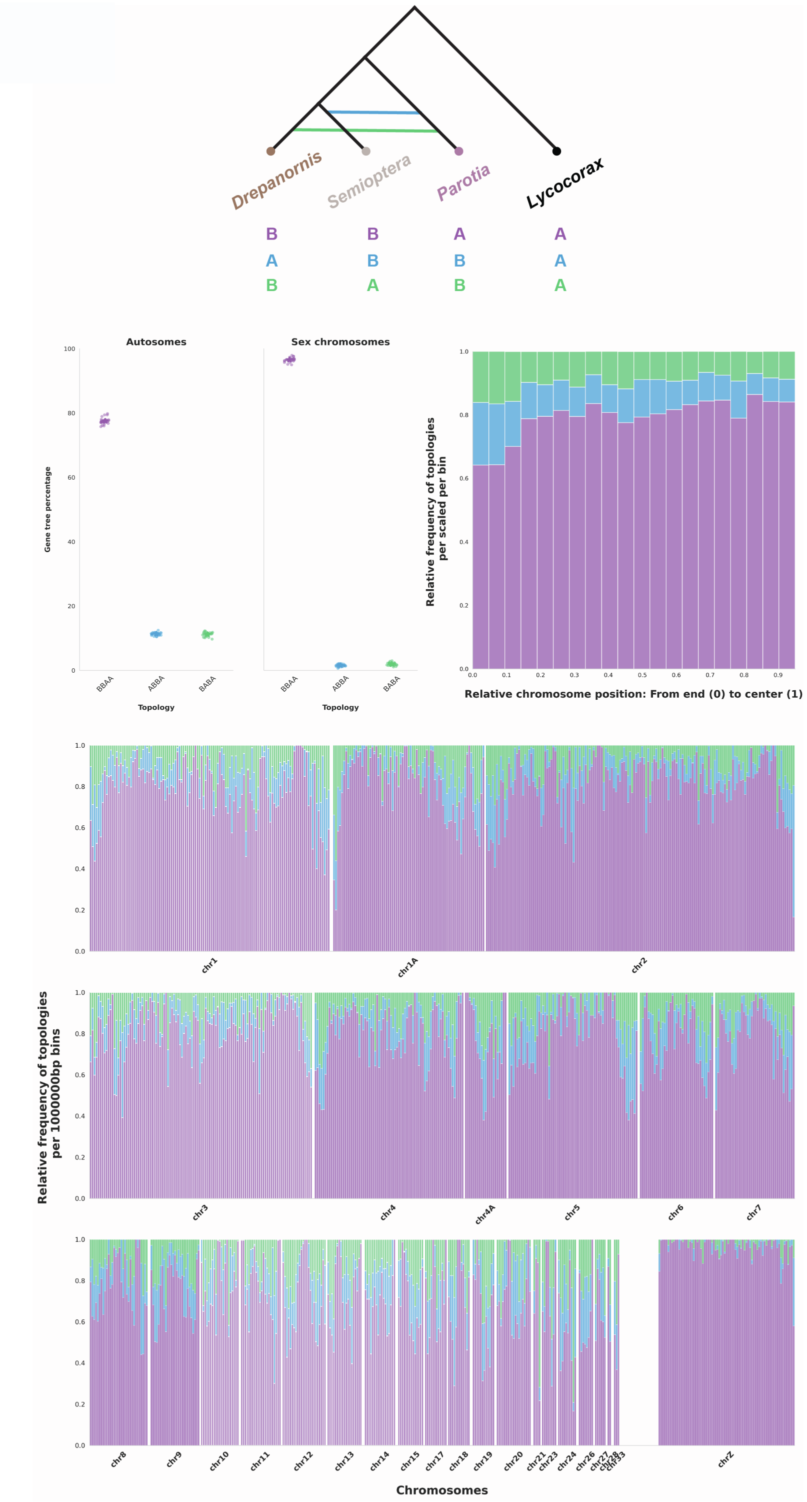

Figure S96. Frequency and genome wide distribution of window tree topologies for each possible Cartesian combination of individuals across four genera. Related to Figure 2 & S92-100. The top-left figure has the relative frequency of ABBA, BABA and BBAA topologies plotted for each of the Cartesian combinations, the top right figure has the distribution of topologies plotted by scaled chromosome position (10 macrochromosomes, summed across all Cartesian combinations) and the bottom plot illustrates the distribution of topologies in bins of 1 Mb. for all chromosomes (summed across all Cartesian combinations). Besides the species tree (BBAA), there is an equal proportion of trees where *Parotia* and *Semioptera* are clustered (ABBA), relative to trees where *Parotia* and *Drepanornis* are clustered (BABA).

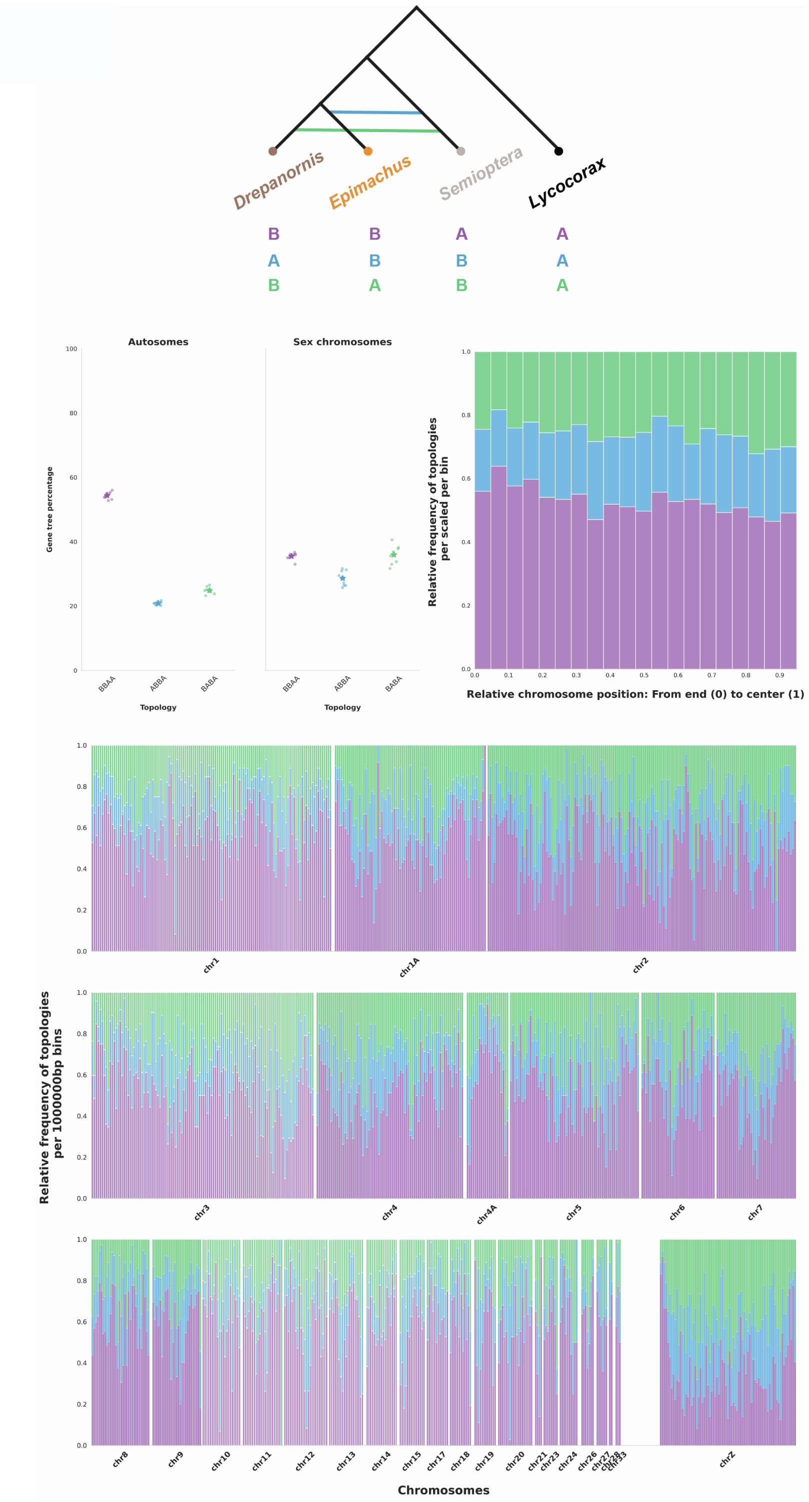

Figure S97. Frequency and genome wide distribution of window tree topologies for each possible Cartesian combination of individuals across four genera. Related to Figure 2 & S92-100. The top-left figure has the relative frequency of ABBA, BABA and BBAA topologies plotted for each of the Cartesian combinations, the top right figure has the distribution of topologies plotted by scaled chromosome position (10 macrochromosomes, summed across all Cartesian combinations) and the bottom plot illustrates the distribution of topologies in bins of 1 Mb. for all chromosomes (summed across all Cartesian combinations). There is discordance between Z and autosomes in the most frequently recovered topology. On the autosomes, *Drepanornis* is more closely related to *Epimachus* (BBAA), but there is almost equal support for the three possible topologies on the Z chromosome. Interestingly, the relative frequency of the three possible topologies vary across chromosomes.

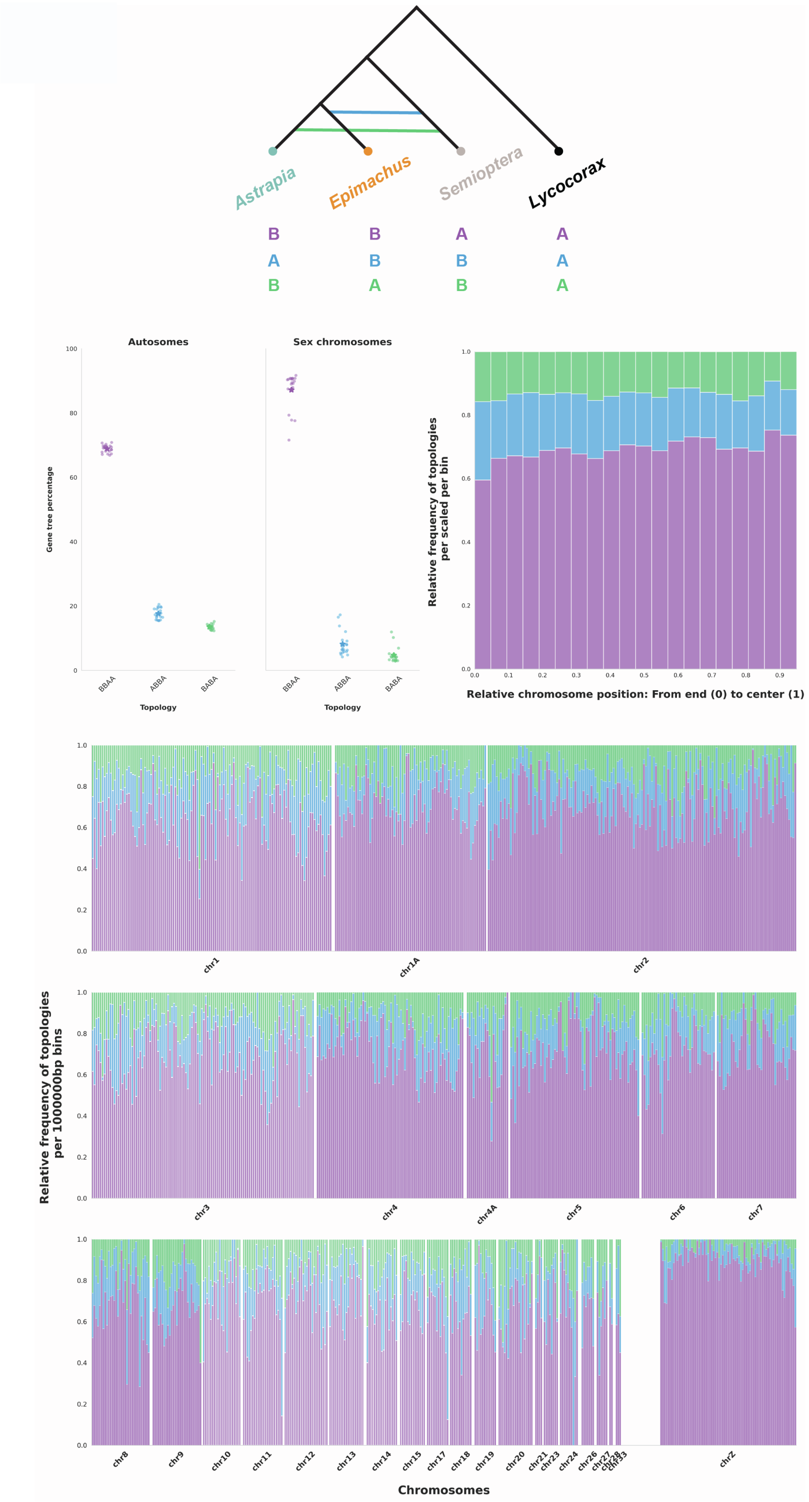

Figure S98. Frequency and genome wide distribution of window tree topologies for each possible Cartesian combination of individuals across four genera. Related to Figure 2 & S92-100. The top-left figure has the relative frequency of ABBA, BABA and BBAA topologies plotted for each of the Cartesian combinations, the top right figure has the distribution of topologies plotted by scaled chromosome position (10 macrochromosomes, summed across all Cartesian combinations) and the bottom plot illustrates the distribution of topologies in bins of 1 Mb. for all chromosomes (summed across all Cartesian combinations). Besides the species tree (BBAA), there is an almost equal proportion of trees where *Epimachus* and *Semioptera* are clustered (ABBA), relative to trees where *Astrapia* and *Semioptera* are clustered (BABA).

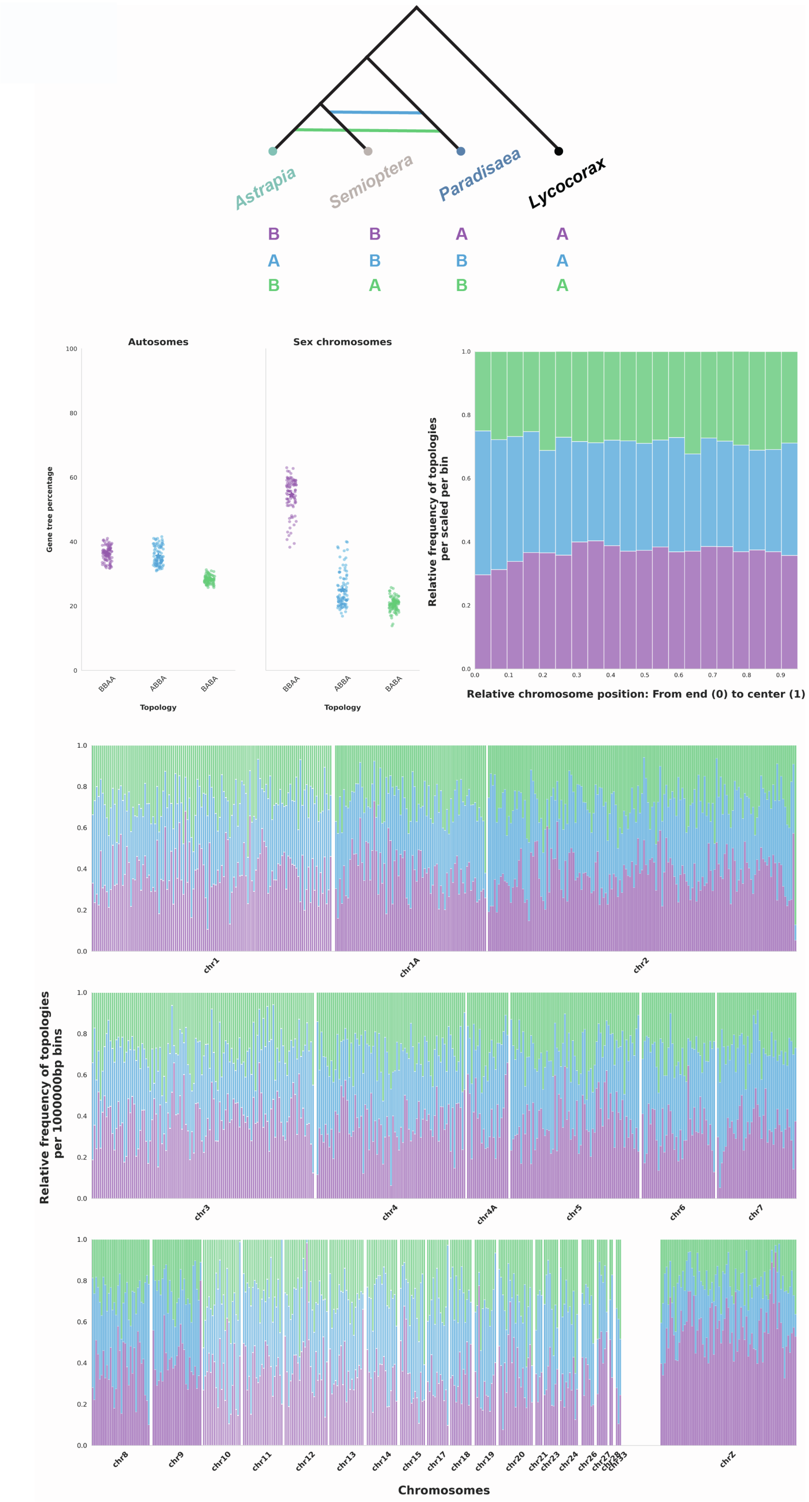

Figure S99. Frequency and genome wide distribution of window tree topologies for each possible Cartesian combination of individuals across four genera. Related to Figure 2 & S92-100. The top-left figure has the relative frequency of ABBA, BABA and BBAA topologies plotted for each of the Cartesian combinations, the top right figure has the distribution of topologies plotted by scaled chromosome position (10 macrochromosomes, summed across all Cartesian combinations) and the bottom plot illustrates the distribution of topologies in bins of 1 Mb. for all chromosomes (summed across all Cartesian combinations). There is discordance between Z and autosomes in the most frequently recovered topology. On the autosomes, there is almost equal support for the three possible topologies. However, *Astrapia* is more closely related to *Semioptera* (BBAA) on the Z chromosome.

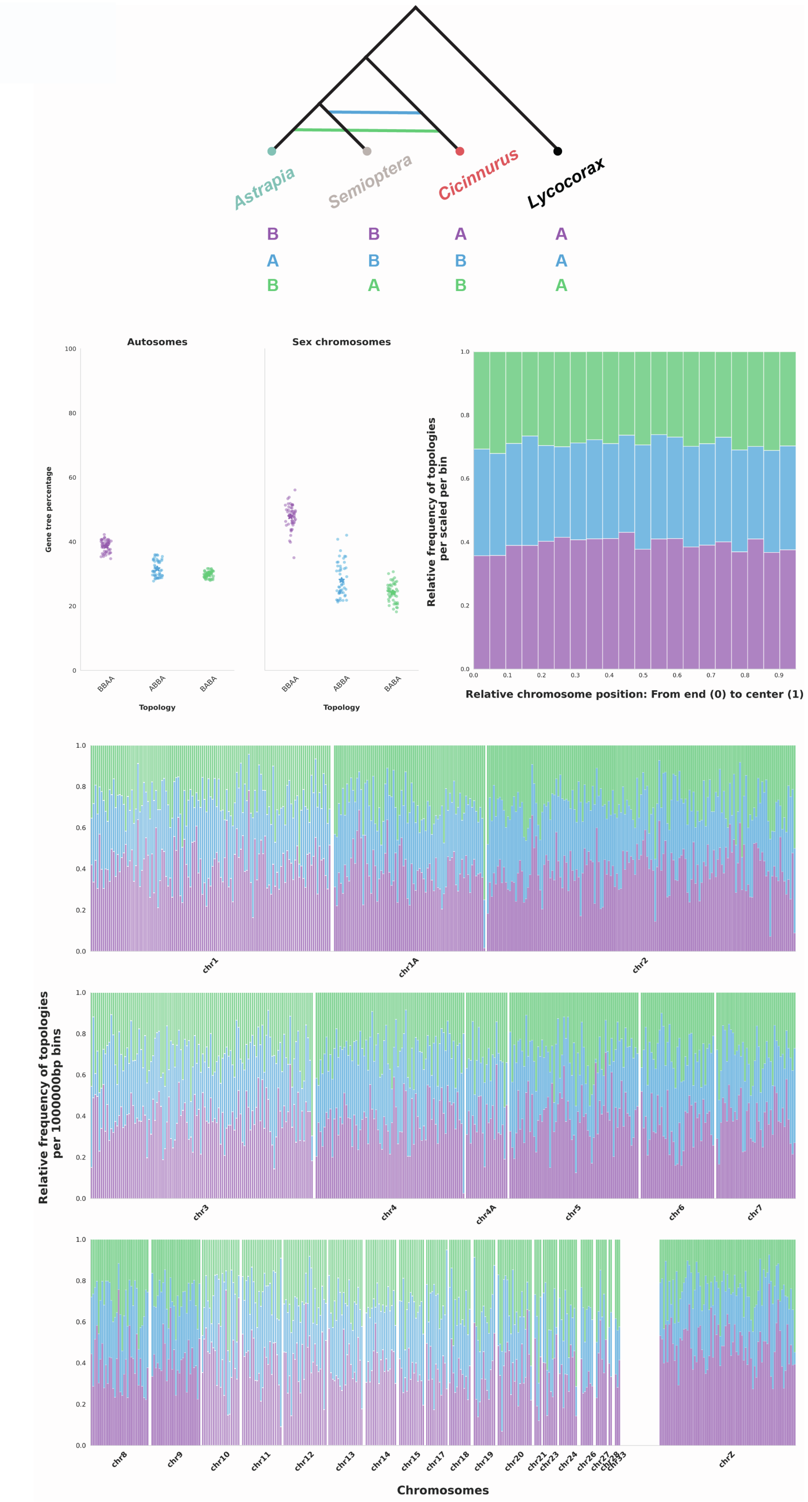

Figure S100. Frequency and genome wide distribution of window tree topologies for each possible Cartesian combination of individuals across four genera. Related to Figure 2 & S92-99. The top-left figure has the relative frequency of ABBA, BABA and BBAA topologies plotted for each of the Cartesian combinations, the top right figure has the distribution of topologies plotted by scaled chromosome position (10 macrochromosomes, summed across all Cartesian combinations) and the bottom plot illustrates the distribution of topologies in bins of 1 Mb. for all chromosomes (summed across all Cartesian combinations). There is discordance between Z and autosomes in the most frequently recovered topology. On the autosomes, there is almost equal support for the three possible topologies. However, *Astrapia* is more closely related to *Semioptera* (BBAA) on the Z chromosome.

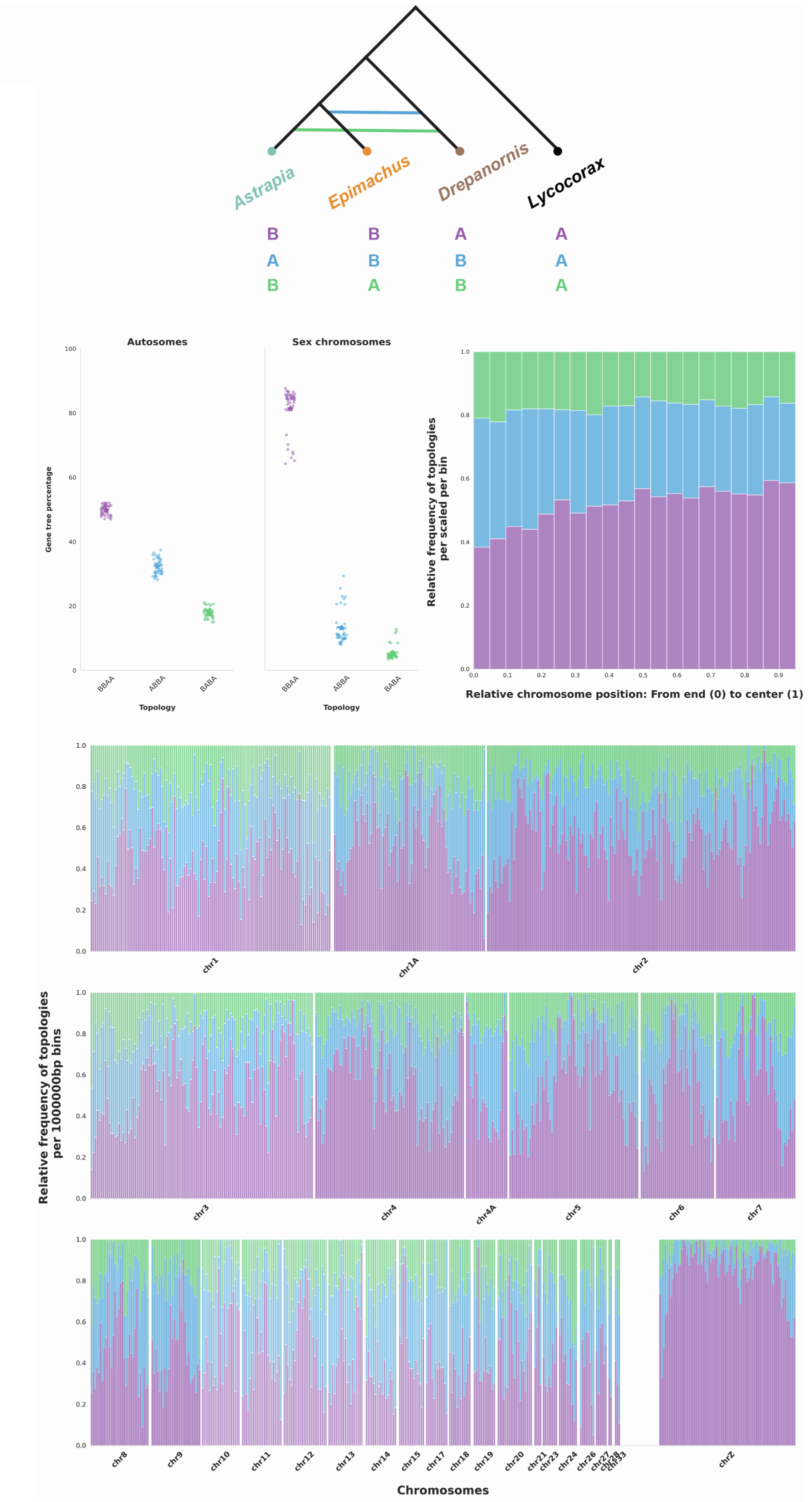

Figure S101. Frequency and genome wide distribution of window tree topologies for each possible Cartesian combination of individuals across four genera. Related to Figure 2 & S102-107. The top-left figure has the relative frequency of ABBA, BABA and BBAA topologies plotted for each of the Cartesian combinations, the top right figure has the distribution of topologies plotted by scaled chromosome position (10 macrochromosomes, summed across all Cartesian combinations) and the bottom plot illustrates the distribution of topologies in bins of 1 Mb. for all chromosomes (summed across all Cartesian combinations). Besides the species tree (BBAA), there is an excess of trees where *Epimachus* and *Drepanornis* are clustered (ABBA), relative to trees where *Drepanornis* and *Astrapia* are clustered (BABA). ABBA topologies are more frequently found towards the end of chromosomes.

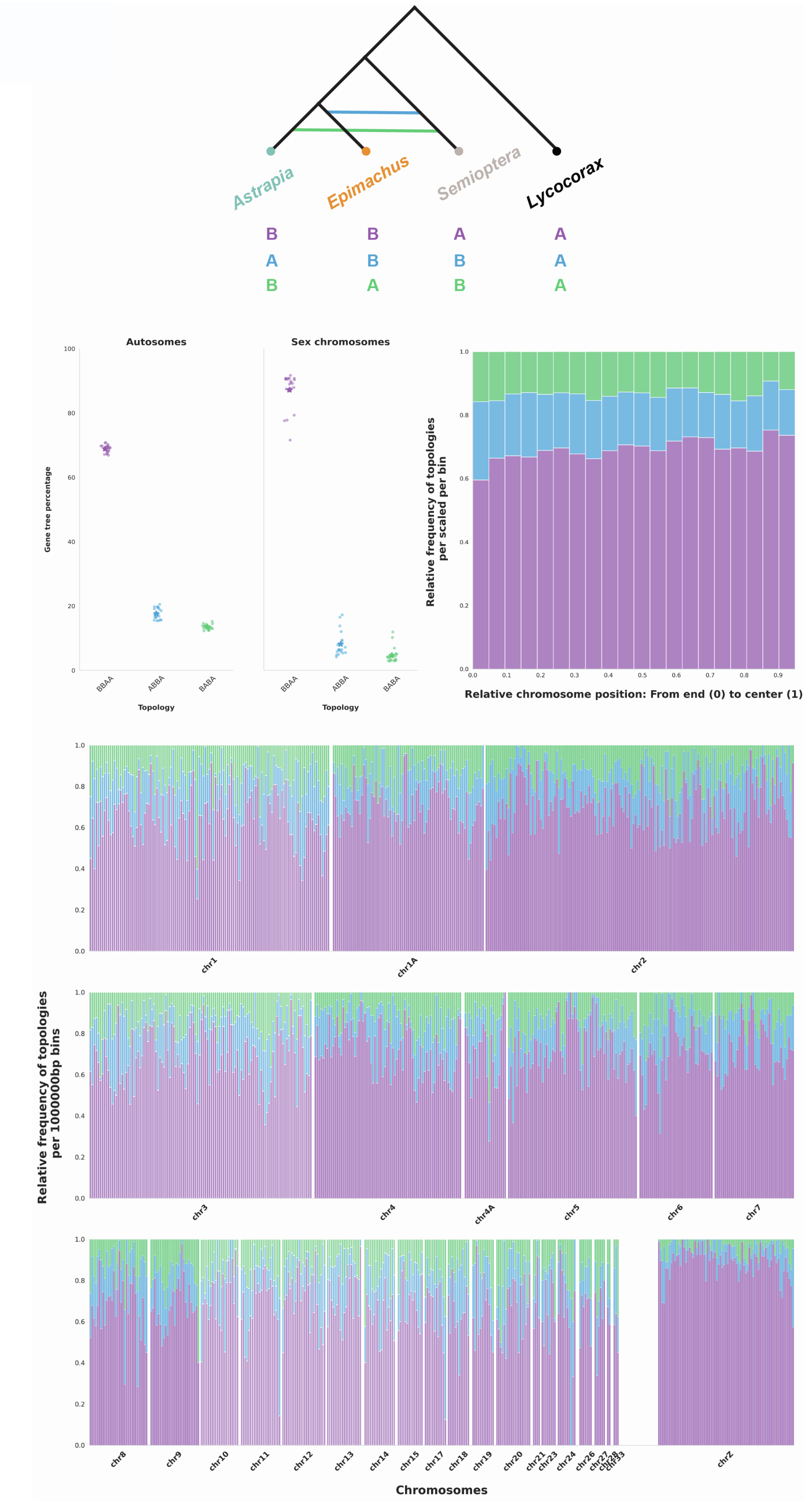

Figure S102. Frequency and genome wide distribution of window tree topologies for each possible Cartesian combination of individuals across four genera. Related to Figure 2 & S101-107. The top-left figure has the relative frequency of ABBA, BABA and BBAA topologies plotted for each of the Cartesian combinations, the top right figure has the distribution of topologies plotted by scaled chromosome position (10 macrochromosomes, summed across all Cartesian combinations) and the bottom plot illustrates the distribution of topologies in bins of 1 Mb. for all chromosomes (summed across all Cartesian combinations). Besides the species tree (BBAA), there is an almost equal proportion of trees where *Epimachus* and *Semioptera* are clustered (ABBA), relative to trees where *Astrapia* and *Semioptera* are clustered (BABA).

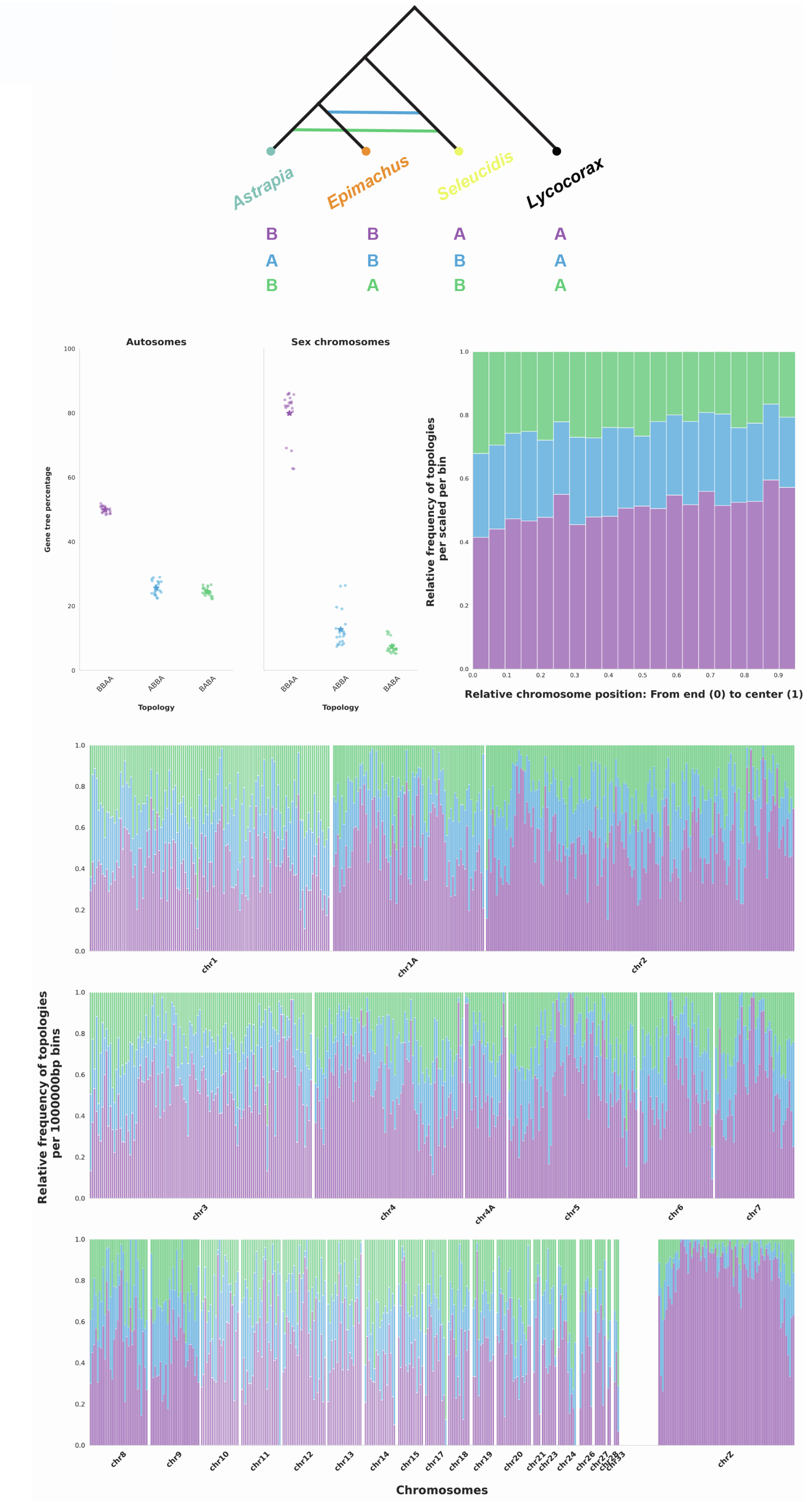

Figure S103. Frequency and genome wide distribution of window tree topologies for each possible Cartesian combination of individuals across four genera. Related to Figure 2 & S101-107. The top-left figure has the relative frequency of ABBA, BABA and BBAA topologies plotted for each of the Cartesian combinations, the top right figure has the distribution of topologies plotted by scaled chromosome position (10 macrochromosomes, summed across all Cartesian combinations) and the bottom plot illustrates the distribution of topologies in bins of 1 Mb. for all chromosomes (summed across all Cartesian combinations). Besides the species tree (BBAA), there is an equal proportion of trees where *Epimachus* and *Seleucidis* are clustered (ABBA), relative to trees where *Astrapia* and *Seleucidis* are clustered (BABA).

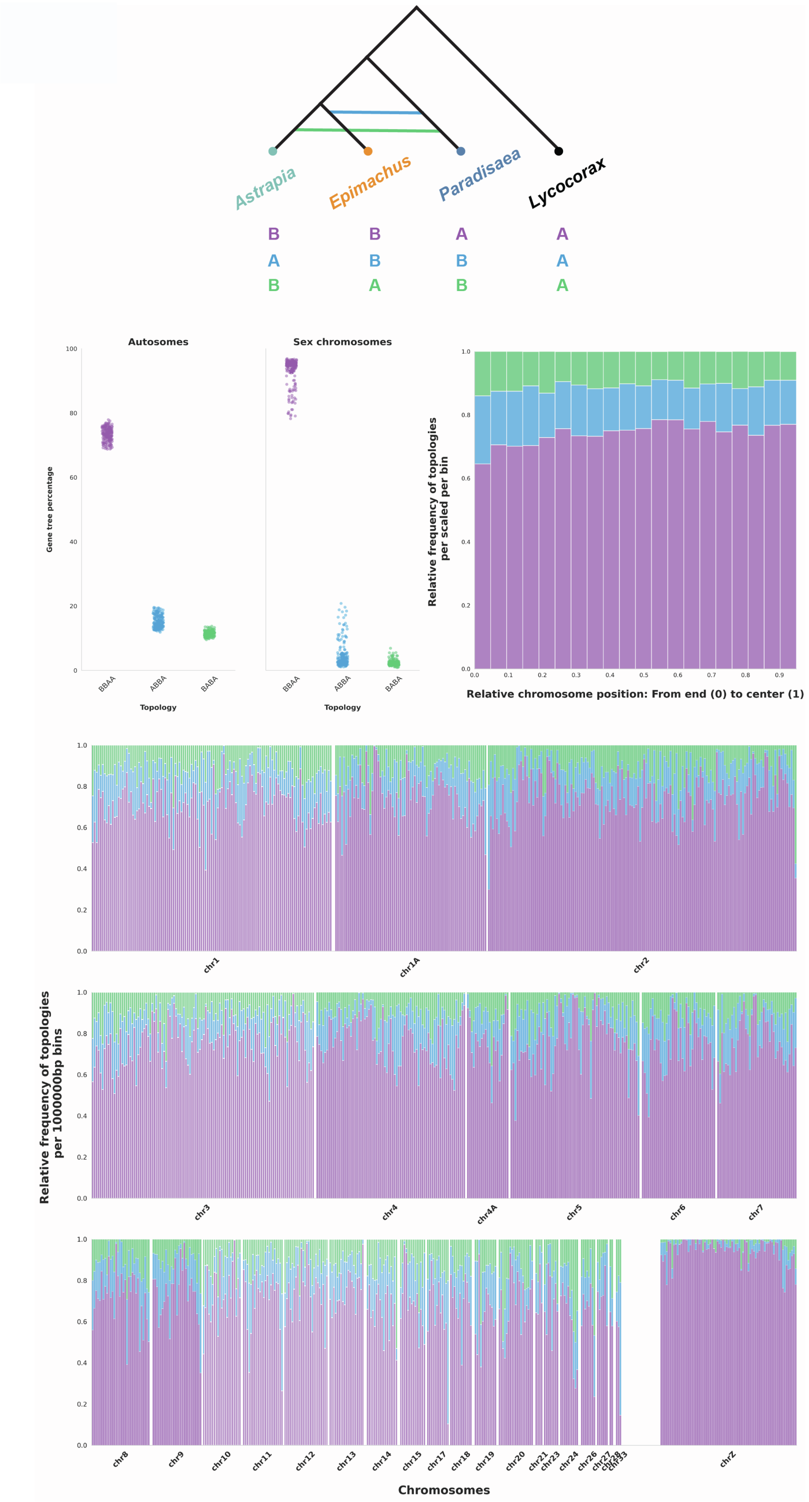

Figure S104. Frequency and genome wide distribution of window tree topologies for each possible Cartesian combination of individuals across four genera. Related to Figure 2 & S101-107. The top-left figure has the relative frequency of ABBA, BABA and BBAA topologies plotted for each of the Cartesian combinations, the top right figure has the distribution of topologies plotted by scaled chromosome position (10 macrochromosomes, summed across all Cartesian combinations) and the bottom plot illustrates the distribution of topologies in bins of 1 Mb. for all chromosomes (summed across all Cartesian combinations). Besides the species tree (BBAA), there is an almost equal proportion of trees where *Epimachus* and *Paradisaea* are clustered (ABBA), relative to trees where *Astrapia* and *Paradisaea* are clustered (BABA).

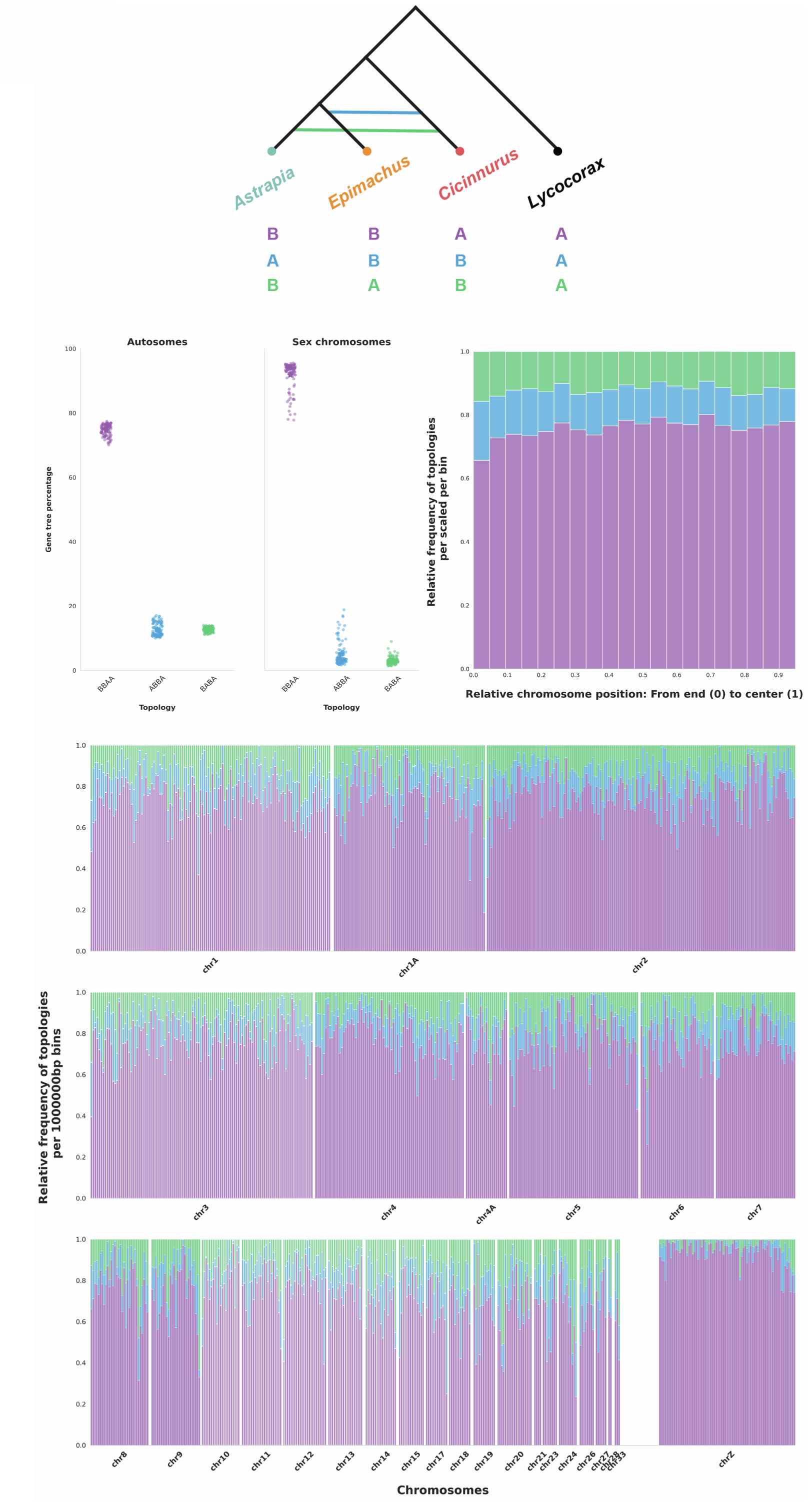

Figure S105. Frequency and genome wide distribution of window tree topologies for each possible Cartesian combination of individuals across four genera. Related to Figure 2 & S101-107. The top-left figure has the relative frequency of ABBA, BABA and BBAA topologies plotted for each of the Cartesian combinations, the top right figure has the distribution of topologies plotted by scaled chromosome position (10 macrochromosomes, summed across all Cartesian combinations) and the bottom plot illustrates the distribution of topologies in bins of 1 Mb. for all chromosomes (summed across all Cartesian combinations). Besides the species tree (BBAA), there is an equal proportion of trees where *Epimachus* and *Cicinnurus/Diphyllodes* are clustered (ABBA), relative to trees where *Astrapia* and *Cicinnurus/Diphyllodes* are clustered (BABA).

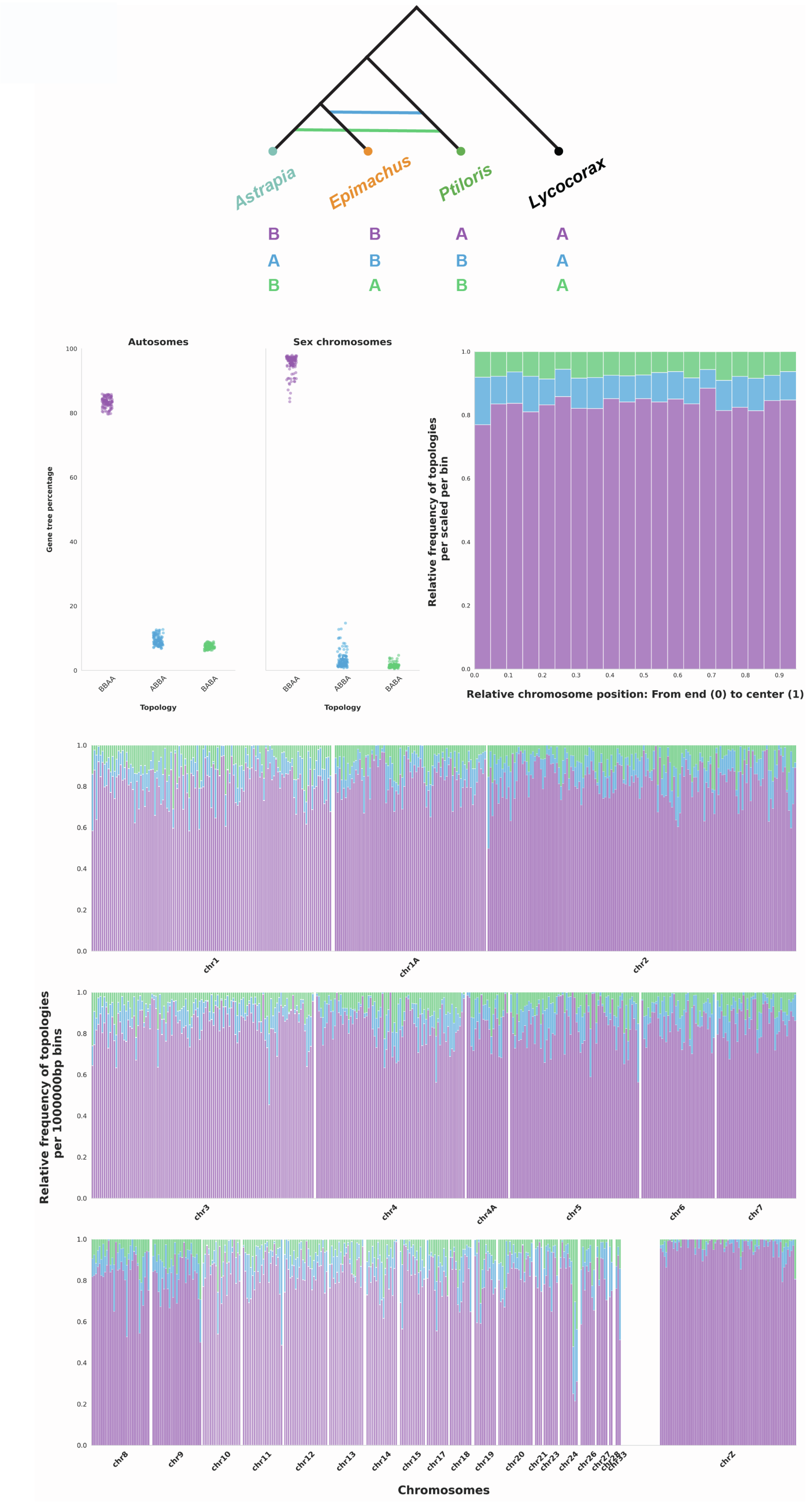

Figure S106. Frequency and genome wide distribution of window tree topologies for each possible Cartesian combination of individuals across four genera. Related to Figure 2 & S101-107. The top-left figure has the relative frequency of ABBA, BABA and BBAA topologies plotted for each of the Cartesian combinations, the top right figure has the distribution of topologies plotted by scaled chromosome position (10 macrochromosomes, summed across all Cartesian combinations) and the bottom plot illustrates the distribution of topologies in bins of 1 Mb. for all chromosomes (summed across all Cartesian combinations). Besides the species tree (BBAA), there is an equal proportion of trees where *Epimachus* and *Ptiloris/Lophorina* are clustered (ABBA), relative to trees where *Astrapia* and *Ptiloris/Lophorina* are clustered (BABA).

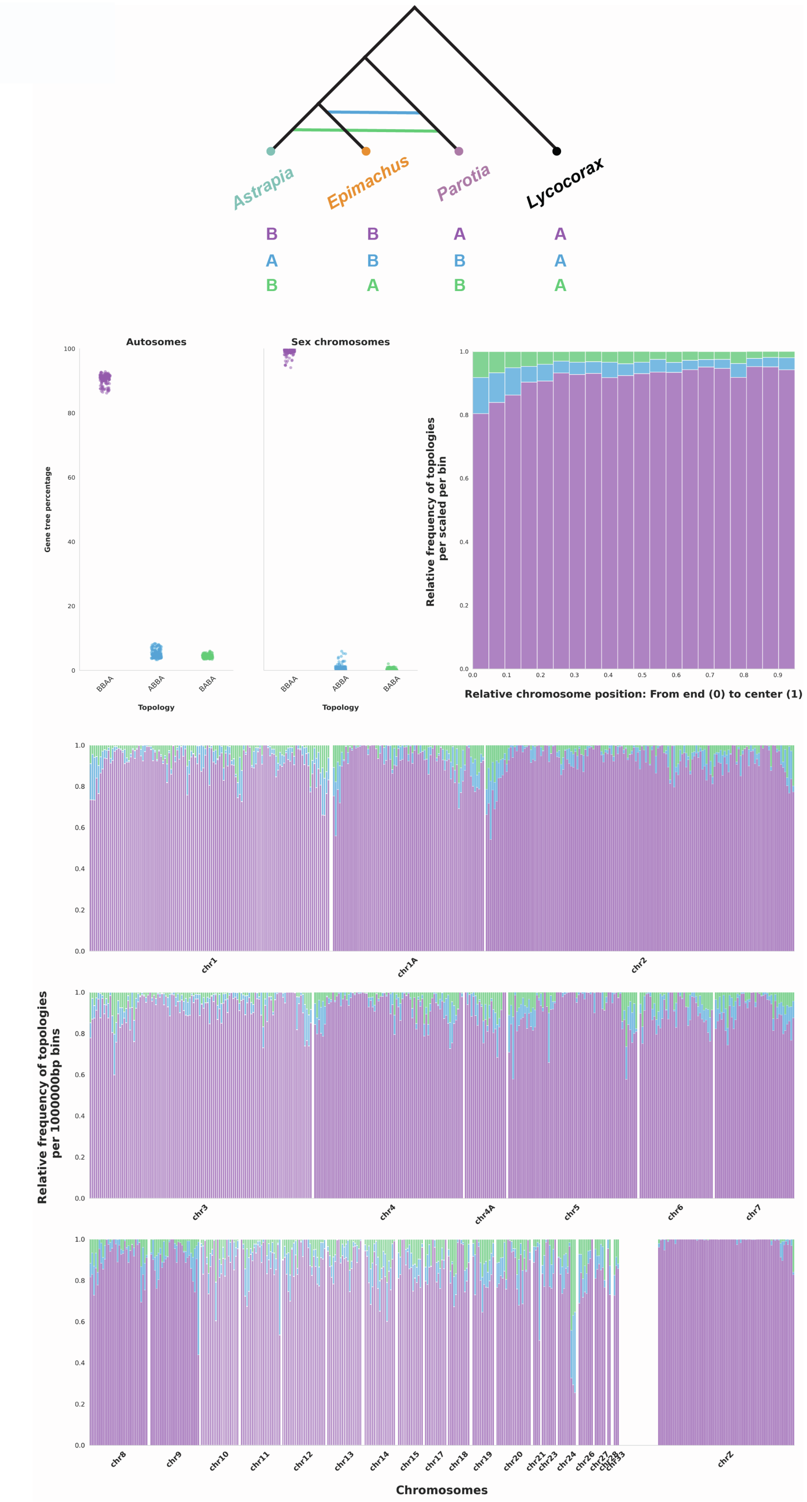

Figure S107. Frequency and genome wide distribution of window tree topologies for each possible Cartesian combination of individuals across four genera. Related to Figure 2 & S101-106. The top-left figure has the relative frequency of ABBA, BABA and BBAA topologies plotted for each of the Cartesian combinations, the top right figure has the distribution of topologies plotted by scaled chromosome position (10 macrochromosomes, summed across all Cartesian combinations) and the bottom plot illustrates the distribution of topologies in bins of 1 Mb. for all chromosomes (summed across all Cartesian combinations). Besides the species tree (BBAA), there is an equal proportion of trees where *Epimachus* and *Parotia* are clustered (ABBA), relative to trees where *Astrapia* and *Parotia* are clustered (BABA).

Table S1. Metadata for all individuals belonging to pure species, including historical information and sequencing yield, related to STAR Methods

Study ID	Genus	Species	Specimen ID	tissue type	Sex	Lat	Long	Province	Country	Lek type	number of reads	number of mapped reads	number of mapped r	mean coverage
AmayM662	Astrapia	mayeri	NRM 569662	SKIN	F	-5,77	144,00	Western Highlands	Papua New Guinea	classic.lek, canopy	364,927,547	300,849,123	82.44	17.3393X
AnigM602	Astrapia	nigra	NRM 551602	SKIN	M	-0,83	132,83	Southwest Papua	Indonesia	exp.lek, canopy	104,377,020	96,471,019	92.43	8.0625X
ArotM245	Astrapia	rothschildi	KU 93602	FRESH	M	-6,10	146,56	Morobe	Papua New Guinea	exp.lek, canopy	731,410,698	708,944,956	96.93	85.4729X
AsplM961	Astrapia	splendidissima	NRM 569961	SKIN	F	-3,58	138,33	Central Papua	Indonesia	classic.lek, canopy	332,034,403	292,228,695	88.01	21.3966X
AsteF677	Astrapia	stephaniae	NRM 551677	SKIN	F	-5,82	144,84	Chimbu	Papua New Guinea	classic.lek, canopy	197,694,889	138,216,300	69.91	9.9217X
CmagF569	Diphyllodes	magnificus	ANWC B27061, MV Z43666	FRESH	F	-8,88	147,73	Oro	Papua New Guinea	exp.lek, ground	807,192,574	788,271,564	97.66	103.6172X
CmagM094	Diphyllodes	magnificus	ANWC B26499, MV Z43634	FRESH	M	-9,15	147,67	Central	Papua New Guinea	exp.lek, ground	134,632,662	132,610,712	98.5	14.56X
CregF628	Cicinnurus	regius	ANWC B24969, MV Z43628	FRESH	F?	-9,08	147,18	Central	Papua New Guinea	exp.lek, canopy	157,052,955	151,261,674	96.31	22.728X
CregM702	Cicinnurus	regius	ANWC B18442, MV Z43721	FRESH	M	-9,03	147,05	Central	Papua New Guinea	exp.lek, canopy	153,382,163	151,190,366	98.57	16.5845X
CresM771	Diphyllodes	respublica	NRM 566771	SKIN	M	-0,87	130,65	Southwest Papua	Indonesia	exp.lek, ground	110,212,992	98,177,487	89.08	7.6464X
DalbM148	Drepanornis	albertisi	ANWC B26506, MV Z43612	FRESH	U	-9,15	147,67	Central	Papua New Guinea	solitary, understory	158,718,828	156,389,378	98.53	17.1866X
DbruM863	Drepanornis	bruijnii	NRM 554863	SKIN	M	-2,55	140,70	Papua	Indonesia	solitary, canopy	334,794,640	305,178,329	91.15	27.7848X
EfasM199	Epimachus	fastosus	NRM 551599	SKIN	M	-1,35	133,95	West Papua	Indonesia	exp.lek, understory	243,505,852	227,427,014	93.4	25.5566X
EmeyM165	Epimachus	meyeri	ANWC B26803, MV Z43689	FRESH	M	-9,18	148,03	Oro	Papua New Guinea	solitary, canopy	143,917,413	141,525,832	98.34	15.5651X
LpyrF013	Lycocorax	obiensis	MZB-34.073	FRESH	F	-1,63	127,74	North Maluku	Indonesia	solitary, understory	156,015,676	154,245,354	98.87	21.613X
LpyrM013	Lycocorax	obiensis	MZB-34.075	FRESH	M	-1,63	127,74	North Maluku	Indonesia	solitary, understory	473,432,840	463,704,819	97.95	51.1856X
LsupM270	Lophorina	minor	ANWC B26603, MV Z43663	FRESH	M	-9,18	148,03	Oro	Papua New Guinea	solitary, ground	162,196,508	159,963,372	98.62	17.5695X
MateM282	Manucodia	ater	KU 98036	FRESH	M	-6,98	145,06	Gulf	Papua New Guinea	solitary, understory	161,002,764	158,901,062	98.69	17.7311X
MchaM019	Manucodia	chalybatus	ANWC B26698, MV Z43744	FRESH	M	-9,17	148,08	Oro	Papua New Guinea	solitary, understory	177,375,597	175,535,189	98.96	19.5268X
McomM485	Manucodia	comrii	RMNH.AVES.140485	SKIN	M	-9,52	150,67	Milne Bay	Papua New Guinea	solitary, understory	317,964,030	248,112,742	78.03	16.7094X
MjobM355	Manucodia	jobiensis	BMNH 1916.5.30.1355	SKIN	F	-4,70	136,92	Central Papua	Indonesia	solitary, understory	101,566,019	87,668,774	86.32	6.8787X
PalbM458	Pteridophora	alberti	NRM 571458	SKIN	M	-5,80	144,79	Jiwaka	Papua New Guinea	solitary, understory	198,355,584	180,939,961	91.22	14.3023X
PapoM493	Paradisaea	apoda	ZMUC 64.493	SKIN	M	Captivity				classic.lek, canopy	231,985,530	223,631,541	96.4	28.4969X
PberM170	Parotia	berlepschi	AMNH 678170	SKIN	M	-2,58	139,00	Papua	Indonesia	exp.lek, ground	309,610,654	262,169,627	84.68	16.8359X
PbreM721	Paradigalla	brevicauda	KU 114260	FRESH	M	-7,06	145,82	East Highlands	Papua New Guinea	solitary, understory	129,577,180	127,280,368	98.23	13.9763X
PcarM325	Parotia	carolae	KU 93605	FRESH	M	-6,60	145,20	East Highlands	Papua New Guinea	exp.lek, ground	180,332,992	177,663,356	98.52	19.465X
PcarM706	Paradigalla	carunculata	BMNH 81.5.1.1706	SKIN	M	-1,08	133,97	West Papua	Indonesia	solitary, understory	237,322,032	68,904,784	29.03	4.7938X
PdecM421	Paradisaea	decora	BMNH 1936.4.2.1	SKIN	M	-9,52	150,67	Milne Bay	Papua New Guinea	classic.lek, canopy	111,938,507	97,677,136	87.26	7.9779X
PguiM185	Paradisaea	guilielmi	BMNH 1921.12.30.185	SKIN	M	-6,52	147,32	Morobe	Papua New Guinea	classic.lek, canopy	356,073,587	285,350,675	80.14	19.7502X
PkerM895	Phonygammus	keraudrenii	ANWC B39848, MV Z38660	FRESH	M	-13,80	143,45	Queensland	Australia	solitary, understory	158,265,909	156,766,345	99.05	17.4514X
PlawF631	Parotia	helenae	ANWC B26535, MV Z43631	FRESH	F	-9,15	147,67	Central	Papua New Guinea	exp.lek, ground	478,603,448	468,948,448	97.98	60.0798X
PlawM320	Parotia	helenae	ANWC B15265, MV Z43698	FRESH	M	-9,15	147,67	Oro	Papua New Guinea	exp.lek, ground	785,155,761	766,374,873	97.61	90.8878X
PmagM227	Ptiloris	intercedens	ANWC B24968, MV Z43615	FRESH	F	-9,07	147,17	Central	Papua New Guinea	solitary, understory	134,440,304	132,547,580	98.59	14.5415X
PmagM784	Ptiloris	magnificus	ANWC B39736, MV Z38082	FRESH	M	-13,70	143,35	Queensland	Australia	solitary, understory	118,931,512	117,282,173	98.61	12.8752X
PminF635	Paradisaea	minor	NRM 551635	SKIN	F	-3,37	133,33	West Papua	Indonesia	classic.lek, canopy	308,919,890	292,932,354	94.82	30.0001X
PminM226	Paradisaea	minor	NRM 700226	SKIN	M	-1,43	134,00	West Papua	Indonesia	classic.lek, canopy	89,862,878	84,966,721	94.55	8.7109X
PparM271	Ptiloris	paradiseus	ANWC B43271	FRESH	M	-30,21	152,99	New South Wales	Australia	solitary, understory	541,862,754	525,845,547	97.04	49.1691X
PragM588	Paradisaea	raggiana	ANWC B26706, MV Z43750	FRESH	M	-9,17	148,08	Oro	Papua New Guinea	classic.lek, canopy	190,453,344	187,687,927	98.55	20.6318X
PrubF686	Paradisaea	rubra	YPM 84686	FRESH	F	Captivity				classic.lek, canopy	460,498,078	449,721,089	97.66	58.8134X
PrubM233	Paradisaea	rubra	NRM 700233	SKIN	M	-0,87	130,65	Southwest Papua	Indonesia	classic.lek, canopy	200,481,884	182,672,648	91.12	15.2955X
PrudM124	Paradisornis	rudolphi	ZMUC 145.124	SKIN	M	-5,17	142,67	Hela	Papua New Guinea	solitary, understory	280,441,934	258,801,437	92.28	24.1677X
PsefM840	Parotia	sefilata	NRM 571840	SKIN	M	-1,43	134,00	West Papua	Indonesia	exp.lek, ground	95,372,119	91,415,261	95.85	9.781X
PvicM967	Ptiloris	victoriae	ANWC B34967	FRESH	F	-17,27	145,64	Queensland	Australia	solitary, understory	155,619,517	153,630,974	98.72	16.8922X
PwahM286	Parotia	wahnesi	KU 93603	FRESH	M	-6,10	146,59	Morobe	Papua New Guinea	exp.lek, ground	197,647,184	194,604,153	98.46	21.34X
SmelM057	Seleucidis	melanoleucus	NRM 552057	SKIN	M	-3,37	133,33	West Papua	Indonesia	solitary, canopy	385,557,758	368,827,896	95.66	43.8122X
SwalM739	Semioptera	wallacii	BMNH 1969.27.739	SKIN	M	-0,56	127,50	North Maluku	Indonesia	classic.lek, canopy	289,761,230	268,581,407	92.69	27.8369X

Table S2. Metadata for 10 historical specimens of putative hybrid origin, including historical information and sequencing yield, related to STAR Methods

Study ID	Species (Maternal - mitochondrial placement)	Species (Paternal)	Specimen ID	tissue type	Sex	Locality	number of reads	number of mapped reads	number of mapped reads in percentage	mean coverage
EpiAst118	Astrapia nigra (Morphology: Epimachus fastosus	Paradigalla carunculata	AMNH 679118	SKIN	M	Dutch NG, presumably Vogelkop	238,210,865	226,346,139	95.02	24.6411X
EpiAst119	Astrapia nigra	Epimachus fastosus	AMNH 679119	SKIN	M	Dutch NG, Vogelkop	121,103,843	74,914,625	61.86	8.3389X
LopAst113	Lophorina (superba) niedda	Paradigalla carunculata	AMNH 679113	SKIN	M	Dutch NG, presumably Vogelkop	169,167,659	157,927,222	93.36	16.4751X
LopCic115	Diphyllodes magnificus	Lophorina superba	AMNH 679115	SKIN	M	Dutch NG	245,800,512	237,038,691	96.44	27.2018X
LopPti116	Ptiloris magnificus	Lophorina superba	AMNH 679116	SKIN	M	British NG	209,567,444	197,983,819	94.47	20.539X
ParLop117	Parotia sefilata	Lophorina (superba) niedda	AMNH 679117	SKIN	M	Dutch NG	191,287,250	182,064,897	95.18	18.4544X
SelLop109	Seleucidis melanoleucus	Ptiloris magnificus	AMNH 679109	SKIN	M	Unknown	228,283,422	217,668,980	95.35	26.0598X
SelLop112	Seleucidis melanoleucus	Ptiloris magnificus	AMNH 679112	SKIN	M	Unknown	209,130,722	198,069,526	94.71	21.8331X
SelPar100	Seleucidis melanoleucus	Paradisaea minor	AMNH 679100	SKIN	M	Near Kaiser Wilhelmshafen, German NG	201,667,383	181,845,693	90.17	19.4906X
SelPar101	Seleucidis melanoleucus	Paradisaea minor	AMNH 679101	SKIN	M	Unknown	173,510,577	128,313,107	73.95	14.2732X

Maternal species as identified based on the placement in mitochondrial phylogeny (Supp. Fig. 1). The species designation is listed as in museum records; i.e. based on morphology, except for EpiAst118 where the maternal species was clearly misidentified.
